# Development of Proteomic Prediction Models for Transition to Psychotic Disorder in the Clinical High-Risk State and Psychotic Experiences in Adolescence

**DOI:** 10.1001/jamapsychiatry.2020.2459

**Published:** 2020-08-26

**Authors:** David Mongan, Melanie Föcking, Colm Healy, Subash Raj Susai, Meike Heurich, Kieran Wynne, Barnaby Nelson, Patrick D. McGorry, G. Paul Amminger, Merete Nordentoft, Marie-Odile Krebs, Anita Riecher-Rössler, Rodrigo A. Bressan, Neus Barrantes-Vidal, Stefan Borgwardt, Stephan Ruhrmann, Gabriele Sachs, Christos Pantelis, Mark van der Gaag, Lieuwe de Haan, Lucia Valmaggia, Thomas A. Pollak, Matthew J. Kempton, Bart P. F. Rutten, Robert Whelan, Mary Cannon, Stan Zammit, Gerard Cagney, David R. Cotter, Philip McGuire

**Affiliations:** 1 Department of Psychiatry, Royal College of Surgeons in Ireland, Dublin, Ireland; 2 School of Pharmacy and Pharmaceutical Sciences, Cardiff University, Cardiff, United Kingdom; 3 School of Biomolecular and Biomedical Science, Conway Institute, University College Dublin, Dublin, Ireland; 4 Centre for Youth Mental Health, University of Melbourne, Parkville, Victoria, Australia; 5 Mental Health Centre Copenhagen, Copenhagen University Hospital, Copenhagen, Denmark; 6 University Paris Descartes, Groupe Hospitalier Universitaire (GHU) Paris–Sainte Anne, Evaluation Centre for Young Adults and Adolescents (C’JAAD), Service Hospitalov–Universitaire, Institut National de la Santé et de la Recherche Medicale (INSERM) U1266, Institut de Psychiatrie (Centre National de la Recherche Scientifique [CNRS] 3557), Paris, France; 7 Department of Psychiatry, Medical Faculty, University of Basel, Basel, Switzerland; 8 LiNC–Lab Interdisciplinar Neurociências Clínicas, Depto Psiquiatria, Escola Paulista de Medicina, Universidade Federal de São Paulo (UNIFESP), São Paulo, Brazil; 9 Departament de Psicologia Clínica i de la Salut (Universitat Autònoma de Barcelona), Fundació Sanitària Sant Pere Claver (Spain), Spanish Mental Health Research Network (Centro de Investigación Biomédica en Red de Salud Mental [CIBERSAM]), Barcelona, Spain; 10 Department of Psychiatry and Psychotherapy, Translational Psychiatry Unit, University zu Lübeck, Lübeck, Germany; 11 Department of Psychiatry and Psychotherapy, Faculty of Medicine and University Hospital Cologne, University of Cologne, Cologne, Germany; 12 Department of Psychiatry and Psychotherapy, Medical University of Vienna, Vienna, Austria; 13 Melbourne Neuropsychiatry Centre, Department of Psychiatry, University of Melbourne and Melbourne Health, Carlton South, Victoria, Australia; 14 Faculty of Behavioural and Movement Sciences, Department of Clinical Psychology and EMGO+ Institute for Health and Care Research, Vrije Universiteit (VU) University, Amsterdam, the Netherlands; 15 Department of Psychosis Research, Parnassia Psychiatric Institute, The Hague, the Netherlands; 16 Academic Medical Centre (AMC), Academic Psychiatric Centre, Department Early Psychosis, Amsterdam, the Netherlands; 17 Institute of Psychiatry, Psychology & Neuroscience, Department of Psychology, King’s College London, London, United Kingdom; 18 Institute of Psychiatry, Psychology & Neuroscience, Department of Psychosis Studies, King’s College London, London, United Kingdom; 19 Department of Psychiatry and Neuropsychology, School for Mental Health and Neuroscience, Maastricht University Medical Centre, Maastricht, the Netherlands; 20 Trinity Institute of Neuroscience, Trinity College Dublin, Dublin, Ireland; 21 Medical Research Council (MRC) Centre for Neuropsychiatric Genetics and Genomics, Cardiff University, Cardiff, United Kingdom; 22 Bristol Medical School, University of Bristol, Bristol, United Kingdom

## Abstract

**Importance:**

Biomarkers that are predictive of outcomes in individuals at risk of psychosis would facilitate individualized prognosis and stratification strategies.

**Objective:**

To investigate whether proteomic biomarkers may aid prediction of transition to psychotic disorder in the clinical high-risk (CHR) state and adolescent psychotic experiences (PEs) in the general population.

**Design, Setting, and Participants:**

This diagnostic study comprised 2 case-control studies nested within the European Network of National Schizophrenia Networks Studying Gene-Environment Interactions (EU-GEI) and the Avon Longitudinal Study of Parents and Children (ALSPAC). EU-GEI is an international multisite prospective study of participants at CHR referred from local mental health services. ALSPAC is a United Kingdom–based general population birth cohort. Included were EU-GEI participants who met CHR criteria at baseline and ALSPAC participants who did not report PEs at age 12 years. Data were analyzed from September 2018 to April 2020.

**Main Outcomes and Measures:**

In EU-GEI, transition status was assessed by the Comprehensive Assessment of At-Risk Mental States or contact with clinical services. In ALSPAC, PEs at age 18 years were assessed using the Psychosis-Like Symptoms Interview. Proteomic data were obtained from mass spectrometry of baseline plasma samples in EU-GEI and plasma samples at age 12 years in ALSPAC. Support vector machine learning algorithms were used to develop predictive models.

**Results:**

The EU-GEI subsample (133 participants at CHR (mean [SD] age, 22.6 [4.5] years; 68 [51.1%] male) comprised 49 (36.8%) who developed psychosis and 84 (63.2%) who did not. A model based on baseline clinical and proteomic data demonstrated excellent performance for prediction of transition outcome (area under the receiver operating characteristic curve [AUC], 0.95; positive predictive value [PPV], 75.0%; and negative predictive value [NPV], 98.6%). Functional analysis of differentially expressed proteins implicated the complement and coagulation cascade. A model based on the 10 most predictive proteins accurately predicted transition status in training (AUC, 0.99; PPV, 76.9%; and NPV, 100%) and test (AUC, 0.92; PPV, 81.8%; and NPV, 96.8%) data. The ALSPAC subsample (121 participants from the general population with plasma samples available at age 12 years (61 [50.4%] male) comprised 55 participants (45.5%) with PEs at age 18 years and 61 (50.4%) without PEs at age 18 years. A model using proteomic data at age 12 years predicted PEs at age 18 years, with an AUC of 0.74 (PPV, 67.8%; and NPV, 75.8%).

**Conclusions and Relevance:**

In individuals at risk of psychosis, proteomic biomarkers may contribute to individualized prognosis and stratification strategies. These findings implicate early dysregulation of the complement and coagulation cascade in the development of psychosis outcomes.

## Key Points

**Question:**

Can plasma proteomic biomarkers aid prediction of transition to psychotic disorder in people at clinical high risk (CHR) of psychosis and adolescent psychotic experiences in the general population?

**Findings:**

In this diagnostic study of 133 individuals at CHR in EU-GEI and 121 individuals from the general population in ALSPAC, models were developed based on baseline proteomic data, with excellent predictive performance for transition to psychotic disorder in individuals at CHR. In a general population sample, models based on proteomic data at age 12 years had fair predictive performance for psychotic experiences at age 18 years.

**Meaning:**

Predictive models based on proteomic biomarkers may contribute to personalized prognosis and stratification strategies in individuals at risk of psychosis.

## Teaser

This diagnostic study investigates whether proteomic biomarkers may aid the prediction of transition to psychotic disorder in the clinical high-risk state and adolescent psychotic experiences in the general population.

## Introduction

Early detection of psychosis may improve clinical outcomes.^1^ Clinical high-risk (CHR) criteria^2^ enable identification of vulnerable groups with 3-year transition rates to first-episode psychosis (FEP) of 16% to 35%.^3^ However, it is difficult to predict outcomes individually. Previous studies have also characterized an extended psychosis phenotype that includes individuals with psychotic experiences (PEs).^4^ These subthreshold symptoms are associated with an increased risk of psychotic and nonpsychotic disorders^5^ and reduced global functioning.^6^

Biomarkers may augment prognosis and stratification strategies.^7^ We aimed to compare plasma protein expression in individuals at CHR who do and do not develop psychosis and to develop models incorporating proteomic data for individualized prediction of transition to FEP. This study also aimed to apply similar methods for prediction of PEs in a general population sample.

## Methods

Ethical approval for this diagnostic study was granted by the Royal College of Surgeons in Ireland. Ethics committees of participating sites granted approval for the European Network of National Schizophrenia Networks Studying Gene-Environment Interactions (EU-GEI). Approval was also obtained from the Avon Longitudinal Study of Parents and Children (ALSPAC) Ethics and Law Committee and local research ethics committees. Informed consent for collection of biological samples was obtained in accordance with the Human Tissue Act 2004.^8^ Informed consent for use of questionnaire and clinic data was obtained following recommendations of the ALSPAC Ethics and Law Committee at the time.

### Study 1: CHR Sample

#### Participants and Study Design

EU-GEI study includes a prospective cohort of 344 participants at CHR recruited across 11 international sites.^9,10^ Individuals with CHR symptoms who were referred by local mental health services were eligible to participate if they met CHR criteria according to the Comprehensive Assessment of At-Risk Mental States^11^ (CAARMS) and provided written informed consent. Exclusion criteria were current or past psychotic disorder, symptoms explained by a medical disorder or drug or alcohol use, and IQ less than 60.

Plasma samples were obtained at baseline, and clinical assessments were performed at baseline, 12 months, and 24 months. After 24 months, or if a face-to-face interview was not possible, attempts were made to confirm transition status via the clinical team or records. Assessors were not systematically blinded to transition status because, in some cases, clinical services contacted the research team in advance to advise that transition had occurred. Accrual began in September 2010. The last baseline assessment was performed in July 2015.

**Figure 1.**
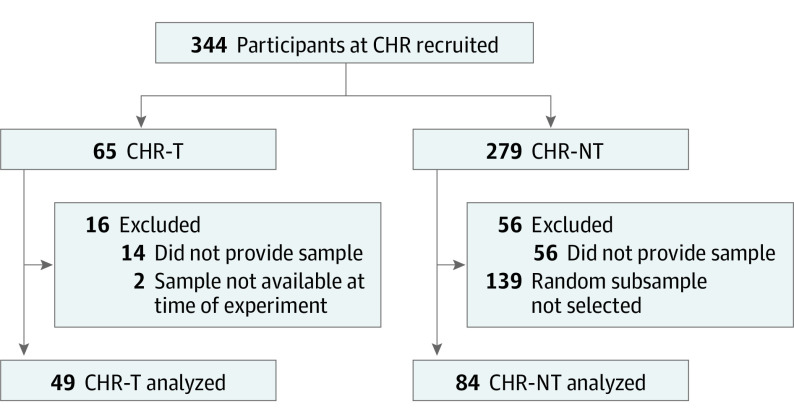
Derivation of Participants Included in the Initial EU-GEI Mass Spectrometry Experiment and Their Provision of Plasma Samples CHR indicates clinical high risk; CHR-NT, participants at clinical high risk who did not transition to psychosis; CHR-T, participants at clinical high risk who transitioned to first-episode psychosis; and EU-GEI, European Network of National Schizophrenia Networks Studying Gene-Environment Interactions.

The present investigation comprised a nested case-control study comparing plasma proteins from participants at CHR who transitioned to psychosis on follow-up (CHR-T) (n = 49) with a control group of randomly selected participants who did not (CHR-NT) (n = 84) (Figure 1). Based on previous experience,^12^ the experiment was limited to this number to ensure optimal technical performance across mass spectrometry runs.

#### Outcome and Clinical Measures

Transition was defined as the onset of nonorganic psychotic disorder as assessed either by CAARMS interview^11^ or by contact with the clinical team or review of clinical records. Sixty-five of 344 participants at CHR (18.9%) developed psychosis on follow-up, 57 within 24 months and 8 after 24 months.

Baseline clinical measures were recorded. These included age, sex, body mass index (BMI; calculated as weight in kilograms divided by height in meters squared), years of education, General Assessment of Functioning (GAF) subscales for symptoms and disability,^13,14^ the Scale for the Assessment of Negative Symptoms (SANS),^15^ the Brief Psychiatric Rating Scale (BPRS),^16^ and the Montgomery-Åsberg Depression Rating Scale (MADRS).^17^

#### Sample Preparation, Proteomics, Validation, and Replication

Laboratory procedures were conducted blind to case-control status. Protein depletion, digestion, and peptide purification were performed using baseline plasma samples. Discovery-based proteomic methods were used.^12^ Briefly, 5 μL from each prepared sample was injected on a Q Exactive (Thermo Scientific) mass spectrometer operated in data-dependent acquisition mode for label-free liquid chromatography mass spectrometry^12,18,19,20^ (eMethods in Supplement 1 and eAppendix in Supplement 2).

Nine proteins in plasma samples from the same participants at CHR described above (Figure 1) were assessed using enzyme-linked immunosorbent assay (ELISA). Details are available in eMethods in Supplement 1.

In an effort to reproduce our findings, we conducted a partial replication of the initial mass spectrometry experiment by analyzing baseline plasma samples from 49 CHR-T cases (2 of these cases were different from the initial experiment) and an entirely new group of 86 CHR-NT control cases. Details are available in eMethods in Supplement 1.

### Study 2: General Population Sample

#### Participants and Study Design

The ALSPAC is a prospective birth cohort.^21,22,23^ Pregnant women in Avon, United Kingdom, with delivery dates between April 1, 1991, and December 31, 1992, were invited to participate, and 14 541 pregnancies were enrolled. When the oldest children were approximately age 7 years, an attempt was made to bolster the sample with children who did not join originally. The sample size for analyses using data from age 7 years is 15 454 pregnancies (14 901 children alive at 1 year).

Plasma samples obtained at age 12 years from ALSPAC participants who did or did not report PEs at age 18 years were previously investigated.^12,20^ In data-independent acquisition analyses focused on proteins of the complement pathway, several proteins were differentially expressed. Herein, we performed data-dependent acquisition analyses (rather than data-independent acquisition) in this sample to achieve broader proteome coverage.

#### Outcome, Sample Preparation, and Proteomics

Psychotic experiences were assessed in participants at age 12 years and age 18 years using the Psychosis-Like Symptoms Interview^4^ and were rated as not present, suspected, or definite. Of 4060 participants assessed at both time points, 190 (4.7%) had suspected or definite PEs at age 18 years but not at age 12 years.^4^ The present study was based on a subsample of case participants (who did not report PEs at age 12 years but reported at least 1 definite PE at age 18 years) and randomly selected control participants (who did not report PEs at either age 12 years or age 18 years).

Plasma samples at age 12 years were prepared as previously described.^12^ Data-dependent acquisition proteomic analyses were performed as for study 1.

### Data and Statistical Analyses

Data were analyzed from September 2018 to April 2020. Clinical data were tested for differences using the 2-sided *t* test for continuous variables and χ^2^ test for categorical variables in SPSS, version 25 (IBM). *P* values were corrected for multiple comparisons using the Benjamini-Hochberg procedure^24^ with a 5% false discovery rate (FDR). The threshold for statistical significance was FDR-corrected *P* < .05.

Label-free quantification was performed in MaxQuant, version 1.5.2.8 (Max Planck Institute of Biochemistry).^25,26^ Proteins identified with at least 2 peptides (1 uniquely assignable to the protein) and quantified in more than 80% of samples were taken forward for analysis and log_2_ transformed. Missing values were imputed using imputeLCMD (version 2.0)^27^ in RStudio.^28^ Label-free quantification values were converted to *z* scores and winsorized within ±3 *z*.

Analysis of covariance was performed in Stata, version 15 (StataCorp LLC), comparing the mean label-free quantification for each protein in cases and controls. Covariates included age, sex, BMI, and years of education in study 1 and sex, BMI at age 12 years, and maternal social class in study 2. *P* values were corrected for multiple comparisons with a 5% FDR.

### Predictive Models

Neurominer, version 1.0, for MatLab 2018a (MathWorks Inc) was used to develop support vector machine (SVM) models (eMethods in Supplement 1). The development of each model is summarized in eTable 1 in Supplement 1.

#### Models 1a-c: Predicting Transition Using Clinical and Proteomic Data

First, we developed a model predicting transition using clinical and proteomic data together (model 1a). eTable 2 in Supplement 1 lists the included clinical features. Geographical generalizability was incorporated using leave-site-out cross-validation (eMethods in Supplement 1) as recommended for multisite consortia.^29^ To assess the relative contribution of clinical and proteomic data, we next developed models using the same cross-validation and training framework but based on clinical (model 1b) and proteomic (model 1c) features separately.

#### Model 2a and b: Parsimonious Model

We sought to generate a parsimonious model based on the 10 highest-weighted proteomic predictors and internally validate this model in unseen data (eFigure 1 in Supplement 1). As the largest site, London, United Kingdom, was chosen as the test site, and data for these participants were held out.

To derive the 10 highest-weighted proteins, a model (model 2a) was generated using proteomic data from all sites except London (n = 30 for CHR-T and n = 50 for CHR-NT). A reduced model was then developed based solely on data for these 10 proteins in the non-London data set (model 2b) and then tested in the held-out London data (n = 19 for CHR-T and n = 34 for CHR-NT). Both models used leave-site-out cross-validation.

#### Model 3: Replication

Because of differences in protein identifications, it was not possible to apply models 1a-c and 2a-b to the replication data set. We instead sought to replicate our initial findings by performing a second discovery analysis, generating a new model (with leave-site-out cross-validation) predicting transition based on clinical and proteomic data in the replication data set.

#### Model 4: Predicting PEs Using Proteomic Data

We developed a model predicting PEs at age 18 years in the ALSPAC based on proteomic data at age 12 years. Repeated nested cross-validation with 5 inner folds and 5 outer folds was used.

### Supplementary Analyses

Several supplementary analyses (eMethods in Supplement 1) were performed. These included the following: the development of a model predicting transition in EU-GEI based on ELISA data (model S1), the development of a model predicting functional outcome in EU-GEI (GAF disability subscale score ≤60 [poor functional outcome] vs >60 [good functional outcome] at 24 months) based on clinical and proteomic data (model S2), investigation of potential EU-GEI site associations for clinical and proteomic data, and the development of multivariate-corrected versions of SVM models whereby the variance associated with multiple covariates was extracted using principal components analysis.

## Results

### Study 1: CHR Sample

Of 344 participants at CHR who were recruited, 152 (44.2%) attended face-to-face interviews at 12 months and 105 (30.5%) at 24 months. Baseline characteristics of participants who did or did not attend at least 1 follow-up interview are compared in eTable 3 in Supplement 1. After FDR correction, participants who attended interviews had a mean of 1 more year of education and a lower mean SANS total global score than those who did not attend interviews but were otherwise comparable.

The subsample for the initial experiment comprised 133 (49 CHR-T and 84 CHR-NT) participants with baseline plasma samples available, of whom 49 (36.8%) developed psychosis (Figure 1). The mean (SD) age of the participants was 22.6 (4.5) years; 68 participants (51.1%) were male. After FDR correction, participants included in the subsample had a higher mean SANS total composite, SANS total global, and BPRS total scores than nonincluded participants but were otherwise comparable on baseline characteristics (eTable 4 in Supplement 1).

Subsample characteristics are listed in Table 1. After FDR correction, there were no statistically significant group differences for CHR-T vs CHR-NT based on baseline characteristics. The median duration from baseline to transition was 219 days (interquartile range, 424 days). The CHR-T participants had lower mean functional outcome scores at 2 years compared with CHR-NT participants (mean GAF symptoms score at 2 years, 42.3 in CHR-T vs 62.2 in CHR-NT; FDR-corrected *P* < .007; mean GAF disability score at 2 years, 44.7 in CHR-T vs 64.5 in CHR-NT; FDR-corrected *P* < .007).

**Table 1.** Sample Characteristics for CHR-T and CHR-NT Groups in the Initial Experiment

Variable	No. (%)			*t* or χ^2^ Statistic	*P* value	Corrected *P* value (FDR 5%)
	Missing data (n = 133)^a^	CHR-T (n = 49)	CHR-NT (n = 84)			
Baseline age, mean (SD), y	0	22.2 (5.0)	22.9 (4.2)	−0.824	.41	.78
Sex	0					
Male		26 (53.1)	42 (50.0)	0.116	.73	.91
Female		23 (46.9)	42 (50.0)			
Baseline body mass index, mean (SD)	20 (15.0)	24.5 (4.5)	24.4 (6.1)	0.116	.91	.91
Baseline years of education, mean (SD)	14 (10.5)	14.1 (3.4)	14.4 (3.0)	−0.625	.53	.79
Race/ethnicity	0					
White		33 (67.3)	58 (69.0)	2.370	.31	.65
Black		8 (16.3)	7 (8.3)			
Other		8 (16.3)	19 (22.6)			
Ever used cannabis	3 (2.3)					
Yes		36 (73.5)	65 (77.4)	0.051	.82	.91
No		11 (22.4)	18 (21.4)			
Not known		2 (4.1)	1 (1.2)			
Baseline cannabis use	29 (21.8)					
Yes		15 (30.6)	26 (31.0)	0.030	.86	.91
No		22 (44.9)	41 (48.8)			
Not known		12 (24.5)	17 (20.2)			
Baseline tobacco use^b^	14 (10.5)					
Yes		21 (42.9)	43 (51.2)	0.373	.54	.79
No		21 (42.9)	34 (40.5)			
Not known		7 (14.3)	7 (8.3)			
Baseline alcohol use^c^	3 (2.3)					
Yes		35 (71.4)	58 (69.0)	0.071	.79	.91
No		13 (26.5)	24 (28.6)			
Not known		1 (2.0)	2 (2.4)			
Baseline medication use	31 (23.3)					
Yes		19 (38.8)	32 (38.1)	0.042	.84	.91
Antidepressant		13	24			
Antipsychotic		9	6			
Hypnotic		2	6			
Other		3	13			
No		20 (40.8)	31 (36.9)			
Not known		10 (20.4)	21 (25.0)			
Baseline, mean (SD)						
GAF symptoms score	12 (9.0)	52.4 (10.3)	56.0 (10.0)	−1.906	.06	.19
GAF disability score	5 (3.8)	52.3 (12.4)	54.8 (11.3)	−1.148	.25	.60
SANS total composite score	19 (14.3)	20.9 (14.0)	16.2 (11.6)	1.903	.06	.19
SANS total global score	11 (8.3)	6.6 (4.1)	5.8 (3.7)	1.158	.25	.60
BPRS total score	10 (7.5)	49.1 (11.5)	44.2 (10.2)	2.452	.02	.08
MADRS total score	7 (5.3)	20.3 (10.4)	19.2 (9.2)	0.657	.51	.79
GAF symptoms score at 2 y, mean (SD)^d^	62 (46.6)	42.3 (13.2)	62.2 (10.3)	−7.125	<.001	<.007
GAF disability score at 2 y, mean (SD)^e^	54 (40.6)	44.7 (9.1)	64.5 (12.8)	−8.024	<.001	<.007
GAF disability score at 2 y, dichotomous outcome^f^	54 (40.6)					
Poor functioning		29 (59.2)	18 (21.4)	27.734	<.001	<.007
Good functioning		1 (2.0)	31 (36.9)			
Not known		19 (38.8)	35 (41.7)			

Abbreviations: CHR-NT, participants at clinical high risk who did not transition to psychosis; CHR-T, participants at clinical high risk who transitioned to first-episode psychosis; EU-GEI, European Network of National Schizophrenia Networks Studying Gene-Environment Interactions; FDR, false discovery rate; GAF, General Assessment of Functioning; MADRS, Montgomery-Åsberg Depression Rating Scale (high score, greater number and severity of depressive symptoms; low score, lower number and severity of depressive symptoms).

^a^ Missing data were excluded in hypothesis tests.

^b^ Daily tobacco use for at least 1 month over the previous 12 months.

^c^ At least 12 alcoholic beverages over the previous 12 months.

^d^ Data available for 71 of 133 participants (27 CHR-T and 44 CHR-NT).

^e^ Data available for 79 of 133 participants (30 CHR-T and 49 CHR-NT).

^f^ A GAF disability subscale score of 60 or less indicates poor functioning, and a score greater than 60 indicates good functioning.

#### Differential Expression

Of 345 proteins identified, 166 were quantified in more than 80% of plasma samples. There was nominally statistically significant (*P* < .05) differential expression of 56 proteins in CHR-T vs CHR-NT, of which 35 remained statistically significant after FDR correction (eTables 5 and 6 in Supplement 1). eFigure 2 in Supplement 1 shows a functional association network^30^ for these proteins, and eTable 7 in Supplement 1 lists protein-protein interactions. On functional enrichment analysis, the topmost implicated pathway was the complement and coagulation cascade (eTable 8 in Supplement 1).

#### Model 1a: Predicting Transition Using Clinical and Proteomic Data

**Figure 2.**
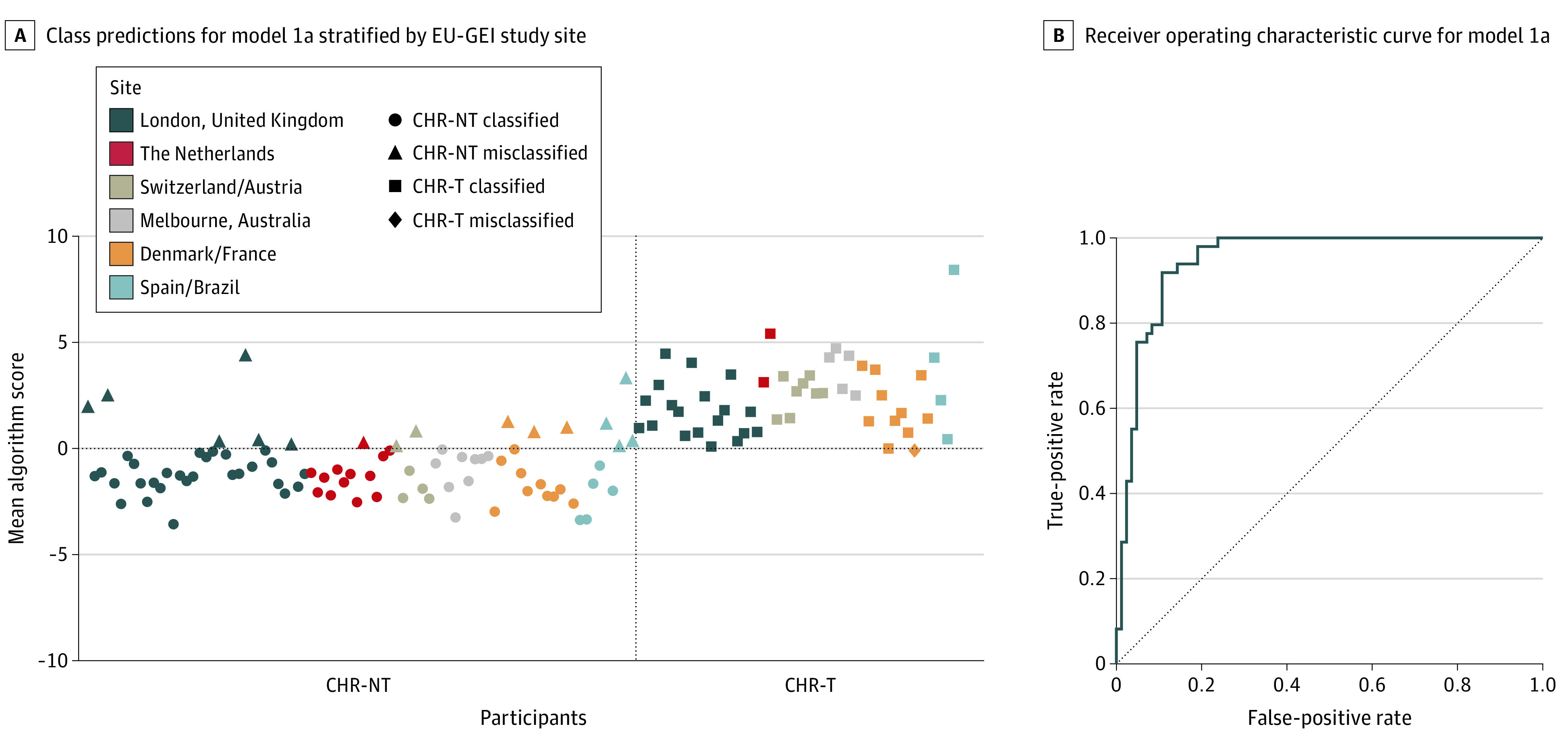
Model 1a Predicting Transition to Psychosis Using Clinical and Proteomic Data A, The algorithm score is a decision score used to determine the predicted outcome class. Herein, a score greater than 0 is assigned as CHR-T, and a score less than 0 is assigned as CHR-NT. The dashed lines divide the graph into quadrants according to predicted vs actual outcome (ie, top right is true positive, bottom left is true negative, top left is false positive, and bottom right is false negative). B, The dashed line is the line of no discrimination (area under the receiver operating characteristic curve, 0.5). CHR-NT indicates participants at clinical high risk who did not transition to psychosis; CHR-T, participants at clinical high risk who transitioned to first-episode psychosis; and EU-GEI, European Network of National Schizophrenia Networks Studying Gene-Environment Interactions.

An SVM model predicted transition status based on clinical and proteomic features (model 1a), with excellent performance (area under the receiver operating characteristic curve [AUC], 0.95; [*P* < .001]; sensitivity, 98.0%; specificity, 81.0%; positive predictive value [PPV], 75.0%; and negative predictive value [NPV], 98.6%). Performance metrics are listed in Table 2. Figure 2A shows the mean algorithm scores and predicted outcomes stratified by site. The receiver operating characteristic curve is shown in Figure 2B. Table 3 lists the 10% highest-weighted features according to the mean feature weight. For example, the 5 highest-ranked predictive features were alpha-2-macroglobulin (A2M) (mean weight, −0.330), immunoglobulin heavy constant mu (IGHM) (mean weight, −0.256), C4b-binding protein alpha chain (C4BPA) (mean weight, −0.161), complement component 8 alpha chain (C8A) (mean weight, 0.158), and phospholipid transfer protein (PLTP) (mean weight, −0.146).

**Table 2.** Performance Metrics for Unadjusted Support Vector Machine Models

Model description	Transition, No./total No. (%)		Nontransition, No./total No. (%)		Sensitivity, %	Specificity, %	Balanced accuracy, %	AUC (95% CI)	PPV, %	NPV, %	Positive likelihood ratio	Negative likelihood ratio
	True positive	False negative	True negative	False positive								
**Model 1a: EU-GEI clinical and proteomic data**^a^ Data set: EU-GEI initial experiment, all sites Features: 69 clinical and 166 proteomic Target: transition status N: 49 transition, 84 nontransition	48/49 (98.0)	1/49 (2.0)	68/84 (81.0)	16/84 (19.0)	98.0	81.0	89.5	0.95 (0.91-0.99)	75.0	98.6	5.1	<0.1
**Model 1b: EU-GEI clinical data**^a^ Data set: EU-GEI initial experiment, all sites Features: 69 clinical Target: transition status N: 49 transition, 84 nontransition	23/49 (46.9)	26/49 (53.1)	45/84 (53.6)	39/84 (46.4)	46.9	53.6	50.3	0.48 (0.38-0.58)	37.1	63.4	1.0	1.0
**Model 1c: EU-GEI proteomic data**^a^ Data set: EU-GEI initial experiment, all sites Features: 166 proteomic Target: transition status N: 49 transition, 84 nontransition	49/49 (100)	0/49 (0)	71/84 (84.5)	13/84 (15.5)	100	84.5	92.3	0.96 (0.92-1.00)	79.0	100	6.5	<0.1
**Model 2a: EU-GEI proteomic data (non-London)**^a^ Data set: EU-GEI initial experiment, all sites except London Features: 166 proteomic Target: transition status N: 30 transition, 50 nontransition	28/30 (93.3)	2/30 (6.7)	40/50 (80.0)	10/50 (20.0)	93.3	80.0	86.7	0.94 (0.88-1.00)	73.7	95.2	4.7	0.1
**Model 2b: top 10, training data**^a^ Data set: EU-GEI initial experiment, all sites except London Features: 10 proteomic Target: transition status N: 30 transition, 50 nontransition	30/30 (100)	0/30	41/50 (82.0)	9/50 (18.0)	100	82.0	91.0	0.99 (0.96-1.00)	76.9	100	5.6	<0.1
**Model 2b: top 10, test data**^a^ Data set: EU-GEI initial experiment, London site Features: 10 proteomic Target: transition status N: 19 transition, 34 nontransition	18/19 (94.7)	1/19 (5.3)	30/34 (88.2)	4/34 (11.8)	94.7	88.2	91.5	0.92 (0.83-1.00)	81.8	96.8	8.1	0.1
**Model 3: EU-GEI clinical and proteomic data**^a^ Data set: EU-GEI replication experiment, all sites Features: 69 clinical and 119 proteomic Target: transition status N: 49 transition, 86 nontransition	48/49 (98.0)	1/49 (2.0)	77/86 (89.5)	9/86 (10.5)	98.0	89.5	93.7	0.98 (0.95-1.00)	84.2	98.7	9.4	<0.1
**Model 4: ALSPAC proteomic data**^a^ Data set: ALSPAC Features: 265 proteomic Target: PEs at age 18 y N: 55 PEs, 66 no PE	40/55 (72.7)	15/55 (27.3)	47/66 (71.2)	19/66 (28.8)	72.7	71.2	72.0	0.74 (0.65-0.83)	67.8	75.8	2.5	0.4
**Model S1: ELISA** Data set: EU-GEI initial experiment, all sites Features: 9 ELISA Target: transition status N: 44 transition, 82 nontransition	33/44 (75.0)	11/44 (25.0)	51/82 (62.2)	31/82 (37.8)	75.0	62.2	68.6	0.76 (0.67-0.85)	51.6	82.3	2.0	0.4
**Model S2: functional outcome** Data set: EU-GEI initial experiment, all sites Features: 69 clinical and 166 proteomic Target: functional outcome N: 47 poor functioning (GAF ≤60); 32 good functioning (GAF >60)	27/47 (57.4)	20/47 (42.6)	22/32 (68.8)	10/32 (31.3)	57.4	68.8	63.1	0.74 (0.63-0.85)	73.0	52.4	1.8	0.6

Abbreviations: ALSPAC, Avon Longitudinal Study of Parents and Children; AUC, area under the receiver operating characteristic curve; ELISA, enzyme-linked immunosorbent assay; EU-GEI, European Network of National Schizophrenia Networks Studying Gene-Environment Interactions; NPV, negative predictive value; PE, psychotic experience; PPV, positive predictive value.

^a^ Models 1a-c, 2, and 3 are adjusted for age, sex, body mass index, and years of education, and model 4 is additionally adjusted for race/ethnicity and tobacco use.

**Table 3.** Ten Percent Highest-Weighted Features for Model 1a, Model 3, and Model 4^a^

Model/Feature	Mean weight
**Model 1a: EU-GEI clinical and proteomic data, initial experiment, all sites**	
P01023 Alpha-2-macroglobulin	−0.330
P01871 Immunoglobulin heavy constant mu	−0.256
P04003 C4b-binding protein alpha chain	−0.161
P07357 Complement component 8 alpha chain	0.158
P55058 Phospholipid transfer protein	−0.146
O75636 Ficolin 3	−0.145
P02774 Vitamin D–binding protein	0.135
P07225 Vitamin K–dependent protein S	−0.132
P43320 Beta-crystallin B2	0.132
P02766 Transthyretin	−0.130
P23142 Fibulin 1	0.125
P10909 Clusterin	0.121
P05155 Plasma protease C1 inhibitor	−0.114
Sex	−0.111
P00747 Plasminogen	0.111
P13671 Complement component 6	0.111
P02747 Complement C1q subcomponent subunit C	0.109
P02753 Retinol-binding protein 4	0.109
Q76LX8 A disintegrin and metalloproteinase with thrombospondin motifs 13	−0.108
P08697 Alpha-2-antiplasmin	−0.106
P19827 Inter-alpha-trypsin inhibitor heavy chain H1	0.105
MADRS: concentration difficulties	−0.104
P02489 Alpha-crystallin A chain	0.101
**Model 3: EU-GEI clinical and proteomic data, replication experiment, all sites**	
P01023 Alpha-2-macroglobulin	−0.286
P22792 Carboxypeptidase N subunit 2	0.210
P01871 Immunoglobulin heavy constant mu	−0.193
P09871 Complement C1s subcomponent	−0.181
P01011 Alpha-1-antichymotrypsin	0.168
P00747 Plasminogen	0.163
P08571 Monocyte differentiation antigen CD14	0.161
P10909 Clusterin	0.158
Q16610 Extracellular matrix protein 1	0.157
G3XAM2 Complement factor I	0.140
P04003 C4b-binding protein alpha chain	−0.140
P13671 Complement component 6	0.132
P25311 Zinc alpha-2-glycoprotein	−0.131
P07359 Platelet glycoprotein Ib alpha chain	0.126
P01031 Complement C5	0.125
O75882 Attractin	0.123
P0DOY3 Immunoglobulin lambda constant 3	−0.120
P15169 Carboxypeptidase N catalytic chain (CPN)	0.115
**Model 4: ALSPAC proteomic data**	
P04003 C4b-binding protein alpha chain	−0.227
P27169 Serum paraoxonase/arylesterase 1	−0.180
Q03591 Complement factor H–related protein 1	−0.152
P07225 Vitamin K–dependent protein S	−0.145
P61626 Lysozyme C	−0.142
P55103 Inhibin beta C chain	0.139
Q08380 Galectin 3–binding protein	0.132
P24593 Insulinlike growth factor–binding protein 5	0.122
P00746 Complement factor D	0.120
P01019 Angiotensinogen	−0.118
P01871 Immunoglobulin heavy constant mu	−0.116
O75636 Ficolin 3	0.115
Q9H4A9 Dipeptidase 2	−0.115
P01023 Alpha-2-macroglobulin	−0.113
P04275 von Willebrand factor	−0.111
Q9NQ79 Cartilage acidic protein 1	0.107
P24592 Insulinlike growth factor–binding protein 6	0.106
P09871 Complement C1s subcomponent	−0.105
P10909 Clusterin	−0.105
O95497 Pantetheinase	0.105
P02654 Apolipoprotein C-I	−0.099
P02679 Fibrinogen gamma chain	−0.099
P07358 Complement component C8 beta chain	0.097
Q5T7F0 Neuropilin	−0.097
P04040 Catalase	0.094
P43251 Biotinidase	0.094

Abbreviations: ALSPAC, Avon Longitudinal Study of Parents and Children; BPRS, Brief Psychiatric Rating Scale; EU-GEI, European Network of National Schizophrenia Networks Studying Gene-Environment Interactions; MADRS, Montgomery-Åsberg Depression Rating Scale; SANS, Scale for the Assessment of Negative Symptoms.

^a^ Ranked according to the mean feature weight for models selected in cross-validation inner loop. Proteins are presented with their UniProt accession number and corresponding protein name.

#### Model 1b and 1c: Clinical and Proteomic Data

The clinical model (model 1b) demonstrated poor predictive performance (AUC, 0.48; *P* = .63). These results are summarized in Table 2 and eFigure 3 in Supplement 1. For example, sensitivity was 46.9%, specificity was 53.6%, PPV was 37.1%, and NPV was 63.4%.

The proteomic model (model 1c) demonstrated excellent predictive performance (AUC, 0.96; *P* < .001). These results are summarized in Table 2 and eFigure 4 in Supplement 1. For example, sensitivity was 100%, specificity was 84.5%, PPV was 79.0%, and NPV was 100%.

#### Model 2a and b: Parsimonious Model

The AUC for the model based on proteomic data from all sites except London (model 2a) was 0.94 (*P* < .001) (Table 2 and eFigure 5 in Supplement 1). The 10 highest-weighted features were alpha-2-macroglobulin (A2M), immunoglobulin heavy constant mu (IGHM), C4b-binding protein alpha chain (C4BPA), vitamin K–dependent protein S, fibulin 1, transthyretin, *N*-acetylmuramoyl-l-alanine amidase, vitamin D–binding protein, clusterin, and complement component 6 (C6).

A reduced model based solely on these 10 most predictive proteins was developed using data from all sites except London (model 2b), with an AUC of 0.99 (*P* < .001), sensitivity of 100%, specificity of 82.0%, PPV of 76.9%, and NPV of 100%) (Table 2 and eFigure 6 in Supplement 1). This model predicted transition status in the held-out London data, with an AUC of 0.92, sensitivity of 94.7%, specificity of 88.2%, PPV of 81.8%, and NPV of 96.8% (Table 2).

#### ELISA Validation

After FDR correction, 2 proteins assessed by ELISA showed statistically significant mean differences between CHR-T and CHR-NT. These were A2M and complement component 1r (C1r) (eTables 9 and 10 in Supplement 1). The A2M mean in CHR-T was 1173.1 μg/mL vs 11 501.7 μg/mL in CHR-T (FDR-corrected *P* = .02), and the C1r mean in CHR-T was 65 008.9 μg/mL vs 52 803.9 μg/mL in CHR-NT (FDR-corrected *P* = .04).

#### Model 3: Replication

Replication subsample characteristics are listed in eTables 11 and 12 in Supplement 1. Of 485 proteins identified, 119 were quantified in more than 80% of plasma samples. There was nominally statistically significant (*P* < .05) differential expression of 82 proteins, of which 78 remained statistically significant after FDR correction (eTable 13 in Supplement 1).

Model 3 demonstrated excellent performance for prediction of transition in the replication data set (AUC, 0.98 [*P* < .001]; sensitivity, 98.0%; specificity, 89.5%; PPV, 84.2%; and NPV, 98.7%) (Table 2 and eFigure 7 in Supplement 1). The highest-weighted 10% of features are listed in Table 3. For example, the 5 highest-ranked predictive features were A2M (mean weight, −0.286), carboxypeptidase N subunit 2 (mean weight, 0.210), IGHM (mean weight, −0.193), complement C1s subcomponent (mean weight, −0.181), and alpha-1-antichymotrypsin (mean weight, 0.168). Proteins among the highest-weighted 10% of features in both model 1a and model 3 (and weighted in similar directions) included A2M, IGHM, C4BPA, plasminogen, and C6.

### Study 2: General Population Sample

The initial subsample was composed of plasma samples from 132 participants (65 case and 67 control samples). Eleven plasma samples were excluded because of poor protein identification profiles, resulting in 55 case and 66 control samples from 121 participants (61 [50.4%] male). Case samples were more likely to be from female participants. There was no evidence for differences in BMI, race/ethnicity, or maternal social class (eTable 14 in Supplement 1).

#### Differential Expression

Of 506 proteins identified, 265 were quantified in more than 80% of samples. There was nominally statistically significant (*P* < .05) differential expression of 40 proteins at age 12 years (eTable 15 in Supplement 1), of which the following 5 remained statistically significant after FDR correction: C4BPA (ratio of means in PE vs no PE, 0.77), serum paraoxonase/arylesterase 1 (ratio of means, 0.80), IGHM (ratio of means, 0.78), inhibin beta chain (ratio of means, 1.31), and clusterin (ratio of means, 0.92).

#### Model 4: Predicting PEs Using Proteomic Data

An SVM model using 265 proteomic features from plasma samples obtained at age 12 years predicted PEs at age 18 years, with an AUC of 0.74 (*P* < .001), sensitivity of 72.7%, specificity of 71.2%, PPV of 67.8%, and NPV of 75.8% (Table 2 and eFigure 8 in Supplement 1). For example, the 5 highest-ranked predictive features were C4BPA (mean weight, −0.227), serum paraoxonase/arylesterase 1 (mean weight, −0.180), complement factor H–related protein 1 (mean weight, −0.152), vitamin K–dependent protein S (mean weight, −0.145), and lysozyme C (mean weight, −0.142) (Table 3).

### Supplementary Analyses

Model S1 used ELISA data to predict transition status in EU-GEI, with an AUC of 0.76 (*P* < .001). These results are summarized in Table 2 and eFigure 9 in Supplement 1.

Model S2 used clinical and proteomic data to predict poor (GAF disability subscale score ≤60) vs good (>60) functional outcome at 2 years in EU-GEI, with an AUC of 0.74 (*P* = .003) (Table 2 and eFigure 10 in Supplement 1). The 10% highest-weighted features are listed in eTable 16 in Supplement 1.

There was evidence of differences for the clinical data between the London and the Netherlands sites compared with others (eTable 17, eFigure 11, and eFigure 23 in Supplement 1), likely because of group differences in age, years in education, and BPRS score (eMethods and eFigures 13-22 in Supplement 1). There was no strong evidence of systematic site associations for the proteomic data (eTable 18, eFigure 12, and eFigure 24 in Supplement 1).

Performance metrics of multivariate-corrected SVM models are listed in eTable 19 in Supplement 1. There were generally slight reductions in AUCs of the corrected models compared with their uncorrected counterparts (median change in AUC, 0.04; range, 0.01-0.10), although in all cases the 95% CIs overlapped.

## Discussion

We described evidence of differential baseline plasma protein expression in individuals at CHR who developed psychosis compared with those who did not. Machine learning algorithms that incorporated clinical and proteomic data were used to predict transition outcome (AUC, 0.95). Proteomic features were of greater predictive value than clinical features. A parsimonious model based on 10 highly predictive proteins showed excellent performance in training (AUC, 0.99) and test (AUC, 0.92) data. Furthermore, a predictive model was developed using proteomic data at age 12 years for PEs at age 18 years in a general population sample (AUC, 0.74).

Although only 16% to 35% of individuals at CHR transition to FEP,^3^ the CHR state remains a strong risk factor.^31^ Clinical data have previously shown value for prediction of transition,^32,33,34,35,36,37^ and the poor performance of the clinical features in our study does not imply that clinical data in general are of little prognostic use. Previous studies have attempted to augment accuracy using neuroimaging^38,39,40,41^ and neurocognitive^42^ data, but blood-based tests have the advantage of greater accessibility. Perkins et al^43^ derived a panel of 15 proteins using immunoassays that distinguished between CHR-T and CHR-NT, with an AUC of 0.88. Chan et al^44^ used 22 blood-based biomarkers to predict schizophrenia onset, with an AUC of 0.82 that increased to 0.90 with incorporation of the CAARMS positive symptoms subscale. Our parsimonious model used data for 10 proteins, and, with further validation, may contribute to individualized prognosis and treatment stratification strategies.^45^

eTable 20 in Supplement 1 summarizes our findings of differential expression in CHR-T vs CHR-NT and the predicted functional implications (modeled in eFigure 25 in Supplement 1). We found particularly strong evidence for dysregulation of the complement and coagulation cascade, previously implicated in schizophrenia.^46,47,48,49,50^ Similar processes have been previously implicated in proteomic studies of the development of PEs in the general population.^12,20^ Changes in the present CHR study that were consistent with results from these previous PE studies include increases in plasminogen, C1r, clusterin, and complement factor H and decreases in A2M and IGHM. The primary causes of these changes remain unknown but are consistent with evidence of enhanced inflammatory tone preceding psychosis and other mental disorders^43,44,51,52,53,54,55^ and schizophrenia risk associated with genetic variation of complement C4.^56^

Several complement proteins emerged as important predictors of transition, including C4BPA, C1r of the antibody-antigen complex mediated pathway, key regulatory protease complement factor I, and terminal pathway components C6 and C8A. These arise from common pathways or functionally interact with coagulation proteins plasminogen and vitamin K–dependent protein S, supporting hypotheses of coagulation activation in psychosis.^57^ In both the initial and replication experiments, the most highly weighted predictor of transition was A2M (decreased in CHR-T vs CHR-NT), a protease inhibitor with diverse functions, including inhibition of proinflammatory cytokines such as interleukin 1β^58^ (consistently elevated in FEP^59^). A2M is a key coagulation inhibitor^60^ and thus links functionally to our observations of elevated plasminogen in CHR-T. This finding is intriguing given the evidence that blood-derived plasminogen is associated with brain inflammation^61^ and complement activation.^62^ In models of multiple sclerosis, blood-brain barrier disruption facilitates transfer of fibrinogen into the brain, where it is deposited as fibrin, causing local inflammation.^63^ Given evidence for blood-brain barrier disruption in psychosis,^64^ fibrin may be associated with etiopathogenic mechanisms providing novel therapeutic avenues,^65^ but this hypothesis requires further investigation.

We validated differential expression of A2M and C1r using ELISA. The ELISA-based model (model S1) demonstrated fair, although reduced, predictive accuracy. This finding may reflect reduced sensitivity of ELISA and the inability to accurately quantify specific protein isoforms. Several proteins in the highest-weighted 10% of features for transition in study 1 were similarly highly weighted for PEs in study 2, including C4BPA, vitamin K–dependent protein S, A2M, and IGHM (eTable 21 in Supplement 1 summarizes the directionality of association of the 10% highest predictors in model 1a, model 3, and model 4). This observation may suggest a degree of similarity in proteomic changes between young people in the general population who develop PEs and help-seeking individuals at CHR who develop psychosis, but this hypothesis requires confirmation.

Outside of psychosis outcomes, several proteomic features contributed to prediction of functional outcome (model S2). A2M, IGHM, phospholipid transfer protein, and clusterin were among the 10% highest-weighted predictors. The results of the present study are also in keeping with studies in bipolar disorder and depression reporting decreased A2M, IgM, and C4BPA.^66^ At least some of these proteomic changes may be common to multiple clinical phenotypes, including neurodegenerative disorders, such as Alzheimer disease.^67^ Rather than considering such changes as biomarkers of individual disorders, phenotypic manifestations may be clinical markers of a variety of overlapping neuroimmune abnormalities that have their origin in combined genetic^56,68^ and environmental^69,70,71,72^ factors.

### Limitations

This study has some limitations. First, these models require validation in independent cohorts to assess generalizability and real-world applicability. Second, differences in protein identifications precluded application of models between studies. However, there are valid reasons not to do so, including differences in outcome (psychotic disorder vs PEs) and age (postpubertal vs peripubertal). Third, data on duration of follow-up and reasons for dropout were not systematically collected in EU-GEI, and we were unable to fully assess the potential implications of these factors. Fourth, the replication experiment was partial because only 2 CHR-T cases were different from the initial experiment. Although our findings were generally replicated, no statement can be made regarding generalizability of model sensitivity. Fifth, participants were nonfasting, and there were no restrictions on time of sample collection. Sixth, other factors, such as childhood adversity, may have contributed to the proteomic changes that we observed,^10,71^ but these factors require further study.

## Conclusions

We developed models incorporating proteomic data predicting transition to psychotic disorder in the CHR state. In a general population sample, several of the same proteins contributed to prediction of PEs. Further studies are required to validate these findings, evaluate their causes, and elucidate tractable targets for prediction and prevention of psychosis.

## Notes

Supplement 1.
eMethods.

eReferences.
**eTable 1.** Summary of Support Vector Machine Models **eTable 2.** List of 69 Baseline EU-GEI Clinical Variables Included in Model 1a, Model 1b, and Model 3 **eTable 3.** Comparison of Baseline Characteristics for CHR Participants Who Attended at Least One Follow-up Interview vs CHR Participants Who Did Not in EU-GEI **eTable 4.** Comparison of Characteristics for Participants Included in Initial Experiment (N = 133) From Total EU-GEI Clinical High-Risk Cohort (N = 344) **eTable 5.** Results of ANCOVA (Adjusted for Age, Sex, BMI, and Years in Education) and Fold Changes (CHR-T vs CHR-NT) for Proteins Identified in EU-GEI Baseline Plasma Samples in the Initial Experiment **eTable 6.** Coefficients of Variation for Proteins Across Quality Control Standards in EU-GEI Initial Experiment **eTable 7.** Summary of Protein-Protein Interactions Identified From the BIOGRID Database for Significantly Differentially Expressed Proteins Between CHR-T and CHR-NT in EU-GEI Initial Experiment **eTable 8.** Functional Enrichment Analysis of Differentially Expressed Proteins (Following False Discovery Rate Correction) Between CHR-T and CHR-NT in EU-GEI Initial Experiment: 6 KEGG Pathways Significantly Enriched **eTable 9.** Results of Enzyme-Linked Immunosorbent Assays in CHR-T and CHR-NT Participants in EU-GEI Initial Experiment **eTable 10.** Correlations Between 5 Proteins Assessed by ELISA and by Mass Spectrometry in EU-GEI Initial Experiment **eTable 11.** Comparison of Characteristics for Participants Included in Replication Experiment (N = 135) From Total EU-GEI Clinical High-Risk Cohort (N = 344) **eTable 12.** Sample Characteristics for CHR-T and CHR-NT Groups in the Replication Experiment **eTable 13.** Results of ANCOVA (Adjusted for Age, Sex, BMI, Years in Education, Tobacco Use, and Ethnicity) and Fold Changes (CHR-T vs CHR-NT) for Proteins Identified in EU-GEI Baseline Plasma Samples in the Replication Experiment **eTable 14.** Sample Characteristics for ALSPAC Subsample Cases and Controls **eTable 15.** Results of ANCOVA (Adjusted for Sex, BMI, and Maternal Social Class) and Fold Changes (Definite PEs at 18 vs no PEs at 18) for Proteins Identified in ALSPAC Age 12 Plasma Samples **eTable 16.** Ten Percent Highest-Weighted Features for Model S2 (Support Vector Machine Model Predicting Functional Outcome at 24 Months in EU-GEI) **eTable 17.** Table Comparing Performance Metrics for Multiclass Site Prediction Models Based on 69 Clinical Features From Model 1b **eTable 18.** Table Comparing Performance Metrics for Multiclass Site Prediction Models Based on 166 Proteomic Features From Model 1c **eTable 19.** Comparison of Performance Metrics for Uncorrected vs Corrected Support Vector Machine Models **eTable 20.** Proteins Differentially Expressed in CHR-T vs CHR-NT on ANCOVA (*P* < .05) in EU-GEI Baseline Plasma Samples in the Initial and Replication Experiment and Predicted Systemic Impact on Coagulation and Complement Activation and Regulation **eTable 21.** Table Summarizing Directionality of Effect of Ten Percent Highest-Weighted Features in Model 1a (EU-GEI Initial Experiment), Model 3 (EU-GEI Replication Experiment), and Model 4 (ALSPAC Proteomic Data) **eFigure 1.** Derivation and Testing of Model 2b: Parsimonious (10-Predictor) Proteomic Model **eFigure 2.** STRING Functional Protein Association Network for Proteins Differentially Expressed (Following False Discovery Rate Correction) Between CHR-T and CHR-NT in EU-GEI Initial Experiment **eFigure 3.** Mean Algorithm Scores and Class Predictions (A) and Receiver Operating Characteristic Curve (B) for Model 1b: Clinical Data **eFigure 4.** Mean Algorithm Scores and Class Predictions (A) and Receiver Operating Characteristic Curve (B) for Model 1c: Proteomic Data **eFigure 5.** Mean Algorithm Scores and Class Predictions (A) and Receiver Operating Characteristic Curve (B) for Model 2a: Proteomic (Non-London) **eFigure 6.** Mean Algorithm Scores and Class Predictions (A) and Receiver Operating Characteristic Curve (B) for Model 2b: Parsimonious (10-Predictor) Proteomic Model, Training Data (Non-London) **eFigure 7.** Mean Algorithm Scores and Class Predictions (A) and Receiver Operating Characteristic Curve (B) for Model 3: Replication **eFigure 8.** Mean Algorithm Scores and Class Predictions (A) and Receiver Operating Characteristic Curve (B) for Model 4: ALSPAC Proteomic Data **eFigure 9.** Mean Algorithm Scores and Class Predictions (A) and Receiver Operating Characteristic Curve (B) for Model S1: ELISA **eFigure 10.** Mean Algorithm Scores and Class Predictions (A) and Receiver Operating Characteristic Curve (B) for Model S2: Functional Outcome **eFigure 11.** Model 1b (Clinical Data) Decision Scores Stratified by EU-GEI Site **eFigure 12.** Model 1c (Proteomic Data) Decision Scores Stratified by EU-GEI Site **eFigure 13.** Age Stratified by EU-GEI Site **eFigure 14.** Years in Education Stratified by EU-GEI Site **eFigure 15.** Body Mass Index Stratified by EU-GEI Site **eFigure 16.** Sex Stratified by EU-GEI Site **eFigure 17.** General Assessment of Functioning Symptoms Subscale Stratified by EU-GEI Site **eFigure 18.** General Assessment of Functioning Disability Subscale Stratified by EU-GEI Site **eFigure 19.** Scale for Assessment of Negative Symptoms (Composite Score) Stratified by EU-GEI Site **eFigure 20.** Scale for Assessment of Negative Symptoms (Global Score) Stratified by EU-GEI Site **eFigure 21.** Brief Psychiatric Rating Scale Score Stratified by EU-GEI Site **eFigure 22.** Montgomery-Asberg Depression Rating Scale Score Stratified by EU-GEI Site **eFigure 23.** Multiclass Receiver Operating Curves for Site Prediction Based on 69 Clinical Features From Model 1b **eFigure 24.** Multiclass Receiver Operating Curves for Site Prediction Based on 166 Proteomic Features From Model 1c **eFigure 25.** Illustration of the Complement and Coagulation Pathways Depicting the Impact Model of Differentially Expressed Complement and Coagulation Proteins in CHR-T vs CHR-NT

Supplement 2. **eAppendix.** MS2-Determined Peptide Sequences Used to Match to Parent Proteins in the Human FASTA Database (Excel File)
