# Supplementary material for: Development of Proteomic Prediction Models for Transition to Psychotic Disorder in the Clinical High-Risk State and Psychotic Experiences in Adolescence
Source: JAMA Psychiatry. 2020 Aug 26;78(1):77–90. doi: 10.1001/jamapsychiatry.2020.2459 (PMC7450406; doi:10.1001/jamapsychiatry.2020.2459)
Supplement: Supplement 1. — eMethods. eReferences. eTable 1. Summary of Support Vector Machine Models eTable 2. List of 69 Baseline EU-GEI Clinical Variables Included in Model 1a, Model 1b, and Model 3 eTable 3. Comparison of Baseline Characteristics for CHR Participants Who Attended at Least One Follow-up Interview vs CHR Participants Who Did Not in EU-GEI eTable 4. Comparison of Characteristics for Participants Included in Initial Experiment (N = 133) From Total EU-GEI Clinical High-Risk Cohort (N = 344) eTable 5. Results of ANCOVA (Adjusted for Age, Sex, BMI, and Years in Education) and Fold Changes (CHR-T vs CHR-NT) for Proteins Identified in EU-GEI Baseline Plasma Samples in the Initial Experiment eTable 6. Coefficients of Variation for Proteins Across Quality Control Standards in EU-GEI Initial Experiment eTable 7. Summary of Protein-Protein Interactions Identified From the BIOGRID Database for Significantly Differentially Expressed Proteins Between CHR-T and CHR-NT in EU-GEI Initial Experiment eTable 8. Functional Enrichment Analysis of Differentially Expressed Proteins (Following False Discovery Rate Correction) Between CHR-T and CHR-NT in EU-GEI Initial Experiment: 6 KEGG Pathways Significantly Enriched eTable 9. Results of Enzyme-Linked Immunosorbent Assays in CHR-T and CHR-NT Participants in EU-GEI Initial Experiment eTable 10. Correlations Between 5 Proteins Assessed by ELISA and by Mass Spectrometry in EU-GEI Initial Experiment eTable 11. Comparison of Characteristics for Participants Included in Replication Experiment (N = 135) From Total EU-GEI Clinical High-Risk Cohort (N = 344) eTable 12. Sample Characteristics for CHR-T and CHR-NT Groups in the Replication Experiment eTable 13. Results of ANCOVA (Adjusted for Age, Sex, BMI, Years in Education, Tobacco Use, and Ethnicity) and Fold Changes (CHR-T vs CHR-NT) for Proteins Identified in EU-GEI Baseline Plasma Samples in the Replication Experiment eTable 14. Sample Characteristics for ALSPAC Subsample Cases and Controls eTable 15. [file jamapsychiatry-e202459-s001.pdf]

Supplementary Online Content

Mongan D, Föcking M, Healy C, et al; European Network of National Schizophrenia Networks Studying Gene-Environment Interactions (EU-GEI) High Risk Study Group. Development of proteomic prediction models for transition to psychotic disorder in the clinical high-risk state and psychotic experiences in adolescence. *JAMA Psychiatry*. Published online August 26, 2020. doi:10.1001/jamapsychiatry.2020.2459

eMethods.

eReferences.

eTable 1. Summary of Support Vector Machine Models

eTable 2. List of 69 Baseline EU-GEI Clinical Variables Included in Model 1a, Model 1b, and Model 3

eTable 3. Comparison of Baseline Characteristics for CHR Participants Who Attended at Least One Follow-up Interview vs CHR Participants Who Did Not in EU-GEI

eTable 4. Comparison of Characteristics for Participants Included in Initial Experiment (N=133) From Total EU-GEI Clinical High-Risk Cohort (N=344)

eTable 5. Results of ANCOVA (Adjusted for Age, Sex, BMI, and Years in Education) and Fold Changes (CHR-T vs CHR-NT) for Proteins Identified in EU-GEI Baseline Plasma Samples in the Initial Experiment

eTable 6. Coefficients of Variation for Proteins Across Quality Control Standards in EU-GEI Initial Experiment

eTable 7. Summary of Protein-Protein Interactions Identified From the BIOGRID Database for Significantly Differentially Expressed Proteins Between CHR-T and CHR-NT in EU-GEI Initial Experiment

eTable 8. Functional Enrichment Analysis of Differentially Expressed Proteins (Following False Discovery Rate Correction) Between CHR-T and CHR-NT in EU-GEI Initial Experiment: 6 KEGG Pathways Significantly Enriched

eTable 9. Results of Enzyme-Linked Immunosorbent Assays in CHR-T and CHR-NT Participants in EU-GEI Initial Experiment

eTable 10. Correlations Between 5 Proteins Assessed by ELISA and by Mass Spectrometry in EU-GEI Initial Experiment

eTable 11. Comparison of Characteristics for Participants Included in Replication Experiment (N=135) From Total EU-GEI Clinical High-Risk Cohort (N=344)

eTable 12. Sample Characteristics for CHR-T and CHR-NT Groups in the Replication Experiment

eTable 13. Results of ANCOVA (Adjusted for Age, Sex, BMI, Years in Education, Tobacco Use, and Ethnicity) and Fold Changes (CHR-T vs CHR-NT) for Proteins Identified in EU-GEI Baseline Plasma Samples in the Replication Experiment

eTable 14. Sample Characteristics for ALSPAC Subsample Cases and Controls

© 2020 Mongan D et al. *JAMA Psychiatry.*

eTable 15. Results of ANCOVA (Adjusted for Sex, BMI, and Maternal Social Class) and Fold Changes (Definite PEs at 18 vs no PEs at 18) for Proteins Identified in ALSPAC Age 12 Plasma Samples

© 2020 American Medical Association. All rights reserved.

eTable 16. Ten Percent Highest-Weighted Features for Model S2 (Support Vector Machine Model Predicting Functional Outcome at 24 Months in EU-GEI)

eTable 17. Table Comparing Performance Metrics for Multiclass Site Prediction Models Based on 69 Clinical Features From Model 1b

eTable 18. Table Comparing Performance Metrics for Multiclass Site Prediction Models Based on 166 Proteomic Features From Model 1c

eTable 19. Comparison of Performance Metrics for Uncorrected vs Corrected Support Vector Machine Models

eTable 20. Proteins Differentially Expressed in CHR-T vs CHR-NT on ANCOVA (P<.05) in EU-GEI Baseline Plasma Samples in the Initial and Replication Experiment and Predicted Systemic Impact on Coagulation and Complement Activation and Regulation

eTable 21. Table Summarizing Directionality of Effect of Ten Percent Highest-Weighted Features in Model 1a (EU-GEI Initial Experiment), Model 3 (EU-GEI Replication Experiment), and Model 4 (ALSPAC Proteomic Data)

eFigure 1. Derivation and Testing of Model 2b: Parsimonious (10-Predictor) Proteomic Model

eFigure 2. STRING Functional Protein Association Network for Proteins Differentially Expressed (Following False Discovery Rate Correction) Between CHR-T and CHR-NT in EU-GEI Initial Experiment

eFigure 3. Mean Algorithm Scores and Class Predictions (A) and Receiver Operating Characteristic Curve (B) for Model 1b: Clinical Data

eFigure 4. Mean Algorithm Scores and Class Predictions (A) and Receiver Operating Characteristic Curve (B) for Model 1c: Proteomic Data

eFigure 5. Mean Algorithm Scores and Class Predictions (A) and Receiver Operating Characteristic Curve (B) for Model 2a: Proteomic (Non-London)

eFigure 6. Mean Algorithm Scores and Class Predictions (A) and Receiver Operating Characteristic Curve (B) for Model 2b: Parsimonious (10-Predictor) Proteomic Model, Training Data (Non-London)

eFigure 7. Mean Algorithm Scores and Class Predictions (A) and Receiver Operating Characteristic Curve (B) for Model 3: Replication

eFigure 8. Mean Algorithm Scores and Class Predictions (A) and Receiver Operating Characteristic Curve (B) for Model 4: ALSPAC Proteomic Data

eFigure 9. Mean Algorithm Scores and Class Predictions (A) and Receiver Operating Characteristic Curve (B) for Model S1: ELISA

eFigure 10. Mean Algorithm Scores and Class Predictions (A) and Receiver Operating Characteristic Curve (B) for Model S2: Functional Outcome

eFigure 11. Model 1b (Clinical Data) Decision Scores Stratified by EU-GEI Site

eFigure 12. Model 1c (Proteomic Data) Decision Scores Stratified by EU-GEI Site

eFigure 13. Age Stratified by EU-GEI Site

eFigure 14. Years in Education Stratified by EU-GEI Site

eFigure 15. Body Mass Index Stratified by EU-GEI Site

eFigure 16. Sex Stratified by EU-GEI Site

eFigure 17. General Assessment of Functioning Symptoms Subscale Stratified by EU-GEI Site

eFigure 18. General Assessment of Functioning Disability Subscale Stratified by EU-GEI Site

eFigure 19. Scale for Assessment of Negative Symptoms (Composite Score) Stratified by EU-GEI Site

eFigure 20. Scale for Assessment of Negative Symptoms (Global Score) Stratified by EU-GEI Site

eFigure 21. Brief Psychiatric Rating Scale Score Stratified by EU-GEI Site

eFigure 22. Montgomery-Asberg Depression Rating Scale Score Stratified by EU-GEI Site

eFigure 23. Multiclass Receiver Operating Curves for Site Prediction Based on 69 Clinical Features From Model 1b

eFigure 24. Multiclass Receiver Operating Curves for Site Prediction Based on 166 Proteomic Features From Model 1c

eFigure 25. Illustration of the Complement and Coagulation Pathways Depicting the Impact Model of Differentially Expressed Complement and Coagulation Proteins in CHR-T vs CHR-NT

This supplementary material has been provided by the authors to give readers additional information about their work.

© 2020 American Medical Association. All rights reserved.

eMethods

1. Sample preparation: further details

1.1 Sample collection

Blood samples were collected according to a standardised protocol across the participating EU-GEI sites. BD Vacutainer P100 tubes (BD Biosciences, USA) were used for sample collection. Samples were stored on ice for a maximum of 90 minutes, then centrifuged at 2500g for 20 minutes. ALSPAC samples were collected into heparin Sarstedt S-Monovette tubes (Sarstedt, Germany) and stored on ice for a maximum of 90 minutes until processed. In both studies, participants were non-fasting and there were no restrictions regarding time of sample collection. Aliquots of plasma were stored in a -80°C freezer until processing for proteomic analysis as described below. All samples were subject to one freeze-thaw cycle prior to analysis. The standard quality of the plasma samples was ensured by assessing the overall MS protein profile to facilitate the identification of outlier protein expression profiles.

1.2 Protein depletion of plasma samples

To improve the dynamic range for proteomic analysis, 40µl of plasma from each case in all samples was immunodepleted of the 14 most abundant proteins (α-1-antitrypsin, A1-acid glycoprotein, Serum Albumin, α2-macroglobulin, Apolipoprotein A-I, Apolipoprotein A-II, Complement C3, Fibrinogen α/β/γ, Haptoglobin, IgA, IgG, IgM, Transthyretin, and Serotransferrin), using the Agilent Hu14 Affinity Removal System (MARS) coupled to a High Performance Liquid Chromatography (HPLC) system.[1] Protein depletion was undertaken according to the manufacturer's instructions and buffer exchange was performed with 50mM ammonium bicarbonate using spin columns with a 10kDA-molecular weight cut-off (Merck Millipore). Prior to sample preparation for mass spectrometry (MS), the protein concentration was determined using a Bradford Assay[2] according to the manufacturer's (BioRad) instructions.

1.3 Sample preparation for mass spectrometry

Protein digestion and peptide purification was performed as previously described.[3] For quality control (QC), an equal aliquot from each protein digest in the experiment was pooled into one sample for use as an internal QC. This QC standard was injected 3 times at the beginning of the MS study to condition the column, and after every 10 injections throughout the experiment to monitor the MS performance.

© 2020 Mongan D et al. *JAMA Psychiatry.*

2. Discovery proteomic analysis using data dependent acquisition (DDA): further methodological details

All samples were injected on a Thermo Scientific Q Exactive mass spectrometer connected to a Dionex Ultimate 3000 (RSLCnano) chromatography system. Tryptic peptides (5μl of digest) from each sample were loaded onto a fused silica emitter (75 μm ID), pulled using a laser puller (Sutter Instruments P2000), packed with UChrom C18 (1.8 μm) reverse phase media (nanoLCMS Solutions LCC) and separated by an increasing acetonitrile gradient over 90 minutes at a flow rate of 250 nL/min. The mass spectrometer was operated in data dependent TopN 8 mode, with the following settings: mass range 300-1600Th; resolution for MS1 scan 70000; AGC target 3e6; resolution for MS2 scan 17500; AGC target 2e4; charge exclusion unassigned, 1; dynamic exclusion 40 s. The peptide sequences used to match to parent proteins in the human FASTA database are provided in the eAppendix. The co-efficients of variation (CV) across the injected pooled QC standards for the 35 differentially expressed proteins in the initial experiment are provided in eTable 6. This table also provides the CV for proteins not among these 35 that were either among the 10% highest-weighted proteins in Model 1a, or among the 10 proteins included in Model 2b. The majority of these (80%) had CV <20%.

3. Confirmatory analyses using enzyme-linked immunosorbent assays (ELISA)

To validate our proteomic differential expression findings using another method, we assessed several human complement and coagulation proteins and apolipoproteins in the plasma samples of the same CHR-T and CHR-NT subjects who contributed to the initial EU-GEI proteomic experiment using enzyme-linked immunoassays (ELISA). Candidate proteins were chosen on the basis of differential expression and machine learning results as well as previous studies from our group ([4-7]). Specifically, we tested human α-2 macroglobulin (Abcam ab108888, 1:400), apolipoprotein E (ThermoFisher Scientific, EHAPOE, 1:2,000), Complement C1q (Abcam ab170246, 1:100,000), Complement C1r (Abcam ab170245, 1:40,000), Complement C4 binding protein (Abcam, ab222866, 1:40,000), Complement C8 (Abcam ab 137971, 1:10,000), complement factor H (Hykult Biotech HK342, 1:10,000), immunoglobulin M (Abcam, ab137982, 1:60,000), and plasminogen (Abcam ab108893, 1:20,000). Each kit had an optimised and defined sample dilution recommendation. The assays were performed according to the manufacturer's instructions to ensure sample concentrations measured would fall in the linear portion of the standard curve in which analyte

© 2020 Mongan D et al. *JAMA Psychiatry.*

concentration can be determined accurately. Each plate contained at least 8 wells in duplicate used for a standard curve of known protein concentrations. The assays have been used reliably in other studies.[8-20] Results are provided in eTable 9. With respect to concordance with the mass spectrometry data, ELISA and mass spectrometry results were available for 5 proteins (alpha-2-macroglobulin, plasminogen, complement component 1r, complement factor H and apolipoprotein E) of which 3 were significantly positively correlated (alpha-2-macroglobulin, plasminogen and apolipoprotein E); see eTable 10.

4. **Bioinformatics and statistical analysis: further details**

4.1 Predictive models

We used Neurominer v.1.0 (https://www.pronia.eu/neurominer/) for MatLab 2018a (MathWorks Inc.) to develop support vector machine (SVM) models. For all models, hyperparameters were optimised in repeated nested cross-validation and area under the receiver-operating characteristic curve (AUC) was the performance evaluation criterion. We used the LIBLINEAR program with L2 regularisation to attenuate over-fitting[21] whereby weights of non-predictive features are minimised, but not reduced to zero (thus more closely modelling the biological effects of functionally inter-related proteins). Hyperplane weighting was enabled (increasing the misclassification penalty in the minority class) which reduces risk of bias in unbalanced group sizes.[22] Random-label permutation analysis[23-25] with 1000 permutations was used to verify models against a null distribution and derive *p*-values for statistical significance. Prior to model training, missing clinical data were replaced using the mean (for continuous) or modal value (for categorical variables). Continuous clinical variables were converted to z-scores and winsorised within ±3z.

4.2 EU-GEI clinical features

A full list of the baseline clinical features included in Model 1a, Model 1b and Model 3 is provided in eTable 2.

4.3 Leave-site-out repeated nested cross-validation

The data were first split into folds in the ‘outer loop’ of cross-validation. To incorporate geographical generalisability, we split the data into groups by study site. Several of the smaller sites from the EU-GEI study were combined to ensure a large enough transition sample was present at each site. Thus, Amsterdam and The Hague were combined (Netherlands), Basel and Vienna were combined (Switzerland/ Austria),

© 2020 Mongan D et al. *JAMA Psychiatry.*

Copenhagen and Paris were combined (Denmark/ France), and Barcelona and Sao Paulo were combined (Spain/ Brazil). This resulted in 6 final groups (as shown below):

Group	CHR-NT, n (%)	CHR-T, n (%)
**London**	34 (64%)	19 (36%)
**Denmark/ France**	13 (54%)	11 (46%)
**Netherlands**	13 (87%)	2 (13%)
**Melbourne**	9 (64%)	5 (36%)
**Switzerland/ Austria**	6 (43%)	8 (57%)
**Spain/ Brazil**	9 (69%)	4 (31%)

These groups were folds in the outer cross-validation loop. For each cycle of cross-validation, data from each of the 6 groups were held out and the rest of the data moved into the 'inner loop' for training.

Within the inner loop, we used 5 non-overlapping folds with iterative training-test cycles. Thus, training was applied to four-fifths of the data in the inner loop and then tested against the final one-fifth, with the 5 different inner loop folds as the test fold. Models were trained and tested within the inner loop using a range of regularisation parameter values (in 11 steps from 0.015625 to 16). The optimal models thus derived were tested against the held-out site in the outer loop. This process was then repeated, with each site in the outer loop as the test site, to determine the overall optimal model and final predictive accuracy. For a detailed description of repeated nested cross-validation, see the supplementary material of Koutsouleris et al[26] and the Neurominer manual (available from https://www.pronia.eu/neurominer/).

4.4 Confidence intervals for area under the receiver-operating characteristic curve

95% confidence intervals for the area under the receiver-operating characteristic curve (AUC) for each model were calculated according to the method of Hanley & McNeil.[27]

4.5 ELISA results

Concentrations for the unknown samples were interpolated using 4 parameter logistic curve fit in GraphPad Prism 8 software. Means for each protein were compared using the t-test with unequal variances in Stata version 15.

4.6 Protein-protein interactions

To synthesise evidence for direct protein-protein interactions, searches for each of the 35 significantly differentially expressed proteins between CHR-T and CHR-NT in the initial experiment were performed using

© 2020 Mongan D et al. *JAMA Psychiatry.*

the BIOGRID curated database of protein-protein interactions (https://thebiogrid.org/). 34 of the 35 proteins were listed in the database (ADAMTS13 was not) and 11 unique interactions were identified (eTable 7). The same 35 proteins were entered into the STRING database (https://string-db.org/) to facilitate functional enrichment analysis (eTable 8) and generation of a functional protein association network (eFigure 2).

5. EU-GEI replication dataset

The replication dataset included 49 CHR-T participants (2 of whom were different from those in the original dataset used for Models 1a-d) and 86 CHR-NT participants (all of whom were different from those in the original dataset). Baseline characteristics of participants included in the replication dataset are compared to those not included in eTable 11. Included participants were more likely to be male, although this difference was no longer significant after false discovery rate (FDR) correction. Characteristics of the replication subsample are presented stratified by transition status in eTable 12. Regarding baseline characteristics, there was evidence of nominally significant differences between CHR-T and CHR-NT for ethnicity, tobacco use and mean total SANS composite, SANS global and BPRS scores, though not after FDR correction.

6. Supplementary analyses

6.1 Development of a model to predict transition outcome in EU-GEI based on ELISA results (Model S1)

Concentrations from the 9 ELISAs performed (as described in Section 3 above) were z-transformed and winsorised within ±3z. The results were used as features in an L2-regularised SVM algorithm using leave-site-out repeated nested cross-validation. Hyperplane weighting was enabled and area under the receiver-operating characteristic curve (AUC) was the performance evaluation criterion.

The performance metrics for this model are presented in Table 2. The mean algorithm scores, predicted classes and receiver-operating characteristic (ROC) curve are shown in eFigure 9.

6.2 Development of a model to predict functional outcome at 24 months in EU-GEI based on clinical and proteomic data (Model S2)

To investigate the performance of the available clinical and proteomic data for prediction of a transdiagnostic outcome, we developed a model based on the 69 clinical and 166 proteomic features used in Model 1a, but for prediction of functional outcome (Model S2). We used the General Assessment of Functioning (GAF)[28,29] disability subscale, recorded at the follow-up assessment closest to 2 years from baseline, as a measure of

© 2020 Mongan D et al. *JAMA Psychiatry.*

general functioning. For use as a classification target variable (and in line with previous approaches[30]) the score was dichotomised into ‘poor functioning’ (≤60 points, i.e. moderate or severe impairment) or ‘good functioning’ (>60 points, i.e. mild or no impairment).

An L2-regularised SVM algorithm was used to derive the model with AUC as the performance evaluation criterion. Hyperplane weighting was enabled. Compared to transition status, fewer participants had data available for functional outcome at 24 months (n=79). Therefore, this model used repeated nested cross-validation with 5 random inner and outer folds, irrespective of study site.

The performance metrics for this model are presented in Table 2 and the 10% highest-weighted features are shown in eTable 16. The mean algorithm scores, predicted classes and ROC curve are shown in eFigure 10.

6.3 Investigation of site associations in EU-GEI

Given the multisite nature of the EU-GEI study, we wished to investigate whether the clinical and proteomic data and predictions varied systematically by site.

First, we performed Kruskall-Wallis tests to determine whether the decision scores for Model 1b (clinical model) and Model 1c (proteomic model) differed according to the 6 site groupings used in the original models' leave-site-out cross validation schemes (i.e. London, Netherlands, Melbourne, Switzerland/Austria, Denmark/France and Spain/Brazil). The results are presented in eFigure 11 (for Model 1b, clinical data) and eFigure 12 (for Model 1c, proteomic data). There was evidence for differences in decision scores by site with respect to the clinical data (Model 1b) and post-hoc Dunn's tests[31] with Bonferroni correction suggested that the pertinent sites were London and Netherlands. There was little evidence for differences in decision scores by site with respect to the proteomic data (Model 1c).

On further investigation (eFigures 13–22), there were significant differences ($p<0.05$) between the 6 different site groups according to the Kruskall-Wallis test for 6 clinical variables: age, years in education, GAF symptoms subscale, GAF disability subscale, SANS total global score and BPRS total score. Post-hoc Dunn's tests with Bonferroni correction were performed for these 6 variables (eFigures 13, 14, 17, 18, 20, 21). The results indicated that site associations in the clinical data may have been driven primarily by differences in age between London vs. the other sites and Netherlands vs. the other sites (participants from London and Netherlands had slightly higher median age compared to the other sites), and possibly by differences in years

© 2020 Mongan D et al. *JAMA Psychiatry.*

in education between Netherlands vs. the other sites. Participants from the London site also had higher median total BPRS score compared to participants from most of the other sites.

Second, we trained SVM models to predict the site provenance of EU-GEI cases based firstly on the clinical data (69 clinical features as per Model 1b) and secondly on the proteomic data (166 proteomic features as per Model 1c). In both cases, in keeping with the original models, we used the LIBLINEAR program with area under the receiver-operating curve (AUC) as the performance evaluation criterion. Hyperplane weighting was enabled. Models were trained using 5-fold pooled cross-validation with 5 inner and 5 outer folds. We used a 'one-vs-all' scheme for multiclass prediction of the 6 EU-GEI site groupings that were used in the original models' leave-site-out cross-validation schemes, as shown below:

1. London vs. REST
2. Netherlands vs. REST
3. Melbourne vs REST
4. Switzerland/Austria vs. REST
5. Denmark/France vs REST
6. Spain/Brazil vs REST

The performance metrics and ROC curves for the multiclass site prediction models based on clinical data are shown in eTable 17 and eFigure 23, and for the proteomic data in eTable 18 and eFigure 24.

The results of this analysis indicated little evidence of systematic site associations for the proteomic data. The 95% confidence intervals (95% CI) for the AUC for prediction of each site included 0.5 (the value of no discrimination) with the exception of London vs. REST, although here the lower bound was close to 0.5 (AUC 0.61, 95% CI: 0.51 – 0.71).

In keeping with the Kruskall-Wallis analyses above, there was greater evidence of site associations in relation to the clinical data for London vs. REST (AUC 0.76, 95% CI 0.67 – 0.85) and Netherlands vs. REST (AUC 0.76, 95% CI 0.61 – 0.91), likely driven by the differences in age, education and total BPRS score for these sites.

6.4 Multivariate correction

© 2020 Mongan D et al. *JAMA Psychiatry.*

Since our models were predictive (rather than causal), the primary analyses did not include correction for possible covariates. However, to investigate this, we developed corrected versions of the SVM models using multivariate correction via principal components analysis (PCA). This was performed using the 'Extract variance components from data' pre-processing function in Neurominer v1.0 (https://www.pronia.eu/neurominer/) whereby a PCA was performed on a source matrix of covariates. Correlations (Spearman's correlation coefficient) between the eigenvariates from the PCA reduction were determined and those greater than 0.2 (indicating weak correlation) were identified. The target matrix was projected to the source matrix PCA space (90% retained variance), then back-reconstructed to the original input space without the identified PCA components, thus removing the variance associated with those components.

The primary covariates of interest were age, sex, BMI and years in education (as a proxy socio-economic measure) in Study 1 and sex, BMI and maternal social class in Study 2. However, we additionally corrected for tobacco use and ethnicity in the replication dataset for Study 1, due to evidence of baseline differences for these variables between CHR-T and CHR-NT in this dataset (see eTable 12).

For each corrected model, the covariates for which correction was performed are listed below:

**Model**	**Covariates in PCA**
**Model 1a (corrected):** clinical and proteomic	Age, sex, BMI, years in education
**Model 1b (corrected):** clinical	Age, sex, BMI, years in education
**Model 1c (corrected):** proteomic	Age, sex, BMI, years in education
**Model 2a (corrected):** proteomic, non-London	Age, sex, BMI, years in education
**Model 2b (corrected):** top 10 proteomic, non-London	Age, sex, BMI, years in education
**Model 3 (corrected):** replication	Age, sex, BMI, years in education, tobacco use, ethnicity
**Model 4 (corrected):** ALSPAC PEs	Sex, BMI, maternal social class
**Model S1 (corrected):** ELISA	Age, sex, BMI, years in education
**Model S2 (corrected):** functional outcome	Age, sex, BMI, years in education

Performance metrics of uncorrected and corrected models are presented in eTable 19. There were generally slight reductions in the AUCs of the corrected models when compared to their uncorrected counterparts (median change in AUC 0.04, range 0.01 – 0.10), although in all cases the 95% confidence intervals for the AUC of the uncorrected and corrected models overlapped.

7. Information on reporting of ethnicity

© 2020 Mongan D et al. *JAMA Psychiatry.*

In the current study, data was collected for ethnicity on the basis of potential relationships to the outcomes of interest.[32,33] Options for ethnicity were investigator-defined according to the Medical Research Council Socio-Demographic Schedule (amended)[34] in EU-GEI and a self-reported questionnaire in ALSPAC.

eReferences

1. Levin Y, Wang L, Schwarz E, Koethe D, Leweke FM, Bahn S. Global proteomic profiling reveals altered proteomic signature in schizophrenia serum. *Mol Psychiatry.* 2010;15(11):1088-1100.
2. Bradford MM. A rapid and sensitive method for the quantitation of microgram quantities of protein utilizing the principle of protein-dye binding. *Anal Biochem.* 1976;72:248-254.
3. English JA, Fan Y, Focking M, et al. Reduced protein synthesis in schizophrenia patient-derived olfactory cells. *Translational psychiatry.* 2015;5:e663.
4. English JA, Lopez LM, O'Gorman A, et al. Blood-Based Protein Changes in Childhood Are Associated With Increased Risk for Later Psychotic Disorder: Evidence From a Nested Case-Control Study of the ALSPAC Longitudinal Birth Cohort. *Schizophr Bull.* 2018;44(2):297-306.
5. Föcking M, Sabherwal S, Cates HM, et al. Complement pathway changes at age 12 are associated with psychotic experiences at age 18 in a longitudinal population-based study: evidence for a role of stress. *Molecular Psychiatry.* 2019.
6. Sabherwal S, English JA, Focking M, Cagney G, Cotter DR. Blood biomarker discovery in drug-free schizophrenia: the contributionof proteomics and multiplex immunoassays. *Expert review of proteomics.* 2016;13(12):1141-1155.
7. Sabherwal S, Focking M, English JA, et al. ApoE elevation is associated with the persistence of psychotic experiences from age 12 to age 18: Evidence from the ALSPAC birth cohort. *Schizophr Res.* 2019.
8. Ghale-Noie ZN, Hassani M, Kachooei AR, Kerachian MA. High Serum Alpha-2-Macroglobulin Level in Patients with Osteonecrosis of the Femoral Head. *The archives of bone and joint surgery.* 2018;6(3):219-224.
9. Bjelosevic S, Pascovici D, Ping H, et al. Quantitative Age-specific Variability of Plasma Proteins in Healthy Neonates, Children and Adults. *Molecular & cellular proteomics : MCP.* 2017;16(5):924-935.
10. Robinson MD, Mishra I, Deodhar S, et al. Water T2 as an early, global and practical biomarker for metabolic syndrome: an observational cross-sectional study. *Journal of translational medicine.* 2017;15(1):258.
11. Sunderic M, Mihailovic N, Nedic O. Protein molecular forms of insulin-like growth factor binding protein-2 change with aging. *Experimental gerontology.* 2014;58:154-158.
12. Banville N, Burgess JK, Jaffar J, et al. A quantitative proteomic approach to identify significantly altered protein networks in the serum of patients with lymphangioleiomyomatosis (LAM). *PloS one.* 2014;9(8):e105365.
13. Zeng J, Deng P, Li J, Feng W, Chen J, Zeng Y. Increased serum protein levels by Yuanshi Shengmai Chenggu Tablet in treatment of avascular osteonecrosis of the femoral head. *Molecular medicine reports.* 2018;17(2):2121-2126.
14. Zhou ZQ, Tong DN, Guan J, et al. Follicular helper T cell exhaustion induced by PD-L1 expression in hepatocellular carcinoma results in impaired cytokine expression and B cell help, and is associated with advanced tumor stages. *American journal of translational research.* 2016;8(7):2926-2936.
15. Guy JH, Pyne DB, Deakin GB, Miller CM, Edwards AM. Acclimation Training Improves Endurance Cycling Performance in the Heat without Inducing Endotoxemia. *Frontiers in physiology.* 2016;7:318.
16. Ferrin G, Rodriguez-Peralvarez M, Aguilar-Melero P, et al. Plasma protein biomarkers of hepatocellular carcinoma in HCV-infected alcoholic patients with cirrhosis. *PloS one.* 2015;10(3):e0118527.
17. Liang Y, Xie SB, Wu CH, et al. Coagulation cascade and complement system in systemic lupus erythematosus. *Oncotarget.* 2018;9(19):14862-14881.
18. Ting KR, Henry M, Meiller J, et al. Novel panel of protein biomarkers to predict response to bortezomib-containing induction regimens in multiple myeloma patients. *BBA clinical.* 2017;8:28-34.

© 2020 Mongan D et al. *JAMA Psychiatry.*

19. McGuire JL, Gill AJ, Douglas SD, Kolson DL. The complement system, neuronal injury, and cognitive function in horizontally-acquired HIV-infected youth. *Journal of neurovirology.* 2016;22(6):823-830.
20. Lui H, Zhang J, Makinson SR, et al. Progranulin Deficiency Promotes Circuit-Specific Synaptic Pruning by Microglia via Complement Activation. *Cell.* 2016;165(4):921-935.
21. Fan R-E, Chang K-W, Hsieh C-J, Wang X-R, Lin C-J. LIBLINEAR: A Library for Large Linear Classification. *J Mach Learn Res.* 2008;9:1871-1874.
22. Krawczyk B. Learning from imbalanced data: open challenges and future directions. *Progress in Artificial Intelligence.* 2016;5(4):221-232.
23. Gaonkar B, Davatzikos C. Analytic estimation of statistical significance maps for support vector machine based multi-variate image analysis and classification. *Neuroimage.* 2013;78:270-283.
24. Koutsouleris N, Kahn RS, Chekroud AM, et al. Multisite prediction of 4-week and 52-week treatment outcomes in patients with first-episode psychosis: a machine learning approach. *The Lancet Psychiatry.* 2016;3(10):935-946.
25. Golland P, Fischl B. Permutation Tests for Classification: Towards Statistical Significance in Image-Based Studies. Paper presented at: Information Processing in Medical Imaging; 2003//, 2003; Berlin, Heidelberg.
26. Koutsouleris N, Kahn RS, Chekroud AM, et al. Multisite prediction of 4-week and 52-week treatment outcomes in patients with first-episode psychosis: a machine learning approach. *Lancet Psychiatry.* 2016;3(10):935-946.
27. Hanley JA, McNeil BJ. The meaning and use of the area under a receiver operating characteristic (ROC) curve. *Radiology.* 1982;143(1):29-36.
28. Aas IH. Global Assessment of Functioning (GAF): properties and frontier of current knowledge. *Ann Gen Psychiatry.* 2010;9:20.
29. Goldman HH, Skodol AE, Lave TR. Revising axis V for DSM-IV: a review of measures of social functioning. *Am J Psychiatry.* 1992;149(9):1148-1156.
30. Koutsouleris N, Kambeitz-Ilankovic L, Ruhrmann S, et al. Prediction Models of Functional Outcomes for Individuals in the Clinical High-Risk State for Psychosis or With Recent-Onset Depression: A Multimodal, Multisite Machine Learning Analysis. *JAMA psychiatry.* 2018;75(11):1156-1172.
31. Dinno A. Nonparametric pairwise multiple comparisons in independent groups using Dunn's test. *Stata Journal.* 2015;15(1):292-300.
32. Morgan C, Charalambides M, Hutchinson G, Murray RM. Migration, ethnicity, and psychosis: toward a sociodevelopmental model. *Schizophr Bull.* 2010;36(4):655-664.
33. Kelleher I, Cannon M. Psychotic-like experiences in the general population: characterizing a high-risk group for psychosis. *Psychological medicine.* 2011;41(1):1-6.
34. Mallett R. Sociodemographic Schedule. Section of Social Psychiatry, Institute of Psychiatry; London: 1997

© 2020 Mongan D et al. *JAMA Psychiatry.*

eTable 1: Summary of support vector machine models

Model	Aim	Source cohort	Target outcome to be predicted	Data modalities	Repeated nested cross-validation method	Development data (*n* in each group)	Test data (*n* in each group)
**Model 1a**	Predict transition in CHR sample using baseline clinical and proteomic data	EU-GEI	Transition status	69 clinical and 166 proteomic features	Leave-site-out	Initial experiment, all sites (49 CHR-T, 84 CHR-NT)	N/A
**Model 1b**	Predict transition in CHR sample using baseline clinical data	EU-GEI	Transition status	69 clinical features	Leave-site-out	Initial experiment, all sites (49 CHR-T, 84 CHR-NT)	N/A
**Model 1c**	Predict transition in CHR sample using baseline proteomic data	EU-GEI	Transition status	166 proteomic features	Leave-site-out	Initial experiment, all sites (49 CHR-T, 84 CHR-NT)	N/A
**Model 2a**	Derivation of 10 highest-weighted proteomic features	EU-GEI	Transition status	166 proteomic features	Leave-site-out	Initial experiment, all sites except London (30 CHR-T, 50 CHR-NT)	N/A
**Model 2b**	Predict transition in CHR sample using 10 proteomic features, and test in held-out data	EU-GEI	Transition status	10 highest-weighted proteomic features from Model 2a	Leave-site-out	Initial experiment, all sites except London (30 CHR-T, 50 CHR-NT)	Initial experiment, London site (19 CHR-T, 34 CHR-NT)
**Model 3**	Replicate findings from Model 1a	EU-GEI	Transition status	69 clinical and 119 proteomic features	Leave-site-out	Replication experiment, all sites (49 CHR-T, 86 CHR-NT)	N/A
**Model 4**	Predict age 18 psychotic experiences in general population sample using proteomic	ALSPAC	Psychotic experiences (PEs)	265 proteomic features	Pooled (5 random inner and outer folds)	ALSPAC subsample (55 PE, 66 no PE)	N/A

© 2020 Mongan D et al. *JAMA Psychiatry.*

	data at age 12						
**Supplementary models**							
**Model**	**Aim**	**Source cohort**	**Target outcome to be predicted**	**Data modalities**	**Repeated nested cross-validation method**	**Development data (*n* in each group)**	**Test data (*n* in each group)**
**Model S1**	Predict transition in CHR sample using baseline ELISA results	EU-GEI	Transition status	9 ELISA features	Leave-site-out	Initial experiment, all sites (44 CHR-T, 82 CHR-NT)	N/A
**Model S2**	Predict functional outcome in CHR sample using baseline clinical and proteomic data	EU-GEI	Functional outcome	69 clinical and 166 proteomic features	Pooled (5 random inner and outer folds)	Initial experiment, all sites (47 GAF≤60, 32 GAF>60)	N/A

CHR: clinical high risk; ELISA: enzyme-linked immunosorbent assay; EU-GEI: European Network of National Schizophrenia Networks Studying Gene-Environment Interactions; ALSPAC: Avon Longitudinal Study of Parents and Children

© 2020 Mongan D et al. *JAMA Psychiatry.*

eTable 2: List of 69 baseline EU-GEI clinical features included in Model 1a, Model 1b and Model 3

Age
Sex
Body mass index
Years in education
GAF symptoms
GAF disability
SANS: unchanging facial expression
SANS: decreased spontaneous movements
SANS: paucity of expressive gestures
SANS: poor eye contact
SANS: affective non-responsivity
SANS: inappropriate affect
SANS: lack of vocal inflections
SANS: global rating of affective flattening
SANS: poverty of speech
SANS: poverty of speech content
SANS: blocking
SANS: increased latency of response
SANS: global rating of alogia
SANS: grooming and hygiene
SANS: impersistence at work or school
SANS: physical anergia
SANS: global rating for avolition - apathy
SANS: recreational interests and activities
SANS: sexual activity
SANS: ability to feel intimacy and closeness
SANS: relationship with friends and peers
SANS: global rating of anhedonia - asociality
SANS: social inattentiveness
SANS: inattentiveness during mental status testing
SANS: global rating of attention
Total SANS composite score
Total SANS global score
BPRS: somatic concern
BPRS: anxiety
BPRS: depression
BPRS: suicidality
BPRS: guilt
BPRS: hostility

© 2020 Mongan D et al. *JAMA Psychiatry.*

BPRS: elevated mood
BPRS: grandiosity
BPRS: suspiciousness
BPRS: hallucinations
BPRS: unusual thought content
BPRS: bizarre behaviour
BPRS: self-neglect
BPRS: disorientation
BPRS: conceptual disorganisation
BPRS: blunted affect
BPRS: emotional withdrawal
BPRS: motor retardation
BPRS: tension
BPRS: uncooperativeness
BPRS: excitement
BPRS: distractibility
BPRS: motor hyperactivity
BPRS: mannersims and posturing
Total BPRS score
MADRS: apparent sadness
MADRS: reported sadness
MADRS: inner tension
MADRS: reduced sleep
MADRS: reduced appetite
MADRS: concentration difficulties
MADRS: lassitude
MADRS: inability to feel
MADRS: pessimistic thoughts
MADRS: suicidal thoughts
Total MADRS score

GAF: General Assessment of Functioning; SANS: Scale for the Assessment of Negative Symptoms; BPRS: Brief Psychiatric Rating Scale; MADRS: Montgomery Asberg Depression Rating Scale; EU-GEI: European Network of National Schizophrenia Networks Studying Gene-Environment Interactions

© 2020 Mongan D et al. *JAMA Psychiatry.*

eTable 3: Comparison of baseline characteristics for CHR participants who attended at least one follow-up interview vs. CHR participants who did not in EU-GEI

	Missing data, n (%)	Attended at least one follow-up interview, N=171	Did not attend at least one follow-up interview, N=173	t/ χ^2	*p*	Corrected *p* (FDR 5%)
**Baseline age in years, mean (SD)**	0	23.0 (5.1)	21.8 (4.7)	2.240	0.026	0.104
**Sex, n (%)**	0	92 male (53.8%)<br>79 female (46.2%)	93 male (53.8%)<br>80 female (46.2%)	0.000	0.993	0.993
**Baseline BMI in kg/m^2, mean (SD)**	53 (15.4%)	23.9 (5.2)	24.0 (5.1)	-0.274	0.784	0.941
**Baseline years in education, mean (SD)**	41 (11.9%)	15.0 (2.8)	13.7 (3.2)	3.689	<0.001	<0.008
**Ethnicity, n (%)**	3 (0.9%)	124 white (72.1%)<br>18 black (10.5%)<br>30 other (17.4%)	122 white (72.2%)<br>15 black (8.9%)<br>32 other (18.9%)	0.327	0.849	0.941
**Ever used cannabis, n (%)**	13 (3.8%)	129 yes (77.2%)<br>38 no (22.8%)	112 yes (68.3%)<br>52 no (31.7%)	3.350	0.067	0.214
**Baseline cannabis use, n (%)**	98 (28.5%)	46 yes (35.4%)<br>84 no (64.6%)	40 yes (34.5%)<br>76 no (65.5%)	0.022	0.882	0.941
**Baseline tobacco use, n (%)**	41 (11.9%)	89 yes (55.6%)<br>71 no (44.4%)	71 yes (49.7%)<br>72 no (50.3%)	1.082	0.298	0.681
**Baseline alcohol use, n (%)**	15 (4.4%)	117 yes (69.6%)<br>51 no (30.4%)	116 yes (72.0%)<br>45 no (28.0%)	0.230	0.631	0.941
**Baseline medication use, n (%)**	89 (25.9%)	69 yes (50.4%)<br>Antipsychotic 9<br>Antidepressant 54<br>Hypnotic 15<br>Other 21<br>68 no (49.6%)	57 yes (48.3%)<br>Antipsychotic 22<br>Antidepressant 37<br>Hypnotic 8<br>Other 15<br>61 no (51.7%)	0.108	0.743	0.941
**Baseline GAF symptoms score, mean (SD)**	27 (7.8%)	55.3 (9.9)	54.9 (10.3)	0.280	0.779	0.941
**Baseline GAF disability score, mean (SD)**	12 (3.5%)	56.5 (12.0)	54.3 (12.5)	1.613	0.108	0.288

© 2020 Mongan D et al. *JAMA Psychiatry.*

**Baseline SANS total composite score, mean (SD)**	48 (14.0%)	14.0 (10.5)	17.5 (12.7)	-2.600	0.010	0.053
**Baseline SANS total global score, mean (SD)**	32 (9.3%)	4.8 (3.4)	6.2 (3.7)	-3.357	0.001	0.008
**Baseline BPRS total score, mean (SD)**	28 (8.1%)	43.1 (10.7)	44.0 (9.7)	-0.794	0.428	0.856
**Baseline MADRS total score, mean (SD)**	19 (5.5%)	18.8 (9.1)	19.0 (9.2)	-0.261	0.794	0.941

Tobacco use was defined as daily use for at least 1 month over the previous 12 months.

Alcohol use was defined as at least 12 or more alcoholic beverages over the previous 12 months.

Missing data excluded in hypothesis tests.

EU-GEI: European Network of National Schizophrenia Networks Studying Gene-Environment Interactions; CHR-T: clinical high risk, transitioned to psychosis; CHR-NT: clinical high risk, did not transition to psychosis; FDR: false discovery rate; BMI: body mass index; GAF: General Assessment of Functioning; SANS: Scale for the Assessment of Negative Symptoms; BPRS: Brief Psychiatric Rating Scale; MADRS: Montgomery Asberg Depression Rating Scale

© 2020 Mongan D et al. *JAMA Psychiatry.*

eTable 4: Comparison of characteristics for participants included in initial experiment (N=133) from total EU-GEI clinical high-risk cohort (N=344)

	Missing data, n (%)	Included, N = 133<br><br>(49 CHR-T,<br>84 CHR-NT)	Not included, N = 211<br><br>(16 CHR-T,<br>195 CHR-NT)	t/ χ^2	*p*	Corrected *p* (FDR 5%)
**Baseline age in years, mean (SD)**	0	22.6 (4.5)	22.3 (5.2)	0.686	0.493	0.681
**Gender, n (%)**	0	68 male (51.1%)<br><br>65 female (49.9%)	117 male (55.5%)<br><br>94 female (45.5%)	0.613	0.434	0.681
**Baseline BMI in kg/m^2, mean (SD)**	50 (14.5%)	24.4 (5.6)	23.7 (4.9)	1.190	0.235	0.439
**Baseline years in education, mean (SD)**	38 (11.0%)	14.3 (3.1)	14.4 (3.0)	-0.318	0.751	0.793
**Ethnicity, n (%)**	0	91 white (68.4%)<br><br>15 black (11.3%)<br><br>27 other (20.3%)	156 white (73.9%)<br><br>19 black (9.0%)<br><br>36 other (17.1%)	1.239	0.538	0.681
**Ever used cannabis, n (%)**	10 (2.9%)	101 yes (75.9%)<br><br>29 no (21.8%)<br><br>3 not known (2.3%)	143 yes (67.8%)<br><br>61 no (28.9%)<br><br>7 not known (3.3%)	2.326	0.127	0.302
**Baseline cannabis use, n (%)**	95 (27.6%)	41 yes (30.8%)<br><br>63 no (47.4%)<br><br>29 not known (21.8%)	47 yes (22.3%)<br><br>98 no (46.4%)<br><br>66 not known (31.3%)	1.302	0.254	0.439
**Baseline tobacco use, n (%)**	38 (11.0%)	64 yes (48.1%)<br><br>55 no (41.4%)<br><br>14 not known (10.5%)	97 yes (46.0%)<br><br>90 no (42.7%)<br><br>24 not known (11.4%)	0.106	0.744	0.793
**Baseline alcohol use, n (%)**	12 (3.5%)	93 yes (69.9%)<br><br>37 no (27.8%)<br><br>3 not known (2.3%)	141 yes (66.8%)<br><br>61 no (28.9%)<br><br>9 not known (4.3%)	0.115	0.735	0.793
**Baseline medication use, n (%)**	87 (25.3%)	51 yes (38.3%)<br>Antidepressant 38<br>Antipsychotic 15<br>Hypnotic 9<br>Other 16<br><br>51 no (38.3%)	77 yes (36.5%)<br>Antidepressant 53<br>Antipsychotic 16<br>Hypnotic 14<br>Other 20<br><br>78 no (37.0%)	0.003	0.960	0.960

© 2020 Mongan D et al. *JAMA Psychiatry.*

		31 not known (23.3%)	56 not known (26.5%)			
**Baseline GAF symptoms score, mean (SD)**	27 (7.8%)	54.7 (10.2)	55.4 (10.0)	-0.634	0.526	0.681
**Baseline GAF disability score, mean (SD)**	12 (3.5%)	53.9 (11.7)	56.4 (12.5)	-1.827	0.069	0.187
**Baseline SANS total composite score, mean (SD)**	45 (13.1%)	18.0 (12.7)	14.2 (10.7)	2.782	0.006	0.029
**Baseline SANS total global score, mean (SD)**	29 (8.4%)	6.1 (3.9)	5.0 (3.4)	2.528	0.012	0.046
**Baseline BPRS total score, mean (SD)**	25 (7.3%)	46.0 (10.9)	42.2 (9.6)	3.174	0.002	0.013
**Baseline MADRS total score, mean (SD)**	16 (4.7%)	19.6 (9.7)	18.4 (8.8)	1.155	0.249	0.439
**2 year GAF symptoms score, mean (SD)**	142 (41.3%)	54.6 (15.0)	63.0 (11.6)	-4.083	<0.001	<0.010
**2 year GAF disability score, mean (SD)**	124 (36.0%)	56.9 (15.0)	63.6 (13.8)	-3.333	0.001	0.010
**2 year GAF disability score, dichotomous outcome [a]**	124 (36.0%)	32 good (24.1%)<br>47 poor (35.3%)<br>54 not known (40.6%)	80 good (37.9%)<br>61 poor (28.9%)<br>70 not known (33.2%)	5.337	0.021	0.067

[a] Poor functioning: GAF disability score ≤60; good functioning: GAF disability score >60

Tobacco use was defined as daily use for at least 1 month over the previous 12 months. Alcohol use was defined as at least 12 or more alcoholic beverages over the previous 12 months. Missing data excluded in hypothesis tests.

EU-GEI: European Network of National Schizophrenia Networks Studying Gene-Environment Interactions; CHR-T: clinical high risk, transitioned to psychosis; CHR-NT: clinical high risk, did not transition to psychosis; FDR: false discovery rate; BMI: body mass index; GAF: General Assessment of Functioning; SANS: Scale for the Assessment of Negative Symptoms; BPRS: Brief Psychiatric Rating Scale; MADRS: Montgomery Asberg Depression Rating Scale

© 2020 Mongan D et al. *JAMA Psychiatry.*

eTable 5: Results of ANCOVA (adjusted for age, sex, BMI and years in education) and fold changes (CHR-T vs. CHR-NT) for proteins identified in EU-GEI baseline plasma samples in the initial experiment

Uniprot No.	Protein name	F	*p*	Corrected *p* (5% FDR)	Direction of effect (T vs. NT)	Ratio of means (T vs. NT)
P01023	Alpha-2-macroglobulin	146	7.55E-23	1.25E-20	↓	0.33
P01871	Immunoglobulin heavy constant mu	72.16	4.53E-14	3.76E-12	↓	0.41
P07357	Complement component C8 alpha chain	44.25	7.76E-10	4.29E-08	↑	1.48
P02774	Vitamin D-binding protein	40.97	2.72E-09	1.13E-07	↑	1.43
P02747	Complement C1q subcomponent subunit C	36.52	1.56E-08	5.18E-07	↑	1.53
P00747	Plasminogen	31.39	1.25E-07	3.46E-06	↑	1.29
P10909	Clusterin	29.74	2.48E-07	5.88E-06	↑	1.29
P23142	Fibulin-1	28.56	4.06E-07	8.42E-06	↑	1.52
P55058	Phospholipid transfer protein	19.09	2.57E-05	0.0005	↓	0.67
P00736	Complement C1r subcomponent	18.07	4.09E-05	0.0007	↑	1.27
O75882	Attractin	16.85	0.0001	0.0011	↑	1.30
P03951	Coagulation factor XI	17.61	0.0001	0.0011	↑	1.36
P05156	Complement factor I	16.72	0.0001	0.0011	↑	1.23
P08603	Complement factor H	16.18	0.0001	0.0011	↑	1.16
P43320	Beta-crystallin B2	16.39	0.0001	0.0011	↑	1.80
P04003	C4b-binding protein alpha chain	14.71	0.0002	0.0020	↓	0.76
P19827	Inter-alpha-trypsin inhibitor heavy chain H1	15.05	0.0002	0.0020	↑	1.19
O75636	Ficolin-3	14.09	0.0003	0.0028	↓	0.70
P01860	Immunoglobulin heavy constant gamma 3	13.19	0.0004	0.0033	↓	0.70
P15144	Aminopeptidase N	13.38	0.0004	0.0033	↓	0.72
P02489	Alpha-crystallin A chain	12.08	0.0007	0.0055	↑	1.63
P06396	Gelsolin	11.83	0.0008	0.0058	↑	1.17
Q14520	Hyaluronan-binding protein 2	11.87	0.0008	0.0058	↑	1.21
P05155	Plasma protease C1 inhibitor	11.01	0.0012	0.0083	↓	0.85
P02766	Transthyretin	10.79	0.0013	0.0086	↓	0.60
P02749	Beta-2-glycoprotein 1	9.93	0.0020	0.0123	↑	1.27
P04217	Alpha-1B-glycoprotein	9.97	0.0020	0.0123	↓	0.83
P22891	Vitamin K-dependent protein Z	9.86	0.0021	0.0125	↓	0.68
P00751	Complement factor B	9.41	0.0026	0.0149	↑	1.17
P05546	Heparin cofactor 2	8.91	0.0034	0.0188	↓	0.84
P06276	Cholinesterase	8.71	0.0038	0.0203	↑	1.18
P51884	Lumican	8.4	0.0044	0.0228	↑	1.25
P02649	Apolipoprotein E	8.25	0.0048	0.0241	↑	1.28
Q76LX8	A disintegrin and metalloproteinase with thrombospondin motifs 13	7.56	0.0068	0.0332	↓	0.84

© 2020 Mongan D et al. *JAMA Psychiatry.*

Q06033	Inter-alpha-trypsin inhibitor heavy chain H3	7.15	0.0085	0.0403	↓	0.74
P02656	Apolipoprotein C-III	6.59	0.0114	0.0526	↑	1.37
P02751	Fibronectin	6.43	0.0124	0.0542	↑	1.30
P05543	Thyroxine-binding globulin	6.43	0.0124	0.0542	↑	1.18
P00450	Ceruloplasmin	6.32	0.0132	0.0562	↑	1.14
Q04756	Hepatocyte growth factor activator	5.69	0.0185	0.0768	↑	1.14
P05090	Apolipoprotein D	5.61	0.0193	0.0781	↑	1.17
Q08380	Galectin-3-binding protein	5.38	0.0219	0.0866	↓	0.77
P11226	Mannose-binding protein C	5.18	0.0246	0.0924	↑	1.36
P10643	Complement component C7	5.17	0.0247	0.0924	↑	1.17
P07225	Vitamin K-dependent protein S	5.12	0.0254	0.0924	↓	0.87
Q9BXR6	Complement factor H-related protein 5	5.1	0.0256	0.0924	↑	1.30
P49747	Cartilage oligomeric matrix protein	4.94	0.0280	0.0989	↑	1.16
P02675	Fibrinogen beta chain	4.79	0.0305	0.1033	↑	1.15
P02671	Fibrinogen alpha chain	4.77	0.0307	0.1033	↑	1.12
P04114	Apolipoprotein B-100	4.75	0.0311	0.1033	↓	0.89
P01042	Kininogen-1	4.69	0.0322	0.1048	↑	1.09
P43652	Afamin	4.49	0.0361	0.1152	↑	1.12
P05160	Coagulation factor XIII B chain	4.26	0.0410	0.1254	↑	1.14
Q9NZP8	Complement C1r subcomponent-like protein	4.23	0.0417	0.1254	↑	1.27
P02753	Retinol-binding protein 4	4.22	0.0421	0.1254	↑	1.36
P00742	Coagulation factor X	4.21	0.0423	0.1254	↑	1.11
P36980	Complement factor H-related protein 2	3.72	0.0559	0.1628	↑	1.31
P07195	L-lactate dehydrogenase B chain	3.68	0.0572	0.1637	↑	1.16
P02748	Complement component C9	3.64	0.0585	0.1646	↓	0.91
P01024	Complement C3	3.56	0.0615	0.1702	↑	1.13
P01876	Immunoglobulin heavy constant alpha 1	3.4	0.0675	0.1837	↑	1.14
P02743	Serum amyloid P-component	3.33	0.0705	0.1888	↑	1.12
P05452	Tetranectin	3.21	0.0756	0.1992	↑	1.11
P20742	Pregnancy zone protein	2.87	0.0930	0.2412	↓	0.76
P00740	Coagulation factor IX	2.83	0.0949	0.2424	↑	1.12
P01859	Immunoglobulin heavy constant gamma 2	2.55	0.1125	0.2830	↑	1.13
P00748	Coagulation factor XII	2.49	0.1170	0.2899	↑	1.14
P68871	Hemoglobin subunit beta	2.3	0.1315	0.3166	↑	1.33
P02787	Serotransferrin	2.3	0.1316	0.3166	↑	1.05
P01011	Alpha-1-antichymotrypsin	2.27	0.1347	0.3194	↑	1.06
P07737	Profilin-1	2.07	0.1526	0.3568	↓	0.75
Q92954	Proteoglycan 4	2.03	0.1565	0.3608	↓	0.88
P13671	Complement component C6	1.86	0.1745	0.3968	↑	1.06
P09871	Complement C1s subcomponent	1.84	0.1776	0.3984	↑	1.06
P07358	Complement component C8 beta chain	1.77	0.1856	0.4054	↓	0.91
Q03591	Complement factor H-related protein 1	1.77	0.1856	0.4054	↑	1.19

© 2020 Mongan D et al. *JAMA Psychiatry.*

P02654	Apolipoprotein C-I	1.55	0.2147	0.4629	↓	0.69
P48740	Mannan-binding lectin serine protease 1	1.43	0.2336	0.4971	↑	1.10
P02760	Protein AMBP	1.41	0.2371	0.4982	↑	1.08
P02790	Hemopexin	1.37	0.2439	0.5033	↓	0.94
P29622	Kallistatin	1.34	0.2484	0.5033	↑	1.06
P04004	Vitronectin	1.34	0.2486	0.5033	↓	0.96
P14618	Pyruvate kinase PKM	1.32	0.2530	0.5060	↓	0.84
P22792	Carboxypeptidase N subunit 2	1.26	0.2639	0.5215	↑	1.06
P17936	Insulin-like growth factor-binding protein 3	1.21	0.2727	0.5275	↓	0.91
Q96PD5	N-acetylmuramoyl-L-alanine amidase	1.21	0.2734	0.5275	↓	0.93
P01019	Angiotensinogen	1.19	0.2767	0.5275	↓	0.89
Q96KN2	Beta-Ala-His dipeptidase	1.17	0.2808	0.5275	↓	0.92
P02652	Apolipoprotein A-II	1.16	0.2828	0.5275	↓	0.97
P27169	Serum paraoxonase/arylesterase 1	1.13	0.2894	0.5338	↑	1.11
P22105	Tenascin-X	1.08	0.3001	0.5474	↑	1.13
P09172	Dopamine beta-hydroxylase	0.95	0.3314	0.5980	↑	1.11
P60174	Triosephosphate isomerase	0.89	0.3481	0.6213	↓	0.86
P15169	Carboxypeptidase N catalytic chain	0.8	0.3724	0.6411	↑	1.07
P08185	Corticosteroid-binding globulin	0.8	0.3725	0.6411	↓	0.87
P23528	Cofilin-1	0.8	0.3737	0.6411	↑	1.11
P80108	Phosphatidylinositol-glycan-specific phospholipase D	0.79	0.3746	0.6411	↑	1.08
P02775	Platelet basic protein	0.78	0.3801	0.6438	↓	0.78
P06681	Complement C2	0.72	0.3974	0.6663	↓	0.94
P00734	Prothrombin	0.67	0.4130	0.6745	↑	1.03
P60709	Actin, cytoplasmic 1	0.67	0.4157	0.6745	↑	1.03
P35858	Insulin-like growth factor-binding protein complex acid labile subunit	0.66	0.4173	0.6745	↓	0.94
P02647	Apolipoprotein A-I	0.66	0.4185	0.6745	↓	0.89
P02763	Alpha-1-acid glycoprotein 1	0.63	0.4285	0.6769	↓	0.99
P04075	Fructose-bisphosphate aldolase A	0.62	0.4332	0.6769	↓	0.95
P18428	Lipopolysaccharide-binding protein	0.62	0.4335	0.6769	↓	0.91
P01834	Immunoglobulin kappa constant	0.61	0.4363	0.6769	↓	0.98
P04196	Histidine-rich glycoprotein	0.58	0.4474	0.6877	↑	1.08
Q9Y6R7	IgGFc-binding protein	0.56	0.4560	0.6906	↑	1.03
P08697	Alpha-2-antiplasmin	0.55	0.4594	0.6906	↓	0.97
Q14624	Inter-alpha-trypsin inhibitor heavy chain H4	0.54	0.4618	0.6906	↑	1.02
P00738	Haptoglobin	0.53	0.4680	0.6936	↓	0.60
O43866	CD5 antigen-like	0.5	0.4793	0.6981	↓	0.93
P0C0L5	Complement C4-B	0.5	0.4794	0.6981	↑	1.12
Q12913	Receptor-type tyrosine-protein phosphatase eta	0.49	0.4863	0.7020	↑	1.04
P04070	Vitamin K-dependent protein C	0.44	0.5103	0.7303	↓	0.88
P12814	Alpha-actinin-1	0.41	0.5251	0.7388	↓	0.82

© 2020 Mongan D et al. *JAMA Psychiatry.*

P11021	Endoplasmic reticulum chaperone BiP	0.41	0.5252	0.7388	↑	1.02
P04275	von Willebrand factor	0.36	0.5505	0.7679	↑	1.08
P36955	Pigment epithelium-derived factor	0.33	0.5696	0.7839	↑	1.04
P03952	Plasma kallikrein	0.32	0.5714	0.7839	↑	1.03
P07360	Complement component C8 gamma chain	0.3	0.5837	0.7928	–	1.00
P02750	Leucine-rich alpha-2-glycoprotein	0.29	0.5908	0.7928	↑	1.03
Q15582	Transforming growth factor-beta-induced protein ig-h3	0.29	0.5922	0.7928	↓	0.95
P49908	Selenoprotein P	0.27	0.6054	0.8027	↑	1.06
Q16610	Extracellular matrix protein 1	0.26	0.6124	0.8027	↑	1.01
P19823	Inter-alpha-trypsin inhibitor heavy chain H2	0.26	0.6141	0.8027	↓	0.97
P19320	Vascular cell adhesion protein 1	0.23	0.6290	0.8142	↓	0.93
P43251	Biotinidase	0.23	0.6327	0.8142	↓	0.91
P01031	Complement C5	0.19	0.6677	0.8491	↓	0.98
Q86UX7	Fermitin family homolog 3	0.18	0.6701	0.8491	↓	0.87
P21333	Filamin-A	0.18	0.6763	0.8505	↓	0.94
P00488	Coagulation factor XIII A chain	0.13	0.7166	0.8917	↓	0.96
Q9UGM5	Fetuin-B	0.13	0.7198	0.8917	↓	0.98
P04278	Sex hormone-binding globulin	0.11	0.7403	0.9103	↓	0.86
P30041	Peroxiredoxin-6	0.09	0.7667	0.9266	↓	0.99
P02746	Complement C1q subcomponent subunit B	0.09	0.7703	0.9266	↑	1.02
P02679	Fibrinogen gamma chain	0.08	0.7720	0.9266	↑	1.02
Q9NPH3	Interleukin-1 receptor accessory protein	0.08	0.7759	0.9266	↓	0.86
P00338	L-lactate dehydrogenase A chain	0.07	0.7934	0.9407	↑	1.02
P07996	Thrombospondin-1	0.06	0.8058	0.9439	↓	0.91
P25311	Zinc-alpha-2-glycoprotein	0.06	0.8074	0.9439	↑	1.01
Q9Y490	Talin-1	0.06	0.8149	0.9460	↓	0.89
P0CG06	Immunoglobulin lambda constant 3	0.05	0.8228	0.9485	↓	0.88
Q96IY4	Carboxypeptidase B2	0.03	0.8738	0.9920	↓	0.96
O00533	Neural cell adhesion molecule L1-like protein	0.02	0.8774	0.9920	↑	1.03
P08571	Monocyte differentiation antigen CD14	0.02	0.8899	0.9920	↑	1.03
P02765	Alpha-2-HS-glycoprotein	0.02	0.8933	0.9920	↑	1.01
P01857	Immunoglobulin heavy constant gamma 1	0.01	0.9034	0.9920	↓	0.98
P01008	Antithrombin-III	0.01	0.9108	0.9920	↑	1.01
P00915	Carbonic anhydrase 1	0.01	0.9113	0.9920	↑	1.05
Q6EMK4	Vasorin	0.01	0.9118	0.9920	↓	0.96
O00391	Sulfhydryl oxidase 1	0.01	0.9143	0.9920	↓	0.99
P12259	Coagulation factor V	0.01	0.9247	0.9928	–	1.00
P07359	Platelet glycoprotein Ib alpha chain	0.01	0.9291	0.9928	↓	0.98
P06727	Apolipoprotein A-IV	0.01	0.9391	0.9928	↑	1.01
P26927	Hepatocyte growth factor-like protein	0	0.9457	0.9928	↓	0.98
P13796	Plastin-2	0	0.9503	0.9928	↑	1.01

© 2020 Mongan D et al. *JAMA Psychiatry.*

P63104	14-3-3 protein zeta/delta	0	0.9538	0.9928	↓	0.99
P01623	Immunoglobulin kappa variable 3-20	0	0.9579	0.9928	↓	0.92
P05154	Plasma serine protease inhibitor	0	0.9629	0.9928	↓	0.95
P0C0L4	Complement C4-A	0	0.9748	0.9978	↑	1.01
P22352	Glutathione peroxidase 3	0	0.9805	0.9978	↓	0.94
P18206	Vinculin	0	0.9858	0.9978	↓	0.91
Q9UK55	Protein Z-dependent protease inhibitor	0	0.9926	0.9986	↓	0.99
P01009	Alpha-1-antitrypsin	0	0.9993	0.9993	↓	0.86

CHR-T: clinical high-risk participants who transitioned to first episode psychosis; CHR-NT: clinical high-risk participants who did not transition; FDR: false discovery rate; EU-GEI: European Network of National Schizophrenia Networks Studying Gene-Environment Interactions

© 2020 Mongan D et al. *JAMA Psychiatry*.

eTable 6: Coefficients of variation for proteins across quality control standards in EU-GEI initial experiment

Protein	Coefficient of variation (%)
**35 significantly differentially expressed proteins on analysis of covariance:**	
Alpha-2-macroglobulin	5.9
Immunoglobulin heavy constant mu	8.7
Complement component C8 alpha chain	20.2
Vitamin D-binding protein	20.3
Complement C1q subcomponent subunit C	15.0
Plasminogen	17.0
Clusterin	13.5
Fibulin-1	29.7
Phospholipid transfer protein	16.5
Complement C1r subcomponent	14.8
Attractin	12.0
Coagulation factor XI	10.5
Complement factor I	7.8
Complement factor H	9.1
Beta-crystallin B2	33.6
C4b-binding protein alpha chain	10.0
Inter-alpha-trypsin inhibitor heavy chain H1	13.5
Ficolin-3	12.0
Immunoglobulin heavy constant gamma 3	5.1
Aminopeptidase N	8.6
Alpha-crystallin A chain	25.7
Gelsolin	12.4
Hyaluronan-binding protein 2	11.1
Plasma protease C1 inhibitor	13.7
Transthyretin	29.8
Beta-2-glycoprotein 1	17.8
Alpha-1B-glycoprotein	14.0
Vitamin K-dependent protein Z	19.6
Complement factor B	5.6
Heparin cofactor 2	11.0
Cholinesterase	11.0

© 2020 Mongan D et al. *JAMA Psychiatry.*

Lumican	8.9
Apolipoprotein E	13.2
A disintegrin and metalloproteinase with thrombospondin motifs 13	38.1
Inter-alpha-trypsin inhibitor heavy chain H3	14.1
**Proteins not included in list above that were among 10% highest-weighted proteins in Model 1a:**	
Vitamin K dependent protein S	12.3
Complement component 6	7.0
Retinol-binding protein 4	33.1
Alpha-2-antiplasmin	14.2
**Proteins not included in both lists above that were among the 10 proteins included in Model 2b:**	
N-acetylmuramoyl-L-alanine- amidase	9.5

© 2020 Mongan D et al. *JAMA Psychiatry.*

eTable 7: Summary of protein-protein interactions identified from the BIOGRID database for significantly differentially expressed proteins between CHR-T and CHR-NT in EU-GEI initial experiment

Interactor A	Interactor B	Experimental system type	Notes	Source (Pubmed ID)
Alpha-2-macroglobulin	Apolipoprotein E	Affinity capture – Western	Two-dimensional non-denaturing gradient gel electrophoresis of human plasma resulted in the separation of an apolipoprotein E-containing complex which co-migrated with plasma alpha-2-macroglobulin	Krimbou et al 1998 (9831625)
Alpha-2-macroglobulin	Apolipoprotein E	Reconstituted complex	Isolation of alpha-2-macroglobulin by gel filtration chromatography, electroelution, or immunoprecipitation resulted in co-isolation of apolipoprotein E	Krimbou et al 1998 (9831625)
Transthyretin	Alpha-2-macroglobulin	Two-hybrid	Yeast two-hybrid study. Directionality predicted by naïve Bayesian classifier (alpha-2-microglobulin upstream of transthyretin)	Vinayagam et al 2011 (21900206)
Alpha-2-macroglobulin	Apolipoprotein E	Two-hybrid	Yeast two-hybrid study	Soler-Lopez et al 2011 (21163940)
Transthyretin	Clusterin	Reconstituted complex	Interaction between clusterin and transthyretin (TTR) proteins including wild-type TTR and TTR variants V30M and L55P was assessed using a glutathione S-transferase pull-down assay. Clusterin was found to strongly interact with wild-type TTR and TTR variants V30M and L55P under acidic conditions. Clusterin was also found to inhibit the amyloid fibril formation of TTR variants	Lee et al 2009 (19664600)
Apolipoprotein E	Phospholipid transfer protein	Affinity capture – Western	Co-elution on chromatography of HepG2 (human hepatoma cell line) cell culture medium. When medium subjected to chromatography on an anti-apolipoprotein E affinity column, a portion of phospholipid transfer protein applied was bound and could be eluted together with apolipoprotein E at low pH	Siggins et al 2003 (12810820)
Complement C1r	Plasma protease C1 inhibitor	Co-fractionation	Interactions analysed by sucrose-density-gradient ultracentrifugation and sodium dodecyl sulphate/polyacrylamide-gel electrophoresis. The interaction of C1 inhibitor with dimeric C1r in the presence of EDTA resulted into two bimolecular complexes accounting for a disruption of C1r	Chesne et al 1982 (6282262)
Alpha crystallin A chain	Beta-crystallin B2	Two-hybrid	Mammalian two-hybrid study	Fu et al 2002 (11700327)

© 2020 Mongan D et al. *JAMA Psychiatry.*

Beta-crystallin B2	Alpha crystallin A chain	Two-hybrid	Mammalian two-hybrid study	Fu et al 2002 (11700327)
Beta-crystallin B2	Alpha crystallin A chain	Affinity capture – Western	Complex formation on Western blot	Fu et al 2002 (11700327)
Alpha crystallin A chain	Beta-crystallin B2	Two-hybrid	Mammalian two-hybrid study	Fu et al 2003 (12601044)

BIOGRID database: https://thebiogrid.org/

© 2020 Mongan D et al. *JAMA Psychiatry.*

eTable 8: Functional enrichment analysis of differentially expressed proteins (following false discovery rate correction) between CHR-T and CHR-NT in EU-GEI initial experiment: 6 KEGG pathways significantly enriched

**KEGG pathway**	**Count in gene set**	**Fisher's exact test *p* (corrected for false discovery rate)**
Complement and coagulation cascades	13 of 78	2.23E-21
Staphylococcus aureus infection	6 of 51	5.29E-09
Pertussis	4 of 74	6.38E-05
Cholesterol metabolism	3 of 48	0.00047
Systemic lupus erythematosus	3 of 94	0.0025
Prion diseases	2 of 33	0.0058

CHR-T: clinical high-risk participants who transitioned to first episode psychosis; CHR-NT: clinical high-risk participants who did not transition; EU-GEI: European Network of National Schizophrenia Networks Studying Gene-Environment Interactions; KEGG: Kyoto Encyclopedia of Genes and Genomes
https://www.genome.jp/kegg/

© 2020 Mongan D et al. *JAMA Psychiatry.*

eTable 9: Results of enzyme-linked immunosorbent assays in CHR-T and CHR-NT participants in EU-GEI initial experiment

	CHR-T mean (SD)	CHR-NT mean (SD)	t	*p*	Corrected *p* (5% FDR)
**Alpha-2-macroglobulin (μg/ml)**	1173.1 (459.1)	11501.7 (711.1)	3.2202	0.002	0.018
**Apolipoprotein E (ng/ml)[a]**	163751.3 (47433.8)	151740.6 (50903.5)	-1.3449	0.182	0.387
**Complement C1q (ng/ml)**	82811.7 (35347.1)	80204.6 (33535.8)	-0.4153	0.679	0.724
**Complement C1r (μg/ml)**	65008.9 (27901.6)	52803.9 (18481.6)	-2.7099	0.008	0.036
**Complement C4 binding protein (ng/ml)**	495765.9 (222274.7)	482208.2 (192019.3)	-0.3538	0.724	0.724
**Complement C8 (ng/ml)**	58233.5 (22885.2)	55706.0 (21938.2)	-0.6196	0.537	0.690
**Complement factor H (ng/ml)[b]**	701713.1 (207717.4)	663292.1 (199397.1)	-1.0112	0.315	0.473
**Immunoglobulin M (ng/ml)**	1752941.0 (770671.5)	1941142.0 (936493.0)	1.2460	0.215	0.387
**Plasminogen (ng/ml)**	206880.2 (73232.4)	176929.9 (62709.8)	-2.3786	0.020	0.060

Data available for 48 CHR-T and 84 CHR-NT, except for [a]Apolipoprotein E (46 CHR-T, 84 CHR-NT) and [b]Complement factor H (45 CHR-T, 82 CHR-NT).

Means (and standard deviations) are presented and are compared using 2-sided t-test with unequal variances.

CHR-T: clinical high-risk participants who transitioned to first episode psychosis; CHR-NT: clinical high-risk participants who did not transition; SD: standard deviation; FDR: false discovery rate; EU-GEI: European Network of National Schizophrenia Networks Studying Gene-Environment Interactions

© 2020 Mongan D et al. *JAMA Psychiatry.*

eTable 10: Correlations between 5 proteins assessed by ELISA and by mass spectrometry in EU-GEI initial experiment

Protein	Spearman's rho	*p*	Corrected *p* (5% FDR)
Alpha-2-macroglobulin	0.25	0.0049	0.0080
Plasminogen	0.47	<0.0001	<0.0003
Complement component 1r	0.11	0.2089	0.2611
Complement factor H	0.07	0.4160	0.4160
Apolipoprotein E	0.41	<0.0001	<0.0003

ELISA: enzyme immunosorbent immunoassay; EU-GEI: European Network of National Schizophrenia Networks Studying Gene-Environment Interactions; FDR: false discovery rate

© 2020 Mongan D et al. *JAMA Psychiatry.*

eTable 11: Comparison of characteristics for participants included in replication experiment (N=135) from total EU-GEI clinical high risk cohort (N=344)

	Missing data, n (%)	Included, N = 135<br><br>(49 CHR-T, 86 CHR-NT)	Not included, N = 209<br><br>(CHR-T 16, CHR-NT 193)	t/ χ^2	*p*	Corrected *p* (5% FDR)
**Baseline age in years, mean (SD)**	0	22.2 (4.8)	22.5 (5.0)	-0.570	0.569	0.751
**Sex, n (%)**	0	82 male (60.7%)<br><br>53 female (39.3%)	103 male (49.3%)<br><br>106 female (50.7%)	4.322	0.037	0.320
**Baseline BMI in kg/m^2, mean (SD)**	50 (14.5%)	23.7 (4.1)	24.2 (5.8)	-0.936	0.350	0.700
**Baseline years in education, mean (SD)**	38 (11.0%)	14.3 (2.9)	14.5 (3.2)	-0.574	0.566	0.751
**Ethnicity, n (%)**	0	93 white (68.9%)<br><br>14 black (10.4%)<br><br>28 other (20.7%)	154 white (73.7%)<br><br>20 black (9.6%)<br><br>35 other (16.7%)	1.030	0.597	0.751
**Ever used cannabis, n (%)**	10 (2.9%)	101 yes (74.8%)<br><br>31 no (23.0%)<br><br>3 not known (2.2%)	143 yes (68.4%)<br><br>59 no (28.2%)<br><br>7 not known (3.3%)	1.328	0.249	0.619
**Baseline cannabis use, n (%)**	95 (27.6%)	43 yes (31.9%)<br><br>60 no (44.4%)<br><br>32 not known (23.7%)	45 yes (21.5%)<br><br>101 no (48.3%)<br><br>63 not known (30.1%)	3.155	0.076	0.320
**Baseline tobacco use, n (%)**	38 (11.0%)	72 yes (53.3%)<br><br>51 no (37.8%)<br><br>12 not known (8.9%)	89 yes (42.6%)<br><br>94 no (45.0%)<br><br>26 not known (12.4%)	2.893	0.089	0.320
**Baseline alcohol use, n (%)**	12 (3.5%)	36 yes (26.7%)<br><br>97 no (71.9%)<br><br>2 not known (1.5%)	137 yes (65.6%)<br><br>62 no (29.7%)<br><br>10 not known (4.8%)	0.640	0.424	0.751
**Baseline medication use, n (%)**	87 (25.3%)	51 yes (37.8%)<br>Antidepressant 34<br>Antipsychotic 15<br>Hypnotic 4<br>Other 12<br><br>50 no (37.0%)	77 yes (36.8%)<br>Antidepressant 57<br>Antipsychotic 16<br>Hypnotic 19<br>Other 24<br><br>79 no (37.8%)	0.032	0.859	0.960

© 2020 Mongan D et al. *JAMA Psychiatry.*

		34 not known (25.2%)	53 not known (25.4%)			
**Baseline GAF symptoms score, mean (SD)**	27 (7.8%)	54.4 (10.2)	55.6 (10.0)	-1.103	0.271	0.619
**Baseline GAF disability score, mean (SD)**	12 (3.5%)	55.5 (13.7)	55.4 (11.3)	0.006	0.996	0.996
**Baseline SANS total composite score, mean (SD)**	45 (13.1%)	17.1 (12.7)	14.6 (10.8)	1.784	0.075	0.320
**Baseline SANS total global score, mean (SD)**	29 (8.4%)	5.6 (3.8)	5.4 (3.5)	0.510	0.610	0.751
**Baseline BPRS total score, mean (SD)**	25 (7.3%)	44.9 (11.2)	42.9 (9.6)	1.650	0.100	0.320
**Baseline MADRS total score, mean (SD)**	16 (4.7%)	18.8 (9.5)	18.9 (8.9)	-0.126	0.900	0.960

[a] Poor functioning: GAF disability score ≤60; good functioning: GAF disability score >60

Tobacco use was defined as daily use for at least 1 month over the previous 12 months. Alcohol use was defined as at least 12 or more alcoholic beverages over the previous 12 months. Missing data excluded in hypothesis tests.

EU-GEI: European Network of National Schizophrenia Networks Studying Gene-Environment Interactions; CHR-T: clinical high risk, transitioned to psychosis; CHR-NT: clinical high risk, did not transition to psychosis; EU-GEI: European Network of National Schizophrenia Networks Studying Gene-Environment Interactions; CHR-T: clinical high risk, transitioned to psychosis; CHR-NT: clinical high risk, did not transition to psychosis; FDR: false discovery rate; BMI: body mass index; GAF: General Assessment of Functioning; SANS: Scale for the Assessment of Negative Symptoms; BPRS: Brief Psychiatric Rating Scale; MADRS: Montgomery Asberg Depression Rating Scale; BMI: body mass index; GAF: General Assessment of Functioning; SANS: Scale for the Assessment of Negative Symptoms; BPRS: Brief Psychiatric Rating Scale; MADRS: Montgomery Asberg Depression Rating Scale

© 2020 Mongan D et al. *JAMA Psychiatry.*

eTable 12: Sample characteristics for CHR-T and CHR-NT groups in the replication experiment

	Missing data, n (%)	CHR-T N = 49	CHR-NT N = 86	t/ χ^2	*p*	Corrected *p* (5% FDR)
**Baseline age in years, mean (SD)**	0	22.0 (4.7)	22.3 (4.9)	t = -0.339	0.735	0.776
**Sex, n (%)**	0	26 male (53%)<br><br>23 female (47%)	56 male (65%)<br><br>30 female (35%)	χ^2 = 1.902	0.168	0.290
**Baseline BMI in kg/m^2, mean (SD)**	21 (15.6%)	24.5 (4.5)	23.2 (3.8)	t = 1.722	0.088	0.186
**Baseline years in education, mean (SD)**	12 (8.8%)	14.0 (3.1)	14.3 (2.6)	t = -0.573	0.568	0.762
**Ethnicity, n (%)**	0	31 white (63.3%)<br><br>10 black (20.4%)<br><br>8 other (16.3%)	62 white (72.1%)<br><br>4 black (4.7%)<br><br>20 other (23.3%)	χ^2 = 8.549	0.014	0.057
**Ever used cannabis, n (%)**	3 (2.2%)	35 yes (71.4%)<br><br>12 no (24.5%)<br><br>2 not known (4.1%)	66 yes (76.7%)<br><br>19 no (22.1%)<br><br>1 not known (1.2%)	χ^2 = 0.170	0.680	0.762
**Baseline cannabis use, n (%)**	32 (23.7%)	14 yes (28.6%)<br><br>22 no (44.9%)<br><br>13 not known (26.5%)	29 yes (33.7%)<br><br>38 no (44.2%)<br><br>19 not known (22.1%)	χ^2 = 0.186	0.666	0.762
**Baseline tobacco use, n (%)**	12 (8.9%)	19 yes (38.8%)<br><br>23 no (46.9%)<br><br>7 not known (14.3%)	53 yes (61.6%)<br><br>28 no (32.6%)<br><br>5 not known (5.8%)	χ^2 = 4.647	0.031	0.074
**Baseline alcohol use, n (%)**	2 (1.5%)	34 yes (69.4%)<br><br>14 no (28.6%)<br><br>1 not known (2.0%)	63 yes (73.3%)<br><br>22 no (25.6%)<br><br>1 not known (1.2%)	χ^2 = 0.168	0.682	0.762
**Baseline medication use, n (%)**	34 (25.2%)	20 yes (40.8%)<br>Antidepressant 13<br>Antipsychotic 10<br>Hypnotic 2<br>Other 4<br><br>20 no (40.8%)<br><br>9 not known (18.4%)	31 yes (36.0%)<br>Antidepressant 21<br>Antipsychotic 5<br>Hypnotic 2<br>Other 8<br><br>30 no (34.9%)<br><br>25 not known (29.1%)	χ^2 = 0.006	0.936	0.936

© 2020 Mongan D et al. *JAMA Psychiatry.*

**Baseline GAF symptoms score, mean (SD)**	4 (3.0%)	53.0 (10.1)	55.1 (10.2)	t = -1.089	0.278	0.440
**Baseline GAF disability score, mean (SD)**	4 (3.0%)	53.0 (12.6)	56.8 (14.1)	t = -1.531	0.128	0.243
**Baseline SANS total composite score, mean (SD)**	15 (11.1%)	20.9 (14.1)	14.9 (11.4)	t = 2.389	0.019	0.060
**Baseline SANS total global score, mean (SD)**	10 (7.4%)	6.6 (4.1)	5.0 (3.5)	t = 2.252	0.026	0.071
**Baseline BPRS total score, mean (SD)**	10 (7.4%)	48.1 (11.2)	43.1 (10.8)	t = 2.456	0.015	0.057
**Baseline MADRS total score, mean (SD)**	6 (4.4%)	19.9 (10.2)	18.1 (9.1)	t = 1.004	0.317	0.463
**2 year GAF symptoms score, mean (SD)** [a]	49 (36.3%)	43.6 (14.1)	63.5 (10.6)	t = -7.281	<0.001	<0.007
**2 year GAF disability score, mean (SD)** [b]	44 (32.6%)	45.3 (9.5)	65.1 (13.9)	t = -7.969	<0.001	<0.007
**2 year GAF disability score, dichotomous outcome** [c]	44 (32.6%)	28 poor functioning (57.1%)<br><br>2 good functioning (4.1%)<br><br>19 not known (38.8%)	23 poor functioning (26.7%)<br><br>38 good functioning (44.2%)<br><br>25 not known (29.1%)	χ^2 = 25.261	<0.001	<0.007

[a] Data available for 86 of 135 participants (CHR-NT n=27, CHR-T n=59)

[b] Data available for 91 of 135 participants (CHR-NT n=30, CHR-T n=61)

[c] Poor functioning: GAF disability score ≤60; good functioning: GAF disability score >60

Tobacco use was defined as daily use for at least 1 month over the previous 12 months.
Alcohol use was defined as at least 12 or more alcoholic beverages over the previous 12 months.

Missing data excluded in hypothesis tests.

EU-GEI: European Network of National Schizophrenia Networks Studying Gene-Environment Interactions; CHR-T: clinical high risk, transitioned to psychosis; CHR-NT: clinical high risk, did not transition to psychosis; FDR: false discovery rate; BMI: body mass index; GAF: General Assessment of Functioning; SANS: Scale for the Assessment of Negative Symptoms; BPRS: Brief Psychiatric Rating Scale; MADRS: Montgomery Asberg Depression Rating Scale

© 2020 Mongan D et al. *JAMA Psychiatry.*

eTable 13: Results of ANCOVA (adjusted for age, sex, BMI, years in education, tobacco use and ethnicity) and fold changes (CHR-T vs. CHR-NT) for proteins identified in EU-GEI baseline plasma samples in the replication experiment

Uniprot no.	Protein name	F	*p*	Corrected *p* (5% FDR)	Direction of effect	Ratio of means (T vs. NT)
P01023	Alpha-2-macroglobulin	264.41	9.81E-33	1.17E-30	↓	0.24
P01871	Immunoglobulin heavy constant mu	109.92	7.11E-19	4.23E-17	↓	0.33
P00747	Plasminogen	78.47	6.43E-15	2.55E-13	↑	1.30
P10909	Clusterin	73.41	3.17E-14	9.44E-13	↑	1.36
P01011	Alpha-1-antichymotrypsin	68.88	1.38E-13	3.28E-12	↑	1.38
G3XAM2	Complement factor I	65.48	4.24E-13	8.4E-12	↑	1.27
P0DOY3	Immunoglobulin lambda constant 3	58.29	4.9E-12	8.33E-11	↓	0.56
P01860	Immunoglobulin heavy constant gamma 3	56.09	1.06E-11	1.42E-10	↓	0.52
P08603	Complement factor H (H factor 1)	56.05	1.08E-11	1.42E-10	↑	1.18
P13671	Complement component C6	55.01	1.55E-11	1.7E-10	↑	1.39
P04004	Vitronectin	54.97	1.57E-11	1.7E-10	↑	1.33
P22792	Carboxypeptidase N subunit 2	52.23	4.18E-11	4.15E-10	↑	1.29
P07360	Complement component C8 gamma chain	49.11	1.3E-10	1.19E-09	↑	1.28
P08571	Monocyte differentiation antigen CD14	47.31	2.52E-10	2.14E-09	↑	1.39
P01031	Complement C5	44.69	6.71E-10	5.32E-09	↑	1.19
B7ZKJ8	ITIH4 protein	43.28	1.14E-09	8.51E-09	↑	1.21
P07359	Platelet glycoprotein Ib alpha chain	35.10	2.79E-08	1.96E-07	↑	1.40
P15169	Carboxypeptidase N catalytic chain (CPN)	33.90	4.53E-08	2.99E-07	↑	1.33
P36955	Pigment epithelium-derived factor	32.23	8.97E-08	5.62E-07	↑	1.22
P07357	Complement component C8 alpha chain	31.63	1.15E-07	6.82E-07	↑	1.25
P27169	Serum paraoxonase/arylesterase 1	30.66	1.71E-07	9.69E-07	↑	1.30
Q96IY4	Carboxypeptidase B2	29.85	2.39E-07	1.29E-06	↑	1.41
Q16610	Extracellular matrix protein 1	26.68	9.12E-07	4.72E-06	↑	1.38
P04003	C4b-binding protein alpha chain	26.26	1.09E-06	5.41E-06	↓	0.77
P12259	Coagulation factor V	24.43	2.4E-06	1.14E-05	↑	1.30
P06727	Apolipoprotein A-IV	23.96	2.95E-06	1.35E-05	↑	1.16
P80108	Phosphatidylinositol-glycan-specific phospholipase D (PI-G PLD)	22.81	4.87E-06	2.15E-05	↑	1.27

© 2020 Mongan D et al. *JAMA Psychiatry.*

P05155	Plasma protease C1 inhibitor	22.67	5.19E-06	2.00E-04	↑	1.23
P19827	Inter-alpha-trypsin inhibitor heavy chain H1	20.64	1.28E-05	0.0001	↑	1.15
P0C0L4	Complement C4-A (Acidic complement C4)	19.53	2.11E-05	0.0001	↓	0.57
B4E1Z4	cDNA FLJ55673	18.98	2.71E-05	0.0001	↑	1.13
P51884	Lumican	18.90	2.81E-05	0.0001	↑	1.26
P00736	Complement C1r subcomponent	18.64	3.17E-05	0.0001	↑	1.13
O43866	CD5 antigen-like	18.47	3.42E-05	0.0001	↓	0.65
P02765	Alpha-2-HS-glycoprotein	17.11	0.0001	0.0003	↑	1.16
P06396	Gelsolin	16.15	0.0001	0.0003	↑	1.15
Q04756	Hepatocyte growth factor activator	16.22	0.0001	0.0003	↑	1.19
P19823	Inter-alpha-trypsin inhibitor heavy chain H2	15.64	0.0001	0.0003	↑	1.11
O00391	Sulfhydryl oxidase 1	14.82	0.0002	0.0006	↑	1.18
P02748	Complement component C9	13.25	0.0004	0.0012	↑	1.19
P00738	Haptoglobin	11.99	0.0007	0.0020	↓	0.49
P02790	Hemopexin	11.97	0.0007	0.0020	↑	1.10
P08697	Alpha-2-antiplasmin	11.36	0.0010	0.0027	↑	1.10
P23142	Fibulin-1	11.37	0.0010	0.0027	↑	1.37
P05452	Tetranectin	11.15	0.0011	0.0029	↑	1.15
P06681	Complement C2	10.96	0.0012	0.0031	↑	1.18
P02751	Fibronectin	10.62	0.0014	0.0035	↑	1.39
P02649	Apolipoprotein E	10.26	0.0017	0.0041	↑	1.21
P07358	Complement component C8 beta chain	10.24	0.0017	0.0041	↑	1.18
O75882	Attractin	10.07	0.0019	0.0044	↑	1.19
P01834	Immunoglobulin kappa constant	10.09	0.0019	0.0044	↓	0.74
P00734	Prothrombin	9.98	0.0020	0.0046	↑	1.09
P17936	Insulin-like growth factor-binding protein 3	9.46	0.0026	0.0058	↑	1.30
P00746	Complement factor D	9.15	0.0030	0.0066	↑	1.23
P02675	Fibrinogen beta chain	8.48	0.0043	0.0093	↓	0.87
P00488	Coagulation factor XIII A chain	8.32	0.0046	0.0098	↑	1.26
P43652	Afamin	8.04	0.0053	0.0111	↑	1.10
P35858	Insulin-like growth factor-binding protein complex acid labile subunit (ALS)	7.33	0.0077	0.0158	↑	1.11
P05543	T4-binding globulin	7.30	0.0079	0.0159	↑	1.27
P10643	Complement component C7	7.09	0.0088	0.0175	↑	1.29
P02787	Serotransferrin	6.92	0.0096	0.0187	↓	0.83
P26927	Hepatocyte growth factor-like protein (Macrophage stimulatory protein)	6.80	0.0102	0.0193	↑	1.24

© 2020 Mongan D et al. *JAMA Psychiatry.*

P18428	Lipopolysaccharide-binding protein (LBP)	6.81	0.0102	0.0193	↑	1.27
P02760	Protein AMBP	6.63	0.0112	0.0208	↑	1.05
P00450	Ceruloplasmin	6.38	0.0128	0.0234	↓	0.93
P05154	Plasma serine protease inhibitor	6.25	0.0137	0.0247	↑	1.15
P02766	Transthyretin	6.14	0.0145	0.0258	↓	0.82
Q14520-2	Hyaluronan-binding protein 2	6.09	0.0149	0.0260	↑	1.20
P02679-2	Fibrinogen gamma chain	6.07	0.0151	0.0260	↓	0.90
P00740	Coagulation factor IX	5.92	0.0164	0.0279	↑	1.10
Q96KN2	Beta-Ala-His dipeptidase	5.82	0.0173	0.0290	↑	1.27
P01019	Angiotensinogen	5.42	0.0215	0.0355	↑	1.12
P04114	Apolipoprotein B-100	5.29	0.0231	0.0377	↑	1.07
P02647	Apolipoprotein A-I	5.03	0.0266	0.0428	↓	0.89
P05546	Heparin cofactor 2	4.81	0.0302	0.0479	↑	1.06
P01009	Alpha-1-antitrypsin	4.77	0.0307	0.0481	↓	0.79
P04275	von Willebrand factor	4.71	0.0319	0.0493	↑	1.37
P02743	Serum amyloid P-component (SAP)	4.68	0.0324	0.0494	↑	1.22
P01857	Immunoglobulin heavy constant gamma 1	4.63	0.0334	0.0503	↑	1.14
P01008	Antithrombin-III	4.27	0.0408	0.0607	↓	0.91
P25311	Zinc-alpha-2-glycoprotein	4.08	0.0456	0.0670	↑	1.01
P05160	Coagulation factor XIII B chain	3.40	0.0675	0.0980	↑	1.06
P22891	Vitamin K-dependent protein Z	3.13	0.0795	0.1140	↑	1.10
P02746	Complement C1q subcomponent subunit B	2.85	0.0936	0.1326	↑	1.17
P0C0L5	Complement C4-B (Basic complement C4)	2.72	0.1014	0.1420	↑	1.09
Q96PD5	N-acetylmuramoyl-L-alanine amidase	2.63	0.1075	0.1488	↑	1.05
P01876	Immunoglobulin heavy constant alpha 1	2.54	0.1135	0.1546	↓	0.72
P02763	Alpha-1-acid glycoprotein 1	2.51	0.1154	0.1546	↓	0.84
P01042	Kininogen-1	2.51	0.1156	0.1546	↑	1.03
K7ERI9	Apolipoprotein C-I	2.17	0.1433	0.1895	↓	0.84
Q06033	Inter-alpha-trypsin inhibitor heavy chain H3	2.07	0.1524	0.1993	↑	1.13
P02750	Leucine-rich alpha-2-glycoprotein	2.01	0.1589	0.2055	↑	1.08
D6RF35	Vitamin D-binding protein	1.89	0.1721	0.2202	↑	1.02
P00748	Coagulation factor XII	1.85	0.1762	0.2231	↓	0.93
P09871	Complement C1s subcomponent	1.70	0.1951	0.2444	↓	0.92
P07996	Thrombospondin-1	1.11	0.2932	0.3634	↑	1.10
Q9NZP8	Complement C1r subcomponent-like protein	1.06	0.3051	0.3743	↑	1.12
P02747	Complement C1q subcomponent subunit C	1.02	0.3148	0.3823	↑	1.05
P04278	Sex hormone-binding globulin	0.64	0.4244	0.5072	↑	1.17

© 2020 Mongan D et al. *JAMA Psychiatry.*

P29622	Kallistatin (Kallikrein inhibitor)	0.64	0.4262	0.5072	↑	1.02
P03952	Plasma kallikrein	0.58	0.4476	0.5274	↑	1.08
P43251	Biotinidase (Biotinase)	0.55	0.4607	0.5375	↓	0.92
P01024	Complement C3	0.45	0.5025	0.5775	↑	1.01
P04196	Histidine-rich glycoprotein	0.45	0.5047	0.5775	↑	1.02
P02656	Apolipoprotein C-III	0.43	0.5145	0.5831	↑	1.09
O75636	Ficolin-3	0.36	0.5475	0.6146	↑	1.10
P27918	Properdin	0.30	0.5819	0.6472	↑	1.05
P00742	Coagulation factor X	0.27	0.6070	0.6688	↑	1.06
P02749	Beta-2-glycoprotein 1	0.23	0.6357	0.6940	↑	1.01
P06276	Acylcholine acylhydrolase	0.20	0.6588	0.7127	↑	1.01
P08185	Corticosteroid-binding globulin	0.13	0.7170	0.7687	–	1.00
P02671	Fibrinogen alpha chain	0.08	0.7845	0.8335	↓	0.97
P03951	Coagulation factor XI	0.05	0.8228	0.8628	↑	1.12
P19652	Alpha-1-acid glycoprotein 2 (AGP 2)	0.05	0.8265	0.8628	↓	0.97
Q9UK55	Protein Z-dependent protease inhibitor	0.04	0.8393	0.8685	↑	1.01
P07225	Vitamin K-dependent protein S	0.03	0.8521	0.8741	–	1.00
Q5VY30	Retinol-binding protein	0.02	0.8787	0.8937	↓	0.97
P68871	Hemoglobin subunit beta	0.02	0.9022	0.9079	↑	1.06
P22352	Glutathione peroxidase 3 (GPx-3)	0.01	0.9079	0.9079	↑	1.03

CHR-T: clinical high-risk participants who transitioned to first episode psychosis; CHR-NT: clinical high-risk participants who did not transition; FDR: false discovery rate; EU-GEI: European Network of National Schizophrenia Networks Studying Gene-Environment Interactions

© 2020 Mongan D et al. *JAMA Psychiatry.*

eTable 14: Sample characteristics for ALSPAC subsample cases and controls

	**Cases**<br>**N = 55**	**Controls**<br>**N = 66**	**t/ χ^2**	***p***
**Sex, n (%)**	22 male (40.0%)<br><br>33 female (60.0%)	39 male (59.1%)<br><br>27 female (40.9%)	4.374	0.036
**BMI age 12 in kg/m^2, mean (SD)**	18.1 (2.8)	17.7 (2.5)	0.749	0.455
**Maternal social class, n (%)**	40 non-manual (72.7%)<br><br>7 manual (12.7%)<br><br>8 not known (14.6%)	44 non-manual (66.7%)<br><br>8 manual (12.1%)<br><br>14 not known (21.2%)	0.005	0.946

Cases: participants with no PEs age 12 and definite PEs age 18; Controls: participants with no PEs age 12 and no PEs age 18

PEs: psychotic experiences; BMI: body mass index

Missing data excluded in hypothesis tests.

Data for ethnicity are not presented in the table because of potential risk of statistical disclosure due to small cell counts. There were no significant differences between cases and controls for ethnicity (χ^2 = 0.729, p = 0.202).

ALSPAC: Avon Longitudinal Study of Parents and Children

© 2020 Mongan D et al. *JAMA Psychiatry.*

eTable 15: Results of ANCOVA (adjusted for sex, BMI and maternal social class) and fold changes (definite PEs at 18 vs. no PEs at 18) for proteins identified in ALSPAC age 12 plasma samples

Uniprot No.	Protein name	F	*p*	Corrected *p* (5%FDR)	Direction of effect	Ratio of means (PE vs no PE)
P04003	C4b-binding protein alpha chain	24.59	2.44E-06	0.0006	↓	0.77
P27169	Serum paraoxonase/arylesterase 1	17.78	4.94E-05	0.0065	↓	0.80
P01871	Immunoglobulin heavy constant mu	16.04	0.0001	0.0066	↓	0.78
P55103	Inhibin beta C chain	16.68	0.0001	0.0066	↑	1.31
P10909	Clusterin	12.71	0.0005	0.0265	↓	0.92
P01591	Immunoglobulin J chain	9.69	0.0023	0.1007	↓	0.70
P01860	Immunoglobulin heavy constant gamma 3	9.25	0.0029	0.1007	↓	0.80
P0DOY3	Immunoglobulin lambda constant 3	8.90	0.0035	0.1007	↓	0.81
P07225	Vitamin K-dependent protein S	8.83	0.0036	0.1007	↓	0.90
Q03591	Complement factor H-related protein 1	8.74	0.0038	0.1007	↓	0.79
P01023	Alpha-2-macroglobulin	7.92	0.0057	0.1373	↓	0.85
P01623	Immunoglobulin kappa variable 3-20	7.49	0.0072	0.1590	↓	0.81
P24593	Insulin-like growth factor-binding protein 5	7.34	0.0078	0.1590	↑	1.26
P01019	Angiotensinogen	6.74	0.0106	0.1943	↓	0.91
P26038	Moesin	6.68	0.0110	0.1943	↑	1.16
P04040	Catalase	6.53	0.0119	0.1971	↑	1.36
P12109	Collagen alpha-1 (VI) chain	6.36	0.0130	0.1988	↑	1.16
P08571	Monocyte differentiation antigen CD14	6.29	0.0135	0.1988	↓	0.91
B9A064	Immunoglobulin lambda-like polypeptide 5	6.03	0.0155	0.2107	↓	0.85
P09871	Complement C1s subcomponent	5.99	0.0159	0.2107	↓	0.93
P08697	Alpha-2-antiplasmin	5.50	0.0207	0.2495	↓	0.94
P15151	Poliovirus receptor	5.44	0.0214	0.2495	↑	1.16
P01717	Immunoglobulin lambda variable 3-25	5.34	0.0226	0.2495	↓	0.85
Q12884	Prolyl endopeptidase FAP	5.34	0.0226	0.2495	↑	1.24
P00746	Complement factor D	5.16	0.0249	0.2554	↑	1.12

© 2020 Mongan D et al. *JAMA Psychiatry.*

P01615	Immunoglobulin kappa variable 2D-28	5.10	0.0258	0.2554	↓	0.84
P02671	Fibrinogen alpha chain	5.05	0.0266	0.2554	↓	0.89
P23142	Fibulin-1	4.99	0.0275	0.2554	↑	1.36
P01834	Immunoglobulin kappa constant	4.95	0.0280	0.2554	↓	0.90
P24592	Insulin-like growth factor-binding protein 6	4.85	0.0297	0.2554	↑	1.15
P02679	Fibrinogen gamma chain	4.77	0.0310	0.2554	↓	0.89
P02675	Fibrinogen beta chain	4.76	0.0311	0.2554	↓	0.90
P03951	Coagulation factor XI	4.72	0.0318	0.2554	↓	0.91
P80748	Immunoglobulin lambda variable 3-21	4.63	0.0334	0.2603	↓	0.85
P55290	Cadherin-13	4.43	0.0374	0.2832	↓	0.88
P02652	Apolipoprotein A-II	4.37	0.0388	0.2856	↓	0.85
P07358	Complement component C8 beta chain	4.12	0.0446	0.3061	↑	1.08
D6RAR4	Hepatocyte growth factor activator	4.11	0.0450	0.3061	↓	0.89
P15144	Aminopeptidase N	4.07	0.0460	0.3061	↑	1.06
Q99878	Histone H2A type 1-J	4.06	0.0462	0.3061	↑	1.37
P08294	Extracellular superoxide dismutase [Cu-Zn]	3.91	0.0505	0.3264	↑	1.13
P02750	Leucine-rich alpha-2-glycoprotein	3.83	0.0528	0.3331	↓	0.92
P61626	Lysozyme C	3.61	0.0600	0.3686	↓	0.91
P12111	Collagen alpha-3	3.55	0.0621	0.3686	↑	1.09
P00915	Carbonic anhydrase 1	3.53	0.0626	0.3686	↑	1.24
P14151	L-selectin	3.35	0.0700	0.4033	↑	1.08
O43866	CD5 antigen-like	3.26	0.0737	0.4155	↓	0.86
P02654	Apolipoprotein C-I	3.14	0.0791	0.4220	↓	0.91
P01024	Complement C3	3.09	0.0814	0.4220	↓	0.94
P29622	Kallistatin	3.08	0.0820	0.4220	↑	1.11
P02647	Apolipoprotein A-I	3.07	0.0823	0.4220	↓	0.86
Q14624	Inter-alpha-trypsin inhibitor heavy chain H4	3.06	0.0828	0.4220	↓	0.91
O00187	Mannan-binding lectin serine protease 2	2.92	0.0901	0.4404	↑	1.07
P16403	Histone H1.2	2.91	0.0907	0.4404	↑	1.19
P06276	Cholinesterase	2.90	0.0914	0.4404	↑	1.03

© 2020 Mongan D et al. *JAMA Psychiatry.*

Q9NQ79	Cartilage acidic protein 1	2.84	0.0946	0.4435	↑	1.08
Q9H4A9	Dipeptidase 2	2.83	0.0954	0.4435	↓	0.84
H0Y755	Low affinity immunoglobulin gamma Fc region receptor III-A	2.74	0.1008	0.4606	↑	1.11
Q9UBQ6	Exostosin-like 2	2.50	0.1164	0.5142	↑	1.08
Q9UGM5	Fetuin-B	2.48	0.1178	0.5142	↓	0.94
O75636	Ficolin-3	2.47	0.1191	0.5142	↑	1.07
O95497	Pantetheinase	2.45	0.1203	0.5142	↑	1.15
P43251	Biotinidase	2.38	0.1254	0.5220	↑	1.06
Q08380	Galectin-3-binding protein	2.36	0.1276	0.5220	↑	1.08
P63261	Actin, cytoplasmic 2	2.34	0.1289	0.5220	↑	1.10
Q9Y5Y7	Lymphatic vessel endothelial hyaluronic acid receptor 1	2.33	0.1300	0.5220	↑	1.09
P00747	Plasminogen	2.26	0.1358	0.5361	↑	1.07
P22792	Carboxypeptidase N subunit 2	2.21	0.1395	0.5361	↓	0.95
P01033	Metalloproteinase inhibitor 1	2.21	0.1396	0.5361	↓	0.87
Q9ULI3	Protein HEG homolog 1	2.11	0.1488	0.5633	↑	1.13
Q96PD5	N-acetylmuramoyl-L-alanine amidase	2.06	0.1539	0.5638	↑	1.09
P05019	Insulin-like growth factor I	2.06	0.1540	0.5638	↑	1.24
P35858	Insulin-like growth factor-binding protein complex acid labile subunit	2.05	0.1553	0.5638	↑	1.10
P02753	Retinol-binding protein 4	2.01	0.1592	0.5701	↑	1.13
P02452	Collagen alpha-1 (I) chain	1.90	0.1706	0.5923	↑	1.18
P39060	Collagen alpha-1 (XVIII) chain	1.90	0.1707	0.5923	↑	1.06
P02747	Complement C1q subcomponent subunit C	1.89	0.1721	0.5923	↓	0.96
P04004	Vitronectin	1.78	0.1853	0.6295	↑	1.06
P07942	Laminin subunit beta-1	1.75	0.1887	0.6330	↑	1.01
P02745	Complement C1q subcomponent subunit A	1.70	0.1944	0.6440	↓	0.93
P07359	Platelet glycoprotein Ib alpha chain	1.65	0.2010	0.6535	↑	1.03
P07737	Profilin-1	1.65	0.2022	0.6535	↑	1.17
P00751	Complement factor B	1.58	0.2116	0.6756	↓	0.97

© 2020 Mongan D et al. *JAMA Psychiatry.*

H0YD13	CD44 antigen	1.53	0.2185	0.6818	↑	1.09
P48740	Mannan-binding lectin serine protease 1	1.53	0.2192	0.6818	↓	0.91
P07357	Complement component C8 alpha chain	1.50	0.2227	0.6818	↑	1.05
P08603	Complement factor H	1.48	0.2263	0.6818	↑	1.04
Q16706	Alpha-mannosidase 2	1.48	0.2264	0.6818	↓	0.92
P20851	C4b-binding protein beta chain	1.46	0.2290	0.6819	↓	0.89
Q12913	Receptor-type tyrosine-protein phosphatase eta	1.43	0.2341	0.6870	↓	0.93
Q15582	Transforming growth factor-beta-induced protein ig-h3	1.42	0.2359	0.6870	↑	1.04
Q6UXB8	Peptidase inhibitor 16	1.39	0.2407	0.6933	↑	1.09
P69905	Hemoglobin subunit alpha	1.31	0.2553	0.7051	↑	3.63
F5GZZ9	Scavenger receptor cysteine-rich type 1 protein M130	1.29	0.2586	0.7051	↑	1.20
P13591	Neural cell adhesion molecule 1	1.27	0.2619	0.7051	↑	1.03
Q15113	Procollagen C-endopeptidase enhancer 1	1.26	0.2633	0.7051	↑	1.07
P26927	Hepatocyte growth factor-like protein	1.24	0.2671	0.7051	↑	1.03
Q12841	Follistatin-related protein 1	1.24	0.2675	0.7051	↑	1.04
Q15848	Adiponectin	1.24	0.2687	0.7051	↓	0.95
P27487	Dipeptidyl peptidase 4	1.23	0.2706	0.7051	↑	1.06
Q86U17	Serpin A11	1.22	0.2711	0.7051	↑	1.11
Q9UNW1	Multiple inositol polyphosphate phosphatase 1	1.22	0.2714	0.7051	↓	0.93
P43652	Afamin	1.20	0.2758	0.7096	↑	1.07
P51884	Lumican	1.18	0.2798	0.7130	↑	1.04
P13671	Complement component C6	1.15	0.2868	0.7195	↓	0.99
P04278	Sex hormone-binding globulin	1.14	0.2883	0.7195	↓	0.89
P03950	Angiogenin	1.13	0.2905	0.7195	↓	0.96
E9PBC5	Plasma kallikrein	1.10	0.2973	0.7295	↓	0.96
P04275	von Willebrand factor	1.07	0.3030	0.7309	↓	0.93
P02748	Complement component C9	1.07	0.3034	0.7309	↓	0.97
P08185	Corticosteroid-binding globulin	1.02	0.3139	0.7494	↑	1.02
P14625	Endoplasmin	1.00	0.3189	0.7545	↑	1.05
O75882	Attractin	0.97	0.3256	0.7636	↑	1.02

© 2020 Mongan D et al. *JAMA Psychiatry.*

P68871	Hemoglobin subunit beta	0.96	0.3300	0.7671	↑	2.75
P11021	Endoplasmic reticulum chaperone BiP	0.94	0.3342	0.7701	↓	0.96
P02787	Serotransferrin	0.90	0.3459	0.7862	↓	0.97
P18206	Vinculin	0.89	0.3471	0.7862	↑	1.08
Q5T7F0	Neuropilin	0.87	0.3521	0.7907	↓	0.96
P07360	Complement component C8 gamma chain	0.83	0.3655	0.8139	↑	1.04
P55058	Phospholipid transfer protein	0.78	0.3783	0.8265	↑	1.05
P01765	Immunoglobulin heavy variable 3-23	0.77	0.3806	0.8265	↓	0.89
P13727	Bone marrow proteoglycan	0.74	0.3900	0.8265	↑	1.09
P12259	Coagulation factor V	0.73	0.3962	0.8265	↓	0.97
P00742	Coagulation factor X	0.72	0.3970	0.8265	↑	1.03
P18065	Insulin-like growth factor-binding protein 2	0.70	0.4043	0.8265	↓	0.92
P54289	Voltage-dependent calcium channel subunit alpha-2/delta-1	0.70	0.4060	0.8265	↑	1.03
P13473	Lysosome-associated membrane glycoprotein 2	0.70	0.4061	0.8265	↓	0.92
P32119	Peroxiredoxin-2	0.69	0.4084	0.8265	↑	1.02
P04196	Histidine-rich glycoprotein	0.69	0.4087	0.8265	↑	1.05
P02649	Apolipoprotein E	0.69	0.4092	0.8265	↓	0.96
P02749	Beta-2-glycoprotein 1	0.68	0.4107	0.8265	↓	0.98
P02655	Apolipoprotein C-II	0.68	0.4117	0.8265	↑	1.06
P00734	Prothrombin	0.65	0.4218	0.8404	↓	0.97
Q9UNN8	Endothelial protein C receptor	0.64	0.4267	0.8438	↑	1.10
P02743	Serum amyloid P-component	0.62	0.4319	0.8478	↓	0.98
P08709	Coagulation factor VII	0.59	0.4429	0.8591	↑	1.02
P02746	Complement C1q subcomponent subunit B	0.59	0.4448	0.8591	↑	1.05
P04180	Phosphatidylcholine-sterol acyltransferase	0.58	0.4474	0.8591	↓	0.95
P24821	Tenascin	0.55	0.4606	0.8756	↑	1.05
P01042	Kininogen-1	0.54	0.4626	0.8756	↓	0.98
P23470	Receptor-type tyrosine-protein phosphatase gamma	0.53	0.4680	0.8791	↓	1.00
P07195	L-lactate dehydrogenase B chain	0.52	0.4712	0.8791	↓	0.95

© 2020 Mongan D et al. *JAMA Psychiatry.*

Q13822	Ectonucleotide pyrophosphatase/ phosphodiesterase family member 2	0.51	0.4744	0.8791	↑	1.02
P49908	Selenoprotein P	0.50	0.4810	0.8852	↓	1.00
P04114	Apolipoprotein B-100	0.49	0.4845	0.8855	↓	0.99
P02766	Transthyretin	0.47	0.4941	0.8909	↓	0.95
P27918	Properdin	0.47	0.4942	0.8909	↑	1.05
H0Y897	Target of Nesh-SH3	0.44	0.5101	0.9024	↑	1.06
P01344	Insulin-like growth factor II	0.44	0.5104	0.9024	↓	0.97
P01031	Complement C5	0.42	0.5163	0.9024	↑	1.01
P02776	Platelet factor 4	0.41	0.5214	0.9024	↓	0.94
P06727	Apolipoprotein A-IV	0.41	0.5222	0.9024	↑	1.01
P05155	Plasma protease C1 inhibitor	0.41	0.5226	0.9024	↓	0.96
P15169	Carboxypeptidase N catalytic chain	0.41	0.5251	0.9024	↓	0.97
Q9BXR6	Complement factor H-related protein 5	0.39	0.5319	0.9024	↓	1.00
P02656	Apolipoprotein C-III	0.39	0.5337	0.9024	↓	0.95
Q16270	Insulin-like growth factor-binding protein 7	0.39	0.5361	0.9024	↓	0.96
O95445	Apolipoprotein M	0.37	0.5426	0.9024	↓	0.98
P00488	Coagulation factor XIII A chain	0.37	0.5441	0.9024	↑	1.01
Q76LX8	A disintegrin and metalloproteinase with thrombospondin motifs 13	0.36	0.5492	0.9024	↓	0.97
Q8IUL8	Cartilage intermediate layer protein 2	0.35	0.5553	0.9024	↑	1.07
P61769	Beta-2-microglobulin	0.34	0.5628	0.9024	↑	1.05
Q99983	Osteomodulin	0.33	0.5661	0.9024	↑	1.04
P54802	Alpha-N-acetylglucosaminidase	0.32	0.5723	0.9024	↑	1.11
P11226	Mannose-binding protein C	0.32	0.5724	0.9024	↓	0.98
P05543	Thyroxine-binding globulin	0.32	0.5731	0.9024	↓	0.99
P10721	Mast/stem cell growth factor receptor Kit	0.32	0.5743	0.9024	↑	1.01
Q14520	Hyaluronan-binding protein 2	0.31	0.5759	0.9024	↓	0.98
P01861	Immunoglobulin heavy constant gamma 4	0.31	0.5783	0.9024	↓	0.82
P00748	Coagulation factor XII	0.31	0.5789	0.9024	↓	0.98
P09172	Dopamine beta-hydroxylase	0.29	0.5883	0.9081	↓	0.97

© 2020 Mongan D et al. *JAMA Psychiatry.*

Q16610	Extracellular matrix protein 1	0.29	0.5894	0.9081	↓	0.98
P36980	Complement factor H-related protein 2	0.28	0.5952	0.9084	↓	0.91
Q9UHG3	Prenylcysteine oxidase 1	0.28	0.5982	0.9084	↑	1.02
P01008	Antithrombin-III	0.28	0.5999	0.9084	↓	0.98
P01009	Alpha-1-antitrypsin	0.24	0.6231	0.9357	↓	0.99
P33151	Cadherin-5	0.24	0.6250	0.9357	↓	0.96
P17936	Insulin-like growth factor-binding protein 3	0.23	0.6329	0.9416	↑	1.06
P01876	Immunoglobulin heavy constant alpha 1	0.23	0.6360	0.9416	↓	0.96
P22352	Glutathione peroxidase 3	0.22	0.6413	0.9439	↓	1.00
P01034	Cystatin-C	0.21	0.6447	0.9439	↑	1.03
P28827	Receptor-type tyrosine-protein phosphatase mu	0.20	0.6555	0.9504	↓	0.98
P36955	Pigment epithelium-derived factor	0.20	0.6563	0.9504	↓	1.00
P59666	Neutrophil defensin 3	0.19	0.6605	0.9513	↓	0.99
P18428	Lipopolysaccharide-binding protein	0.18	0.6698	0.9594	↑	1.05
P43121	Cell surface glycoprotein MUC18	0.17	0.6784	0.9665	↓	0.98
P19652	Alpha-1-acid glycoprotein 2	0.15	0.7034	0.9799	↑	1.13
P04075	Fructose-bisphosphate aldolase A	0.14	0.7057	0.9799	↓	0.97
Q6EMK4	Vasorin	0.14	0.7064	0.9799	↓	0.99
Q14515	SPARC-like protein 1	0.14	0.7085	0.9799	↑	1.05
P05109	Protein S100-A8	0.14	0.7099	0.9799	↑	1.01
P05546	Heparin cofactor 2	0.14	0.7100	0.9799	↑	1.03
P49747	Cartilage oligomeric matrix protein	0.13	0.7175	0.9812	↓	1.00
Q9NPY3	Complement component C1q receptor	0.13	0.7183	0.9812	↑	1.01
Q9Y4L1	Hypoxia up-regulated protein 1	0.12	0.7318	0.9927	↑	1.02
P02774	Vitamin D-binding protein	0.11	0.7460	0.9927	↓	0.97
P00736	Complement C1r subcomponent	0.10	0.7467	0.9927	↓	0.99
P19827	Inter-alpha-trypsin inhibitor heavy chain H1	0.10	0.7490	0.9927	↓	0.98
P06702	Protein S100-A9	0.10	0.7506	0.9927	↓	1.00
O00391	Sulfhydryl oxidase 1	0.10	0.7547	0.9927	↑	1.01
Q92820	Gamma-glutamyl hydrolase	0.09	0.7590	0.9927	↑	1.03

© 2020 Mongan D et al. *JAMA Psychiatry.*

P80108	Phosphatidylinositol-glycan-specific phospholipase D	0.09	0.7649	0.9927	↑	1.01
Q9NPH3	Interleukin-1 receptor accessory protein	0.09	0.7649	0.9927	↑	1.06
P01011	Alpha-1-antichymotrypsin	0.08	0.7712	0.9927	↓	0.98
P35542	Serum amyloid A-4 protein	0.08	0.7743	0.9927	↑	1.03
P02763	Alpha-1-acid glycoprotein 1	0.08	0.7752	0.9927	↓	0.90
P20742	Pregnancy zone protein	0.08	0.7787	0.9927	↑	1.05
P02775	Platelet basic protein	0.08	0.7815	0.9927	↓	0.99
O95479	GDH/6PGL endoplasmic bifunctional protein	0.08	0.7835	0.9927	↓	0.99
P05362	Intercellular adhesion molecule 1	0.07	0.7875	0.9927	↓	0.95
O14791	Apolipoprotein L1	0.07	0.7904	0.9927	↑	1.06
P33908	Mannosyl-oligosaccharide 1,2-alpha-mannosidase IA	0.07	0.7947	0.9934	↓	0.98
P22891	Vitamin K-dependent protein Z	0.06	0.8010	0.9934	↓	0.91
Q13740	CD166 antigen	0.06	0.8022	0.9934	↓	0.97
P22105	Tenascin-X	0.05	0.8160	0.9976	↓	1.00
P05156	Complement factor I	0.05	0.8205	0.9976	↑	1.01
Q96KN2	Beta-Ala-His dipeptidase	0.05	0.8245	0.9976	↑	1.03
P10643	Complement component C7	0.04	0.8343	0.9976	↓	1.00
O00533	Neural cell adhesion molecule L1-like protein	0.04	0.8359	0.9976	↓	0.97
Q9BWP8	Collectin-11	0.04	0.8409	0.9976	↓	0.98
Q99969	Retinoic acid receptor responder protein 2	0.04	0.8425	0.9976	↓	0.99
P04070	Vitamin K-dependent protein C	0.04	0.8443	0.9976	↑	1.02
P04066	Tissue alpha-L-fucosidase	0.04	0.8513	0.9976	↓	0.94
P55056	Apolipoprotein C-IV	0.03	0.8585	0.9976	↑	1.12
P0C0L5	Complement C4-B	0.03	0.8646	0.9976	↓	0.99
P00450	Ceruloplasmin	0.03	0.8653	0.9976	↓	0.99
P02760	Protein AMBP	0.03	0.8721	0.9976	↓	1.00
P19320	Vascular cell adhesion protein 1	0.02	0.8840	0.9976	↓	0.98
P22692	Insulin-like growth factor-binding protein 4	0.02	0.8848	0.9976	↓	0.59
P02751	Fibronectin	0.02	0.8998	0.9976	↓	0.97
P08519	Apolipoprotein	0.01	0.9049	0.9976	↑	1.20

© 2020 Mongan D et al. *JAMA Psychiatry.*

P12830	Cadherin-1	0.01	0.9146	0.9976	↓	0.94
Q96IY4	Carboxypeptidase B2	0.01	0.9190	0.9976	↑	1.01
P17813	Endoglin	0.01	0.9194	0.9976	↓	0.97
P35443	Thrombospondin-4	0.01	0.9285	0.9976	↑	1.11
P98160	Basement membrane-specific heparan sulfate proteoglycan core protein	0.01	0.9306	0.9976	↓	0.98
P00738	Haptoglobin	0.01	0.9311	0.9976	↓	0.98
P19823	Inter-alpha-trypsin inhibitor heavy chain H2	0.01	0.9331	0.9976	↓	1.00
P02790	Hemopexin	0.01	0.9342	0.9976	↓	1.00
Q06033	Inter-alpha-trypsin inhibitor heavy chain H3	0.01	0.9358	0.9976	↓	0.97
Q07954	Prolow-density lipoprotein receptor-related protein 1	0.01	0.9388	0.9976	↓	0.98
P0C0L4	Complement C4-A	0.01	0.9390	0.9976	↑	1.03
P00740	Coagulation factor IX	0.00	0.9441	0.9976	↑	1.01
P07333	Macrophage colony-stimulating factor 1 receptor	0.00	0.9442	0.9976	↓	0.98
Q92954	Proteoglycan 4	0.00	0.9448	0.9976	↑	1.01
Q6YHK3	CD109 antigen	0.00	0.9453	0.9976	↓	0.97
Q99784	Noelin	0.00	0.9490	0.9976	↓	1.00
Q01459	Di-N-acetylchitobiase	0.00	0.9502	0.9976	↓	0.99
Q9Y6R7	IgGFc-binding protein	0.00	0.9507	0.9976	↑	1.06
Q9NZP8	Complement C1r subcomponent-like protein	0.00	0.9535	0.9976	↑	1.01
P04217	Alpha-1B-glycoprotein	0.00	0.9550	0.9976	↓	1.00
K7EMN2	6-phosphogluconate dehydrogenase, decarboxylating	0.00	0.9590	0.9976	↓	0.95
G3V2W1	Protein Z-dependent protease inhibitor	0.00	0.9607	0.9976	↓	1.00
P12955	Xaa-Pro dipeptidase	0.00	0.9610	0.9976	↓	0.94
P06681	Complement C2	0.00	0.9688	0.9976	↑	1.02
B7ZKJ8	ITIH4 protein	0.00	0.9701	0.9976	↓	0.99
P05154	Plasma serine protease inhibitor	0.00	0.9707	0.9976	↓	1.00
P13796	Plastin-2	0.00	0.9799	0.9976	↓	0.98
P09486	SPARC	0.00	0.9809	0.9976	↓	0.95
P02765	Alpha-2-HS-glycoprotein	0.00	0.9812	0.9976	↑	1.01

© 2020 Mongan D et al. *JAMA Psychiatry.*

P05160	Coagulation factor XIII B chain	0.00	0.9826	0.9976	↓	1.00
P54108	Cysteine-rich secretory protein 3	0.00	0.9883	0.9976	↓	0.98
Q16853	Membrane primary amine oxidase	0.00	0.9943	0.9976	↓	0.98
P05090	Apolipoprotein D	0.00	0.9949	0.9976	↓	0.92
Q10588	ADP-ribosyl cyclase/cyclic ADP-ribose hydrolase 2	0.00	0.9976	0.9976	↓	0.97

PEs: psychotic experiences; FDR: false discovery rate; ALSPAC: Avon Longitudinal Study of Parents and Children

© 2020 Mongan D et al. *JAMA Psychiatry.*

eTable 16: Ten percent highest-weighted features for Model S2 (support vector machine model predicting functional outcome at 24 months in EU-GEI)

Feature	Mean weight
BPRS: suspiciousness	0.197
P01023 Alpha-2-macroglobulin	-0.191
P55058 Phospholipid transfer protein	-0.186
P01871 Immunoglobulin heavy constant mu	-0.182
Q9UGM5 Fetuin-B	0.148
O43866 CD5 antigen-like	-0.145
P14618 Pyruvate kinase	-0.133
P19827 Inter-alpha-trypsin inhibitor heavy chain H1	0.129
SANS: blocking	0.118
SANS: increased latency of response	0.111
P10909 Clusterin	0.107
P08603 Complement factor H	0.104
P36955 Pigment epithelium-derived factor	0.103
MADRS: suicidal thoughts	-0.103
Impersistence at work or school	0.102
P17936 Insulin-like growth factor-binding protein 3	0.102
Age	-0.101
P04196 Histidine-rich glycoprotein	0.099
Q08380 Galectin-3-binding protein	0.097
SANS: grooming and hygeine	0.097
SANS: ability to feel intimacy and closeness	0.095
SANS: sexual activity	-0.093

© 2020 Mongan D et al. *JAMA Psychiatry.*

P11226 Mannose-binding protein C	0.090

Features are ranked according to mean feature weight for models selected in the cross-validation inner loop.

EU-GEI: European Network of National Schizophrenia Networks Studying Gene-Environment Interactions; BPRS: Brief Psychiatric Rating Scale; SANS: Scale for Assessment of Negative Symptoms; MADRS: Montgomery-Asberg Depression Rating Scale

© 2020 Mongan D et al. *JAMA Psychiatry.*

eTable 17: Table comparing performance metrics for multi-class site prediction models based on 69 clinical features from Model 1b

Multiclass prediction	TP, n	FN, n	TN, n	FP, n	Sens., %	Spec., %	Balanced accuracy, %	AUC (95% confidence interval)	PPV, %	NPV, %	LR+	LR-
**London vs. REST**	42	11	58	22	79.2	72.5	75.9	0.76 (0.67 – 0.85)	65.6	84.1	2.9	0.3
**Netherlands vs. REST**	12	3	85	33	80.0	72.0	76.0	0.76 (0.61 – 0.91)	26.7	96.6	2.9	0.3
**Melbourne vs. REST**	1	13	103	16	7.1	86.6	46.8	0.47 (0.31 – 0.63)	5.9	88.8	0.5	1.1
**Switzerland/ Austria vs. REST**	1	13	116	3	7.1	97.5	52.3	0.52 (0.36 – 0.68)	25.0	89.9	2.8	1.0
**Denmark/ France vs. REST**	3	21	109	0	12.5	100.0	56.3	0.56 (0.43 – 0.69)	100.0	83.8	N/A	0.9
**Spain/ Brazil vs. REST**	0	13	120	0	0.0	100.0	50.0	0.50 (0.33 – 0.67)	N/A	90.2	N/A	1.0

TP: true positives; FN: false negatives; TN: true negatives; FP: false positives; Sens.: sensitivity; Spec.: specificity; AUC: area under the receiver-operating curve; PPV: positive predictive value; NPV: negative predictive value; LR+: positive likelihood ratio; LR-: negative likelihood ratio; N/A: not applicable

© 2020 Mongan D et al. *JAMA Psychiatry.*

eTable 18: Table comparing performance metrics for multi-class site prediction models based on 166 proteomic features from Model 1c

Multiclass prediction	TP, n	FN, n	TN, n	FP, n	Sens., %	Spec., %	Balanced accuracy, %	AUC (95% confidence interval)	PPV, %	NPV, %	LR+	LR-
**London vs. REST**	37	16	42	38	69.8	52.5	61.2	0.61 (0.51 – 0.71)	49.3	72.4	1.5	0.6
**Netherlands vs. REST**	8	7	77	41	53.3	65.3	59.3	0.59 (0.43 – 0.75)	16.3	91.7	1.5	0.7
**Melbourne vs. REST**	0	14	112	7	0.0	94.1	47.1	0.47 (0.31 – 0.63)	0.0	88.9	0.0	1.1
**Switzerland/ Austria vs. REST**	1	13	118	1	7.1	99.2	53.2	0.53 (0.37 – 0.69)	50.0	90.1	8.5	0.9
**Denmark/ France vs. REST**	0	24	109	0	0.0	100.0	50.0	0.50 (0.37 – 0.63)	N/A	82.0	N/A	1.0
**Spain/ Brazil vs. REST**	0	13	120	0	0.0	100.0	50.0	0.50 (0.33 – 0.67)	N/A	90.2	N/A	1.0

TP: true positives; FN: false negatives; TN: true negatives; FP: false positives; Sens.: sensitivity; Spec.: specificity; AUC: area under the receiver-operating curve; PPV: positive predictive value; NPV: negative predictive value; LR+: positive likelihood ratio; LR-: negative likelihood ratio; N/A: not applicable

© 2020 Mongan D et al. *JAMA Psychiatry.*

eTable 19: Comparison of performance metrics for uncorrected vs. corrected support vector machine models

	Model 1a: clinical and proteomic (EU-GEI initial, all sites)		Model 1b: clinical (EU-GEI initial, all sites)		Model 1c: proteomic (EU-GEI initial, all sites)		Model 2a: proteomic, non-London (EU-GEI initial, all sites except London)		Model 2b: top 10, training (EU-GEI initial, all sites except London)	
	**Uncorrected**	**Corrected**	**Uncorrected**	**Corrected**	**Uncorrected**	**Corrected**	**Uncorrected**	**Corrected**	**Uncorrected**	**Corrected**
**True positives, n (%)**	48 (98%)	40 (82%)	23 (47%)	29 (59%)	49 (100%)	43 (88%)	28 (93%)	26 (87%)	30 (100%)	23 (77%)
**False negatives, n (%)**	1 (2%)	9 (18%)	26 (53%)	20 (41%)	0 (0%)	6 (12%)	2 (7%)	4 (13%)	0 (0%)	7 (23%)
**True negatives, n (%)**	68 (81%)	71 (85%)	45 (54%)	47 (56%)	71 (85%)	71 (85%)	40 (80%)	42 (84%)	41 (82%)	44 (88%)
**False positives, n (%)**	16 (19%)	13 (15%)	39 (46%)	37 (44%)	13 (15%)	13 (15%)	10 (20%)	8 (16%)	9 (18%)	6 (12%)
**Sensitivity, %**	98.0	81.6	46.9	59.2	100.0	87.8	93.3	86.7	100.0	76.7
**Specificity, %**	81.0	84.5	53.6	56.0	84.5	84.5	80.0	84.0	82.0	88.0
**Balanced accuracy, %**	89.5	83.1	50.3	57.6	92.3	86.1	86.7	85.3	91.0	82.3
**Area under the curve (95% confidence interval)**	0.95 (0.91 – 0.99)	0.91 (0.85 – 0.97)	0.48 (0.38 – 0.58)	0.52 (0.42 – 0.62)	0.96 (0.92 – 1.00)	0.94 (0.89 – 0.99)	0.94 (0.88 – 1.00)	0.96 (0.91 – 1.00)	0.99 (0.96 – 1.00)	0.91 (0.84 – 0.98)
**Positive predictive value, %**	75.0	75.5	37.1	43.9	79.0	76.8	73.7	76.5	76.9	79.3
**Negative predictive value, %**	98.6	88.8	63.4	70.1	100.0	92.2	95.2	91.3	100.0	86.3
**Positive likelihood ratio**	5.1	5.3	1.0	1.3	6.5	5.7	4.7	5.4	5.6	6.4
**Negative likelihood ratio**	<0.1	0.2	1.0	0.7	<0.1	0.1	0.1	0.2	<0.1	0.3

© 2020 Mongan D et al. *JAMA Psychiatry.*

eTable 19, Cont'd:

	Model 3: replication (EU-GEI replication, all sites)		Model 4: Psychotic experiences (ALSPAC)		Model S1: ELISA (EU-GEI initial, all sites)		Model S2: functional outcome (EU-GEI initial, all sites)	
	**Uncorrected**	**Corrected**	**Uncorrected**	**Corrected**	**Uncorrected**	**Corrected**	**Uncorrected**	**Corrected**
**True positives, n (%)**	48 (98%)	46 (94%)	40 (73%)	29 (53%)	33 (75%)	29 (66%)	27 (57%)	32 (68%)
**False negatives, n (%)**	1 (2%)	3 (6%)	15 (27%)	26 (47%)	11 (25%)	15 (34%)	20 (43%)	15 (32%)
**True negatives, n (%)**	77 (90%)	79 (92%)	47 (71%)	39 (59%)	51 (62%)	54 (66%)	22 (69%)	19 (59%)
**False positives, n (%)**	9 (10%)	7 (8%)	19 (29%)	27 (41%)	31 (38%)	28 (34%)	10 (31%)	13 (41%)
**Sensitivity, %**	98.0	93.9	72.7	52.7	75.0	65.9	57.4	68.1
**Specificity, %**	89.5	91.9	71.2	59.1	62.2	65.9	68.8	59.4
**Balanced accuracy, %**	93.7	92.9	72.0	55.9	68.6	65.9	63.1	63.7
**Area under the curve (95% confidence interval)**	0.98 (0.95 – 1.00)	0.97 (0.94 – 1.00)	0.74 (0.65 – 0.83)	0.64 (0.54 – 0.74)	0.76 (0.67 – 0.85)	0.68 (0.58 – 0.78)	0.74 (0.63 – 0.85)	0.71 (0.60 – 0.82)
**Positive predictive value, %**	84.2	86.8	67.8	51.8	51.6	50.9	73.0	71.1
**Negative predictive value, %**	98.7	96.3	75.8	60.0	82.3	78.3	52.4	55.9
**Positive likelihood ratio**	9.4	11.5	2.5	1.3	2.0	1.9	1.8	1.7
**Negative likelihood ratio**	<0.1	0.1	0.4	0.8	0.4	0.5	0.6	0.5

EU-GEI: European Network of National Schizophrenia Networks studying Gene-Environment Interactions; ALSPAC: Avon Longitudinal Study of Parents and Children; ELISA: enzyme-linked immunosorbent assay

© 2020 Mongan D et al. *JAMA Psychiatry.*

eTable 20: Proteins differentially expressed in CHR-T vs. CHR-NT on ANCOVA (*p*<0.05*)* in EU-GEI baseline plasma samples in the initial and replication experiment and predicted systemic impact on coagulation and complement activation and regulation

Pathway	CHR-T vs. CHR-NT, initial experiment (p<0.05)	CHR-T vs. CHR-NT, replication experiment (p<0.05)	Predicted impact
**Complement**	C1Q (↑), C1R (↑) C4BP (↓)<br>SERPING1 (↓)<br><br>FIC3 (↓)<br><br>CFB (↑)<br>CFI (↑), CFH (↑)<br><br>C8 (↑), CLU (↑)	C1Q (↑), C1R (↑), C2 (↑), C4-A (↓)<br>C4BP (↓)<br>SERPING1 (↓)<br><br>FIC3 (↓)<br><br>CFD (↑)<br>CFI (↑), CFH (↑)<br><br>C5 (↑), C6 (↑), C7(↑), C8 (↑), C9 (↑),CLU (↑), VTN, (↑) CPN (↑), CPB2 (↑)	Classical pathway: activation ↑ regulation ↓<br><br>iC3b generation ↑<br><br>Terminal pathway: activation ↑ regulation ↑
**Coagulation**	FXI (↑)<br>SERPING1(↓)<br>SERPIND1 (↓)<br><br>ADAMTS13 (↓)<br><br>PLG (↑)	IX (↑), CPN (↑) SERPING1(↑)<br>VWF (↑)<br><br>FII (↑), FV (↑), SERPINF2 (↑)<br>SERPIND1 (-)<br>CPB2 (↑)<br><br>XIII (↑), FIB (↓)<br><br>PLG (↑)	Intrinsic pathway activation ↑ regulation ↓<br><br>Thrombin generation ↑<br>Thrombin regulation ↓<br><br>Fibrin generation ↑<br><br>Plasmin generation ↑
**Associated components**	IGM, IGG (↓)<br><br>A2M (↓)	IGM, IGG (↓)<br><br>A2M (↓)	Complement classical pathway ↑<br><br>Plasmin/thrombin regulation ↓

Notes

The directions of the differentially expressed proteins indicate a general upregulation of activation components and downregulation of regulatory proteins.

With regards to the complement system, we observe upregulation of complement classical pathway components (C1Q, C1R, C2, C4 and IGG, IGM), while key regulators (SERPING1, C4BP) are downregulated. Classical pathway activation would lead to alternative pathway activation and amplification leading to opsonin C3b generation.[1] Key alternative pathway regulatory components (CFH, CFI) are upregulated resulting in increased iC3b, both C3b and iC3b serve as ligands for selective complement receptors on leukocytes.[2] Terminal pathway components are upregulated (C5, C6, C7, C8, C9) leading to an increase in the terminal complement complex C5b-9, while terminal pathway regulators are also upregulated (CLU, VTN), which bind to the nascent amphiphilic C5b-9 complex, rendering it soluble and lytically inactive.[3] Overall, we expect an enhanced output of complement activation products, as a result of enhanced classical pathway activation and dysregulation, amplified through the alternative pathway, while increased terminal pathway components drive the generation of soluble terminal complement component (sTCC).

© 2020 Mongan D et al. *JAMA Psychiatry.*

In the coagulation system, the primary effect is a downregulation of intrinsic pathway control (SERPING1), thrombin (SERPIND1) and plasmin (A2M) regulation while increased plasminogen (PLG) drives plasmin generation. Overall, we expect an increase in thrombin generation and dysregulation, with increased plasmin generation.

1. Merle NS, Church SE, Fremeaux-Bacchi V, Roumenina LT. Complement System Part I - Molecular Mechanisms of Activation and Regulation. Front Immunol. 2015;6:262.

2. van Lookeren Campagne M, Wiesmann C, Brown EJ. Macrophage complement receptors and pathogen clearance. Cellular microbiology. 2007;9(9):2095-102.

3. Choi NH, Nakano Y, Tobe T, Mazda T, Tomita M. Incorporation of SP-40,40 into the soluble membrane attack complex (SMAC, SC5b-9) of complement. International immunology. 1990;2(5):413-7.

© 2020 Mongan D et al. *JAMA Psychiatry.*

eTable 21: Table summarising directionality of effect of ten percent highest weighted features in Model 1a (EU-GEI initial experiment), Model 3 (EU-GEI replication experiment) and Model 4 (ALSPAC proteomic data)

**Model 1a: EU-GEI clinical and proteomic data, initial experiment**		**Model 3: EU-GEI clinical and proteomic data, replication experiment**		**Model 4: ALSPAC proteomic data**	
**Feature**	**Directionality of effect (CHR-T vs. CHR-NT)**	**Feature**	**Directionality of effect (CHR-T vs. CHR-NT)**	**Feature**	**Directionality of effect (PE at 18 vs. no PE at 18)**
P01023 Alpha-2-macroglobulin	↓	P01023 Alpha-2-macroglobulin	↓	P04003 C4b-binding protein alpha chain	↓
P01871 Immunoglobulin heavy constant mu	↓	P22792 Carboxypeptidase N subunit 2	↑	P27169 Serum paraoxonase/arylesterase 1	↓
P04003 C4b-binding protein alpha chain	↓	P01871 Immunoglobulin heavy constant mu	↓	Q03591 Complement factor H-related protein 1	↓
P07357 Complement component 8 alpha chain	↑	P09871 Complement C1s subcomponent	↓	P07225 Vitamin K-dependent protein S	↓
P55058 Phospholipid transfer protein	↓	P01011 Alpha-1-antichymotrypsin	↑	P61626 Lysozyme C	↓
O75636 Ficolin-3	↓	P00747 Plasminogen	↑	P55103 Inhibin beta C chain	↑
P02774 Vitamin D binding protein	↑	P08571 Monocyte differentiation antigen CD14	↑	Q08380 Galectin-3-binding protein	↑
P07225 Vitamin K-dependent protein S	↓	P10909 Clusterin	↑	P24593 Insulin-like growth factor-binding protein 5	↑
P43320 Beta-crystallin B2	↑	Q16610 Extracellular matrix protein 1	↑	P00746 Complement factor D	↑
P02766 Transthyretin	↓	G3XAM2 Complement factor I	↑	P01019 Angiotensinogen	↓
P23142 Fibuln-1	↑	P04003 C4b binding protein alpha chain	↓	P01871 Immunoglobulin heavy constant mu	↓
P10909 Clusterin	↑	P13671 Complement component 6	↑	O75636 Ficolin-3	↑
P05155 Plasma protease C1 inhibitor	↓	P25311 Zinc alpha-2-glycoprotein	↑	Q9H4A9 Dipeptidase 2	↓
Sex (female vs. male)	↓	P07359 Platelet glycoprotein Ib alpha chain	↑	P01023 Alpha-2-macroglobulin	↓
P00747 Plasminogen	↑	P01031 Complement C5	↑	P04275 von Willebrand factor	↓
P13671 Complement component 6	↑	O75882 Attractin	↑	Q9NQ79 Cartilage acidic protein 1	↑
P02747 Complement C1q subcomponent subunit C	↑	P0DOY3 Immunoglobulin lambda constant 3	↓	P24592 Insulin-like growth factor-binding protein 6	↑
P02753 Retinol-binding protein 4	↑	P15169 Carboxypeptidase N catalytic chain (CPN)	↑	P09871 Complement C1s subcomponent	↓

© 2020 Mongan D et al. *JAMA Psychiatry.*

Q76LX8 A disintegrin and metalloproteinase with thrombospondin motifs 13	↓			P10909 Clusterin	↓
P08697 Alpha-2-antiplasmin	↓			O95497 Pantetheinase	↑
P19827 Inter-alpha-trypsin inhibitor heavy chain H1	↑			P02654 Apolipoprotein C-I	↓
MADRS: concentration difficulties	↓			P02679 Fibrinogen gamma chain	↓
P02489 Alpha-crystallin A chain	↑			P07358 Complement component C8 beta chain	↑
				Q5T7F0 Neuropilin	↓
				P04040 Catalase	↑
				P43251 Biotinidase	↑

© 2020 Mongan D et al. *JAMA Psychiatry.*

eFigure 1: Derivation and testing of Model 2b: parsimonious (10-predictor) proteomic model

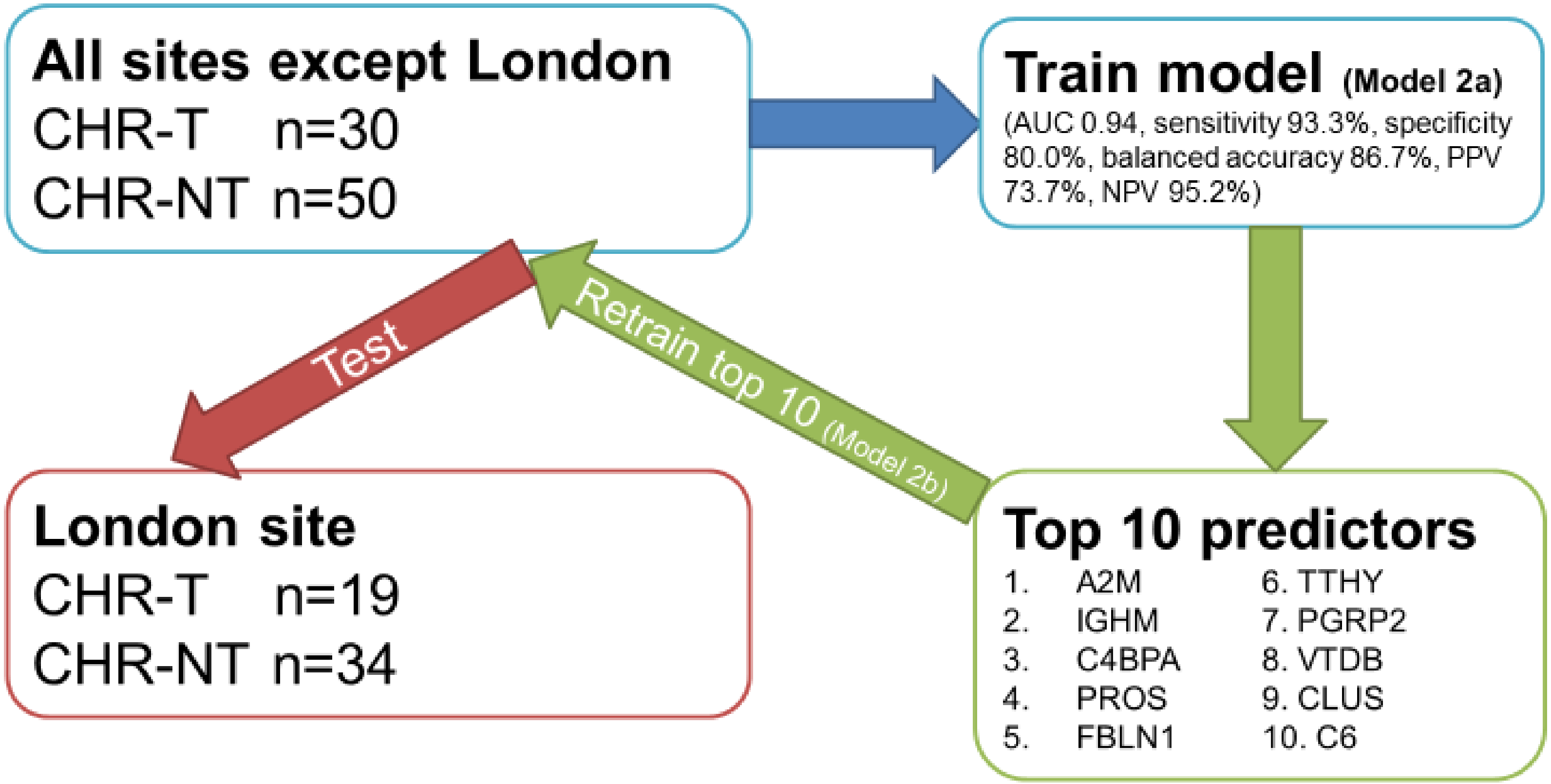

Notes. To derive the 10 highest-weighted proteins for prediction of transition outcome, an L2-regularised SVM model was trained using the proteomic data from all sites except London (CHR-T n=30, CHR-NT n=50), with leave-site-out cross-validation (Model 2a). The top 10 predictors, ranked by mean feature weight across models selected in the inner loop, are presented in the figure. Next, a reduced model based solely on these 10 features (Model 2b) was trained using data from all sites except London (Model 1d). This reduced model was then tested in the held-out London sample (CHR-T n=19, CHR-NT n=34).

AUC: area under the receiver-operating curve; PPV: positive predictive value; NPV: negative predictive value; A2M: alpha-2-macroglobulin; IGHM: immunoglobulin heavy constant mu; C4BPA: C4b-binding protein alpha chain; PROS: vitamin K-dependent protein S; FBLN1: Fibulin-1; TTHY: transthyretin; PGRP2: N-acetylmuramoyl-L-alanine amidase; VTDB: vitamin D binding protein; CLUS: clusterin; C6: complement component 6

© 2020 Mongan D et al. *JAMA Psychiatry.*

eFigure 2: STRING functional protein association network for proteins significantly differentially expressed (following false discovery rate correction) between CHR-T and CHR-NT in EU-GEI initial experiment

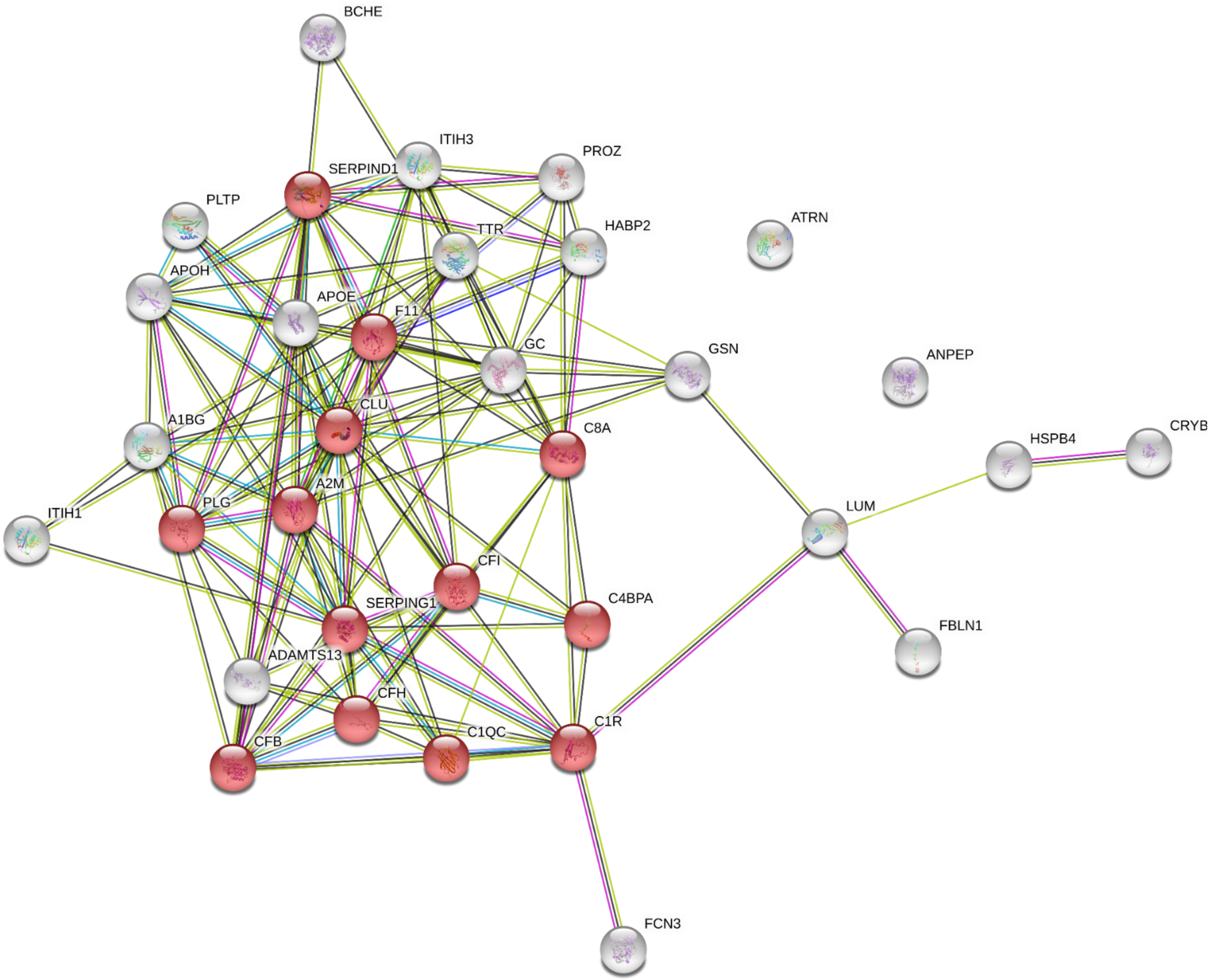

The network nodes are proteins. Proteins implicated in the complement and coagulation cascades are highlighted in red.

The edges represent functional associations between proteins, and the colour of each edge represents the source of evidence for that association:

Red line - fusion evidence
Green line - neighbourhood evidence
Blue line – co-occurrence evidence
Purple line - experimental evidence
Yellow line – text-mining evidence
Light blue line - database evidence
Black line – co-expression evidence.

Note: IGHM is not listed in the STRING database and hence is not shown.

STRING database: https://string-db.org/

© 2020 Mongan D et al. *JAMA Psychiatry.*

eFigure 3: Mean algorithm scores and class predictions (A) and receiver-operating characteristic curve (B) for Model 1b: clinical data

A

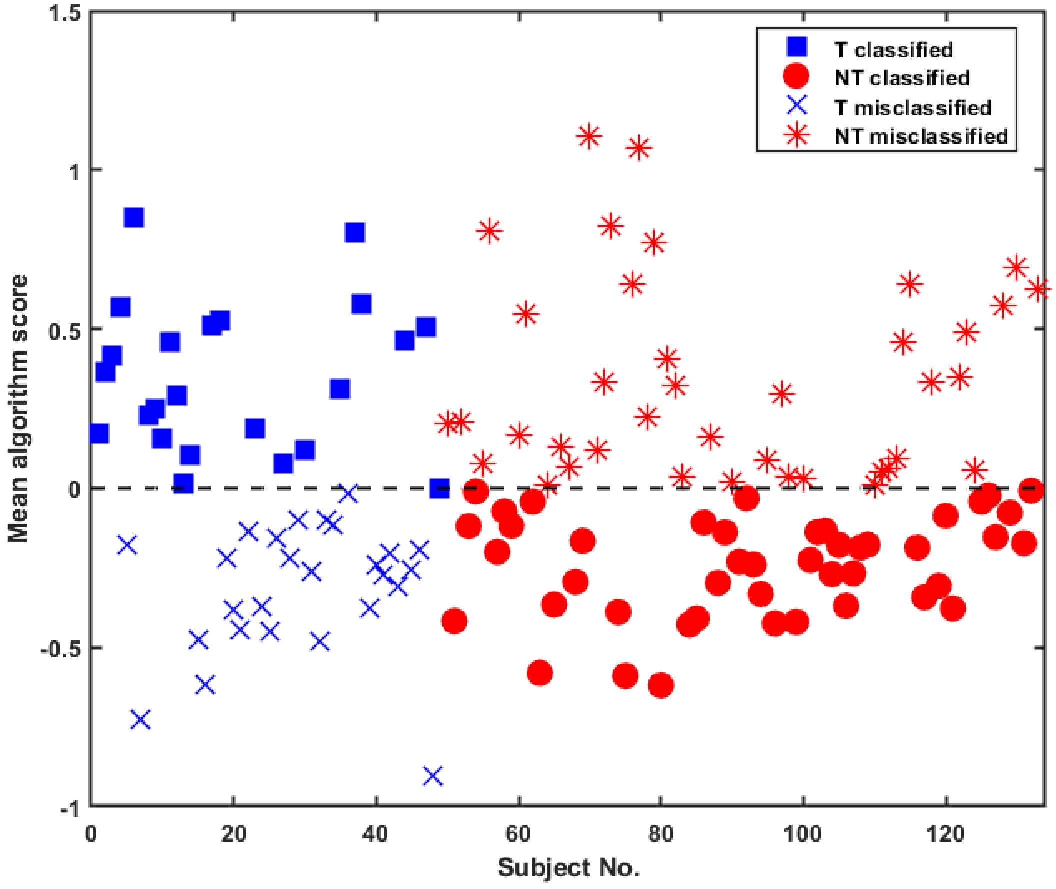

T: clinical high-risk participants who transitioned to first episode psychosis; NT: clinical high-risk participants who did not transition.

B

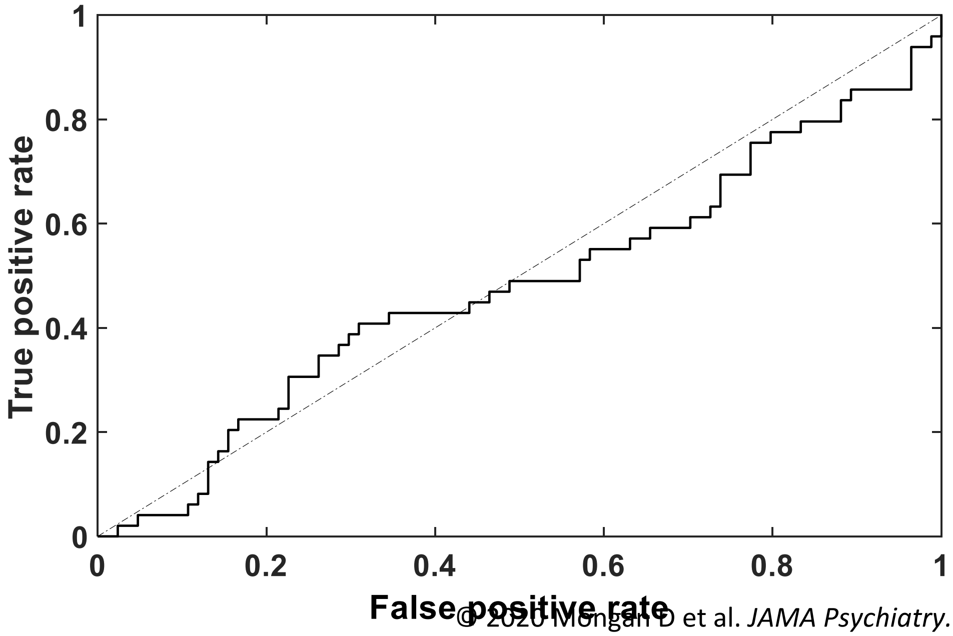

© 2020 Mongan D et al. *JAMA Psychiatry.*

eFigure 4: Mean algorithm scores and class predictions (A) and receiver-operating characteristic curve (B) for Model 1c: proteomic data

A

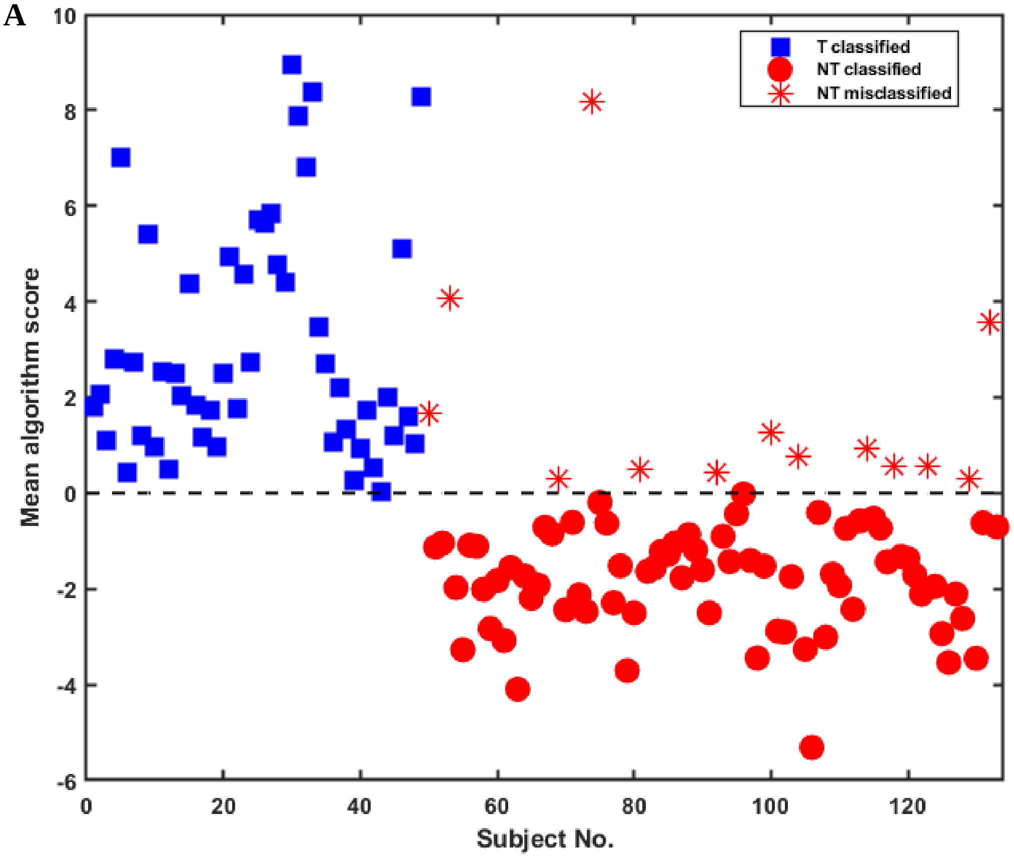

T: clinical high-risk participants who transitioned to first episode psychosis; NT: clinical high-risk participants who did not transition.

B

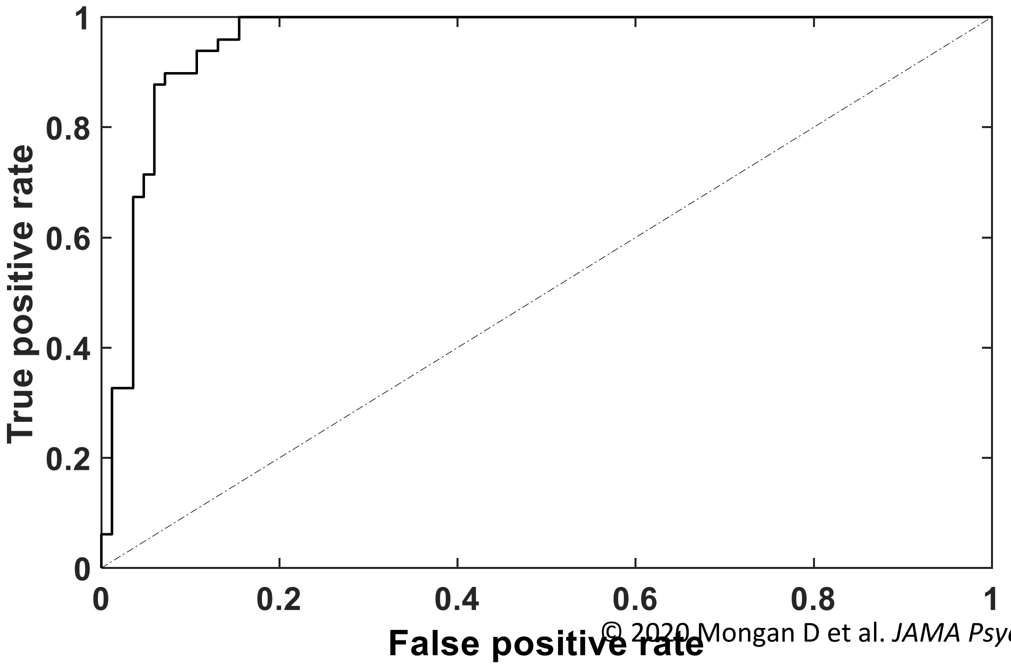

© 2020 Mongan D et al. *JAMA Psychiatry.*

eFigure 5: Mean algorithm scores and class predictions (A) and receiver-operating characteristic curve (B) for Model 2a: proteomic (non-London)

A

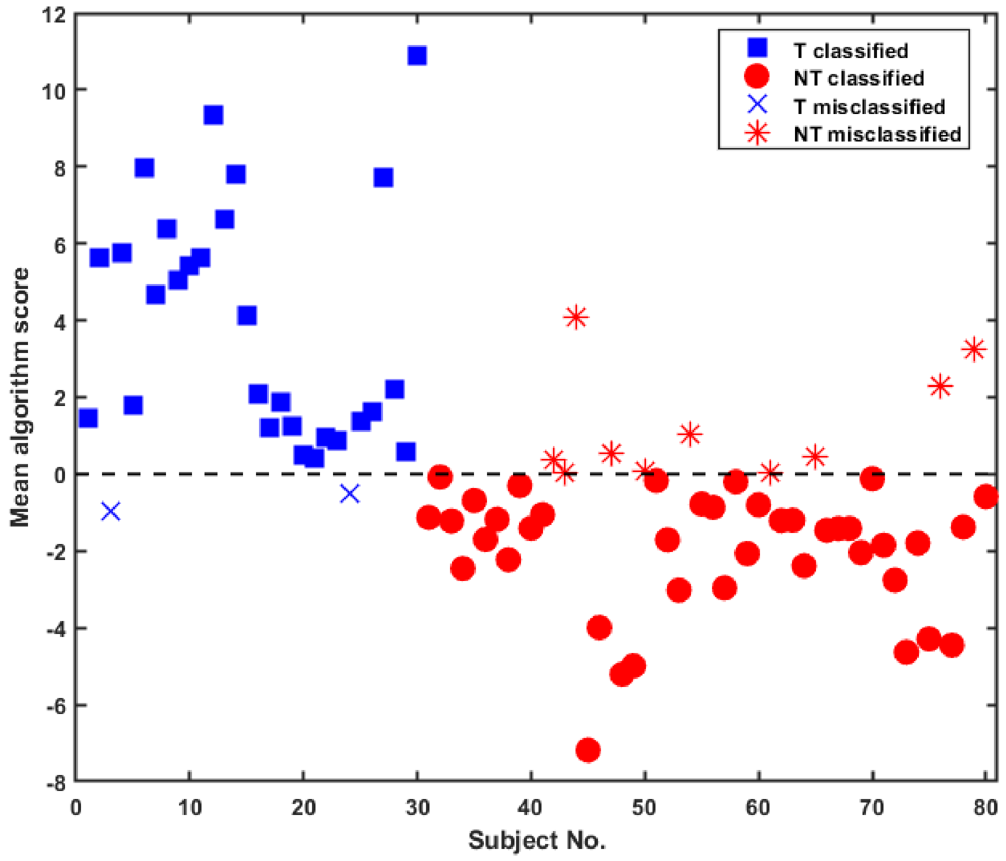

T: clinical high-risk participants who transitioned to first episode psychosis; NT: clinical high-risk participants who did not transition.

B

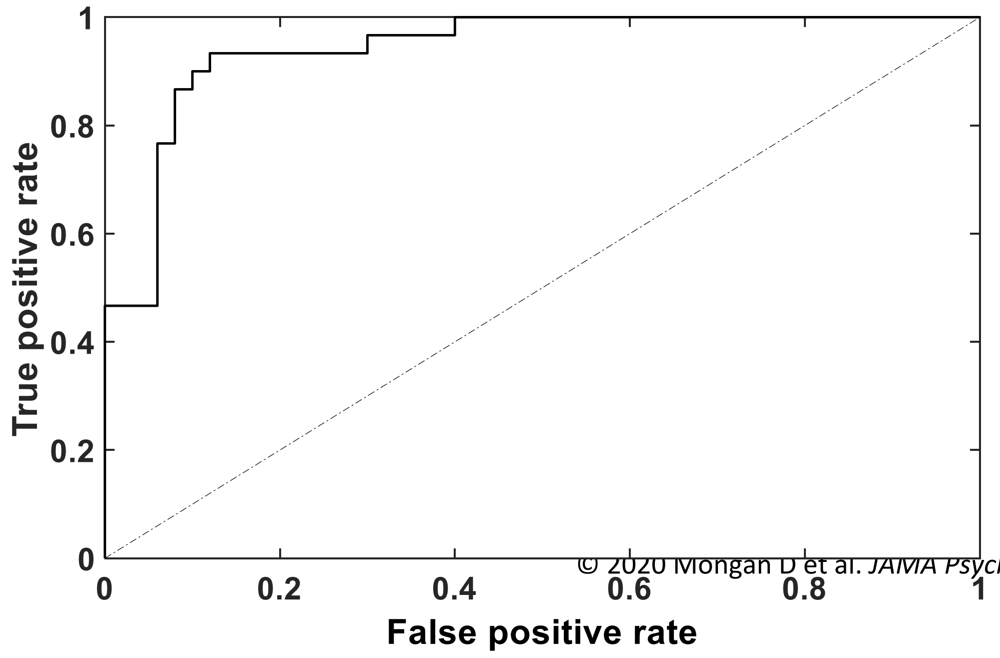

© 2020 Mongan D et al. *JAMA Psychiatry.*

eFigure 6: Mean algorithm scores and class predictions (A) and receiver-operating characteristic curve (B) for Model 2b: parsimonious (10-predictor) proteomic model, training data (non-London)

A

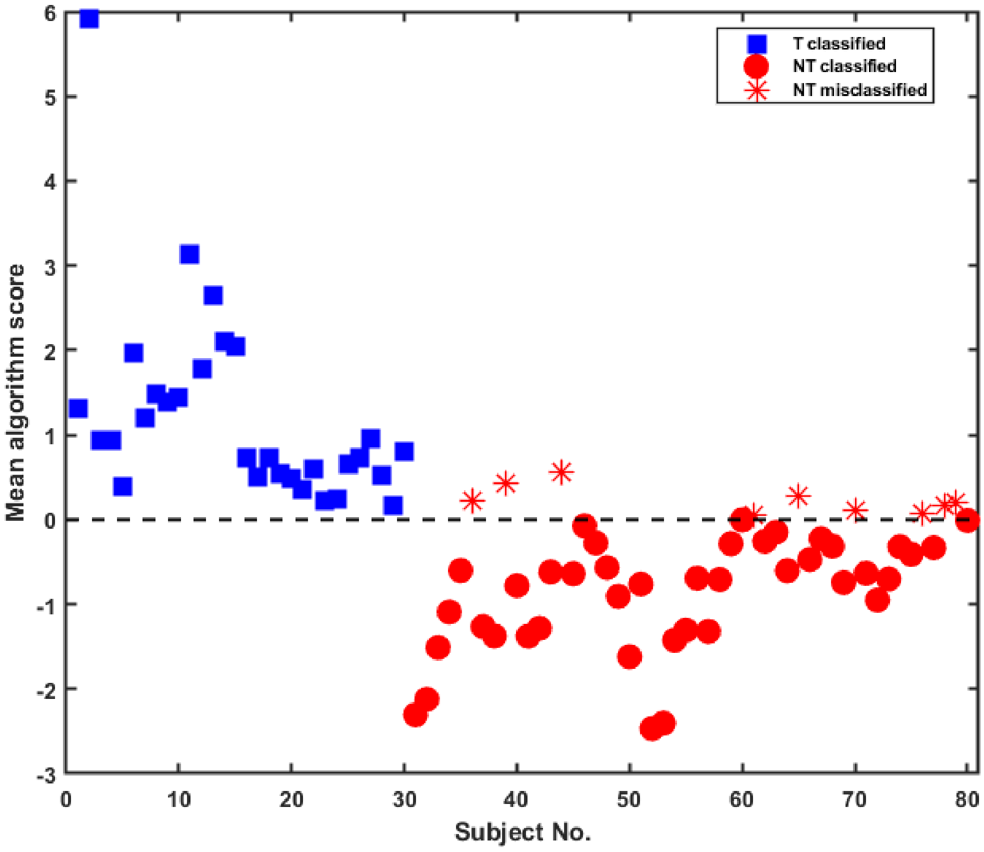

B

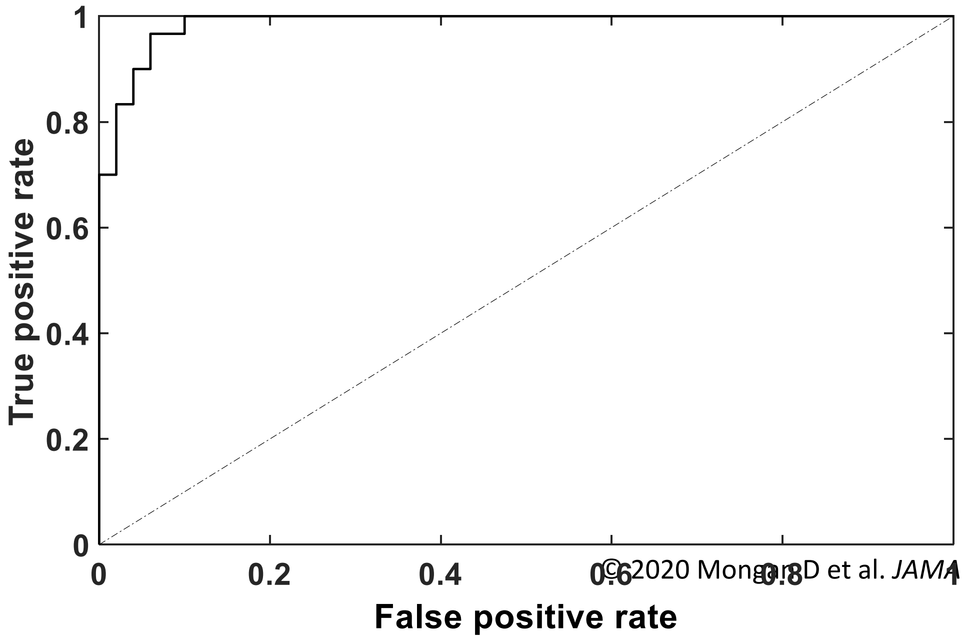

© 2020 Mongan D et al. *JAMA Psychiatry.*

eFigure 7: Mean algorithm scores and class predictions (A) and receiver-operating characteristic curve (B) for Model 3: replication

A

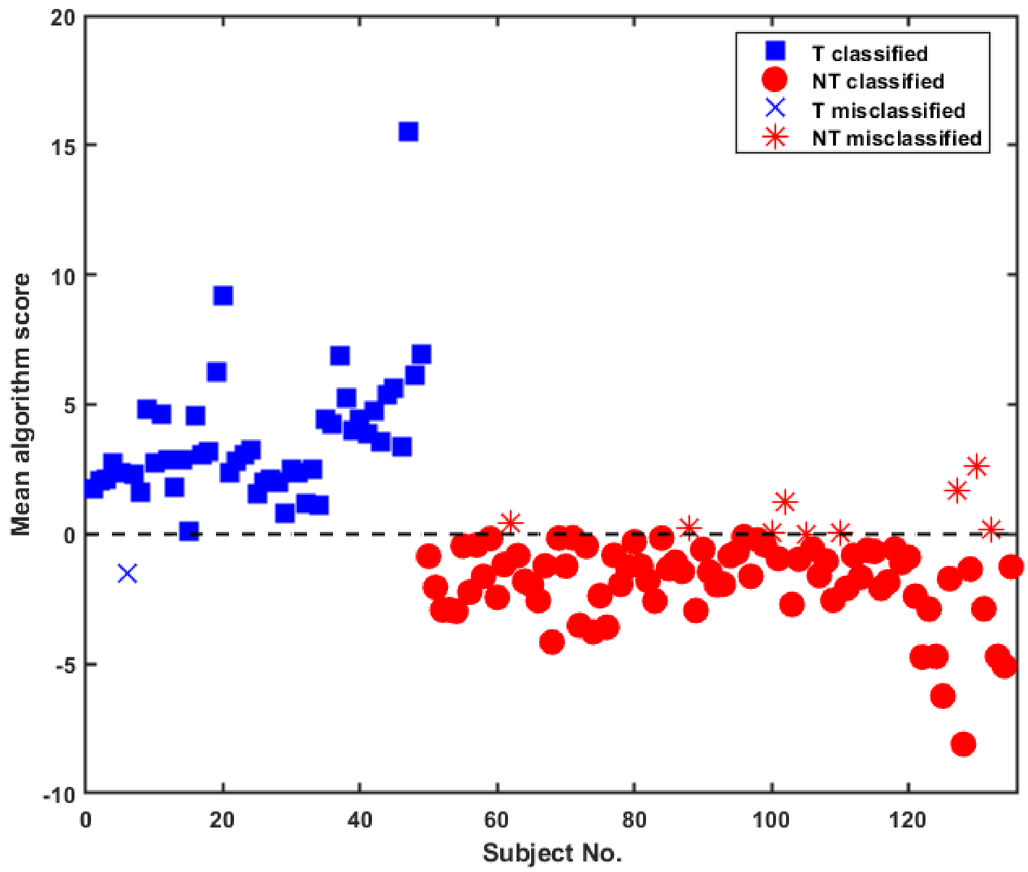

T: clinical high-risk participants who transitioned to first episode psychosis; NT: clinical high-risk participants who did not transition

B

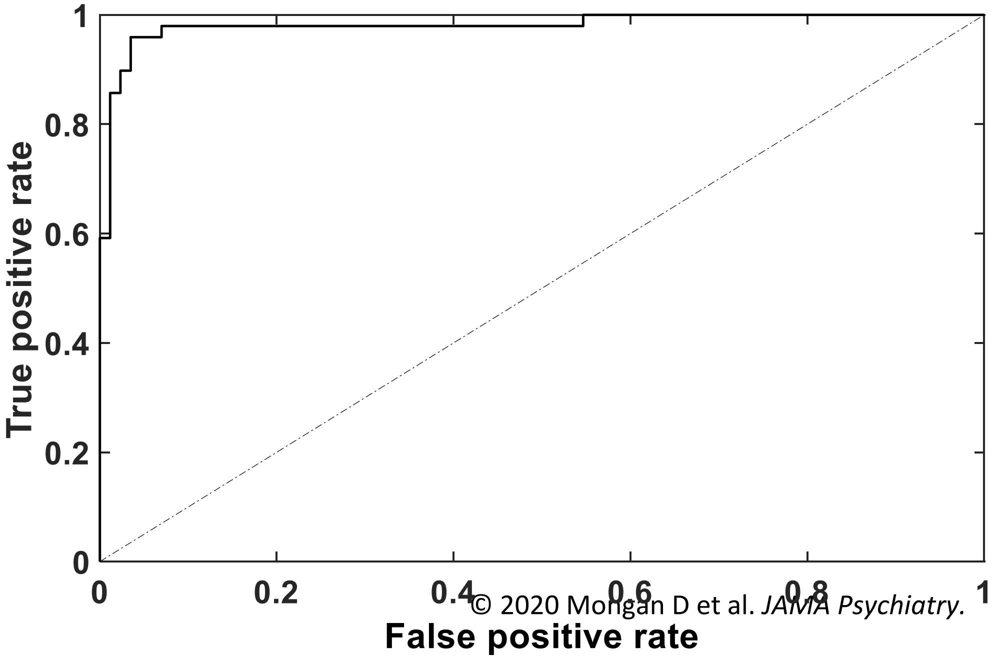

© 2020 Morgan D et al. *JAMA Psychiatry.*

eFigure 8: Mean algorithm scores and class predictions (A) and receiver-operating characteristic curve (B) for Model 4: ALSPAC proteomic data

A

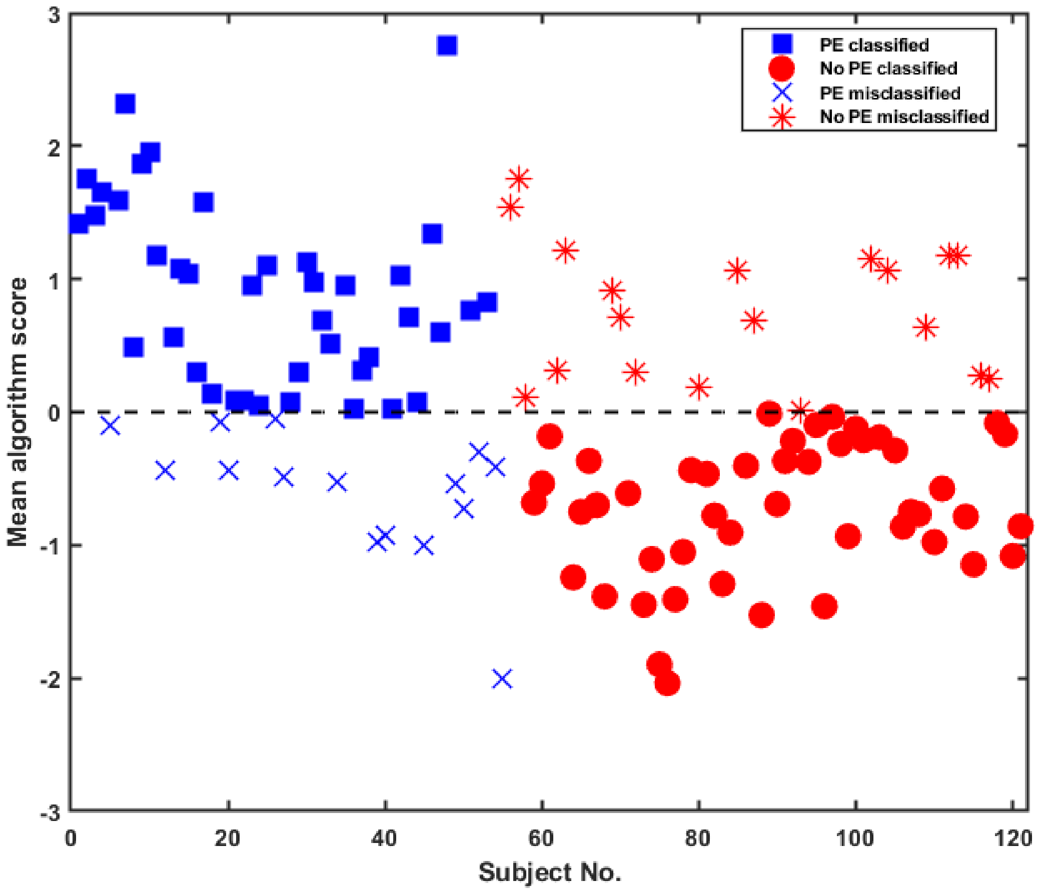

PE: psychotic experiences at 18; No PE: No psychotic experiences at 18.

B

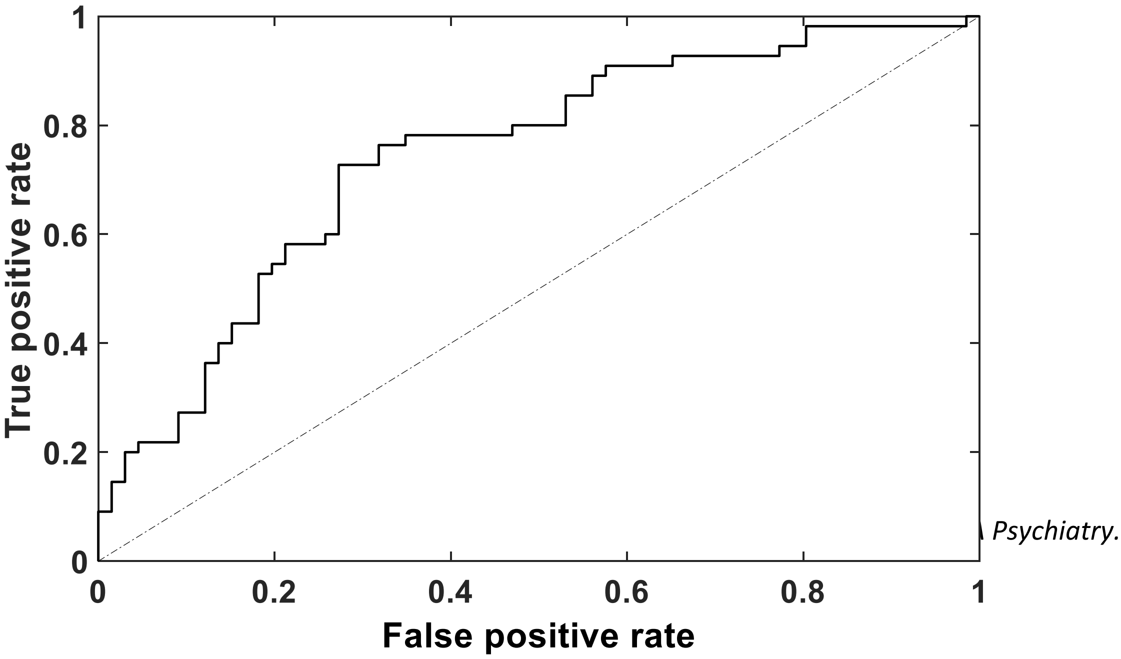

Psychiatry.

eFigure 9: Mean algorithm scores and class predictions (A) and receiver-operating characteristic curve (B) for Model S1: ELISA

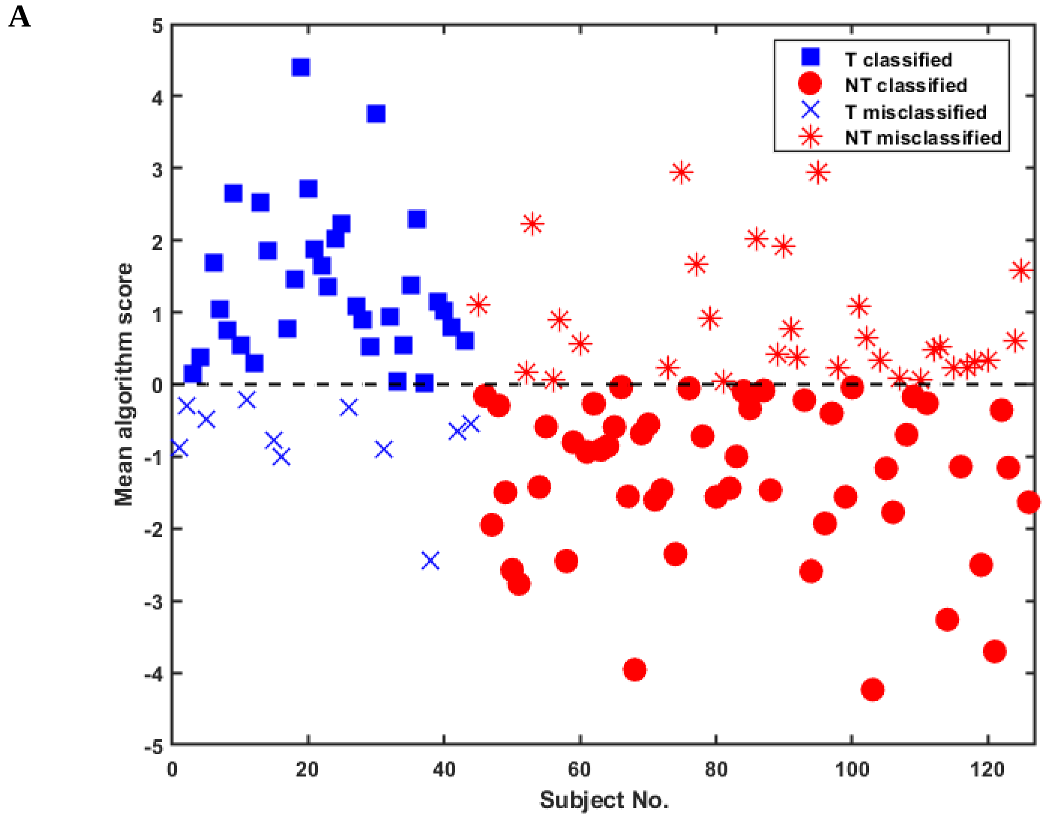

T: clinical high-risk participants who transitioned to first episode psychosis; NT: clinical high-risk participants who did not transition

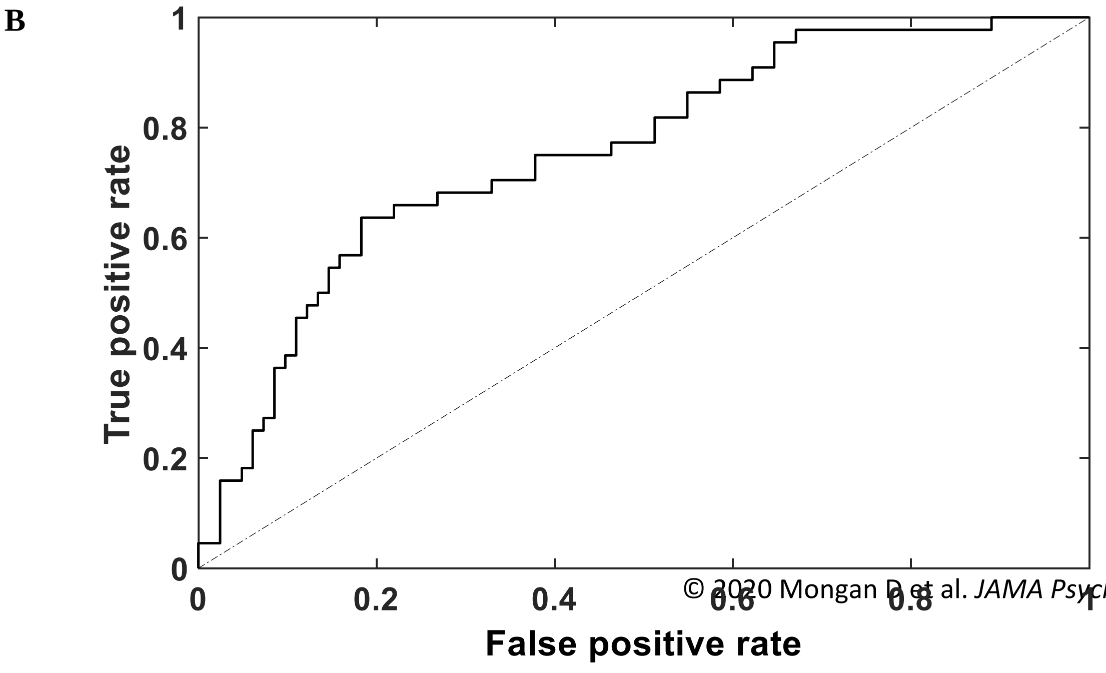

© 2020 Mongan D et al. *JAMA Psychiatry.*

eFigure 10: Mean algorithm scores and class predictions (A) and receiver-operating characteristic curve (B) for Model S2: functional outcome

A

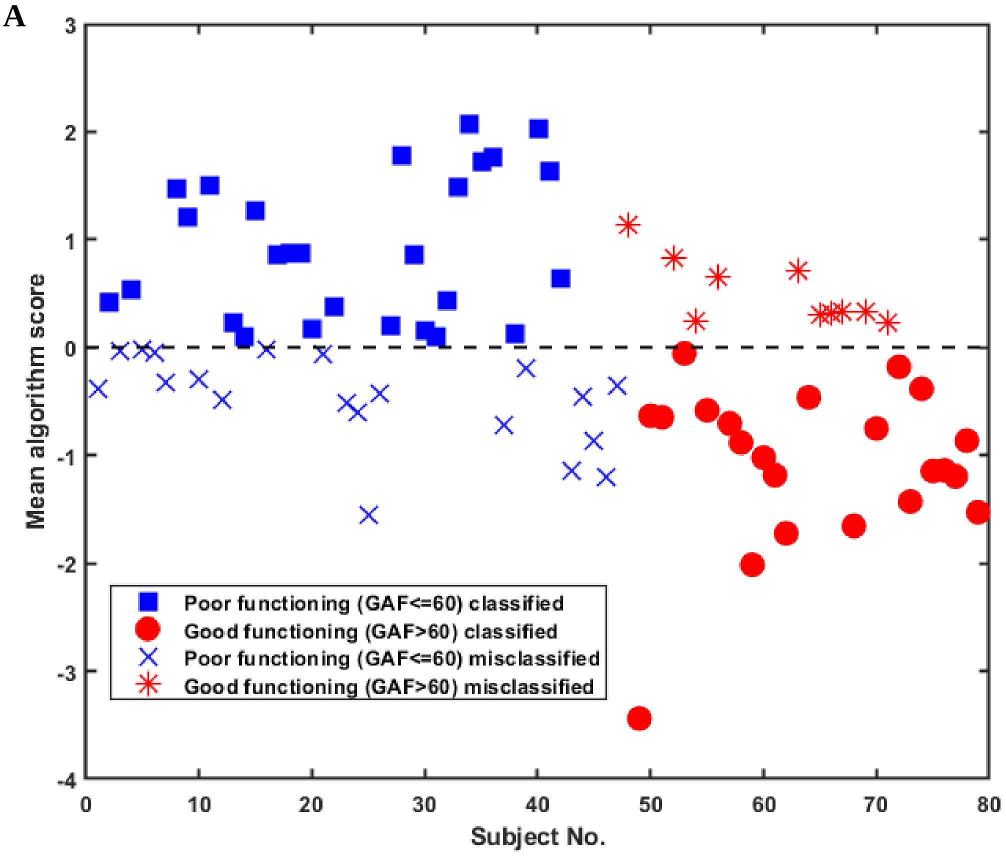

GAF: General Assessment of Functioning (disability subscale); Poor: GAF score ≤60 at 24 months; Good: GAF score >60 at 24 months

B

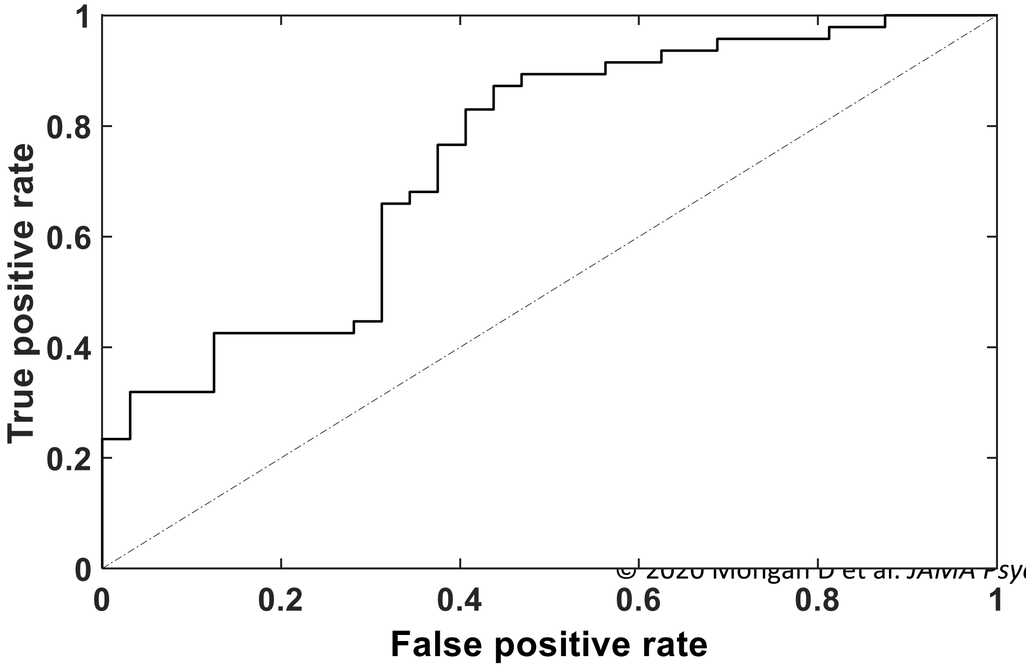

© 2020 Morgan D et al. *JAMA Psychiatry.*

eFigure 11: Model 1b (clinical data) decision scores stratified by EU-GEI site

a. Box plots for Model 1b (clinical data) decision scores, stratified by EU-GEI site

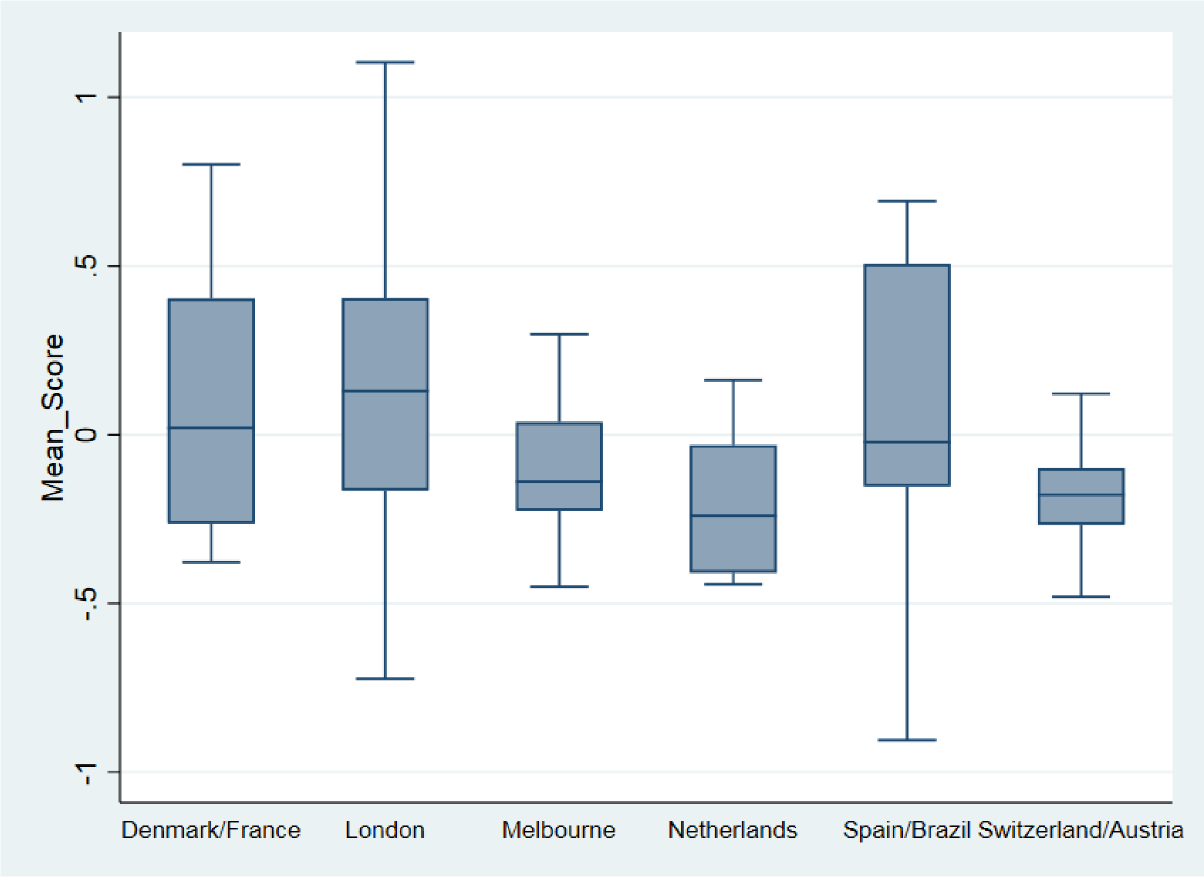

b. Kruskall-Wallis test

	London	Netherlands	Melbourne	Switzerland/Austria	Denmark/France
Netherlands	3.308				
p	**0.007**				
Melbourne	2.021	-0.969			
p	0.324	1.000			
Switzerland/Austria	2.626	-0.480	0.481		
p	0.065	1.000	1.000		
Denmark/France	0.786	-2.352	-1.231	-1.771	
p	1.000	0.140	1.000	0.574	
	0.349	-2.269	-1.297	-1.768	-0.248

© 2020 Mongan D et al. *JAMA Psychiatry.*

Spain/Brazil					
p	1.000	0.175	1.000	0.578	1.000

Chi-squared (5 d.f.): 16.878

c. Post-hoc Dunn's test pairwise comparisons with Bonferroni-corrected p values

p = 0.0047

eFigure 12: Model 1c (proteomic data) decision scores stratified by EU-GEI site

a. Box plots for Model 1c (proteomic data) decision scores stratified by EU-GEI site

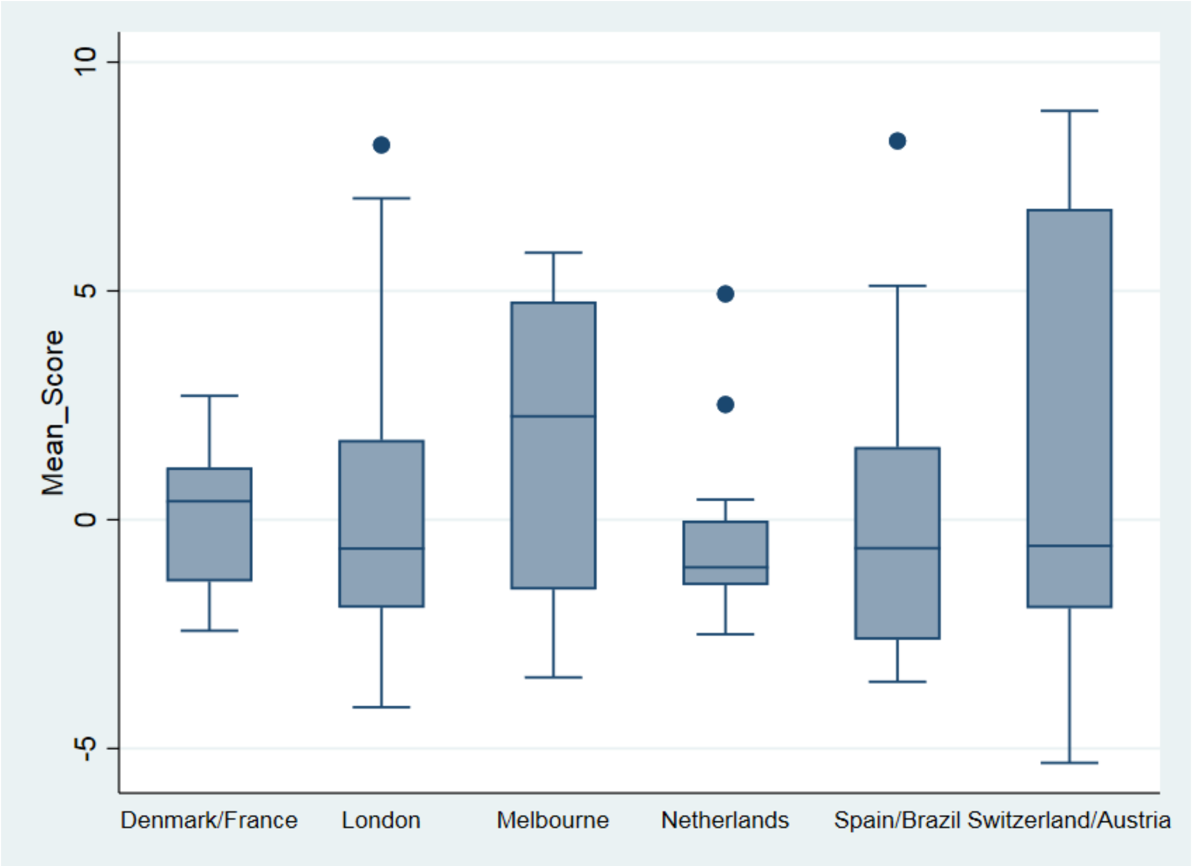

b. Kruskall-Wallis test

Chi-squared (5 d.f.): 3.512
p = 0.6216

© 2020 Mongan D et al. *JAMA Psychiatry.*

eFigure 13: Age stratified by EU-GEI site

a. Box plots for age in years, stratified by EU-GEI site

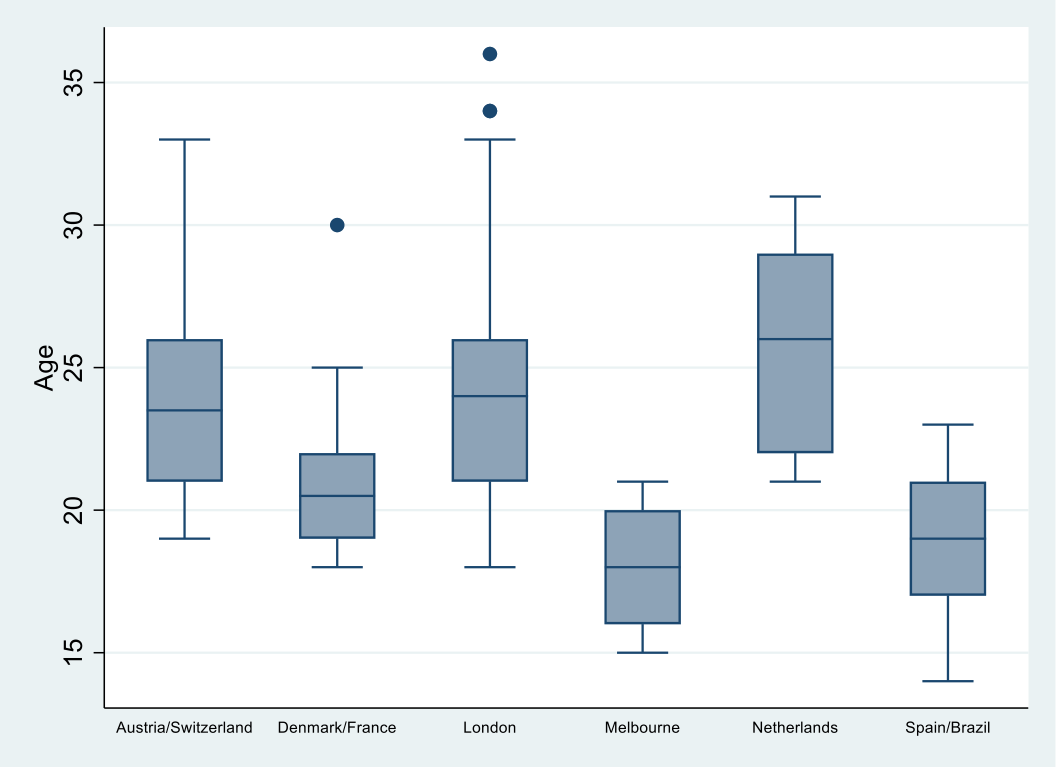

b. Kruskall-Wallis test

Chi-sq (5 d.f.): 49.157
p = 0.0001

c. Post-hoc Dunn's test pairwise comparisons with Bonferroni-corrected p values

	Austria/Switzerland	Denmark/France	London	Melbourne	Netherlands
Denmark/France	2.283				
p	0.168				
London	0.077	-3.027			
p	1.000	**0.019**			
Melbourne	4.034	2.250	4.996		
p	**<0.001**	0.183	**<0.001**		

© 2020 Mongan D et al. *JAMA Psychiatry.*

	Austria/Switzerland	Denmark/France	London	Melbourne	Netherlands
Netherlands	-1.012	-3.476	-1.366	-5.115	
p	1.000	**0.004**	1.000	**<0.001**	
Spain/Brazil	3.297	1.458	4.029	-0.661	4.344
p	**0.007**	1.000	**<0.001**	1.000	**<0.001**

eFigure 14: Years in education stratified by EU-GEI site

a. Box plots for years in education, stratified by EU-GEI site

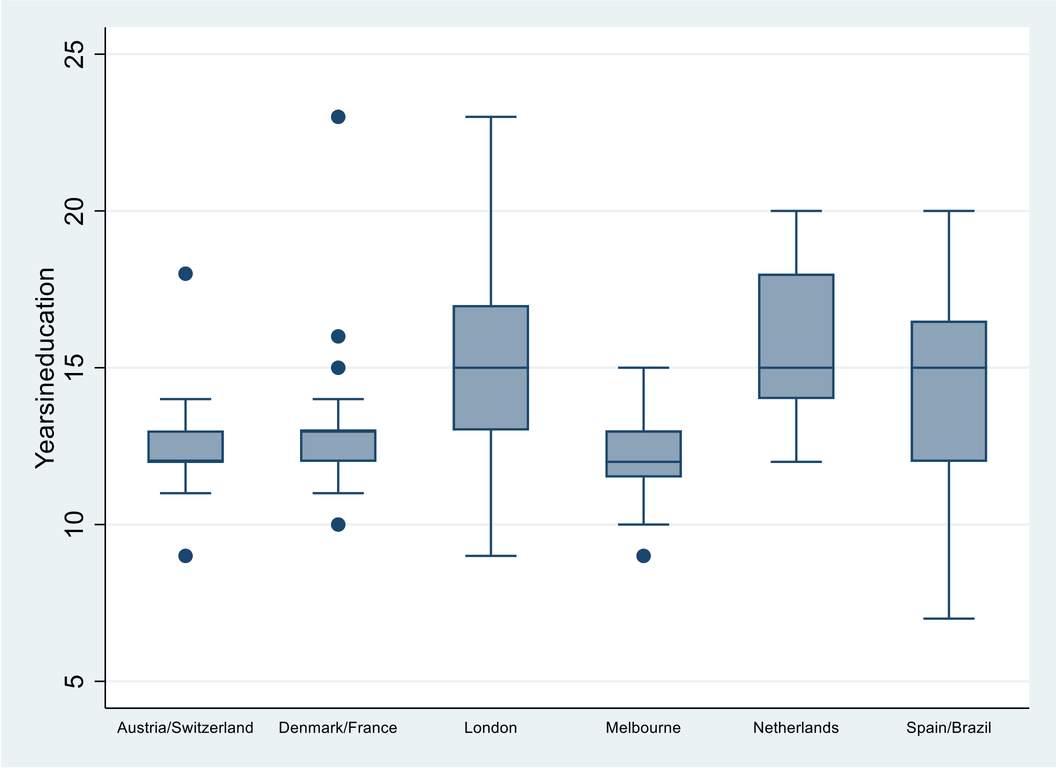

b. Kruskall-Wallis test

Chi-sq (5 d.f.): 23.921
p = 0.0002

c. Post-hoc Dunn's test pairwise comparisons with Bonferroni-corrected p values

	Austria/Switzerland	Denmark/France	London	Melbourne	Netherlands
Denmark/France	-0.350				
p	1.000				
London	-2.528	-2.688			
p	0.086	0.054			
Melbourne	0.291	0.704	3.115		

© 2020 Mongan D et al. *JAMA Psychiatry.*

p	1.000	1.000	**0.014**	
Netherlands	-3.064	-3.184	-1.290	-3.551
p	**0.016**	**0.011**	1.000	**0.003**

eFigure 15: Body mass index stratified by EU-GEI site

a. Box plots for body mass index (BMI) in kg/m^2, stratified by EU-GEI site

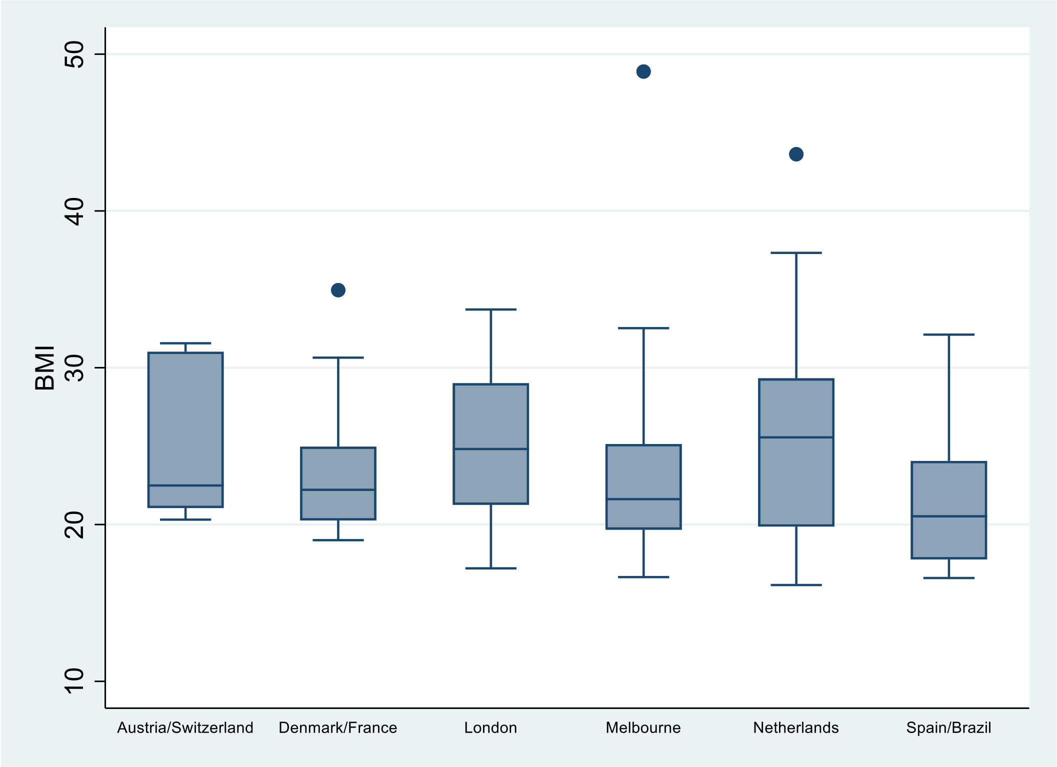

b. Kruskall-Wallis test

Chi-sq (5 d.f.): 8.985
p = 0.1097

© 2020 Mongan D et al. *JAMA Psychiatry.*

eFigure 16: Sex stratified by EU-GEI site

a. Sex distribution, stratified by EU-GEI site

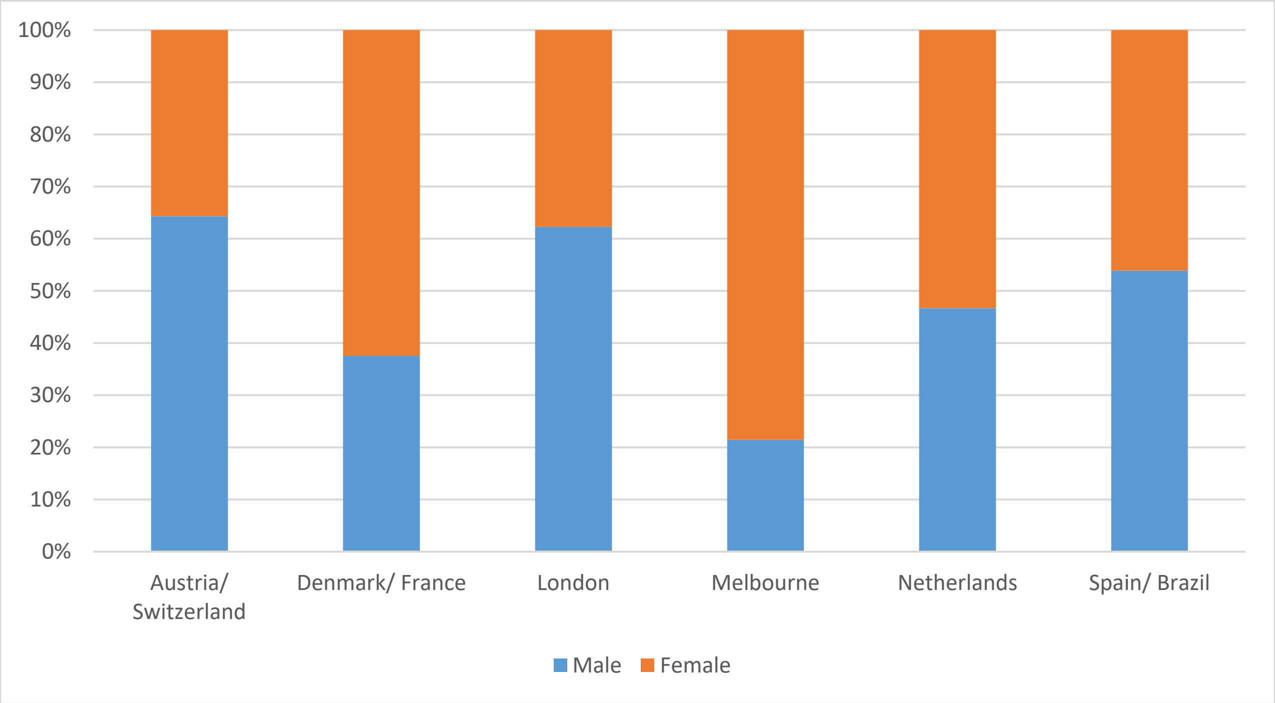

b. Chi-squared test

	Male, n (%)	Female, n (%)
**Austria/ Switzerland**	9 (64%)	5 (36%)
**Denmark/ France**	9 (28%)	15 (62%)
**London**	33 (62%)	20 (38%)
**Melbourne**	3 (21%)	11 (79%)
**Netherlands**	7 (47%)	8 (53%)
**Spain/ Brazil**	7 (54%)	6 (46%)

© 2020 Mongan D et al. *JAMA Psychiatry.*

Chi-squared (5 d.f.): 10.4852, *p*=0.063

eFigure 17: General Assessment of Functioning symptoms subscale stratified by EU-GEI site

a. Box plots for General Assessment of Functioning (GAF) symptoms subscale score, stratified by EU-GEI site

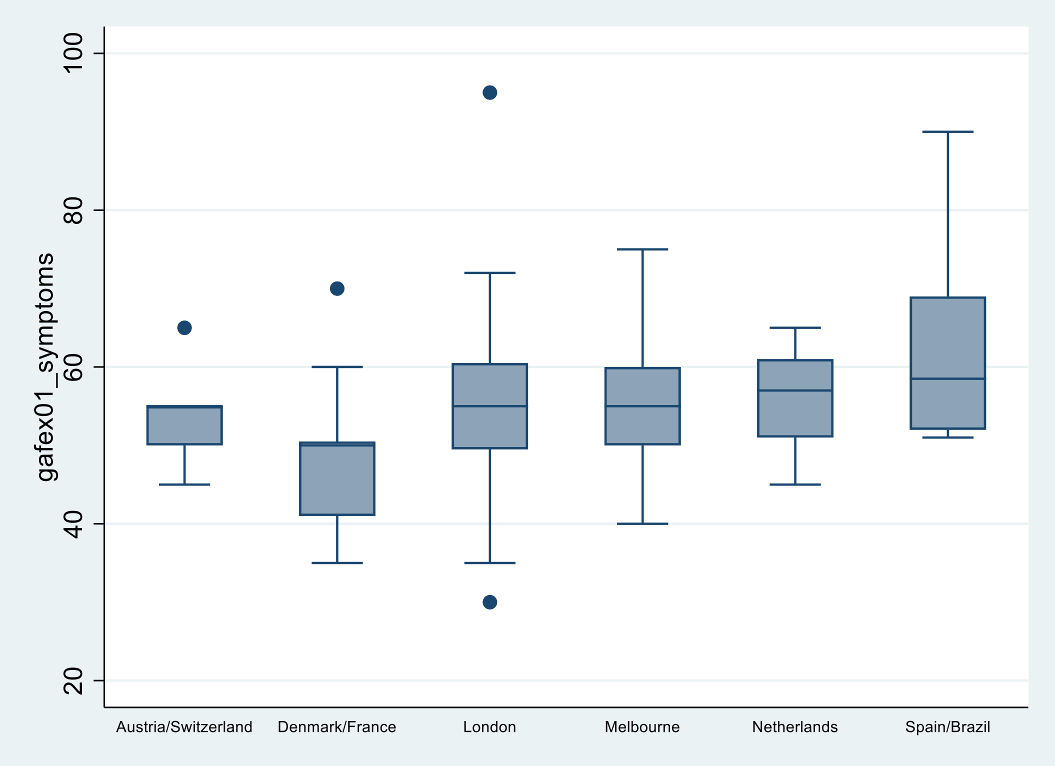

b. Kruskall-Wallis test

Chi-sq (5 d.f.): 15.575
p = 0.0082

c. Post-hoc Dunn's test pairwise comparisons with Bonferroni-corrected p values

	Austria/Switzerland	Denmark/France	London	Melbourne	Netherlands
Denmark/France	1.218				
p	1.000				
London	-0.952	-2.624			
p	1.000	0.065			
Melbourne	-0.688	-1.941	0.082		

© 2020 Mongan D et al. *JAMA Psychiatry.*

p	1.000	0.392	1.000		
Netherlands	-1.527	-2.876	-0.967	-0.815	
p	0.950	**0.030**	1.000	1.000	
Spain/Brazil	-2.275	-3.576	-1.922	-1.601	-0.858
p	[illegible]	[illegible]	[illegible]	[illegible]	[illegible]

eFigure 18: General Assessment of Functioning disability subscale stratified by EU-GEI site

a. Box plots for General Assessment of Functioning (GAF) disability subscale score, stratified by EU-GEI site

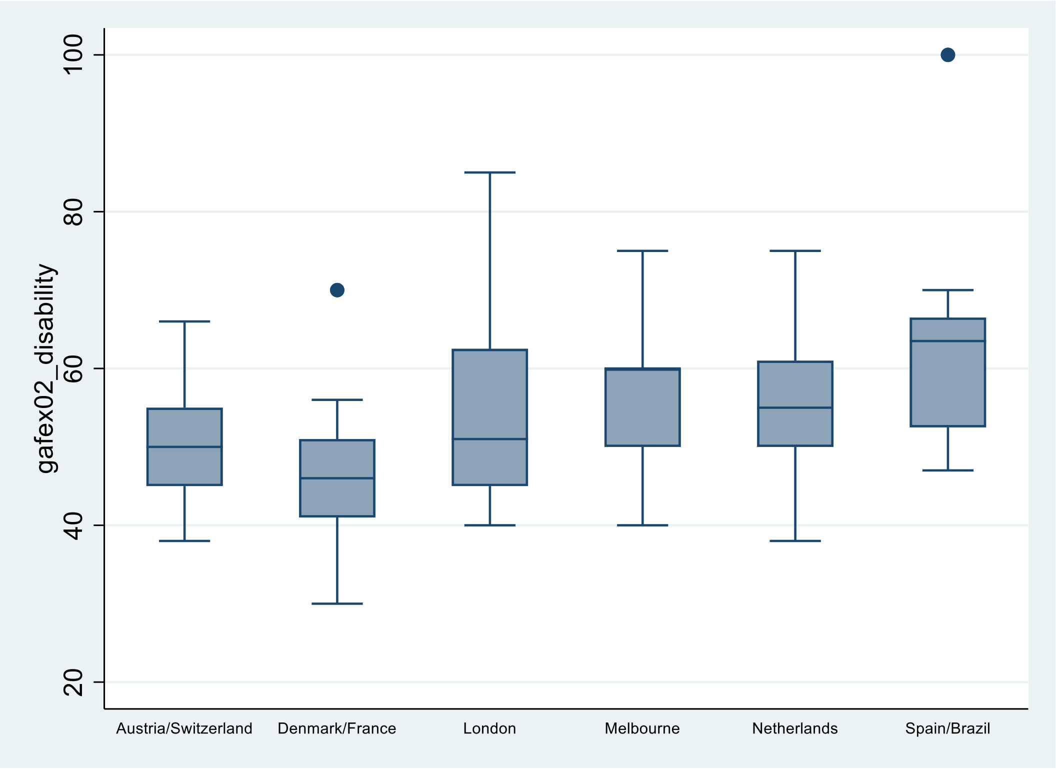

b. Kruskall-Wallis test

Chi-sq (5 d.f.): 15.838
p = 0.0073

c. Post-hoc Dunn's test pairwise comparisons with Bonferroni-corrected p values

	Austria/Switzerland	Denmark/France	London	Melbourne	Netherlands
Denmark/France	1.088				
p	1.000				
London	-0.974	-2.714			
p	1.000	0.050			
Melbourne	-0.929	-2.138	-0.201		
p	1.000	0.244	1.000		

© 2020 Mongan D et al. *JAMA Psychiatry.*

Netherlands	-0.853	-2.111	-0.072	0.108	
p	1.000	0.261	1.000	1.000	
Spain/Brazil	-2.366	-3.720	-2.014	-1.456	-1.611

eFigure 19: Scale for Assessment of Negative Symptoms (composite score) stratified by EU-GEI site

a. Box plots for Scale for Assessment of Negative Symptoms (SANS) total composite score, stratified by EU-GEI site

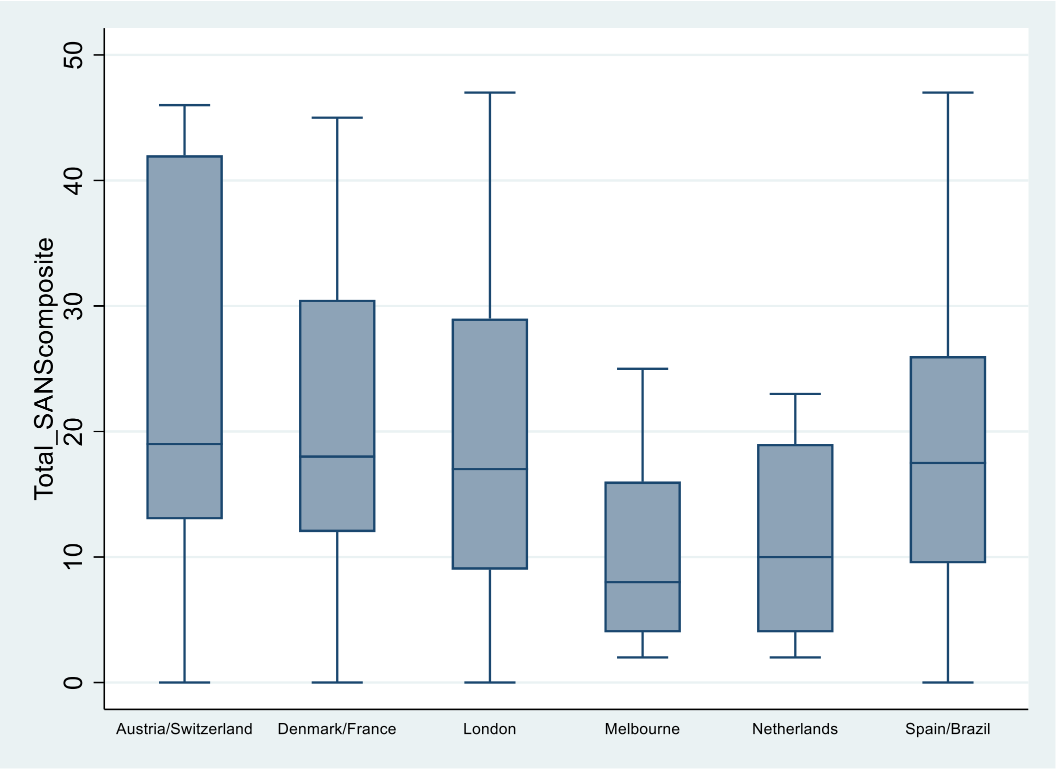

b. Kruskall-Wallis test

Chi-sq (5 d.f.): 9.030
p = 0.1079

© 2020 Mongan D et al. *JAMA Psychiatry.*

eFigure 20: Scale for Assessment of Negative Symptoms (global score) stratified by EU-GEI site

a. Box plots for Scale for Assessment of Negative Symptoms (SANS) total global score, stratified by EU-GEI site

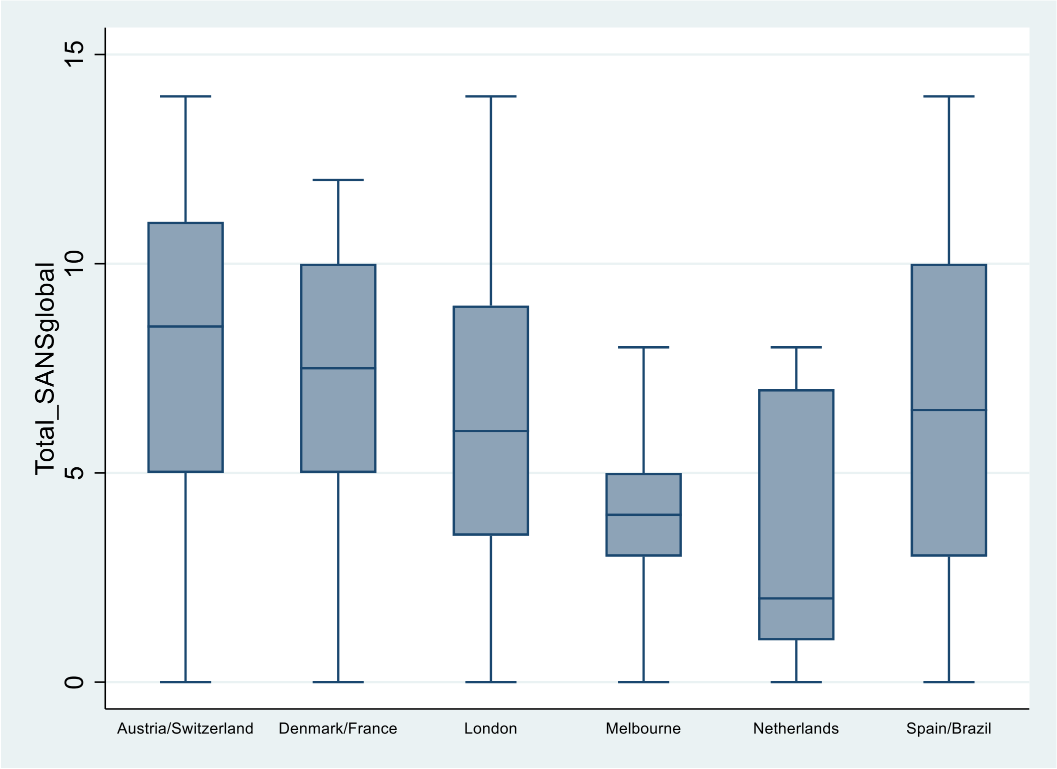

b. Kruskall-Wallis test

Chi-sq (5 d.f.): 11.823
p = 0.0373

c. Post-hoc Dunn's test pairwise comparisons with Bonferroni-corrected p values

	Austria/Switzerland	Denmark/France	London	Melbourne	Netherlands
Denmark/France	0.159				
p	1.000				
London	0.709	0.747			
p	1.000	1.000			
Melbourne	2.066	2.350	1.991		
p	0.291	0.141	0.349		
	2.194	2.540	2.195	0.071	

© 2020 Mongan D et al. *JAMA Psychiatry.*

Netherlands					
p	0.212	0.083	0.211	1.000	
Spain/Brazil	0.519	0.460	-0.075	-1.615	-1.739
p	1.000	1.000	1.000	0.797	0.615

eFigure 21: Brief Psychiatric Rating Scale score stratified by EU-GEI site

a. Box plots for Brief Psychiatric Rating Scale (BPRS) total score, stratified by EU-GEI site

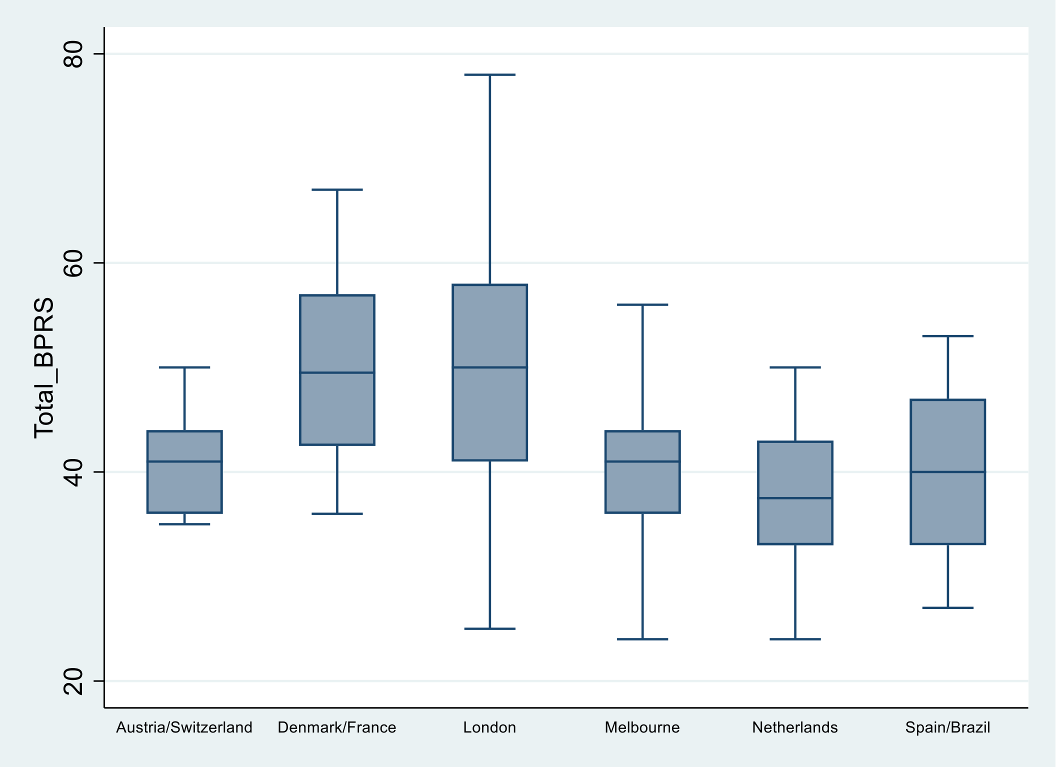

b. Kruskall-Wallis test

Chi-sq (5 d.f.): 28.083
p = 0.0001

c. Post-hoc Dunn's test pairwise comparisons with Bonferroni-corrected p values

	Austria/Switzerland	Denmark/France	London	Melbourne	Netherlands
Denmark/France	-2.426				
p	0.114				
London	-2.603	0.060			
p	0.069	1.000			
Melbourne	0.040	2.613	2.836		

© 2020 Mongan D et al. *JAMA Psychiatry.*

p	1.000	0.067	**0.034**		
Netherlands	0.674	3.434	3.762	0.662	
p	1.000	**0.005**	**0.001**	1.000	
Spain/Brazil	0.083	2.597	2.804	0.045	-0.602
p	1.000	0.071	**0.038**	1.000	1.000

eFigure 22: Montgomery-Asberg Depression Rating Scale score stratified by EU-GEI site

a. Box plots for Montgomery-Asberg Depression Rating Scale (MADRS) total score, stratified by EU-GEI site

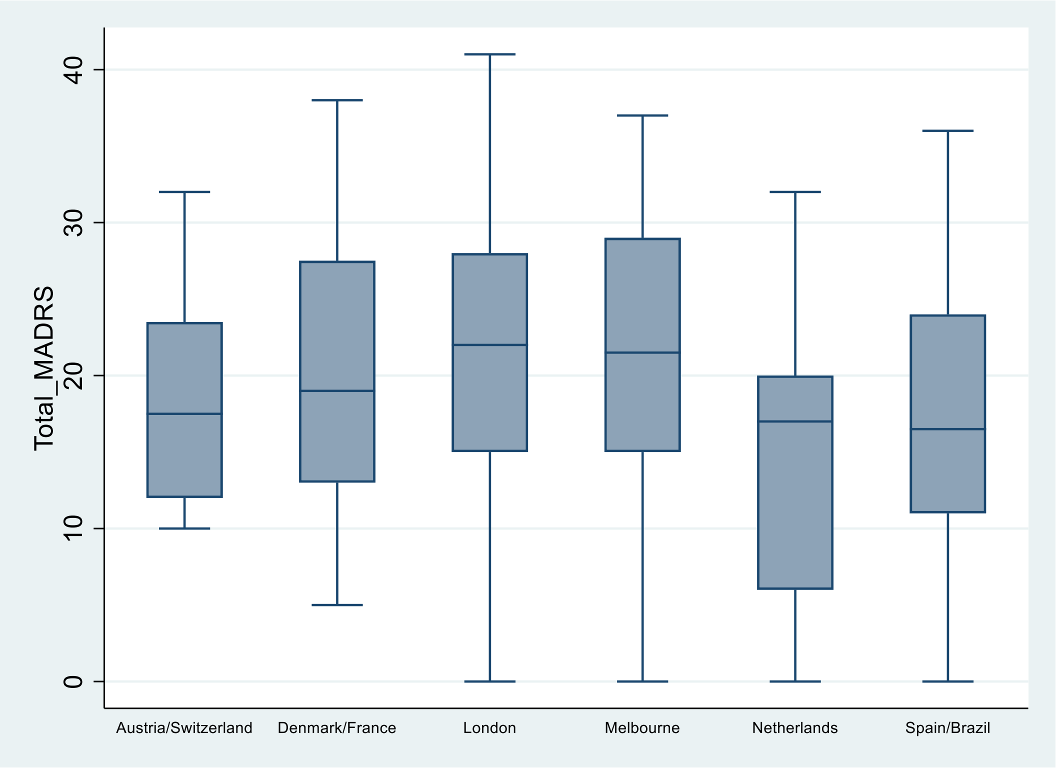

b. Kruskall-Wallis test

Chi-sq (5 d.f.): 5.398
p = 0.3693

© 2020 Mongan D et al. *JAMA Psychiatry.*

eFigure 23: Multi-class receiver-operating curves for site prediction based on 69 clinical features from Model 1b

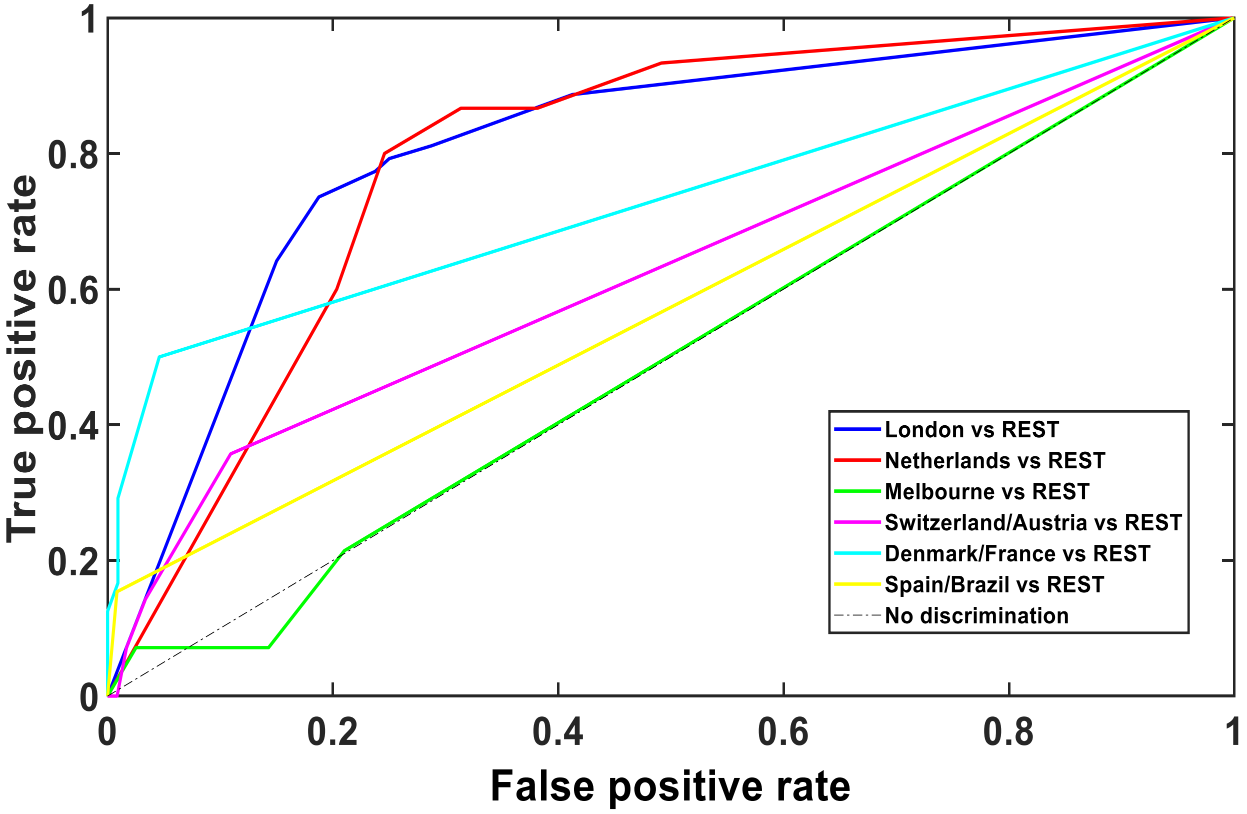

© 2020 Mongan D et al. *JAMA Psychiatry.*

eFigure 24: Multi-class receiver-operating curves for site prediction based on 166 proteomic features from Model 1c

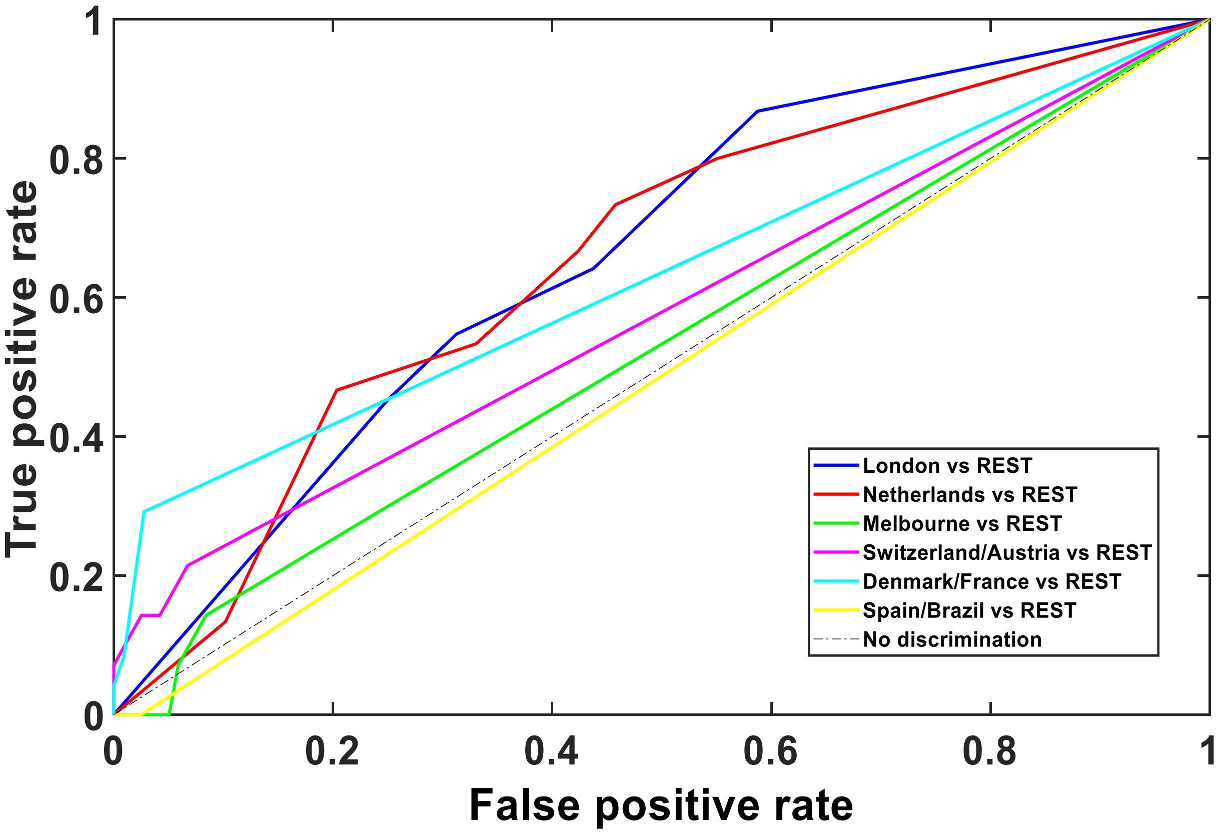

© 2020 Mongan D et al. *JAMA Psychiatry.*

eFigure 25: Illustration of the complement and coagulation pathways depicting the impact model of differentially expressed complement and coagulation proteins in CHR-T vs. CHR-NT

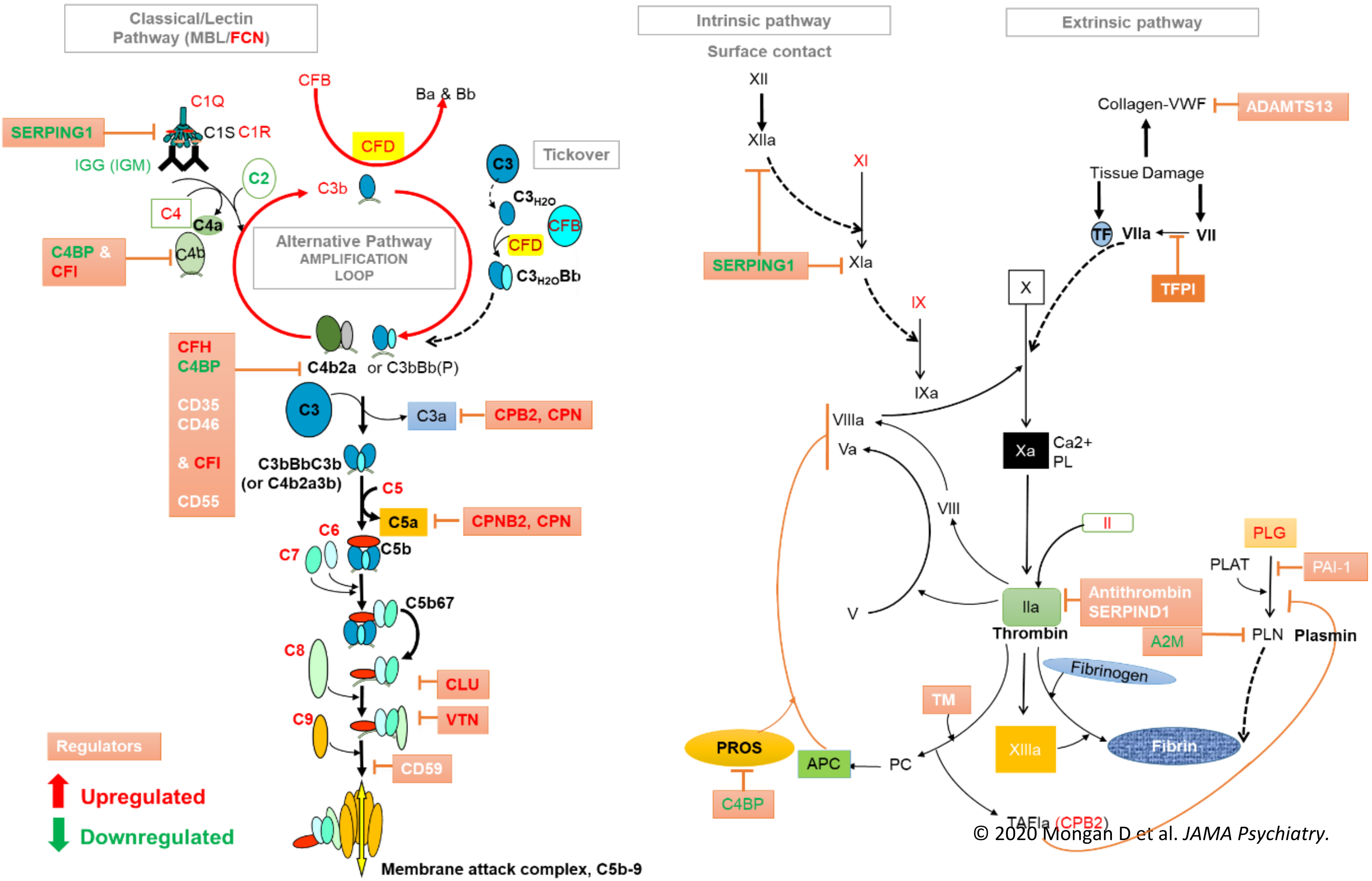

© 2020 Mongan D et al. *JAMA Psychiatry.*

Upregulated proteins shown in red, downregulated components shown in green font colour. Regulatory components are framed in light red boxes.
