# Supplementary material for: Development of Proteomic Prediction Models for Transition to Psychotic Disorder in the Clinical High-Risk State and Psychotic Experiences in Adolescence
Source: JAMA Psychiatry. 2020 Aug 26;78(1):77–90. doi: 10.1001/jamapsychiatry.2020.2459 (PMC7450406; doi:10.1001/jamapsychiatry.2020.2459)
Supplement: Supplement 2. — eAppendix. MS2-Determined Peptide Sequences Used to Match to Parent Proteins in the Human FASTA Database (Excel File) [file jamapsychiatry-e202459-s002.pdf]

Supplementary Online Content

Mongan D, Föcking M, Healy C, et al; European Network of National Schizophrenia Networks Studying Gene-Environment Interactions (EU-GEI) High Risk Study Group. Development of proteomic prediction models for transition to psychotic disorder in the clinical high-risk state and psychotic experiences in adolescence. *JAMA Psychiatry*. Published online August 26, 2020. doi:10.1001/jamapsychiatry.2020.2459

eAppendix.

This supplementary material has been provided by the authors to give readers additional information about their work.

© 2020 Mongan D et al. *JAMA Psychiatry.*

Sequence	N-term cleavage window	C-term cleavage window	Amino acid before	First amino acid	Second amino acid	Second last amino acid	Last amino acid	Amino acid after	A Count	R Count	N Count	D Count	C Count	Q Count	E Count	G Count	H Count	I Count	L Count	K Count	M Count	F Count	P Count	S Count	T Count	W Count	Y Count	V Count	U Count	O Count	Length	Missed cleavages	Mass	Proteins	Leading razor protein	Start position	End position	Unique (Groups)	Unique (Proteins)	Charges	PEP	Score	id	Protein group IDs	Mod. peptide IDs	Evidence IDs	MS/MS IDs	Best MS/MS	Oxidation (M) site IDs	MS/MS Count
AAAITSDILEALGR	EDDLSSFRAAAITSDI	DILEALGRDGHFTLFA	R	A	A	G	R	D	4	1	0	1	0	0	1	1	0	2	2	0	0	0	0	1	1	0	0	0	0	0	14	0	1399.767	sp|Q15063	sp|Q15063|PC	252	265	yes	yes	2	6.60E-32	134.75	1	464	1	14;15;16;17;1	2;3;4;5;6;7	19		21
AACLLPK	ECCQAADKAACLLPKL	KAACLLPKLDELRDEG	K	A	A	P	K	L	2	0	0	0	1	0	0	0	0	0	2	1	0	0	1	0	0	0	0	0	0	0	7	0	771.4313	sp|P02768	sp|P02768|ALI	199	205	yes	no	2	0.036669	91.093	5	11	5	784;785;786;	469;470	469		2
AADIEQQAVFAVFDENK	LDRRGIQRAADIEQQA	FAVFDENKSWYLEDNI	R	A	A	N	K	S	4	0	1	2	0	2	2	0	0	1	0	1	0	2	0	0	0	0	0	2	0	0	17	0	1893.911	sp|P12259	sp|P12259|FA	539	555	no	no	2	0.0009138	81.565	9	264;23	9	1136;1137;11	656;657	656		2
AAFTECCQAADK	LFFAKRYKAAFTECCQ	ECCQAADKAACLLPKL	K	A	A	D	K	A	4	0	0	1	2	1	1	0	0	0	0	1	0	1	0	0	1	0	0	0	0	0	12	0	1370.56	sp|P02768	sp|P02768|ALI	187	198	yes	no	2	2.26E-27	154.85	12	11	12	1301;1302;13	701;702;70	714		98
ACEPGVDYVYK	TLEERLDKACEPGVDY	PGVDYVYKTRLVKVQL	K	A	C	Y	K	T	1	0	0	1	1	0	1	1	0	0	0	1	0	0	1	0	0	0	2	2	0	0	11	0	1299.581	sp|P01024	sp|P01024|CO	1536	1546	no	no	2	0.0008667	83.998	31	97;24	31	4262;4263;42	1742;1743;	1764		30
ADDKETCFAEEGK	AFVEKCCKADDKETCF	TCFAEEGKKLVAASQA	K	A	D	G	K	K	2	0	0	2	1	0	3	1	0	0	0	2	0	1	0	0	1	0	0	0	0	0	13	1	1498.625	sp|P02768	sp|P02768|ALI	585	597	yes	no	2;3	1.50E-86	238.01	36	11	36	4923;4924;49	1966;1967;	1981		36
ADDKETCFAEEGKK	AFVEKCCKADDKETCF	CFAEEGKKLVAASQAA	K	A	D	K	K	L	2	0	0	2	1	0	3	1	0	0	0	3	0	1	0	0	1	0	0	0	0	0	14	2	1626.72	sp|P02768	sp|P02768|ALI	585	598	yes	no	2	0.053376	78.864	37	11	37	5190;5191	2002	2002		1
AEAESLYQSK	EDIAQKSKAEAESLYQ	AESLYQSKYEELQITA	K	A	E	S	K	Y	2	0	0	0	0	1	2	0	0	0	1	1	0	0	0	2	0	0	1	0	0	0	10	0	1124.535	sp|P04264	sp|P04264|K2	367	376	yes	no	2	0.03571	58.433	51	178	51	8761;8762;87	4046	4046		1
AEFAEVSK	RLSQRFPKAEFAEVSK	AEFAEVSKLVTDLTKV	K	A	E	S	K	L	2	0	0	0	0	0	2	0	0	0	0	1	0	1	0	1	0	0	0	1	0	0	8	0	879.4338	sp|P02768	sp|P02768|ALI	250	257	yes	no	2	0.020189	97.798	65	11	65	10521;10522;	4692;4693;	4705		63
AEFAEVSKLVTDLTK	RLSQRFPKAEFAEVSK	KLVTDLTKVHTECCHG	K	A	E	T	K	V	2	0	0	1	0	0	2	0	0	0	2	2	0	1	0	1	2	0	0	2	0	0	15	1	1649.888	sp|P02768	sp|P02768|ALI	250	264	yes	no	3	0.05102	35.757	66	11	66	10826	4755	4755		1
AEFVEVTK	RLSQKFPKAEFVEVTK	AEFVEVTKLVTDLTKV	K	A	E	T	K	L	1	0	0	0	0	0	2	0	0	0	0	1	0	1	0	0	1	0	0	2	0	0	8	0	921.4808	CON__P02	CON__P02769	249	256	yes	yes	2	0.064911	52.786	69	12	69	11334;11335	4889;4890	4889		2
AETECQNTEYQQLLDIK	EEQLQQIRAETECQNT	YQQLLDIKIRLENEIQ	R	A	E	I	K	I	1	0	1	1	1	3	3	0	0	1	2	1	0	0	0	0	2	0	1	0	0	0	17	0	2081.958	sp|P13645	sp|P13645|K1	423	439	yes	no	2	7.61E-73	153.34	81	15	83	13168;13169;	5805;5806	5805		2
AEVDDVIQVR	GILGPIIRAEVDDVIQ	VDDVIQVRFKNLASRP	R	A	E	V	R	F	1	1	0	2	0	1	1	0	0	1	0	0	0	0	0	0	0	0	0	3	0	0	10	0	1142.593	sp|P12259	sp|P12259|FA	1650	1659	no	no	2	0.001033	111.95	82	264;23	84	13172;13173;	5807	5807		1
AFLDCCEYITQLR	LQGDACVKAFLDCCEY	CEYITQLRQQHSRDGA	K	A	F	L	R	Q	1	1	0	1	2	1	1	0	0	1	2	0	0	1	0	0	1	0	1	0	0	0	13	0	1687.77	CON__Q2L	CON__Q2UVX4	721	733	yes	yes	2	3.43E-22	133.81	86	24	88	13662;13663;	5990;5991;	5990		5
AGALNSNDAFVLK	RAVEVLPKAGALNSND	SNDAFVLKTPSAAYLW	K	A	G	L	K	T	3	0	2	1	0	0	0	1	0	0	2	1	0	1	0	1	0	0	0	1	0	0	13	0	1318.688	sp|P06396	sp|P06396|GE	585	597	yes	no	2	3.07E-21	127.4	99	204	101	18423;18424;	8314;8315;	8322		163
AGKEPGLQIWR	VEHPEFLKAGKEPGLQ	EPGLQIWRVEKFDLVP	K	A	G	W	R	V	1	1	0	0	0	1	1	2	0	1	1	1	0	0	1	0	0	1	0	0	0	0	11	1	1253.688	sp|P06396	sp|P06396|GE	62	72	yes	no	3	2.67E-05	80.916	111	204	113	20608;20609;	9421;9422;	9423		4
AGLENTVAETECR	LQSQLSMKAGLENTVA	TVAETECRYALQLQQI	K	A	G	C	R	Y	2	1	1	0	1	0	3	1	0	0	1	0	0	0	0	0	2	0	0	1	0	0	13	0	1448.657	sp|P13646	sp|P13646|K1	343	355	yes	no	2	0.0003256	77.379	113	276	115	20678;20679;	9426	9426		1
AIGGGLSSVGGGSSTIK	GFSSSSGRAIGGGLSS	GGGSSTIKYTTTSSSS	R	A	I	I	K	Y	1	0	0	0	0	0	0	6	0	2	1	1	0	0	0	4	1	0	0	1	0	0	17	0	1446.768	sp|P02538	sp|P02538|K2	534	550	yes	no	2	2.81E-21	112.43	142	5	144	23653;23654	10488	10488		1
ALEEANADLEVK	ASYLDKVRALEEANAD	ANADLEVKIRDWYQRQ	R	A	L	V	K	I	3	0	1	1	0	0	3	0	0	0	2	1	0	0	0	0	0	0	0	1	0	0	12	0	1300.651	sp|P02533	sp|P02533|K1	135	146	no	no	2	0.063193	45.137	169	4;276;13	172	26461;26462;	11442	11442		1
ALEESNYELEGK	ASYLDKVRALEESNYE	SNYELEGKIKEWYEKH	R	A	L	G	K	I	1	0	1	0	0	0	4	1	0	0	2	1	0	0	0	1	0	0	1	0	0	0	12	0	1380.641	sp|P13645	sp|P13645|K1	166	177	yes	no	2	3.13E-10	136.63	170	15	173	26465;26466;	11443;114	11443		16
ALLEMQQTK	QEAPLEWKALLEMQQT	LLEMQQTKMVLETFGG	K	A	L	T	K	M	1	0	0	0	0	2	1	0	0	0	2	1	1	0	0	0	1	0	0	0	0	0	9	0	1060.559	CON__P02	CON__P02672	253	261	yes	yes	2	0.0032584	95.909	187	8	190	30170;30171;	13331	13331		1
APSTYGGGLSVSSSR	VLAGGSCRAPSTYGGG	GLSVSSSRFSSGGACG	R	A	P	S	R	F	1	1	0	0	0	0	0	3	0	0	1	0	0	0	1	5	1	0	1	1	0	0	15	0	1424.69	sp|P02533	sp|P02533|K1	42	56	yes	no	2	0.0031687	58.676	245	4	251	39281;39282;	17257	17257		1
APVLSDSSCK	PSLLQCLKAPVLSDSS	VLSDSSCKSSYPGQIT	K	A	P	C	K	S	1	0	0	1	1	0	0	0	0	0	1	1	0	0	1	3	0	0	0	1	0	0	10	0	1062.502	CON__P00	CON__P00761	148	157	yes	yes	2	0.0020818	88.948	248	1	254	39503;39504;	17296;172	17296		3
AQGYSGLSVK	YWDTNPTRAQGYSGLS	GYSGLSVKVVNSTTGP	R	A	Q	V	K	V	1	0	0	0	0	1	0	2	0	0	1	1	0	0	0	2	0	0	1	1	0	0	10	0	1008.524	sp|P07996	sp|P07996|TSI	1055	1064	yes	no	2	0.009768	73.233	252	223	258	39836;39837;	17336;173	17336		4
AQPVQVAEGSEPDGFWEALGGK	QELLRVLRAQPVQVAE	FWEALGGKAAYRTSPR	R	A	Q	G	K	A	3	0	0	1	0	2	3	4	0	0	1	1	0	1	2	1	0	1	0	2	0	0	22	0	2271.081	sp|P06396	sp|P06396|GE	627	648	yes	yes	2;3	0	246.96	260	204	266	43422;43423;	21035;210	21526		582
AQYEDIAQK	DSIIAEVKAQYEDIAQ	QYEDIAQKSKAEAESL	K	A	Q	Q	K	S	2	0	0	1	0	2	1	0	0	1	0	1	0	0	0	0	0	0	1	0	0	0	9	0	1064.514	sp|P04264	sp|P04264|K2	356	364	yes	yes	2	0.052609	57.047	265	178	271	44528;44529;	21826	21826		1
AQYEEIANR	DSIIAEVKAQYEEIAN	QYEEIANRSRTEAESW	K	A	Q	N	R	S	2	1	1	0	0	1	2	0	0	1	0	0	0	0	0	0	0	0	1	0	0	0	9	0	1092.52	sp|P13647	sp|P13647|K2	344	352	yes	no	2	0.029369	66.073	266	16	272	44558;44559;	21827	21827		1
ASLENSLEETK	LQSQLSMKASLENSLE	ENSLEETKGRYCMQLA	K	A	S	T	K	G	1	0	1	0	0	0	3	0	0	0	2	1	0	0	0	2	1	0	0	0	0	0	11	0	1219.593	sp|P02533	sp|P02533|K1	353	363	no	no	2	6.63E-05	99.815	281	4;13	287	46153;46154;	22458	22458		1
ATDAEADVASLNR	KLEQAEKKATDAEADV	ADVASLNRRIQLVEEE	K	A	T	N	R	R	4	1	1	2	0	0	1	0	0	0	1	0	0	0	0	1	1	0	0	1	0	0	13	0	1331.632	sp|P07951	sp|P07951|TPI	78	90	no	no	2	1.82E-07	92.611	293	222;241	299	47508;47509;	22942;229	22943		2

© 2020 Mongan D et al. *JAMA Psychiatry.*

VLDELTLTK	ADINGLRRVLDELTLT	LDELTLTKADLEMQIE	R	V	L	T	K	A	0	0	0	1	[illegible]
VLVLDTDYK	IDALNENKVLVLDTDY	LVLDTDYKKYLLFCME	K	V	L	Y	K	K	0	0	0	2	[illegible]
VPFDAATLHTSTAMAAQHGMDDDGTGQK	SHIANVERVPFDAATL	DDDGTGQKQIWRIEGS	R	V	P	Q	K	Q	5	0	0	4	[illegible]
VPMMNSK	HFKNSVIKVPMMNSKK	KVPMMNSKKYPVAHFI	K	V	P	S	K	K	0	0	1	0	[illegible]
VPQVSTPTLVEVSR	LLVRYTKKVPQVSTPT	PTLVEVSRNLGKVGSK	K	V	P	S	R	N	0	1	0	0	[illegible]
VPVDPATYGQFYGGDSYIILYNYR	WRIEGSNKVPVDPATY	YIILYNYRHGGRQGQI	K	V	P	Y	R	H	1	1	1	2	[illegible]
VQALEEANNDLENK	RLASYLDKVQALEEAN	ANNDLENKIQDWYDKK	K	V	Q	N	K	I	2	0	3	1	[illegible]
VQELQDKLSPLAQELR	FREGARQKVQELQDKL	SPLAQELRDRARAHVE	K	V	Q	L	R	D	1	1	0	1	[illegible]
VRPQQLVK	GVYELLLKVRPQQLVK	VRPQQLVKHLQMDIHI	K	V	R	V	K	H	0	1	0	0	[illegible]
VSILAAIDEASK	LPVLESLKVSILAAID	AAIDEASKKLNAQ___	K	V	S	S	K	K	3	0	0	1	[illegible]
VSILAAIDEASKK	LPVLESLKVSILAAID	AIDEASKKLNAQ____	K	V	S	K	K	L	3	0	0	1	[illegible]
VSLAGACGVGGYGSR	GGGGGFGRVSLAGACG	GVGGYGSRSLYNLGGS	R	V	S	S	R	S	2	1	0	0	[illegible]
VTFELTYEELLK	VNVAAGSKVTFELTYE	LTYEELLKRHKGKYEM	K	V	T	L	K	R	0	0	0	0	[illegible]
VTMQNLNDR	GLLSGNEKVTMQNLND	TMQNLNDRLASYLDKV	K	V	T	D	R	L	0	1	2	1	[illegible]
WELLQQVDTSTR	QNQVLQTKWELLQQVD	QQVDTSTRTHNLEPYF	K	W	E	T	R	T	0	1	0	1	[illegible]
WNLLQQQTTTTSSK	QNKVLETKWNLLQQQT	QTTTTSSKNLEPLFET	K	W	N	S	K	N	0	0	1	0	[illegible]
WTLLQEQGTK	QNKVLDTKWTLLQEQG	LLQEQGTKTVRQNLEP	K	W	T	T	K	T	0	0	0	0	[illegible]
YDGCCEGDVVQCIR	LLEDVSSKYDGCCEGD	GDVVQCIRGRSKVMSH	K	Y	D	I	R	G	0	1	0	2	[illegible]
YEELQITAGR	AESLYQSKYEELQITA	ELQITAGRHGDSVRNS	K	Y	E	G	R	H	1	1	0	0	[illegible]
YEELQQTAGR	AESWYQTKYEELQQTA	ELQQTAGRHGDDLRNT	K	Y	E	G	R	H	1	1	0	0	[illegible]
YEELQVTAGR	AESWYQTKYEELQVTA	ELQVTAGRHGDDLRNT	K	Y	E	G	R	H	1	1	0	0	[illegible]
YENELALR	AADDFRLKYENELALR	YENELALRQSVEADIN	K	Y	E	L	R	Q	1	1	1	0	[illegible]
YENEVALR	AADDFRLKYENEVALR	YENEVALRQSVEADIN	K	Y	E	L	R	Q	1	1	1	0	[illegible]
YICENQDSISSK	DDRADLAKYICENQDS	NQDSISSKLKECCEKP	K	Y	I	S	K	L	0	0	1	1	[illegible]
YIETDPANR	EALTSAKRYIETDPAN	IETDPANRDRRTPITV	R	Y	I	N	R	D	1	1	1	1	[illegible]
YIYGKPVQGVAYVR	MQLDIQARYIYGKPVQ	VQGVAYVRFGLLDEDG	R	Y	I	V	R	F	1	1	0	0	[illegible]
YLDGLTAER	GYIDSLKRYLDGLTAE	LDGLTAERTSQNSELN	R	Y	L	E	R	T	1	1	0	1	[illegible]
YLGYLEQLLR	KEDVPSERYLGYLEQL	GYLEQLLRLKKYKVPQ	R	Y	L	L	R	L	0	1	0	0	[illegible]
YLYEIAR	NEETFLKKYLYEIARR	KYLYEIARRHPYFYAP	K	Y	L	A	R	R	1	1	0	0	[illegible]
YVLPNFEVK	STQFEVKKYVLPNFEV	VLPNFEVKITPGKPYI	K	Y	V	V	K	I	0	0	1	0	[illegible]
AAAATGTIFTFR	EVDESGTRAAAATGTI	TGTIFTFRSARLNSQR	R	A	A	F	R	S	4	1	0	0	[illegible]
AAANQMR	LHFTKDVKAAANQMRN	KAAANQMRNFLVRASC	K	A	A	M	R	N	3	1	1	0	[illegible]
AAATGPSFWLGNETLK	________________	WLGNETLKVPLALFAL	M	A	A	L	K	V	3	0	1	0	[illegible]
[illegible]	[illegible]	[illegible]	[illegible]	[illegible]	[illegible]	[illegible]	[illegible]	[illegible]	[illegible]	[illegible]	[illegible]	[illegible]	[illegible]

© 2020 Mongan D et al. *JAMA Psychiatry*.

[illegible]

© 2020 Mongan D et al. *JAMA Psychiatry*.

DLFNAIATGK	EDPDYGIRDLFNAIAT	FNAIATGKYPSWTFYI	R	D	L	G	K	Y	2	[illegible]
DLGEELEALK	NKAEKQKRDLGEELEA	GEELEALKTELEDTLD	R	D	L	L	K	T	1	[illegible]
DLHLSDVFLK	LNFTTKAKDLHLSDVF	HLSDVFLKALNHLPLE	K	D	L	L	K	A	0	[illegible]
DLIPNGEYFFR	NKLSWTVKDLIPNGEY	PNGEYFFRVKAVNKVG	K	D	L	F	R	V	0	[illegible]
DLLGIYEK	RRWYVPVKDLLGIYEK	DLLGIYEKLYGRKVIT	K	D	L	E	K	L	0	[illegible]
DLLLDPAWEK	QPEEDWDRDLLLDPAW	LLDPAWEKQQRKTFTA	R	D	L	E	K	Q	1	[illegible]
DLLLPQPDLR	NKLQVLGKDLLLPQPD	LLPQPDLRYLFLNGNK	K	D	L	L	R	Y	0	[illegible]
DLLPASLGSYYR	FLDPFVLRDLLPASLG	ASLGSYYRYTGSLTTP	R	D	L	Y	R	Y	1	[illegible]
DLLQTVEDQYK	EKQDNSIKDLLQTVED	QTVEDQYKQLNQQHSQ	K	D	L	Y	K	Q	0	[illegible]
DLMVLNDVYR	AEPTWNLKDLMVLNDV	MVLNDVYRVMAVDALA	K	D	L	Y	R	V	0	[illegible]
DLQFSEIR	SPVLESPRDLQFSEIR	DLQFSEIRETSAKVNW	R	D	L	I	R	E	0	[illegible]
DLQFVEVTDVK	SDTVPSPRDLQFVEVT	FVEVTDVKVTIMWTPP	R	D	L	V	K	V	0	[illegible]
DLSEAHFAPC	EVVGLDLRDLSEAHFA	SEAHFAPC_________	R	D	L	P	C	-	2	[illegible]
DLTDYLMK	LRLDLAGRDLTDYLMK	DLTDYLMKILTERGYS	R	D	L	M	K	I	0	[illegible]
DLTNFPDNVDQQEEEQGHCPR	VYNLLTVKDLTNFPDN	EEQGHCPRPFFIHNGA	K	D	L	P	R	P	0	[illegible]
DLVGELGTALR	SKDVGPHRDLVGELGT	GELGTALRKRNIRYGL	R	D	L	L	R	K	1	[illegible]
DLYANTVLSGGTTMYPGIADR	KCDVDIRKDLYANTVL	MYPGIADRMQKEITAL	K	D	L	D	R	M	2	[illegible]
DLYSGLIGPLIVCR	YSTVDQVKDLYSGLIG	IGPLIVCRRPYLKVFN	K	D	L	C	R	R	0	[illegible]
DMPASEDLQDLQK	RQGHPENRDMPASEDL	EDLQDLQKKLCRKRCI	R	D	M	Q	K	K	1	[illegible]
DMQWHQR	VQSVRRIRDMQWHQRG	RDMQWHQRGAFHPSN_	R	D	M	Q	R	G	0	[illegible]
DMTEVISSLENANYK	SVLNDNSRDMTEVISS	SLENANYKDHENGTGT	R	D	M	Y	K	D	1	[illegible]
DMYSFLEDMGLK	PVSSTNEKDMYSFLED	FLEDMGLKAFTNSKIR	K	D	M	L	K	A	0	[illegible]
DNCCILDER	CVAYVATRDNCCILDE	NCCILDERFGSYCPTT	R	D	N	E	R	F	0	[illegible]
DNDGWLTSDPR	MFFSTYDRDNDGWLTS	GWLTSDPRKQCSKEDG	R	D	N	P	R	K	0	[illegible]
DNDLISLPK	KLQILSLRDNDLISLP	NDLISLPKEIGELTQL	R	D	N	P	K	E	0	[illegible]
DNEDFQESNR	SEPEKVDKDNEDFQES	EDFQESNRMYSVNGYT	K	D	N	N	R	M	0	[illegible]
DNELLVYK	FSYNTQGRDNELLVYK	DNELLVYKERVGEYSL	R	D	N	Y	K	E	0	[illegible]
DNENVVNEYSSELEK	QKRQKQVKDNENVVNE	EYSSELEKHQLYIDET	K	D	N	E	K	H	0	[illegible]
DNIQGITKPAIR	KRHRKVLRDNIQGITK	GITKPAIRRLARRGGV	R	D	N	I	R	R	1	[illegible]
DNPMYYCNK	FRYENCPKDNPMYYCN	NPMYYCNKKTSCRSCA	K	D	N	N	K	K	0	[illegible]
DNSQVNAVTVLTLLDK	GNTEEAIRDNSQVNAV	TVLTLLDKLVNMLDAV	R	D	N	D	K	L	1	[illegible]
DNSVHWER	LNEEAVKKDNSVHWER	DNSVHWERPQKPKAPV	K	D	N	E	R	P	0	[illegible]
DNVFDGLVR	QASFQGLKDNVFDGLV	NVFDGLVRVTQEFHMK	K	D	N	V	R	V	0	[illegible]
[illegible]	[illegible]	[illegible]	[illegible]	[illegible]	[illegible]	[illegible]	[illegible]	[illegible]	[illegible]	[illegible]

© 2020 Mongan D et al. *JAMA Psychiatry.*

ELISLVEDVSSNYDGCCEGDVVQCIR	KFPKIEFKELISLVED	GDVVQCIRDTSKVMNH	K	E	L	I	R	D	0	1	1	3	3	1	3	2	0	2	2	0	0	0	0	3	0	0	1	4	0	0	26	0	3015.326	sp|P43652	sp|P43652|AF	254	279	yes	yes	2;3;4	1.07E-77	172.24	888	362	906	140838;1408	66517;665	66716	210
ELISNASDALDK	KNKEIFLRELISNASD	NASDALDKIRLISLTD	R	E	L	D	K	I	2	0	1	2	0	0	1	0	0	1	2	1	0	0	0	2	0	0	0	0	0	0	12	0	1274.635	sp|P08238	sp|P14625|EN	103	114	no	no	2	5.26E-09	112.36	889	284;226	907	141445;1414	66735;667	66738	24
ELLALIQLER	PATDPLQRELLALIQL	LALIQLEREFQRCCRQ	R	E	L	E	R	E	1	1	0	0	0	1	2	0	0	1	4	0	0	0	0	0	0	0	0	0	0	0	10	0	1196.713	sp|Q16610	sp|Q16610|EC	334	343	yes	yes	2	1.00E-49	189.7	890	475	908	141543;1415	66759;667	66874	149
ELLDTVTAPQK	PDIHGTYKELLDTVTA	DTVTAPQKNLKSASRI	K	E	L	Q	K	N	1	0	0	1	0	1	1	0	0	0	2	1	0	0	1	0	2	0	0	1	0	0	11	0	1213.655	sp|P36955	sp|P36955|PE	124	134	yes	yes	2	4.03E-06	111.39	891	353	909	141793;1417	66908;669	66919	165
ELLESYIDGR	SLEDKTERELLESYID	LESYIDGRIVEGSDAE	R	E	L	G	R	I	0	1	0	1	0	0	2	1	0	1	2	0	0	0	0	1	0	0	1	0	0	0	10	0	1193.593	sp|P00734	sp|P00734|TH	354	363	yes	yes	2	1.02E-39	182.53	892	80	910	141990;1419	67074;670	67130	634
ELLETVVNR	SLVRGRMKELLETVVN	LLETVVNRTRDGWQWF	K	E	L	N	R	T	0	1	1	0	0	0	2	0	0	0	2	0	0	0	0	0	1	0	0	2	0	0	9	0	1071.592	sp|P55056	sp|P55056|AP	56	64	yes	yes	2	4.07E-14	157.58	893	387	911	142838;1428	67714;677	67722	145
ELLLQPVTISR	EVEVRSSKELLLQPVT	LQPVTISRNEKEKVLI	K	E	L	S	R	N	0	1	0	0	0	1	1	0	0	1	3	0	0	0	1	1	1	0	0	1	0	0	11	0	1267.75	sp|P59998	sp|P59998|AR	45	55	yes	yes	2	1.66E-22	140.88	894	395	912	143010;1430	67860;678	67878	47
ELLPVLISAMK	VNSMNTVKELLPVLIS	PVLISAMKIFVTTKNS	K	E	L	M	K	I	1	0	0	0	0	0	1	0	0	1	3	1	1	0	1	1	0	0	0	1	0	0	11	0	1212.715	sp|P18206	sp|P18206|VI	200	210	yes	yes	2	0.0056445	70.908	895	300	913	143094;1430	67907;679	67907	22
ELLSQGATLSGWYHLCLPEGR	QEGPRNCRELLSQGAT	HLCLPEGRALPVFCDM	R	E	L	G	R	A	1	1	0	0	1	1	2	3	1	0	5	0	0	0	1	2	1	1	1	0	0	0	21	0	2386.174	sp|O75636	sp|O75636|FC	95	115	yes	yes	3	0.0005085	49.188	896	63	914	143180;1431	67929;679	67929	2
ELLTTMGDR	TIQEDYLRELLTTMGD	LLTTMGDRFTDEEVDE	R	E	L	D	R	F	0	1	0	1	0	0	1	1	0	0	2	0	1	0	0	0	2	0	0	0	0	0	9	0	1034.507	sp|P19105	sp|P19105|ML	124	132	yes	no	2	0.035051	62.546	897	50	915	143216;1432	67931	67931	1
ELNELGSK	GKLDVDWRELNELGSK	ELNELGSKKDGERHAF	R	E	L	S	K	K	0	0	1	0	0	0	2	1	0	0	2	1	0	0	0	1	0	0	0	0	0	0	8	0	888.4553	sp|P06681	sp|P06681|CO	422	429	yes	yes	2	0.019921	98.904	898	205	916	143232;1432	67932;679	67933	2
ELNQAGQETLVTGWGYHSSR	PDSGLAERELNQAGQE	GWGYHSSREKEAKRNR	R	E	L	S	R	E	1	1	1	0	0	2	2	3	1	0	2	0	0	0	0	2	2	1	1	1	0	0	20	0	2232.056	sp|P04070	sp|P04070|PR	329	348	yes	yes	3	0.04299	24.875	899	171	917	143284;1432	67934	67934	1
ELPEHTVK	ASEDCMAKELPEHTVK	ELPEHTVKLCDNLSTK	K	E	L	V	K	L	0	0	0	0	0	0	2	0	1	0	1	1	0	0	1	0	1	0	0	1	0	0	8	0	951.5026	sp|P02774	sp|P02774|VT	277	284	yes	yes	3	0.0050121	69.825	900	159	918	143344;1433	67935;679	67937	14
ELPSLQHPNEQK	QEAVPLQKELPSLQHP	LQHPNEQKEGTPAPFG	K	E	L	Q	K	E	0	0	1	0	0	2	2	0	1	0	2	1	0	0	2	1	0	0	0	0	0	0	12	0	1418.715	sp|Q16610	sp|Q16610|EC	116	127	yes	yes	2;3	9.94E-24	148.56	901	475	919	143432;1434	67949;679	67950	8
ELQAAQAR	ETRARLSKELQAAQAR	ELQAAQARLGADMEDV	K	E	L	A	R	L	3	1	0	0	0	2	1	0	0	0	1	0	0	0	0	0	0	0	0	0	0	0	8	0	885.4668	sp|P02649	sp|P02649|AP	114	121	yes	no	2	0.011049	111.69	902	137	920	143480;1434	67957;679	67957	6
ELQAHGADELLK	SISLKFYKELQAHGAD	HGADELLKRVYGSFLV	K	E	L	L	K	R	2	0	0	1	0	1	2	1	1	0	3	1	0	0	0	0	0	0	0	0	0	0	12	0	1322.683	sp|O15144	sp|O15144|AR	66	77	yes	yes	2	0.0027057	66.621	903	52	921	143552;1435	67963;679	67963	2
ELQETNAALQDVR	DLGPQMLRELQETNAA	NAALQDVRELLRQQVR	R	E	L	V	R	E	2	1	1	1	0	2	2	0	0	0	2	0	0	0	0	0	1	0	0	1	0	0	13	0	1485.742	sp|P49747	sp|P49747|CO	37	49	yes	no	2	0.0001433	80.979	904	373	922	143582	67965	67965	1
ELRPWCFTTDPNK	NYCRNPDRELRPWCFT	CFTTDPNKRWELCDIP	R	E	L	N	K	R	0	1	1	1	1	0	1	0	0	0	1	1	0	1	2	0	2	1	0	0	0	0	13	1	1662.782	sp|P00747	sp|P00747|PL	240	252	yes	yes	3	8.55E-11	82.705	905	87	923	143583;1435	67966;679	67968	13
ELSDFISYLQR	PKKYEGGRELSDFISY	DFISYLQREATNPPVI	R	E	L	Q	R	E	0	1	0	1	0	1	1	0	0	1	2	0	0	1	0	2	0	0	1	0	0	0	11	0	1369.688	sp|P30101	sp|P30101|PD	472	482	yes	yes	2	9.38E-08	116.05	906	340	924	143702;1437	67979;679	67980	38
ELSDIAHR	ALTPEQKKELSDIAHR	ELSDIAHRIVAPGKGI	K	E	L	H	R	I	1	1	0	1	0	0	1	0	1	1	1	0	0	0	0	1	0	0	0	0	0	0	8	0	939.4774	sp|P04075	sp|P04075|AL	15	22	yes	yes	2	0.0006531	94.662	907	172	925	143776;1437	68017;680	68018	3
ELSEALGQIFDSQR	THTLDLSRELSEALGQ	GQIFDSQRGCDLSISV	R	E	L	Q	R	G	1	1	0	1	0	2	2	1	0	1	2	0	0	1	0	2	0	0	0	0	0	0	14	0	1591.784	sp|Q08380	sp|Q08380|LG	138	151	yes	yes	2;3	1.29E-110	202.65	908	440	926	143796;1437	68020;680	68039	38
ELSHLPSLYDYSAYR	QLAEPFWKELSHLPSL	LYDYSAYRRLIDQYGT	K	E	L	Y	R	R	1	1	0	1	0	0	1	0	1	0	3	0	0	0	1	3	0	0	3	0	0	0	15	0	1812.868	sp|P10643	sp|P10643|CO	279	293	yes	yes	2;3	1.42E-86	141.46	909	252	927	143923;1439	68060;680	68166	130
ELSSFIDK	AKGPLLKKELSSFIDK	ELSSFIDKGQELCADY	K	E	L	D	K	G	0	0	0	1	0	0	1	0	0	1	1	1	0	1	0	2	0	0	0	0	0	0	8	0	937.4757	sp|P02774	sp|P02774|VT	395	402	yes	yes	2	0.015358	104.21	910	159	928	144094;1440	68193;681	68208	59
ELSYYSLEDLNNK	FDSETAVKELSYYSLE	SLEDLNNKYLIAVTV	K	E	L	N	K	Y	0	0	2	1	0	0	2	0	0	0	3	1	0	0	0	2	0	0	2	0	0	0	13	0	1586.746	sp|P01031	sp|P01031|CO	309	321	yes	yes	2;3	3.65E-128	215.06	911	98	929	144679;1446	68252;682	68460	233
ELTLEDLK	VLAYSRLKELTLEDLK	ELTLEDLKITGTMPPL	K	E	L	L	K	I	0	0	0	1	0	0	2	0	0	0	3	1	0	0	0	0	1	0	0	0	0	0	8	0	959.5175	sp|P08571	sp|P08571|CD	120	127	yes	yes	2	3.09E-06	128.57	912	232	930	144999;1450	68487;684	68610	124
ELTPQVVSAAR	QASVKTARELTPQVVS	PQVVSAARILLRNPGN	R	E	L	A	R	I	2	1	0	0	0	1	1	0	0	0	1	0	0	0	1	1	1	0	0	2	0	0	11	0	1169.64	sp|P18206	sp|P18206|VI	670	680	yes	yes	2	5.56E-06	110.79	913	300	931	145164;1451	68611;686	68622	56
ELVLAGNR	FRGLGSLRELVLAGNR	ELVLAGNRLAYLQPAL	R	E	L	N	R	L	1	1	1	0	0	0	1	1	0	0	2	0	0	0	0	0	0	0	0	1	0	0	8	0	870.4923	sp|P35858	sp|P35858|AL	198	205	yes	yes	2	1.69E-05	144.29	915	351	933	145329;1453	68672;686	68673	93
ELVNNLAEIYGR	FGKDNKKKELVNNLAE	NLAEIYGRIEREHQIS	K	E	L	G	R	I	1	1	2	0	0	0	2	1	0	1	2	0	0	0	0	0	0	0	1	1	0	0	12	0	1389.725	sp|Q9NZN	sp|Q9NZN3|EH	330	341	yes	yes	2	4.90E-10	114.5	916	539	934	145499;1455	68765;687	68769	9
ELWILNR	RAALACSKELWILNRY	KELWILNRYLSYTLNP	K	E	L	N	R	Y	0	1	1	0	0	0	1	0	0	1	2	0	0	0	0	0	0	1	0	0	0	0	7	0	942.5287	sp|P15144	sp|P15144|AM	837	843	yes	yes	2	0.007259	115.83	917	285	935	145589;1455	68775;687	68792	25
EMECAGTYDGSIDACK	YGNRFYEKEMECAGTY	DGSIDACKGDSGGPLV	K	E	M	C	K	G	2	0	0	2	2	0	2	2	0	1	0	1	1	0	0	1	1	0	1	0	0	0	16	0	1805.691	sp|P05156	sp|P05156|CF	507	522	yes	yes	2	2.35E-86	174.42	918	192	936	145754;1457	68800;688	68801	80
EMEDTLNHLK	TEYGPCRREMEDTLNH	EDTLNHLKFLNVLSPR	R	E	M	L	K	F	0	0	1	1	0	0	2	0	1	0	2	1	1	0	0	0	1	0	0	0	0	0	10	0	1228.576	sp|P17936	sp|P17936|IB	216	225	yes	yes	2;3	1.61E-06	113.5	919	297	937	145886;1458	68880;688	68881	3
EMSGSPASGIPVK	FLLQALVREMSGSPAS	PASGIPVKVSATVSSP	R	E	M	V	K	V	1	0	0	0	0	0	1	2	0	1	0	1	1	0	2	3	0	0	0	1	0	0	13	0	1258.623	sp|P0C0L4	sp|P0C0L4|CO	392	404	no	no	2	2.04E-06	90.827	920	245;246	938	145969;1459	68883;688	68890	84
ENADSLQASLR	QRMERVLRENADSLQA	DSLQASLRPHADELKA	R	E	N	L	R	P	2	1	1	1	0	1	1	0	0	0	2	0	0	0	0	2	0	0	0	0	0	0	11	0	1202.589	sp|P06727	sp|P06727|AP	170	180	yes	yes	2	2.60E-32	168.26	921	206	939	146113;1461	68967;689	69000	162
ENADSLQASLRPHADELK	QRMERVLRENADSLQA	RPHADELKAKIDQNVE	R	E	N	L	K	A	3	1	1	2	0	1	2	0	1	0	3	1	0	0	1	2	0	0	0	0	0	0	18	1	1992.987	sp|P06727	sp|P06727|AP	170	187	yes	yes	2;3;4	7.57E-100	177.88	922	206	940	146311;1463	69131;691	69137	58
ENEGFTVTAEGK	LLRSEETKENEGFTVT	FTVTAEGKGQGTLSVV	K	E	N	G	K	G	1	0	1	0	0	0	3	2	0	0	0	1	0	1	0	0	2	0	0	1	0	0	12	0	1280.589	sp|P01024	sp|P01024|CO	1326	1337	yes	yes	2	0.000117	101.38	923	97	941	146521;1465	69189;691	69203	63
ENFAGEATLQR	DIWNLEVKENFAGEAT	AGEATLQRIYSLWEHS	K	E	N	Q	R	I	2	1	1	0	0	1	2	1	0	0	1	0	0	1	0	0	1	0	0	0	0	0	11	0	1234.594	sp|P04114	sp|P04114|AP	3548	3558	yes	yes	2	9.19E-95	207.29	924	174	942	146707;1467	69252;692	69439	225
ENFLFLTPDCK	IVQTNCSKENFLFLTP	LFLTPDCKSLWNGDTG	K	E	N	C	K	S	0	0	1	1	1	0	1	0	0	0	2	1	0	2	1	0	1	0	0	0	0	0	11	0	1382.654	sp|P01042	sp|P01042|KN	209	219	yes	yes	2;3	4.73E-32	166.19	925	103	943	147093;1470	69486;694	69515	321
ENFQNWLK	VGRPQPGRENFQNWLK	ENFQNWLKDGTVLCEL	R	E	N	L	K	D	0	0	2	0	0	1	1	0	0	0	1	1	0	1	0	0	0	1	0	0	0	0	8	0	1077.524	sp|P37802	sp|P37802|TA	50	57	yes	yes	2	4.81E-05	107.66	926	355	944	147524;1475	69815;698	69819	40
ENFVLTTAK	FCGGVIIRENFVLTTA	NFVLTTAKCSLLHRNI	R	E	N	A	K	C	1	0	1	0	0	0	1	0	0	0	1	1	0	1	0	0	2	0	0	1	0	0	9	0	1021.544	sp|P22891	sp|P22891|PR	210	218	yes	yes	2	0.0022473	99.442	927	316	945	147599;1476	69855;698	69899	49
ENLLDFIK	NFEGEVTKENLLDFIK	ENLLDFIKHNQLPLVI	K	E	N	I	K	H	0	0	1	1	0	0	1	0	0	1	2	1	0	1	0	0	0	0	0	0	0	0	8	0	990.5386	sp|P07237	sp|P07237|PD	223	230	yes	yes	2	0.056382	65.252	928	213	946	147689;1476	69904;699	69904	3
ENPAVIDFELAPIVDLVR	SEWLESVKENPAVIDF	APIVDLVRNIPCAVTK	K	E	N	V	R	N	2	1	1	2	0	0	2	0	0	2	2	0	0	1	2	0	0	0	0	3	0	0	18	0	2009.083	sp|P13671	sp|P13671|CO	478	495	yes	yes	2;3	5.14E-257	244.44	929	277	947	147770;1477	69907;699	69911	41
ENQFDPPIVAR	NSDASTIKENQFDPPI	FDPPIVARYIRISPTR	K	E	N	A	R	Y	1	1	1	1	0	1	1	0	0	1	0	0	0	1	2	0	0	0	0	1	0	0	11	0	1284.646	sp|P12259	sp|P12259|FA	2029	2039	yes	yes	2	7.50E-12	128	930	264	948	147927;1479	69950;699	69952	23
ENQLEVLEVSWLHGLK	TLDTLVLKENQLEVLE	VSWLHGLKALGHLDLS	K	E	N	L	K	A	0	0	1	0	0	1	3	1	1	0	4	1	0	0	0	1	0	1	0	2	0	0	16	0	1893	sp|P02750	sp|P02750|A2	149	164	yes	yes	2;3	2.58E-70	158.04	931	152	949	148042;1480	69973;699	70049	96
ENSLLFDPLSSSSSNK	NWGLVTYRENSLLFDP	LSSSSSNKERVVTVIA	R	E	N	N	K	E	0	0	2	1	0	0	1	0	0	0	3	1	0	1	1	6	0	0	0	0	0	0	16	0	1723.827	sp|P15144	sp|P15144|AM	364	379	yes	yes	2	5.42E-64	153.75	932	285	950	148235;1482	70071;700	70075	8
ENSQYQPIK	QLDNGSFKENSQYQPI	NSQYQPIKLQGTLPVE	K	E	N	I	K	L	0	0	1	0	0	2	1	0	0	1	0	1	0	0	1	1	0	0	1	0	0	0	9	0	1105.54	sp|P01031	sp|P01031|CO	1120	1128	yes	yes	2	0.0024354	98.523	933	98	951	148314;1483	70079;700	70080	9
ENTVTNDWIPEGEEDDDYLDLEK	LLPADFHKENTVTNDW	DDYLDLEKIFSEDDDY	K	E	N	E	K	I	0	0	2	5	0	0	5	1	0	1	2	1	0	0	1	0	2	1	1	1	0	0	23	0	2738.172	sp|P05546	sp|P05546|HE	62	84	yes	yes	2;3	1.65E-149	166.04	934	199	952	148406;1484	70088;700	70112	70
ENYPLPWEK	NASVHYIRENYPLPWE	NYPLPWEKDTEKLVAF	R	E	N	E	K	D	0	0	1	0	0	0	2	0	0	0	1	1	0	0	2	0	0	1	1	0	0	0	9	0	1174.566	sp|P80108	sp|P80108|PH	102	110	yes	yes	2	0.0042779	90.601	935	422	953	148702;1487	70158;701	70163	16
EPAVLELEGK	SPKRATKKEPAVLELE	AVLELEGKKWRVENQE	K	E	P	G	K	K	1	0	0	0	0	0	3	1	0	0	2	1	0	0	1	0	0	0	0	1	0	0	10	0	1083.581	sp|Q01518	sp|Q01518|CA	318	327	yes	yes	2	0.046614	55.112	937	429	955	148932;1489	70206;702	70206	2
EPCVESLVSQYFQTVTDYGK	ALVRRQAKEPCVESLV	QTVTDYGKDLMEKVKS	K	E	P	G	K	D	0	0	0	1	1	2	2	1	0	0	1	1	0	1	1	2	2	0	2	3	0	0	20	0	2349.084	sp|P02652	sp|P02652|AP	27	46	yes	yes	2;3	3.96E-179	265.12	938	138	956	148971;1489	70208;702	70234	32
EPEEVATCVGR	LGIPVNSKEPEEVATC	EVATCVGRNGYPIPQV	K	E	P	G	R	N	1	1	0	0	1	0	3	1	0	0	0	0	0	0	1	0	1	0	0	2	0	0	11	0	1245.566	sp|P43121	sp|P43121|MU	154	164	yes	yes	2	0.0012549	81.972	939	359	957	149107;1491	70240;702	70241	2

© 2020 Mongan D et al. *JAMA Psychiatry*.

[illegible]

© 2020 Mongan D et al. *JAMA Psychiatry*.

[illegible]

© 2020 Mongan D et al. *JAMA Psychiatry*.

ITLLSALVETR	ASAATAVKITLLSALV	LSALVETRTIVRFNRP	K	I	T	T	R	T	1	1	0	0	0	0	1	0	0	1	3	0	0	0	0	1	2	0	0	1	2	0	0	1	0	0	11	0	1214.723	sp|P01011	sp|P01011|AA	380	390	yes	yes	2;3	4.80E-237	260.06	1813	94	1845	292464;2924	137347;13	137390	1250
ITLPDFR	VEDIPLARITLPDFRL	RITLPDFRLPEIAIPE	R	I	T	F	R	L	0	1	0	1	0	0	0	0	0	1	1	0	0	1	1	0	1	0	1	0	1	0	0	0	0	0	7	0	860.4756	sp|P04114	sp|P04114|AP	2706	2712	yes	yes	2	2.09E-11	143.37	1814	174	1846	294210;2942	138623;13	138665	329
ITLPDFTGDLR	ALQSELLRITLPDFTG	PDFTGDLRIPHVGRGR	R	I	T	L	R	I	0	1	0	2	0	0	0	1	0	1	2	0	0	1	1	0	2	0	1	0	2	0	0	0	0	0	11	0	1246.656	sp|P18428	sp|P18428|LBI	57	67	yes	yes	2	3.53E-47	183.47	1815	301	1847	294827;2948	138952;13	139087	136
ITLPVDFVTADK	KAEKNGVKITLPVDFV	VDFVTADKFDENAKTG	K	I	T	D	K	F	1	0	0	2	0	0	0	0	0	1	1	1	0	1	1	0	2	0	1	0	2	0	0	2	0	0	12	0	1317.718	sp|P00558	sp|P00558|PG	280	291	yes	yes	2	2.93E-10	116.77	1816	78	1848	295022;2950	139088;13	139095	97
ITLSQVGDVLR	FDRTGDSKITLSQVGD	SQVGDVLRALGTNPTN	K	I	T	L	R	A	0	1	0	1	0	1	0	1	0	1	2	0	0	0	0	1	1	0	0	1	1	0	0	2	0	0	11	0	1199.687	sp|P05976	sp|P05976|MY	70	80	yes	yes	2	7.60E-26	158.76	1817	201	1849	295187;2951	139185;13	139187	3
ITPSYVAFTPEGER	IANDQGNRITPSYVAF	AFTPEGERLIGDAAKN	R	I	T	E	R	L	1	1	0	0	0	0	2	1	0	1	0	0	0	1	2	1	2	0	2	1	2	0	1	1	0	0	14	0	1565.773	sp|P11021	sp|P11021|GR	61	74	yes	yes	2	5.73E-06	88.441	1818	254	1850	295206;2952	139188;13	139188	14
ITVVAGEHNIEETEHTEQK	HCVETGVKITVVAGEH	ETEHTEQKRNVIRIIP	K	I	T	Q	K	R	1	0	1	0	0	1	5	1	2	2	0	1	0	0	0	0	3	0	0	0	3	0	0	2	0	0	19	0	2163.044	sp|P00740	sp|P00740|FA	275	293	yes	yes	3;4	1.15E-13	83.776	1821	84	1853	295754;2957	139332;13	139332	4
ITYGETGGNSPVQEFTVPGSK	AVTVRYYRITYGETGG	EFTVPGSKSTATISGL	R	I	T	S	K	S	0	0	1	0	0	1	2	4	0	1	0	1	0	1	2	2	3	0	2	2	3	0	1	2	0	0	21	0	2167.043	sp|P02751	sp|P02751|FIN	1480	1500	yes	yes	2;3	4.82E-166	196.8	1822	153	1854	295781;2957	139336;13	139601	296
IVACASYK	YGNSDYKRIVACASYK	IVACASYKPSREESSS	R	I	V	Y	K	P	2	0	0	0	1	0	0	0	0	1	0	1	0	0	0	1	0	0	0	1	0	0	1	1	0	0	8	0	910.4582	sp|P01031	sp|P01031|CO	1402	1409	yes	no	2	0.023034	89.142	1823	98	1855	296100;2961	139634;13	139635	6
IVACASYKPSR	YGNSDYKRIVACASYK	CASYKPSREESSSGSS	R	I	V	S	R	E	2	1	0	0	1	0	0	0	0	1	0	1	0	0	1	2	0	0	1	2	0	0	1	1	0	0	11	1	1250.644	sp|P01031	sp|P01031|CO	1402	1412	yes	yes	2;3	0.0038414	93.111	1824	98	1856	296177;2961	139640;13	139642	7
IVAEEFLK	LSVNARARIVAEEFLK	IVAEEFLKNF______	R	I	V	L	K	N	1	0	0	0	0	0	2	0	0	1	1	1	0	1	0	0	0	0	0	0	0	0	0	1	0	0	8	0	947.5328	sp|P59998	sp|P59998|AR	159	166	yes	yes	2	0.023104	79.469	1825	395	1857	296369;2963	139647;13	139648	12
IVAISEDYPR	QNGTYTVKIVAISEDY	AISEDYPRKTITGTVL	K	I	V	P	R	K	1	1	0	1	0	0	1	0	0	2	0	0	0	0	1	1	0	0	1	1	0	0	1	1	0	0	10	0	1161.603	sp|Q14126	sp|Q14126|DS	469	478	yes	yes	2	0.0018084	90.657	1826	459	1858	296464;2964	139659;13	139659	2
IVASTLSNPELFEEWTGNVK	NPPAQGARIVASTLSN	EEWTGNVKTMADRILT	R	I	V	V	K	T	1	0	2	0	0	0	3	1	0	1	2	1	0	1	1	2	2	1	1	2	2	1	0	2	0	0	20	0	2233.127	sp|P17174	sp|P17174|AA	306	325	yes	yes	2	1.06E-35	119.69	1827	294	1859	296496	139661	139661	1
IVDLVSELK	VKKETRGKIVDLVSEL	VDLVSELKKDVLMVLV	K	I	V	L	K	K	0	0	0	1	0	0	1	0	0	1	2	1	0	0	0	1	0	0	0	1	0	0	0	2	0	0	9	0	1014.596	sp|P29622	sp|P29622|KA	194	202	yes	yes	2	0.0007412	117.04	1828	336	1860	296497;2964	139662;13	139686	128
IVDLVSELKK	VKKETRGKIVDLVSEL	DLVSELKKDVLMVLVN	K	I	V	K	K	D	0	0	0	1	0	0	1	0	0	1	2	2	0	0	0	1	0	0	0	1	0	0	0	2	0	0	10	1	1142.691	sp|P29622	sp|P29622|KA	194	203	yes	yes	3	0.0010156	65.278	1829	336	1861	296674;2966	139790;13	139791	7
IVEQINR	HIGGVLLKIVEQINRK	KIVEQINRKQDWSDHA	K	I	V	N	R	K	0	1	1	0	0	1	1	0	0	2	0	0	0	0	0	0	0	0	0	0	0	0	0	1	0	0	7	0	870.4923	sp|Q86UX7	sp|Q86UX7|UI	49	55	yes	yes	2	0.0033618	137.5	1830	497	1862	296737;2967	139797;13	139800	12
IVGGTASVR	CTTKIKPRIVGGTASV	VGGTASVRGEWPWQVT	R	I	V	V	R	G	1	1	0	0	0	0	0	2	0	1	0	0	0	0	0	0	1	1	0	1	1	0	0	2	0	0	9	0	858.4923	sp|P03951	sp|P03951|FA	388	396	yes	yes	2	0.0033119	95.741	1831	165	1863	296811;2968	139809;13	139809	4
IVGPSGAAVPCK	GQGKVASKIVGPSGAA	SGAAVPCKVEPGLGAD	K	I	V	C	K	V	2	0	0	0	1	0	0	2	0	1	0	1	0	0	2	1	0	0	2	1	0	0	0	2	0	0	12	0	1154.612	sp|P21333	sp|P21333|FLN	1008	1019	yes	yes	2	0.0001334	82.287	1832	310	1864	296951;2969	139813;13	139815	14
IVGQLMDGLK	NPLREIDKIVGQLMDG	GQLMDGLKQLKLHRCV	K	I	V	L	K	Q	0	0	0	1	0	1	0	2	0	1	2	1	1	0	0	0	0	0	0	0	0	0	0	1	0	0	10	0	1072.595	sp|Q13822	sp|Q13822|EN	335	344	yes	yes	2	0.058823	52.255	1833	455	1865	297024;2970	139827	139827	1
IVIEYVDR	WIHPDLKRIVIEYVDR	IVIEYVDRIIFHENYN	R	I	V	D	R	I	0	1	0	1	0	0	1	0	0	2	0	0	0	0	0	0	0	0	0	0	0	1	2	0	0	0	8	0	1005.55	sp|P05156	sp|P05156|CF	407	414	yes	yes	2	2.80E-05	141.78	1834	192	1866	297094;2970	139828;13	139849	135
IVILPDYLEIAR	FELDEDVKIVILPDYL	PDYLEIARDGLGGLPD	K	I	V	A	R	D	1	1	0	1	0	0	1	0	0	3	2	0	0	0	1	0	0	0	1	0	0	0	1	1	0	0	12	0	1413.823	sp|P45974	sp|P45974|UB	122	133	yes	yes	2	3.33E-10	116.3	1835	363	1867	297422;2974	139963;13	139965	8
IVLGQEQDSYGGK	YFVEAQPKIVLGQEQD	EQDSYGGKFDRSQSFV	K	I	V	G	K	F	0	0	0	1	0	2	1	3	0	1	1	1	0	0	0	1	0	0	0	1	0	0	1	1	0	0	13	0	1392.689	sp|P02743	sp|P02743|SA	150	162	yes	yes	2	2.30E-113	209.73	1836	146	1868	297491;2974	139973;13	139994	188
IVLLDVPVR	NSQNPNSKIVLLDVPV	VLLDVPVRAEAQVELR	K	I	V	V	R	A	0	1	0	1	0	0	0	0	0	1	2	0	0	0	1	0	0	0	1	0	0	0	0	3	0	0	9	0	1022.649	sp|P08514	sp|P08514|ITA	766	774	yes	yes	2	0.0007743	121.99	1837	229	1869	297943;2979	140164;14	140189	28
IVLVDNK	VKAQEDERIVLVDNKC	RIVLVDNKCKCARITS	R	I	V	N	K	C	0	0	1	1	0	0	0	0	0	1	1	1	0	0	0	0	0	0	0	0	0	0	0	2	0	0	7	0	799.4804	sp|P01591	sp|P01591|IGJ	28	34	yes	yes	2	0.01709	100.07	1838	105	1870	298037;2980	140192;14	140193	2
IVQILPWEQNEQVK	PSQADINKIVQILPWE	WEQNEQVKNFVASHIA	K	I	V	V	K	N	0	0	1	0	0	3	2	0	0	2	1	1	0	0	1	0	0	1	1	0	0	1	0	2	0	0	14	0	1722.931	sp|P04114	sp|P04114|AP	577	590	yes	yes	2;3	1.67E-48	160.77	1839	174	1871	298113;2981	140194;14	140530	381
IVQLIQDTR	EFRNRNQRIVQLIQDT	VQLIQDTRIHILPSMN	R	I	V	T	R	I	0	1	0	1	0	2	0	0	0	2	1	0	0	0	0	0	1	0	0	0	1	0	0	1	0	0	9	0	1084.624	sp|P15169	sp|P15169|CB	114	122	yes	yes	2	1.02E-08	147.29	1840	286	1872	298648;2986	140578;14	140584	46
IVSSAMEPDR	VTAPENGKIVSSAMEP	SSAMEPDREYHFGQAV	K	I	V	D	R	E	1	1	0	1	0	0	1	0	0	1	0	0	1	0	1	2	0	0	1	2	0	0	0	1	0	0	10	0	1103.528	sp|P08603	sp|P08603|CF	157	166	yes	yes	2	7.33E-07	124.23	1841	233	1873	298911;2989	140635;14	140682	64
IVSSAMEPDREYHFGQAVR	VTAPENGKIVSSAMEP	YHFGQAVRFVCNSGYK	K	I	V	V	R	F	2	2	0	1	0	1	2	1	1	1	0	0	1	1	1	2	0	0	1	2	0	0	1	2	0	0	19	1	2191.048	sp|P08603	sp|P08603|CF	157	175	yes	yes	4	7.39E-05	61.102	1842	233	1874	299116;2991	140699;14	140700	6
IVVVTAGVR	YSVTANSKIVVVTAGV	VVVTAGVRQQEGESRL	K	I	V	V	R	Q	1	1	0	0	0	0	0	1	0	1	0	0	0	0	0	0	0	0	0	0	1	0	0	4	0	0	9	0	912.5757	sp|P07195	sp|P07195|LD	92	100	yes	yes	2	4.18E-19	166.05	1843	211	1875	299148;2991	140705;14	140706	49
IWLDNVR	CYGPGVGRIWLDNVRC	RIWLDNVRCSGEEQSL	R	I	W	V	R	C	0	1	1	1	0	0	0	0	0	1	1	0	0	0	0	0	0	0	0	0	0	1	0	1	0	0	7	0	914.4974	sp|O43866	sp|O43866|CD	308	314	yes	yes	2	0.0050603	121.68	1844	57	1876	299277;2992	140755;14	140762	31
IYEDHDATQQLQGFYSQVAK	ENNGRAQRIYEDHDAT	GFYSQVAKPLLVDVDL	R	I	Y	A	K	P	2	0	0	2	0	4	1	1	1	1	1	1	0	1	0	1	1	0	0	1	1	0	2	1	0	0	20	0	2340.102	sp|P19827	sp|P19827|ITI	458	477	yes	yes	2;3	5.76E-289	309.23	1845	305	1877	299354;2993	140786;14	140962	202
IYGNQDTSSQLK	ENHGIAQRIYGNQDTS	QDTSSQLKKFYNQVST	R	I	Y	L	K	K	0	0	1	1	0	2	0	1	0	1	1	1	0	0	0	2	1	0	0	2	1	0	1	0	0	0	12	0	1352.657	sp|P19823	sp|P19823|ITI	476	487	yes	yes	2	4.65E-104	208.03	1846	304	1878	299735;2997	140988;14	141061	82
IYHSHIDAPK	GDGNCVTRIYHSHIDA	HSHIDAPKDIASGLIG	R	I	Y	P	K	D	1	0	0	1	0	0	0	0	2	2	0	1	0	0	1	1	0	0	1	1	0	0	1	0	0	0	10	0	1179.604	sp|P00450	sp|P00450|CEI	178	187	yes	no	2	8.79E-07	120.31	1847	74	1879	300071;3000	141070;14	141071	4

[illegible]

© 2020 Mongan D et al. *JAMA Psychiatry*.

LLDSLPSDTR	NTNNKISRLLDSLPSD	DSLPSDTRLVLLNAIY	R	L	L	T	R	[illegible]
LLELTGPK	TGWTQLSKLLELTGPK	LLELTGPKSLPAPWLS	K	L	L	P	K	[illegible]
LLEPHCFPLSLVPTEFCPSPPALK	DEELEHLRLLEPHCFP	CPSPPALKDGFVQDEG	R	L	L	L	K	[illegible]
LLESPATALGDIR	GRPSLFQRLLESPATA	ATALGDIRREMSEAAQ	R	L	L	I	R	[illegible]
LLETECPQYIR	VYRDDLKKLLETECPQ	TECPQYIRKKGADVWF	K	L	L	I	R	[illegible]
LLETIDQLYLEYAK	EALERTEKLLETIDQL	QLYLEYAKRAAPFNNW	K	L	L	A	K	[illegible]
LLEVPEGR	VFIHPGWKLLEVPEGR	LLEVPEGRTNFDNDIA	K	L	L	G	R	[illegible]
LLGELLQDNAK	DFLTISPKLLGELLQD	ELLQDNAKLVPVLSAK	K	L	L	A	K	[illegible]
LLGEVDHYQLALGK	FAHYATFRLLGEVDHY	HYQLALGKFSEGTAGD	R	L	L	G	K	[illegible]
LLGIETPLPK	STSCDQPKLLGIETPL	GIETPLPKKELLLPGN	K	L	L	P	K	[illegible]
LLGLSLAGK	TEAKEACRLLGLSLAG	LGLSLAGKDQVETALK	R	L	L	G	K	[illegible]
LLGMETMAWQEIR	LMMNNQMRLLGMETMA	TMAWQEIRHAIILLTD	R	L	L	I	R	[illegible]
LLGNVLVCVLAR	HVDPENFRLLGNVLVC	VLVCVLARNFGKEFTP	R	L	L	A	R	[illegible]
LLGQLLR	QCDESNLRLLGQLLRV	RLLGQLLRVLARHDLL	R	L	L	L	R	[illegible]
LLGWIQNK	KKQTPKQRLLGWIQNK	LLGWIQNKLPQLPITN	R	L	L	N	K	[illegible]
LLIGTVFHK	VASVYKGKLLIGTVFH	LIGTVFHKALYCEL__	K	L	L	H	K	[illegible]
LLIYAVLPTGDVIGDSAK	SDIAPVARLLIYAVLP	DVIGDSAKYDVENCLA	R	L	L	A	K	[illegible]
LLIYDASNLESGVPSR	QKPGKAPKLLIYDASN	LESGVPSRFSGGGSGA	K	L	L	S	R	[illegible]
LLIYDASNLETGVPSR	QKPKKAPKLLIYDASN	LETGVPSRFSESGSGT	K	L	L	S	R	[illegible]
LLIYDASNR	QKPGQAPRLLIYDASN	LIYDASNRATGIPARF	R	L	L	N	R	[illegible]
LLIYGASSR	QKPGQAPRLLIYGASS	LIYGASSRATGIPDRF	R	L	L	S	R	[illegible]
LLIYGASTR	QKPGLAPKLLIYGAST	LIYGASTREAGVPSRF	K	L	L	T	R	[illegible]
LLIYGATSR	QKPGQAPRLLIYGATS	LIYGATSRATGIPDRF	R	L	L	S	R	[illegible]
LLIYWASTR	QKPGQPPKLLIYWAST	LIYWASTRESGVPDRF	K	L	L	T	R	[illegible]
[illegible]	[illegible]	[illegible]	[illegible]	[illegible]	[illegible]	[illegible]	[illegible]	[illegible]

© 2020 Mongan D et al. *JAMA Psychiatry*.

MNFRPGVLSSR	QAGAAGSRMNFRPGVL	RPGVLSSRQLGLPGPP	R	M	N	S	R	Q	0	2	1	0	0	0	0	1	0	0	1	0	1	1	1	2	0	0	0	1	0	0	11	1	1262.655	sp|Q14624	sp|Q14624|ITI	658	668	yes	yes	2;3	1.06E-125	225.67	2435	463	2483;2484	391712;3917	183904;18	183906	78	52
MNTVIAALSR	FILPDKGKMNTVIAAL	TVIAALSRDTINRWSA	K	M	N	S	R	D	2	1	1	0	0	0	0	0	0	1	1	0	1	0	0	1	1	0	0	1	0	0	10	0	1074.586	sp|P08185	sp|P08185|CB	273	282	yes	yes	2	3.00E-61	195.88	2436	224	2485	391955;3919	183956;18	184042		146
MNVAPACLPER	LKTPITFRMNVAPACL	APACLPERDWAESTLM	R	M	N	E	R	D	2	1	1	0	1	0	1	0	0	0	1	0	1	0	2	0	0	0	0	1	0	0	11	0	1256.601	sp|P00742	sp|P00742|FA	336	346	yes	yes	2	5.53E-12	129.85	2437	85	2486	392133;3921	184102;18	184154		54
MPGTVATLR	________________	PGTVATLRFQLLPPEP	-	M	P	L	R	F	1	1	0	0	0	0	0	1	0	0	1	0	1	0	1	0	2	0	0	1	0	0	9	0	944.5113	sp|Q66K6	sp|Q66K66|TM	1	9	yes	yes	1;2	0.0002347	113.89	2439	484	2488;2489;	392219;3922	184162;18	184177	80	24
MPYECGPSLDVCAQDER	QNSRCVCKMPYECGPS	DVCAQDERSKRILPLT	K	M	P	E	R	S	1	1	0	2	2	1	2	1	0	0	1	0	1	0	2	1	0	0	1	1	0	0	17	0	2025.823	sp|P10643	sp|P10643|CO	717	733	yes	yes	2	1.40E-149	202.32	2440	252	2491	392456;3924	184186;18	184186		54
MSAEINEIIR	NRNFTVEKMSAEINEI	AEINEIIRVLQLTSWD	K	M	S	I	R	V	1	1	1	0	0	0	2	0	0	3	0	0	1	0	0	1	0	0	0	0	0	0	10	0	1174.602	sp|P18206	sp|P18206|VIN	237	246	yes	yes	2	2.87E-05	111.39	2441	300	2492	392564;3925	184240;18	184240		9
MSAVEGICTSESPVIDHQGTK	SGMQFCVKMSAVEGIC	VIDHQGTKSSKCVRQK	K	M	S	T	K	S	1	0	0	1	1	1	2	2	1	2	0	1	1	0	1	3	2	0	0	2	0	0	21	0	2245.036	sp|P01031	sp|P01031|CO	859	879	yes	yes	3	4.11E-70	140.35	2442	98	2493	392633;3926	184249;18	184252		54
MTGLVDEAIDTK	QWIDNVEKMTGLVDEA	VDEAIDTKSLLDASEE	K	M	T	T	K	S	1	0	0	2	0	0	1	1	0	1	1	1	1	0	0	0	2	0	0	1	0	0	12	0	1291.633	sp|P18206	sp|P18206|VIN	709	720	yes	yes	2	3.49E-19	132.32	2444	300	2495	392780;3927	184304;18	184304		7
MTSNFPVDLSDYPK	GTNVDTKKMTSNFPVD	VDLSDYPKSLHMYANR	K	M	T	P	K	S	0	0	1	2	0	0	0	0	0	0	1	1	1	1	2	2	1	0	1	1	0	0	14	0	1612.744	sp|P04114	sp|P04114|AP	1189	1202	yes	yes	2	2.76E-32	147.62	2446	174	2497;2498	392877;3928	184316;18	184504	48	193
MTVTDQVNCPK	SNNIDVKKMTVTDQVN	TDQVNCPKLS______	K	M	T	P	K	L	0	0	1	1	1	1	0	0	0	0	0	1	1	0	1	0	2	0	0	2	0	0	11	0	1291.59	sp|P05090	sp|P05090|AP	177	187	yes	yes	2	1.55E-15	133.57	2447	188	2499	393205;3932	184509;18	184519		34
MVCGPSGSQLVLLK	LQRVPVAKMVCGPSGS	GSQLVLLKLERSVTLN	K	M	V	L	K	L	0	0	0	0	1	1	0	2	0	0	3	1	1	0	1	2	0	0	0	2	0	0	14	0	1487.784	sp|P26927	sp|P26927|HG	560	573	yes	yes	2	3.14E-25	131.09	2448	330	2500	393353;3933	184543;18	184547		101
MVETTAYALLTSLNLK	VPNTGTARMVETTAYA	LLTSLNLKDINYVNPV	R	M	V	L	K	D	2	0	1	0	0	0	1	0	0	0	4	1	1	0	0	1	3	0	1	1	0	0	16	0	1766.949	sp|P01031	sp|P01031|CO	1247	1262	yes	yes	2;3	2.29E-78	163.78	2449	98	2501	393498;3934	184644;18	184648		90
MVGHLHLQGMEDSLK	________________	QGMEDSLKEQGREGLL	-	M	V	L	K	E	0	0	0	1	0	1	1	2	2	0	3	1	2	0	0	1	0	0	0	1	0	0	15	0	1693.828	sp|Q6ZTI6	sp|Q6ZTI6|F1	1	15	yes	yes	2	0.010294	42.395	2450	490	2502	393677;3936	184734	184734	81	1
MVQQECK	ETTEKSLKMVQQECKH	KMVQQECKHFQNLGKD	K	M	V	C	K	H	0	0	0	0	1	2	1	0	0	0	0	1	1	0	0	0	0	0	0	1	0	0	7	0	921.4048	sp|P43652	sp|P43652|AF	411	417	yes	yes	2	0.016073	100.07	2451	362	2503	393777	184735	184735		1
MVSGFIPLKPTVK	NMAIVDVKMVSGFIPL	IPLKPTVKMLERSNHV	K	M	V	V	K	M	0	0	0	0	0	0	0	1	0	1	1	2	1	1	2	1	1	0	0	2	0	0	13	1	1415.821	sp|P01023	sp|P01023|A2	1385	1397	no	no	2;3	0.0007735	97.813	2452	96;307	2504	393778;3937	184736;18	184746		13
MVSTSATDEPR	SPVRGSKRMVSTSATD	TSATDEPRETPGRPPD	R	M	V	P	R	E	1	1	0	1	0	0	1	0	0	0	0	0	1	0	1	2	2	0	0	1	0	0	11	0	1192.539	sp|Q8IY17	sp|Q8IY17|PLF	361	371	yes	yes	2	0.010508	74.127	2453	501	2505	393874;3938	184749;18	184752	83	6
MVTAVASALSSR	EVQASWQKMVTAVASA	VASALSSRYH______	K	M	V	S	R	Y	3	1	0	0	0	0	0	0	0	0	1	0	1	0	0	3	1	0	0	2	0	0	12	0	1191.628	sp|P69891	sp|P69891|HB	134	145	yes	yes	2	4.30E-08	107.83	2454	417	2506	393940;3939	184755;18	184756		3
MVTGVASALSSR	EVQASWQKMVTGVASA	VASALSSRYH______	K	M	V	S	R	Y	2	1	0	0	0	0	0	1	0	0	1	0	1	0	0	3	1	0	0	2	0	0	12	0	1177.613	sp|P69892	sp|P69892|HB	134	145	yes	yes	2	1.43E-20	139.43	2455	418	2507	393945;3939	184758;18	184758		2
MVVESAYEVIK	ENWKEVHKMVVESAYE	ESAYEVIKLKGYTNWA	K	M	V	I	K	L	1	0	0	0	0	0	2	0	0	1	0	1	1	0	0	1	0	0	1	3	0	0	11	0	1266.653	sp|P07195	sp|P07195|LD	234	244	yes	yes	2	0.0016689	93.111	2456	211	2508	393971;3939	184760;18	184760		6
MWPGNAWRAALFWVPR	________________	AALFWVPRGRRAQSAL	-	M	W	P	R	G	3	2	1	0	0	0	0	1	0	0	1	0	1	1	2	0	0	3	0	1	0	0	16	1	1956.993	sp|O7560	sp|O75600|KB	1	16	yes	yes	2	0.0031326	51.268	2457	62	2509	394052;3940	184766;18	184767	11	7
MYAPAWVAPEALQK	FSFQCPGRMYAPAWVA	VAPEALQKKPEDTNRR	R	M	Y	Q	K	K	4	0	0	0	0	1	1	1	0	2	0	0	1	1	1	0	0	[illegible]	[illegible]	[illegible]	[illegible]	[illegible]	14	0	1573.796	sp|Q13418	sp|Q13418|ILK	350	363	yes	yes	2	0.027119	47.894	2458	450	2510	394083;3940	184773	184773		1
MYLGYEYVTAIR	VPPRMDAKMYLGYEYV	YEYVTAIRNLREGTCP	K	M	Y	I	R	N	1	1	0	0	0	0	1	1	0	1	1	0	1	0	0	0	1	0	3	1	0	0	12	0	1477.728	sp|P02787	sp|P02787|TR	332	343	yes	yes	2;3	1.26E-22	142.43	2460	162	2512;2513	394146;3941	184794;18	184831	40	167
MYQMDIQQELQR	TLEDTRDRMYQMDIQQ	DIQQELQRYLSLVGQV	R	M	Y	Q	R	Y	0	1	0	1	0	4	1	0	0	1	1	0	2	0	0	0	0	0	1	0	0	0	12	0	1581.728	sp|P04114	sp|P04114|AP	2523	2534	yes	yes	2;3	8.33E-137	227.88	2461	174	2514;2515	394384;3943	184965;18	185129	49	196
MYYSAVDPTK	ADPVCLAKMYYSAVDP	YSAVDPTKDIFTGLIG	K	M	Y	T	K	D	1	0	0	1	0	0	0	0	0	0	0	1	1	0	1	1	1	0	2	1	0	0	10	0	1173.538	sp|P00450	sp|P00450|CEI	538	547	yes	no	2	3.43E-07	130.75	2462	74	2516;2517	394696;3946	185161;18	185195	14	149
NAAENGAIQNLIK	QASVATPRNAAENGAI	GAIQNLIKEEAFLGIT	R	N	A	I	K	E	3	0	3	0	0	1	1	1	0	2	1	1	0	0	0	0	0	0	0	0	0	0	13	0	1354.721	sp|P11226	sp|P11226|ME	167	179	yes	yes	2	1.73E-39	159.79	2463	258	2518	395153;3951	185310;18	185310		4
NADPILISLK	AEEWFEGKNADPILIS	DPILISLKHGYIPGKN	K	N	A	L	K	H	1	0	1	1	0	0	0	0	0	2	2	1	0	0	1	1	0	0	0	0	0	0	10	0	1082.634	sp|Q9ULV4	sp|Q9ULV4|CC	382	391	yes	yes	2	0.0028739	83.998	2464	550	2519	395170;3951	185314;18	185316		29
NADYSYSVWK	MLSIMSYRNADYSYSV	DYSYSVWKGGSASTWL	R	N	A	W	K	G	1	0	1	1	0	0	0	0	0	0	1	0	0	0	2	0	1	2	1	0	0	0	10	0	1231.551	sp|P01031	sp|P01031|CO	1061	1070	yes	yes	2	9.87E-07	119.21	2465	98	2520	395291;3952	185343;18	185350		86
NAIWIDCGIHAR	SGKEQAAKNAIWIDCG	IDCGIHAREWISPAFC	K	N	A	A	R	E	2	1	1	1	1	0	0	1	1	3	0	0	0	0	0	0	0	1	0	0	0	0	12	0	1424.698	sp|Q96IY4	sp|Q96IY4|CBI	172	183	yes	yes	2;3	7.27E-28	155.84	2466	516	2521	395490;3954	185429;18	185476		67
NALALFVLPK	VLQMDYSKNALALFVL	LALFVLPKEGQMESVE	K	N	A	P	K	E	2	0	1	0	0	3	1	0	1	1	0	0	0	0	1	0	0	[illegible]	[illegible]	[illegible]	[illegible]	[illegible]	10	0	1084.665	sp|P05543	sp|P05543|TH	264	273	yes	no	1;2	0.0027828	84.568	2467	198	2522	395610;3956	185496;18	185610		124
NALFCLESAWK	TVTHPVVRNALFCLES	FCLESAWKTAQEGDHG	R	N	A	W	K	T	2	0	1	0	1	2	1	0	1	0	1	0	1	0	0	0	0	[illegible]	[illegible]	[illegible]	[illegible]	[illegible]	11	0	1337.644	sp|P01023	sp|P01023|A2	1123	1133	yes	yes	2	3.84E-12	131.44	2468	96	2523	395877;3958	185620;18	185778		158
NALFCLESAWNVAK	PVTNPIVRNALFCLES	ESAWNVAKEGTHGSHV	R	N	A	A	K	E	3	0	2	0	1	2	1	0	1	0	1	0	1	0	1	0	0	[illegible]	[illegible]	[illegible]	[illegible]	[illegible]	14	0	1621.792	sp|P20742	sp|P20742|PZI	1129	1142	yes	yes	2	3.38E-12	101.95	2469	307	2524	396193;3961	185780;18	185791		13
NALTGLPPGLFQASATLDTLVLK	LRVLDLTRNALTGLPP	TLDTLVLKENQLEVLE	R	N	A	L	K	E	3	0	1	1	0	6	1	0	1	2	1	3	0	0	1	0	0	[illegible]	[illegible]	[illegible]	[illegible]	[illegible]	23	0	2339.31	sp|P02750	sp|P02750|A2	126	148	yes	yes	2;3	5.07E-66	146.1	2470	152	2525	396216;3962	185793;18	185798		37
NALWHTGDTESQVR	TGPGEQLRNALWHTGD	GDTESQVRLLWKDPRN	R	N	A	V	R	L	1	1	1	1	0	1	1	1	1	0	0	0	0	1	2	1	0	[illegible]	[illegible]	[illegible]	[illegible]	[illegible]	14	0	1612.759	sp|P49747	sp|P49747|CO	652	665	yes	yes	3	1.46E-13	88.092	2471	373	2526	396375;3963	185836;18	185836		6

[illegible]

© 2020 Mongan D et al. *JAMA Psychiatry*.

PYPPNVGEEIQIGHIPR	TPIRHRPRPYPPNVGE	IQIGHIPREDVDYHLY	R	P	Y	P	R	E	0	1	1	0	[illegible]
PYQYPALTPEQK	________________	PALTPEQKKELSDIAH	M	P	Y	Q	K	K	1	0	0	0	[illegible]
PYTFHSHGITYYK	HLKNLASRPYTFHSHG	SHGITYYKEHEGAIYP	R	P	Y	Y	K	E	0	0	0	0	[illegible]
PYVPQLGTYNVIPLNNEQSSGPVPETVR	AGLGVVERPYVPQLGT	GPVPETVRSYFPETWI	R	P	Y	V	R	S	0	1	3	0	[illegible]
PYYSDVDIMR	NDAQCLTRPYYSDVDI	YSDVDIMRDIASGLIG	R	P	Y	M	R	D	0	1	0	2	[illegible]
QAAASATQTIAAAQHAASTPK	QRLEHAAKQAAASATQ	QHAASTPKASAGPQPL	K	Q	A	P	K	A	9	0	0	0	[illegible]
QAEISASAPTSLR	RRDSWLDKQAEISASA	ASAPTSLRSCDVESNP	K	Q	A	L	R	S	3	1	0	0	[illegible]
QAFTQQIEELK	VSQLSRGKQAFTQQIE	TQQIEELKRQLEEEIK	K	Q	A	L	K	R	1	0	0	0	[illegible]
QAGPASVPLR	GIVSHLKKQAGPASVP	GPASVPLRTEEEFKKF	K	Q	A	L	R	T	2	1	0	0	[illegible]
QALEEFQK	STVRQNMKQALEEFQK	QALEEFQKEVSSCHCA	K	Q	A	Q	K	E	1	0	0	0	[illegible]
QAQCGQDFQCK	SSSTTCVRQAQCGQDF	CGQDFQCKETGRCLKR	R	Q	A	C	K	E	1	0	0	1	[illegible]
QAQQMVQPQSPVAVSQSK	TGASKSKRQAQQMVQP	PVAVSQSKPGCYDNGK	R	Q	A	S	K	P	2	0	0	0	[illegible]
QAVDTAVDGVFIR	RSKSSEKRQAVDTAVD	AVDGVFIRSLKVNCKV	R	Q	A	I	R	S	2	1	0	2	[illegible]
QCQDLGAFTESMVVFGCPN	CQDEENQKQCQDLGAF	MVVFGCPN________	K	Q	C	P	N	-	1	0	1	1	[illegible]
QCTMFYSTSNIK	YEYHRPDKQCTMFYST	FYSTSNIKIQKVCEGA	K	Q	C	I	K	I	0	0	1	0	[illegible]
QCVPHDQCACGVLTSEK	YRLGEDHKQCVPHDQC	CGVLTSEKRAPDLQDL	K	Q	C	E	K	R	1	0	0	1	[illegible]
QDACQGDSGGVFAVR	CAGHPSLKQDACQGDS	SGGVFAVRDPNTDRWV	K	Q	D	V	R	D	2	1	0	2	[illegible]
QDDHGYISR	IHGKHNERQDDHGYIS	DDHGYISREFHRRYRL	R	Q	D	S	R	E	0	1	0	2	[illegible]
QDGHLWCSTTSNYEQDQK	YSCTTEGRQDGHLWCS	SNYEQDQKYSFCTDHT	R	Q	D	Q	K	Y	0	0	1	2	[illegible]
QDGSVDFFR	GWLVFQRRQDGSVDFF	DGSVDFFRSWSSYRAG	R	Q	D	F	R	S	0	1	0	2	[illegible]
QDIVFDGIAQIR	PVTPEICKQDIVFDGI	FDGIAQIRGEIFFFKD	K	Q	D	I	R	G	1	1	0	2	[illegible]
QDNEILIFWSK	IFSYATKRQDNEILIF	EILIFWSKDIGYSFTV	R	Q	D	S	K	D	0	0	1	1	[illegible]
QDPPSVVVTSHQAPGEK	YSKNILDRQDPPSVVV	SHQAPGEKKKLKCLAY	R	Q	D	E	K	K	1	0	0	1	[illegible]
QEATTVSCFR	CGSEAQPRQEATTVSC	ATTVSCFRGKGEGYRG	R	Q	E	F	R	G	1	1	0	0	[illegible]
[illegible]													

© 2020 Mongan D et al. *JAMA Psychiatry*.

SLAPTAAAK	INQLLAERSLAPTAAA	LAPTAAAKRRITRSIL	R	S	L	A	K	R
SLAPYAQDTQEK	QTVEELRRSLAPYAQD	YAQDTQEKLNHQLEGL	R	S	L	E	K	L
SLDFTELDVAAEK	YTPVVLPRSLDFTELD	ELDVAAEKIDRFMQAV	R	S	L	E	K	I
SLECLHPGTK	FPTYCQQKSLECLHPG	ECLHPGTKFLNNGTCT	K	S	L	T	K	F
SLEDLQLTHNK	ESVGPLPKSLEDLQLT	DLQLTHNKITKLGSFE	K	S	L	N	K	I
SLELLVGEK	DIQLLPRKSLELLVGE	LELLVGEKLVLNCTVW	K	S	L	E	K	L
SLEVTFTPVIEDIGK	DRKSLETKSLEVTFTP	PVIEDIGKVLVCRAKL	K	S	L	G	K	V
SLEYLDLSFNQIAR	SAAFKGLKSLEYLDLS	LSFNQIARLPSGLPVS	K	S	L	A	R	L
SLFTDLVAEK	LESGRENRSLFTDLVA	FTDLVAEKDLFHCVSF	R	S	L	E	K	D
SLGECCDVEDSTTCFNAK	KVLEPTLKSLGECCDV	STTCFNAKGPLLKKEL	K	S	L	A	K	G
SLGEISALTSK	KERDDILRSLGEISAL	EISALTSKLADLRRQG	R	S	L	S	K	L
SLGFCDTTNK	SSRGQVRRSLGFCDTT	GFCDTTNKGLFQVVSG	R	S	L	N	K	G
SLGNVIMVCR	YKCRPGYRSLGNVIMV	GNVIMVCRKGEWVALN	R	S	L	C	R	K
SLGPNSCSANGPGLYLIHGPNLYCYSDVEK	DGALCMEKSLGPNSCS	YCYSDVEKLNAAKALP	K	S	L	E	K	L
SLHMYANR	VDLSDYPKSLHMYANR	SLHMYANRLLDHRVPQ	K	S	L	N	R	L
SLITFVNK	DKLSVVKKSLITFVNK	SLITFVNKHLNKLNLE	K	S	L	N	K	H
SLLDASEEAIK	VDEAIDTKSLLDASEE	DASEEAIKKDLDKCKV	K	S	L	I	K	K
SLLQPNK	QDKKYRHRSLLQPNKF	RSLLQPNKFGGTICSG	R	S	L	N	K	F
SLMLHYEFLQR	AHYKLKPRSLMLHYEF	LHYEFLQRVKRLPLEY	R	S	L	Q	R	V
SLPAPWLSMAPVSWITPGLK	LLELTGPKSLPAPWLS	SWITPGLKTTAVCRGV	K	S	L	L	K	T
SLPEGVANGIEVYSTK	PFRLLGKRSLPEGVAN	GIEVYSTKINSKVTSR	R	S	L	T	K	I
SLPEVPETIELEVR	FPGHVFSRSLPEVPET	ETIELEVRTSTASGLL	R	S	L	V	R	T
SLPTCLPVCGLPK	WMNKVLGRSLPTCLPV	LPVCGLPKFSRKLMAR	R	S	L	P	K	F
SLPTEDCENEK	QSTGRVGRSLPTEDCE	TEDCENEKEQAVHNVV	R	S	L	E	K	E
SLPVSDSVLSGFEQR	LSVKLYARSLPVSDSV	VLSGFEQRVQEAHLTE	R	S	L	Q	R	V
SLSPSFR	CKQLGCGKSLSPSFRD	KSLSPSFRDRKCYGPG	K	S	L	F	R	D
SLSQQIENIR	LEVDTTLKSLSQQIEN	SQQIENIRSPEGSRKN	K	S	L	I	R	S

[illegible]

© 2020 Mongan D et al. *JAMA Psychiatry.*

TMQENSTPR	NYVDWIMKTMQENSTP	MQENSTPRED______	K	T	M	P	R	E	0	1	1	0	0	1	1	0	0	0	0	0	1	0	1	1	2	0	0	0	0	0	9	0	1062.476	sp|P09871	sp|P09871|C1	678	686	yes	yes	2	0.0058882	83.206	3368	242	3439	533842;5338	252423;25	252423	2
TMSGLECQAWDSQSPHAHGYIPSK	NYDGKISKTMSGLECQ	AHGYIPSKFPNKNLKK	K	T	M	S	K	F	2	0	0	1	1	2	1	2	2	1	1	1	1	0	2	4	1	1	1	0	0	0	24	0	2686.19	sp|P00747	sp|P00747|PL	200	223	yes	yes	4	5.07E-19	90.475	3369	87	3440	533885;5338	252425;25	252426	11
TNEFWNK	EVFEDSDKTNEFWNKY	KTNEFWNKYKDGDQCE	K	T	N	N	K	Y	0	0	2	0	0	0	1	0	0	0	0	1	0	1	0	0	1	1	0	0	0	0	7	0	937.4294	sp|P00742	sp|P00742|FA	77	83	yes	yes	2	0.010232	106.42	3371	85	3442	533993;5339	252441;25	252444	4
TNFDNDIALVR	LLEVPEGRTNFDNDIA	DNDIALVRLKDPVKMG	R	T	N	V	R	L	1	1	2	2	0	0	0	0	0	1	1	0	0	1	0	0	1	0	0	1	0	0	11	0	1276.641	sp|P09871	sp|P09871|C1	524	534	yes	yes	2	1.77E-96	214.05	3372	242	3443	534050;5340	252445;25	252598	178
TNLESILSYPK	LGAGENTKTNLESILS	ESILSYPKDFTCVHQA	K	T	N	P	K	D	0	0	1	0	0	0	1	0	0	1	2	1	0	0	1	2	1	0	1	0	0	0	11	0	1263.671	sp|P05155	sp|P05155|IC1	191	201	yes	yes	2	1.20E-07	114.5	3373	191	3444	534376;5343	252624;25	252653	419
TNLLQVCER	QCTDKRIRTNLLQVCE	NLLQVCERIPTISTQL	R	T	N	E	R	I	0	1	1	0	1	1	1	0	0	0	2	0	0	0	0	0	1	0	0	1	0	0	9	0	1131.571	sp|P18206	sp|P18206|VIN	1047	1055	yes	yes	2	2.23E-11	154.49	3374	300	3445	534777;5347	253051;25	253075	37
TNQVNSGGVLLR	TFCGHTSKTNQVNSGG	NSGGVLLRLQVGEEVW	K	T	N	L	R	L	0	1	2	0	0	1	0	2	0	0	2	0	0	0	0	1	1	0	0	2	0	0	12	0	1256.684	sp|P02747	sp|P02747|C1	199	210	yes	yes	2	1.08E-77	195.79	3375	149	3446	534829;5348	253088;25	253119	158
TNTGLALR	IRYQGGNRTNTGLALR	TNTGLALRYLSDHSFL	R	T	N	L	R	Y	1	1	1	0	0	0	0	1	0	0	2	0	0	0	0	0	2	0	0	0	0	0	8	0	844.4767	sp|P04275	sp|P04275|VW	1576	1583	yes	yes	2	0.015153	104.45	3376	179	3447	535051;5350	253247	253247	1
TNTNVNCPIECFMPLDVQADR	YSQRYHQRTNTNVNCP	PLDVQADREDSRE___	R	T	N	D	R	E	1	1	3	2	2	1	1	0	0	1	1	0	1	1	2	0	2	0	0	2	0	0	21	0	2493.109	sp|P02751	sp|P02751|FIN	2361	2381	yes	yes	2;3	1.49E-140	226.24	3377	153	3448;3449	535125;5351	253248;25	253250 36	53
TPAQFDADELR	EVVLALLKTPAQFDAD	QFDADELRAAMKGLGT	K	T	P	L	R	A	2	1	0	2	0	1	1	0	0	0	1	0	0	1	1	0	1	0	0	0	0	0	11	0	1261.594	sp|P04083	sp|P04083|AN	114	124	yes	yes	2	0.016262	60.518	3378	173	3450	535264	253306	253306	1
TPAYYPNAGLIK	MTPHSHSRTPAYYPNA	YPNAGLIKNYCRNPDP	R	T	P	I	K	N	2	0	1	0	0	0	0	1	0	1	1	1	0	0	2	0	1	0	2	0	0	0	12	0	1306.692	sp|P08519	sp|P08519|AP	3483	3494	yes	yes	2	6.92E-08	105.36	3379	230	3451	535265;5352	253307;25	253314	9
TPCEEILVK	LVGVHGPRTPCEEILV	PCEEILVKHVGSRLYS	R	T	P	V	K	H	0	0	0	0	1	0	2	0	0	1	1	1	0	0	1	0	1	0	0	1	0	0	9	0	1087.558	sp|P21333	sp|P21333|FLN	2599	2607	yes	yes	2	0.000148	124.08	3380	310	3452	535293;5352	253316;25	253320	24
TPCQQELDQVLER	KLRPPPARTPCQQELD	ELDQVLERISTMRLPD	R	T	P	E	R	I	0	1	0	1	1	3	2	0	0	0	2	0	0	0	1	0	1	0	0	1	0	0	13	0	1614.767	sp|P18065	sp|P18065|IBP	225	237	yes	yes	2	3.58E-16	123.67	3381	298	3453	535365;5353	253340;25	253340	10
TPCTVSCNIPVVSGK	SAQMEYCRTPCTVSCN	NIPVVSGKECEEIIRK	R	T	P	G	K	E	0	0	1	0	2	0	0	1	0	1	0	1	0	0	2	2	2	0	0	3	0	0	15	0	1617.786	sp|P02675	sp|P02675|FIB	225	239	yes	yes	2	1.20E-58	189.24	3382	143	3454	535464;5354	253352;25	253489	152
TPDGSLLVR	LASVSVSRTPDGSLLV	PDGSLLVRQKAGVQVW	R	T	P	V	R	Q	0	1	0	1	0	0	0	1	0	0	2	0	0	0	1	1	1	0	0	1	0	0	9	0	956.5291	sp|Q9Y6R7	sp|Q9Y6R7|FC	5333	5341	yes	yes	2	0.0003361	130.22	3383	559	3455	535661;5356	253505;25	253505	13
TPEGLTLLFK	YQLLQVQRTPEGLTLL	EGLTLLFKRPFGTCDP	R	T	P	F	K	R	0	0	0	0	0	0	1	1	0	0	3	1	0	1	1	0	2	0	0	0	0	0	10	0	1117.638	sp|P09172	sp|P09172|DO	139	148	yes	yes	2	4.20E-12	148.62	3384	238	3456	535781;5357	253518;25	253524	117
TPENFPCK	QTPHTHNRTPENFPCK	TPENFPCKNLDENYCR	R	T	P	C	K	N	0	0	1	0	1	0	1	0	0	0	0	1	0	1	2	0	1	0	0	0	0	0	8	0	991.4433	sp|P00747	sp|P00747|PL	310	317	yes	no	2	4.32E-15	163.67	3385	87	3457	535938;5359	253635;25	253644	44
TPENYPNAGLTMNYCR	MTPHRHQKTPENYPNA	GLTMNYCRNPDADKGP	K	T	P	C	R	N	1	1	3	0	1	0	1	1	0	0	1	0	1	0	2	0	2	0	2	0	0	0	16	0	1899.824	sp|P00747	sp|P00747|PL	412	427	yes	no	2	4.38E-86	171.32	3386	87	3458	536130;5361	253679;25	253694	27
TPENYPNAGLTR	MTPHQHSRTPENYPNA	YPNAGLTRNYCRNPDA	R	T	P	T	R	N	1	1	2	0	0	0	1	1	0	0	1	0	0	0	2	0	2	0	1	0	0	0	12	0	1331.647	sp|P08519	sp|P08519|AP	3597	3608	yes	yes	2	6.55E-14	123.42	3387	230	3459	536243;5362	253706;25	253707	4
TPEVTCVVVDVSHEDPEVK	KDTLMISRTPEVTCVV	SHEDPEVKFNWYVDGV	R	T	P	V	K	F	0	0	0	2	1	0	3	0	1	0	0	1	0	0	2	1	2	0	0	6	0	0	19	0	2138.02	sp|P01857	sp|P01857|IG	139	157	yes	yes	2;3	3.75E-74	190.03	3388	124	3460	536263;5362	253710;25	253714	188
TPEVTCVVVDVSHEDPEVQFK	KDTLMISRTPEVTCVV	EDPEVQFKWYVDGVEV	R	T	P	F	K	W	0	0	0	2	1	1	3	0	1	0	0	1	0	1	2	1	2	0	0	6	0	0	21	0	2413.147	sp|P01860	sp|P01860|IG	186	206	yes	yes	2;3;4	6.51E-249	232.13	3389	126	3461	536584;5365	253898;25	254039	144
TPFDYCALR	WCYTMDPRTPFDYCAL	PFDYCALRRCADDQPP	R	T	P	L	R	R	1	1	0	1	1	0	0	0	0	0	1	0	0	1	1	0	1	0	1	0	0	0	9	0	1141.523	sp|P26927	sp|P26927|HG	438	446	yes	no	2	8.18E-26	173.68	3390	330	3462	536821;5368	254042;25	254053	66
TPGAAANLELIFVGPQHAGNYR	TKAVKTVRTPGAAANL	PQHAGNYRCRYRSWVP	R	T	P	Y	R	C	4	1	2	0	0	1	1	3	1	1	2	0	0	1	2	0	1	0	1	1	0	0	22	0	2295.176	sp|P04217	sp|P04217|A1	448	469	yes	yes	2;3;4	2.52E-268	287.54	3391	177	3463	536983;5369	254108;25	254308	219
TPLGDTTHTCPR	VDKRVELKTPLGDTTH	DTTHTCPRCPEPKSCD	K	T	P	P	R	C	0	1	0	1	1	0	0	1	1	0	1	0	0	0	2	0	4	0	0	0	0	0	12	0	1354.63	sp|P01860	sp|P01860|IG	102	113	yes	yes	2;3	4.71E-40	164.97	3392	126	3464	537417;5374	254328;25	254332	10
TPLTATLSK	TAAYPESKTPLTATLS	PLTATLSKSGNTFRPE	K	T	P	S	K	S	1	0	0	0	0	0	0	0	0	0	2	1	0	0	1	1	3	0	0	0	0	0	9	0	930.5386	sp|P01876	sp|P01876|IG	213	221	yes	yes	2	0.0007445	122.19	3393	129	3465	537494;5374	254338;25	254338	42
TPSCGDICNFPPK	GDGTWSPRTPSCGDIC	DICNFPPKIAHGHYKQ	R	T	P	P	K	I	0	0	1	1	2	0	0	1	0	1	0	1	0	1	3	1	1	0	0	0	0	0	13	0	1491.649	sp|P04003	sp|P04003|C4	419	431	yes	yes	2	3.32E-27	137.89	3395	167	3467	538097;5380	254608;25	254660	74
TPSGLYLGTCER	DCDTGYTRTPSGLYLG	LYLGTCERCSCHGHSE	R	T	P	E	R	C	0	1	0	0	1	0	1	2	0	0	2	0	0	0	1	1	2	0	1	0	0	0	12	0	1352.64	sp|P98160	sp|P98160|PG	1147	1158	yes	yes	2	8.15E-21	140.49	3396	425	3468	538570;5385	254686;25	254686	3
TPSSTGPSPVFDIK	GTDGASQKTPSSTGPS	PSPVFDIKAVSISPTN	K	T	P	I	K	A	0	0	0	1	0	0	0	1	0	1	0	1	0	1	3	3	2	0	0	1	0	0	14	0	1431.725	sp|Q12913	sp|Q12913|PT	113	126	yes	yes	2	4.33E-42	155.97	3397	444	3469	538617;5386	254689;25	254706	20
TPWHVTIK	ANASDQERTPWHVTIK	TPWHVTIKPKSQETCR	R	T	P	I	K	P	0	0	0	0	0	0	0	0	1	1	0	1	0	0	1	0	2	1	0	1	0	0	8	0	980.5444	sp|P06681	sp|P06681|CO	478	485	yes	yes	3	0.0071998	65.474	3398	205	3470	538762;5387	254709;25	254714	11
TQFNNNEYSQDLDAYNTK	QTGTWKLKTQFNNNEY	DLDAYNTKDKIGVELT	K	T	Q	T	K	D	1	0	4	2	0	2	1	0	0	0	1	1	0	1	0	1	2	0	2	0	0	0	18	0	2163.935	sp|P04114	sp|P04114|AP	1985	2002	yes	yes	2;3	2.85E-236	242.4	3399	174	3471	538848;5388	254720;25	254744	246
TQILEWAAER	TELPSFPKTQILEWAA	ILEWAAERGPITSAAE	K	T	Q	E	R	G	2	1	0	0	0	1	2	0	0	1	1	0	0	0	0	0	1	1	0	0	0	0	10	0	1215.625	sp|P17813	sp|P17813|EG	144	153	yes	yes	2	1.35E-12	117.01	3400	296	3472	539226;5392	254966;25	254968	9
TQILSIK	SRTEDETRTQILSIKK	RTQILSIKKVTSEDLK	R	T	Q	I	K	K	0	0	0	0	0	1	0	0	0	2	1	1	0	0	0	1	1	0	0	0	0	0	7	0	801.496	sp|Q9NPH	sp|Q9NPH3|IL	313	319	yes	yes	2	0.040684	89.142	3401	533	3473	539355;5393	254982	254982	1
TQILVGVNK	LELGQFKKTQILVGVN	QILVGVNKDEGTAFLV	K	T	Q	N	K	D	0	0	1	0	0	1	0	1	0	1	1	1	0	0	0	0	1	0	0	2	0	0	9	0	970.5811	sp|P06276	sp|P06276|CH	343	351	yes	yes	2	0.0024487	98.458	3402	202	3474	539429;5394	254983;25	255005	92
[illegible]																																																	

© 2020 Mongan D et al. *JAMA Psychiatry*.

VPLLLSEPINIIDALEMR	TLLDSPIKVPLLLSEP	IIDALEMRDAVEKPQE	K	V	P	M	R	[illegible]
VPQTDMTFR	ANRLLDHRVPQTDMTF	PQTDMTFRHVGSKLIV	R	V	P	F	R	[illegible]
VPSLVGSFIR	MILELFSKVPSLVGSF	SLVGSFIRSQNKEDYA	K	V	P	I	R	[illegible]
VPSVYPLDR	SSVTVSCKVPSVYPLD	PSVYPLDRLEIELLKG	K	V	P	D	R	[illegible]
VPSYTLILPSLELPVLHVPR	SILGSDVRVPSYTLIL	LPVLHVPRNLKLSLPD	R	V	P	P	R	[illegible]
VPTADLEDVLPLAEDITNILSK	NLIKLAQKVPTADLED	DITNILSKCCESASED	K	V	P	S	K	[illegible]
VPTANVSVVDLTCR	KLTGMAFRVPTANVSV	SVVDLTCRLEKPAKYD	R	V	P	C	R	[illegible]
VPTGGVEEGLLER	LTWEVAGKVPTGGVEE	VEEGLLERHSNGSQSQ	K	V	P	E	R	[illegible]
VPVAVQGEDTVQSLTQGDGVAK	PDGSPAYRVPVAVQGE	TQGDGVAKLSINTHPS	R	V	P	A	K	[illegible]
VPVLMESR	FRNGSVSRVPVLMESR	VPVLMESRSFYTAYLQ	R	V	P	S	R	[illegible]
VQAAVGTSAAPVPSDNH	QWAGLVEKVQAAVGTS	APVPSDNH________	K	V	Q	N	H	[illegible]
VQDDFVEIHGK	SPEDLTVKVQDDFVEI	DFVEIHGKHNERQDDH	K	V	Q	G	K	[illegible]
VQEAHLTEDQIFYFPK	VLSGFEQRVQEAHLTE	DQIFYFPKYGFCEAAD	R	V	Q	P	K	[illegible]
VQELGHGCAALVTK	IGSHIKHRVQELGHGC	GCAALVTKAGALQCSP	R	V	Q	T	K	[illegible]
VQFELHYQEVK	VNVLPGAKVQFELHYQ	ELHYQEVKWRKLGSYE	K	V	Q	V	K	[illegible]
VQGNDHSATR	DDGRRTLRVQGNDHSA	GNDHSATRERRLDYQE	R	V	Q	T	R	[illegible]
VQGVEFSHR	YKADTVAKVQGVEFSH	QGVEFSHRLNTDIAGL	K	V	Q	H	R	[illegible]
VQHIQLLQK	LKRKVIEKVQHIQLLQ	QHIQLLQKNVRAQLVD	K	V	Q	Q	K	[illegible]
VQLSPDLLATLPEPASPGR	EACESLTRVQLSPDLL	PEPASPGRQACGRRHK	R	V	Q	G	R	[illegible]
VQLYDLGLQIHK	FPLPLLKRVQLYDLGL	DLGLQIHKDFLFLGAN	R	V	Q	H	K	[illegible]
VQNECPGITR	DLVRTLYKVQNECPGI	NECPGITRVYSIGRSV	K	V	Q	T	R	[illegible]
VQPYLDDFQK	DLEEVKAKVQPYLDDF	PYLDDFQKKWQEEMEL	K	V	Q	Q	K	[illegible]
VQQNVPSGTDTGDPQSK	RAEEAGARVQQNVPSG	DTGDPQSKPLGDWAAG	R	V	Q	S	K	[illegible]
VQQPDCR	SCEQRAARVQQPDCRE	RVQQPDCREPFLSCCQ	R	V	Q	C	R	[illegible]
VQSGTWVGYQYPGYR	QEKVSSVRVQSGTWVG	GYQYPGYRGLQYLLEK	R	V	Q	Y	R	[illegible]
VQSTELCAGHLAGGTDSCQGDSGGPLVCFEK	RYEFLNGRVQSTELCA	GPLVCFEKDKYILQGV	R	V	Q	E	K	[illegible]
VQSTITSR	QVTLYSYKVQSTITSR	VQSTITSRMATTMIQS	K	V	Q	S	R	[illegible]
VQVAFPFDEYFQIECEAK	PTIIKQSKVQVAFPFD	FQIECEAKGNPEPTFS	K	V	Q	A	K	[illegible]
VQVQDNEGCPVEALVK	HGGKAPLRVQVQDNEG	CPVEALVKDNGNGTYS	R	V	Q	V	K	[illegible]
VQVVAGK	IDNVKKARVQVVAGKK	RVQVVAGKKYFIDFVA	R	V	Q	G	K	[illegible]
VQYECATGYYTAGGK	KTFKVKDKVQYECATG	GYYTAGGKKTEEVECL	K	V	Q	G	K	[illegible]
VSALLTPAEQTGTWK	ISAALEHKVSALLTPA	AEQTGTWKLKTQFNNN	K	V	S	W	K	[illegible]
VSEADSSNADWVTK	TYPKIWVKVSEADSSN	SNADWVTKQLNEINYE	K	V	S	T	K	[illegible]
VSEGNHDIALIK	IIIHQNYKVSEGNHDI	NHDIALIKLQAPLNYT	K	V	S	I	K	[illegible]
VSFELFADK	VDGEPLGRVSFELFAD	SFELFADKVPKTAENF	R	V	S	D	K	[illegible]
VSFLSALEEYTK	LPVLESFKVSFLSALE	SALEEYTKKLNTQ___	K	V	S	T	K	[illegible]
VSHSEDDCLAFK	TLIIYLDKVSHSEDDC	EDDCLAFKVHQYFNVE	K	V	S	F	K	[illegible]
VSITSITVENVFVK	PEIAYAYKVSITSITV	TVENVFVKYKATLLDI	K	V	S	V	K	[illegible]
VSLATVDK	KDEDFCKRVSLATVDK	VSLATVDKTVETPSPH	R	V	S	D	K	[illegible]
VSMMQTK	LNEREVVKVSMMQTKG	KVSMMQTKGNFLAAND	K	V	S	T	K	[illegible]
VSQEGLK	SKNIFNFKVSQEGLKL	KVSQEGLKLSNDMMGS	K	V	S	L	K	[illegible]
VSQMAQYFEPLTLAAVGAASK	EASQLGHKVSQMAQYF	AAVGAASKTLSHPQQM	K	V	S	S	K	[illegible]
VSQYIEWLQK	GHFGVYTRVSQYIEWL	QYIEWLQKLMRSEPRP	R	V	S	Q	K	[illegible]
VSSVEECQK	GVNFNVSKVSSVEECQ	SSVEECQKRCTNNIRC	K	V	S	Q	K	[illegible]
VSTAFVYTK	IGRRQHLRVSTAFVYT	STAFVYTKNPNGYSFS	R	V	S	T	K	[illegible]
VSVFVPPR	VIAELPPKVSVFVPPR	VSVFVPPRDGFFGNPR	K	V	S	P	R	[illegible]
VSVGGEK	DDKEHSIKVSVGGEKR	KVSVGGEKRDLEIEVV	K	V	S	E	K	[illegible]
VSVGLLLVK	DHEITGLRVSVGLLLV	SVGLLLVKSVQVKLGD	R	V	S	V	K	[illegible]
VSVHPDYR	LGNHPIRRVSVHPDYR	VSVHPDYRQDESYNFE	R	V	S	Y	R	[illegible]
VSVLCQENYLIQEGEEITCK	LNYRDGEKVSVLCQEN	EGEEITCKDGRWQSIP	K	V	S	C	K	[illegible]
VSVQLEASPAFLAVPVEK	NYLPKCIRVSVQLEAS	FLAVPVEKEQAPHCIC	R	V	S	E	K	[illegible]
VSYVGLVTVR	NEHRGSRRVSYVGLVT	YVGLVTVRAYSHSVSL	R	V	S	V	R	[illegible]
VTAAPQSVCALR	PASHAHLRVTAAPQSV	PQSVCALRAVDQSVLL	R	V	T	L	R	[illegible]
VTAQGPGLEPSGNIANK	KSQGDASKVTAQGPGL	PSGNIANKTTYFEIFT	K	V	T	N	K	[illegible]
VTASDPLDTLGSEGALSPGGVASLLR	GDFNSYVRVTASDPLD	GGVASLLRLPRGCGEQ	R	V	T	L	R	[illegible]
VTCTNAELVK	SEITFIKKVTCTNAEL	CTNAELVKGRQYLIMG	K	V	T	V	K	[illegible]
VTDATETTITISWR	VSPPRRARVTDATETT	TTITISWRTKTETITG	R	V	T	W	R	[illegible]
VTEIWQEVMQR	WIRDMYAKVTEIWQEV	IWQEVMQRRDDDGALH	K	V	T	Q	R	[illegible]
VTEPISAESGEQVER	HASASRPRVTEPISAE	ESGEQVERVNEPSILE	R	V	T	E	R	[illegible]
VTFQLTYEEVLK	LTVNPQSKVTFQLTYE	LTYEEVLKRNHMQYEI	K	V	T	L	K	[illegible]
VTGEGCVYLQTSLK	LPGEYSMKVTGEGCVY	VYLQTSLKYNILPEKE	K	V	T	L	K	[illegible]
VTGESHIGGVLLK	EAESVTLRVTGESHIG	HIGGVLLKIVEQINRK	R	V	T	L	K	[illegible]
VTGLIEGSDYQFR	MTLGVSYKVTGLIEGS	EGSDYQFRVYAINAAG	K	V	T	F	R	[illegible]
VTGVVLFR	TLDAAQPRVTGVVLFR	VTGVVLFRQLAPRAKL	R	V	T	F	R	[illegible]
VTGWGNLK	LQAGYKGRVTGWGNLK	VTGWGNLKETWTANVG	R	V	T	L	K	[illegible]
VTIAQGGVLPNIQAVLLPK	ELNKLLGKVTIAQGGV	IQAVLLPKKTESHHKT	K	V	T	P	K	[illegible]
VTIGLLFWDGR	LLLSDPDKVTIGLLFW	GLLFWDGRGEGLRLLL	K	V	T	G	R	[illegible]
VTIKPAPETEK	TCNKFDLKVTIKPAPE	KPAPETEKRPQDAKNT	K	V	T	E	K	[illegible]
VTIMWTPPESAVTGYR	FVEVTDVKVTIMWTPP	ESAVTGYRVDVIPVNL	K	V	T	Y	R	[illegible]
VTIPTDLIASSGDIIK	AVVGRSFRVTIPTDLI	ASSGDIIKVSAAGKEA	R	V	T	I	K	[illegible]
VTISVDTSK	YNPSLKSRVTISVDTS	TISVDTSKNQFSLKLS	R	V	T	S	K	[illegible]
VTITCQASQDIR	LSASVGDRVTITCQAS	CQASQDIRKHLNWYDQ	R	V	T	I	R	[illegible]
VTLTCVAPLSGVDFQLR	QVLHPGNKVTLTCVAP	SGVDFQLRRGEKELLV	K	V	T	L	R	[illegible]
VTLTSEEEAR	NGISDLVKVTLTSEEE	LTSEEEARLKKSADTL	K	V	T	A	R	[illegible]
VTPPEGYEVVTVFPK	CEKHARERVTPPEGYE	EVVTVFPK________	R	V	T	P	K	[illegible]
VTQEFHMK	NVFDGLVRVTQEFHMK	VTQEFHMKVKHLIDSL	R	V	T	M	K	[illegible]
VTSIQDWVQK	AEYGVYVKVTSIQDWV	SIQDWVQKTIAEN___	K	V	T	Q	K	[illegible]
VTSTLTIK	ESGPTTYKVTSTLTIK	VTSTLTIKESDWLGQS	K	V	T	I	K	[illegible]
VTTSQDMLSIMEK	LLTLPRIKVTTSQDML	DMLSIMEKLEFFDFSY	K	V	T	E	K	[illegible]
VTTVASHTSDSDVPSGVTEVVVK	GEDQYYLRVTTVASHT	GVTEVVVKLFDSDPIT	R	V	T	V	K	[illegible]
VTVFPIGIGDR	ADAARSNRVTVFPIGI	FPIGIGDRYDAAQLRI	R	V	T	D	R	[illegible]
VTVLFAGQHIAK	PEVTGTHKVTVLFAGQ	FAGQHIAKSPFEVYVD	K	V	T	A	K	[illegible]
VTVSCSGGMSLEGPSAFLCGSSLK	PFYTVGEKVTVSCSGG	FLCGSSLKWSPEMKNA	K	V	T	L	K	[illegible]
VTWAPPPSIDLTNFLVR	NIGPDTMRVTWAPPPS	DLTNFLVRYSPVKNEE	R	V	T	V	R	[illegible]
VTWDSSFCAVNPR	YDDIRVSRVTWDSSFC	SFCAVNPRFVAIIIEA	R	V	T	P	R	[illegible]
VTYCPTEPGNYIINIK	DLEDGTCRVTYCPTEP	GNYIINIKFADQHVPG	R	V	T	I	K	[illegible]
VTYDVSR	SLLNGHFKVTYDVSRD	KVTYDVSRDKICDLLV	K	V	T	S	R	[illegible]
VTYTSQEDLVEK	CFHLRFCKVTYTSQED	SQEDLVEKKCLAKKYT	K	V	T	E	K	[illegible]
VVAEGFDFANGINISPDGK	YYSPSEVRVVAEGFDF	INISPDGKYVYIAELL	R	V	V	G	K	[illegible]
VVAGVANALAHK	PVQAAYQKVVAGVANA	VANALAHKYH______	K	V	V	H	K	[illegible]
VVAPTISSPVCQEQLVEAGR	SQLVACTKVVAPTISS	EQLVEAGRLVAKAVEG	K	V	V	G	R	[illegible]
VVAQGVGIPEDSIFTMADR	ETLLQDFRVVAQGVGI	SIFTMADRGECVPGEQ	R	V	V	D	R	[illegible]
VVATTQMQAADAR	YMEGNVRKVVATTQMQ	QMQAADARKSFPCFDE	K	V	V	A	R	[illegible]
VVCSCTEGYR	CKNSADNKVVCSCTEG	CSCTEGYRLAENQKSC	K	V	V	Y	R	[illegible]
VVDVLDSIK	SAKPGLQKVVDVLDSI	VDVLDSIKTKGKSADF	K	V	V	I	K	[illegible]
VVEEQESR	RRRREAPKVVEEQESR	VVEEQESRVHYTVCIW	K	V	V	S	R	[illegible]
VVEESELAR	PRPPCRDRVVEESELA	VEESELARTAGYGINI	R	V	V	A	R	[illegible]
VVFLNTQLCQFR	GSNPNLTKVVFLNTQL	NTQLCQFRPDAFGGLP	K	V	V	F	R	[illegible]
VVFLNTQLCQFRPDAFGGLPR	GSNPNLTKVVFLNTQL	DAFGGLPRLEDLEVTG	K	V	V	P	R	[illegible]
VVFQEFR	CRQGFPNRVVFQEFRQ	RVVFQEFRQRYEILTP	R	V	V	F	R	[illegible]
VVFVFGPDK	KGMPVTARVVFVFGPD	VFVFGPDKKLKLSILY	R	V	V	D	K	[illegible]
VVGGCVAHPHSWPWQVSLR	EPKKCPGRVVGGCVAH	WPWQVSLRTRFGMHFC	R	V	V	L	R	[illegible]
VVGGHPGNSPWTVSLR	DQRRSKLRVVGGHPGN	SPWTVSLRNRQGQHFC	R	V	V	L	R	[illegible]
VVGGLVALR	KSLSSMTRVVGGLVAL	VGGLVALRGAHPYIAA	R	V	V	L	R	[illegible]
VVLAGGVAPALFR	EELYDLSKVVLAGGVA	GVAPALFRGMPAHFSD	K	V	V	F	R	[illegible]
VVLAYEPVWAIGTGK	DNVKDWSKVVLAYEPV	VWAIGTGKTATPQQAQ	K	V	V	G	K	[illegible]
VVLEGGIDPILR	RVFFASWRVVLEGGID	GGIDPILRGLMATPAK	R	V	V	L	R	[illegible]
VVLGDQDLK	DIKTRHLKVVLGDQDL	VLGDQDLKKEEFHEQS	K	V	V	L	K	[illegible]
VVLHPNYSQVDIGLIK	KQLVEIEKVVLHPNYS	QVDIGLIKLKQKVSVN	K	V	V	I	K	[illegible]
VVLLPSCPGAPGSPGEK	PRELEASKVVLLPSCP	APGSPGEKGAPGPQGP	K	V	V	E	K	[illegible]
VVNNSPQPQNVVFDVQIPK	ATTMIQSKVVNNSPQP	VFDVQIPKGAFISNFS	K	V	V	P	K	[illegible]
VVPEGIR	DGVRKSLKVVPEGIRM	KVVPEGIRMNKTVAVR	K	V	V	I	R	[illegible]
VVPEGVK	EILVKTLRVVPEGVKR	RVVPEGVKRESYSGVT	R	V	V	V	K	[illegible]
VVSVDENFR	PGQTVRFRVVSVDENF	VSVDENFRPRNELIPL	R	V	V	F	R	[illegible]
VVSVDENFRPR	PGQTVRFRVVSVDENF	VDENFRPRNELIPLIY	R	V	V	P	R	[illegible]
VVSVLTVLHQDWLNGK	EQYNSTFRVVSVLTVL	HQDWLNGKEYKCKVSN	R	V	V	G	K	[illegible]
VVSVLTVVHQDWLNGK	EQFNSTFRVVSVLTVV	HQDWLNGKEYKCKVSN	R	V	V	G	K	[illegible]
VVTFCGHTSK	LLYRSGVKVVTFCGHT	TFCGHTSKTNQVNSGG	K	V	V	S	K	[illegible]
VVTPLSPPTNLHLEANPDTGVLTVSWER	RDAPIVNKVVTPLSPP	VLTVSWERSTTPDITG	K	V	V	E	R	[illegible]
VVTVLVR	HTHLTGRKVVTVLVRD	KVVTVLVRDGREWEIV	K	V	V	V	R	[illegible]
VVVAENFDEIVNNENK	ESNDGPVKVVVAENFD	EIVNNENKDVLIEFYA	K	V	V	N	K	[illegible]
VVVHPDYR	LGNHPVHRVVVHPDYR	VVVHPDYRQNESHNFS	R	V	V	Y	R	[illegible]
VVVNFAPTIQEIK	FPDVRKVKVVVNFAPT	APTIQEIKSGTVTPGR	K	V	V	I	K	[illegible]
VVVQTESGGR	EPIQGSYRVVVQTESG	VQTESGGRIQHPFTVE	R	V	V	G	R	[illegible]
VWFELTQGSITK	AELQKGERVWFELTQG	LTQGSITKRSLSGTAF	R	V	W	T	K	[illegible]
VWLEVEPVGMLK	PVFYPTEKVWLEVEPV	VEPVGMLKEGDRVEIR	K	V	W	L	K	[illegible]
VWNYFQR	PMYPAFKRVWNYFQRV	RVWNYFQRVLVKKYAS	R	V	W	Q	R	[illegible]
VWPQQPSGELFEIEIDTLETTCHVLDPTPVAR	LNQIDEVKVWPQQPSG	LDPTPVARCSVRQLKE	K	V	W	A	R	[illegible]
VWSPLVTEEGK	TVARSEHKVWSPLVTE	PLVTEEGKRHPYKMNL	K	V	W	G	K	[illegible]
VWVQAFK	AFSVNIFKVWVQAFKV	KVWVQAFKVEGGQFGS	K	V	W	F	K	[illegible]
VWVYPPEK	VFLIKGDKVWVYPPEK	VWVYPPEKKEKGYPKL	K	V	W	E	K	[illegible]
VYACEVTHQGLSSPVTK	KADYEKHKVYACEVTH	GLSSPVTKSFNRGEC_	K	V	Y	T	K	[illegible]
VYALPEDLVEVNPK	MARKIGARVYALPEDL	DLVEVNPKMVMTVFAC	R	V	Y	P	K	[illegible]
VYAYYNLEESCTR	LIQPGAVKVYAYYNLE	NLEESCTRFYHPEKED	K	V	Y	T	R	[illegible]
VYCDMNTENGGWTVIQNR	DSSVKPYRVYCDMNTE	GWTVIQNRQDGSVDFG	R	V	Y	N	R	[illegible]
VYDYYETDEFAIAEYNAPCSK	DLKPAIVKVYDYYETD	EYNAPCSKDLGNA___	K	V	Y	S	K	[illegible]
VYFAGFPR	ENGLLETKVYFAGFPR	VYFAGFPRKVESELIK	K	V	Y	P	R	[illegible]
VYIASSSGSTAIK	_________________	SSGSTAIKKKQQDVLG	R	V	Y	I	K	[illegible]
VYLIYGNDLGLPPVDLDLDK	PGHIHIGRVYLIYGND	PVDLDLDKEAHRILEG	R	V	Y	D	K	[illegible]
VYLSECK	DVVLFEKKVYLSECKT	KVYLSECKTGNGKNYR	K	V	Y	C	K	[illegible]
VYPGEQYTYMLLATEEQSPGEGDGNCVTR	DFQRADDKVYPGEQYT	GDGNCVTRIYHSHIDA	K	V	Y	T	R	[illegible]
VYSLNDDLK	PDQSVKVRVYSLNDDL	YSLNDDLKPAKRETVL	R	V	Y	L	K	[illegible]
VYSLNDDLKPAK	PDQSVKVRVYSLNDDL	NDDLKPAKRETVLTFI	R	V	Y	A	K	[illegible]
VYSTSVTGSR	_________________	STSVTGSREIKSQQSE	R	V	Y	S	R	[illegible]
WAAVVVPSGEEQR	AGDGTFQKWAAVVVPS	VPSGEEQRYTCHVQHE	K	W	A	Q	R	[illegible]
WADLSGITK	GFTDLFSKWADLSGIT	ADLSGITKQQKLEASK	K	W	A	T	K	[illegible]
WALEEYLDEFDPCHCR	SVKKLYLKWALEEYLD	EFDPCHCRPCQNGGLA	K	W	A	C	R	[illegible]
WALSQSNPSALR	AVTMDDFRWALSQSNP	QSNPSALRETVVEVPQ	R	W	A	L	R	[illegible]
WANQCNYR	EAAANAQKWANQCNYR	WANQCNYRHSNPKDRM	K	W	A	Y	R	[illegible]
WCALSHHER	TDECKPVKWCALSHHE	CALSHHERLKCDEWSV	K	W	C	E	R	[illegible]
WCATTHNYDR	SEGSAHRKWCATTHNY	ATTHNYDRDRAWGYCV	K	W	C	D	R	[illegible]
WCAVSEHEATK	AVPDKTVRWCAVSEHE	VSEHEATKCQSFRDHM	R	W	C	T	K	[illegible]
WCGTTQNYDADQK	EGRRDNMKWCGTTQNY	QNYDADQKFGFCPMAA	K	W	C	Q	K	[illegible]
WCHDNGVNYK	HFRCDSSRWCHDNGVN	HDNGVNYKIGEKWDRQ	R	W	C	Y	K	[illegible]
WDEELAAFAK	ASDMLHMRWDEELAAF	EELAAFAKAYARQCVW	R	W	D	A	K	[illegible]
WDSWTSSR	FEKGEYPRWDSWTSSR	WDSWTSSRRTDSLSSL	R	W	D	S	R	[illegible]
WEAEPVYVQR	AAQITKQKWEAEPVYV	AEPVYVQRAKAYLEEE	K	W	E	Q	R	[illegible]
WEDILSDEVNVAR	LFKFRNNRWEDILSDE	SDEVNVARGVASLFAG	R	W	E	A	R	[illegible]
WELALGR	TEWQSGQRWELALGRF	RWELALGRFWDYLRWV	R	W	E	G	R	[illegible]
WELCDIPR	FTTDPNKRWELCDIPR	WELCDIPRCTTPPPSS	R	W	E	P	R	[illegible]
WELDLDIK	YDLRRWEKWELDLDIK	WELDLDIKEVFVHPNY	K	W	E	I	K	[illegible]
WEMPFDPQDTHQSR	NYIFFKAKWEMPFDPQ	PQDTHQSRFYLSKKKW	K	W	E	S	R	[illegible]
WETSFNHK	NYIFFKAKWETSFNHK	WETSFNHKGTQEQDFY	K	W	E	H	K	[illegible]
WEYCNLK	FTTDPSVRWEYCNLKK	RWEYCNLKKCSGTEAS	R	W	E	L	K	[illegible]
WEYYDSVYTER	IAVAPVSRWEYYDSVY	YDSVYTERYMGLPTPE	R	W	E	E	R	[illegible]
WFLLEQPEIQVAHFPFK	QARTYPLRWFLLEQPE	QVAHFPFKNNMSFVVL	R	W	F	F	K	[illegible]
WFYIASAFR	TLDQITGKWFYIASAF	FYIASAFRNEEYNKSV	K	W	F	F	R	[illegible]
WGAAPYR	PAIHPRCRWGAAPYRG	RWGAAPYRGRPKLLQL	R	W	G	Y	R	[illegible]
WGDEHIPGSPYR	GEYTLVVKWGDEHIPG	HIPGSPYRVVVP____	K	W	G	Y	R	[illegible]
WGTVCDDGWDLR	LEVWHGGRWGTVCDDG	CDDGWDLRDAAVACRQ	R	W	G	L	R	[illegible]
WIDNPTVDDR	GKIEQAQRWIDNPTVD	DNPTVDDRGVGQAAIR	R	W	I	D	R	[illegible]
WIISSLTPK	MTVGPEGKWIISSLTP	IISSLTPKHLQAGMQA	K	W	I	P	K	[illegible]
WILTAAHTLYPK	GGALLGDRWILTAAHT	AAHTLYPKEHEAQSNA	R	W	I	P	K	[illegible]
WIVTAAHCVETGVK	GGSIVNEKWIVTAAHC	HCVETGVKITVVAGEH	K	W	I	V	K	[illegible]
WIYHLTEGSTDLR	DGLCVPRKWIYHLTEG	TEGSTDLRTEGRPDMK	K	W	I	L	R	[illegible]
WKETLFSVMPGLK	NRRSSAYKWKETLFSV	FSVMPGLKMTMDKTGL	K	W	K	L	K	[illegible]
WKNFPSPVDAAFR	DRELISERWKNFPSPV	SPVDAAFRQGHNSVFL	R	W	K	F	R	[illegible]
WLDLSHNR	FLGLKALRWLDLSHNR	WLDLSHNRVAGLLEDT	R	W	L	N	R	[illegible]
WLEVINITK	LYPPSPPRWLEVINIT	LEVINITKNTADLKWT	R	W	L	T	K	[illegible]
WLPSSSPVTGYR	QDNSISVKWLPSSSPV	SSPVTGYRVTTTPKNG	K	W	L	Y	R	[illegible]
WLQDNAENVYVWK	CEILYFRRWLQDNAEN	AENVYVWKQGVDVKAM	R	W	L	W	K	[illegible]
WLQGSQELPR	SPKDVLVRWLQGSQEL	QGSQELPREKYLTWAS	R	W	L	P	R	[illegible]
WLSEEQR	NYVNPVIKWLSEEQRY	KWLSEEQRYGGGFYST	K	W	L	Q	R	[illegible]
WLVGEMHCQK	ARCGEDLRWLVGEMHC	VGEMHCQKIACVLPVL	R	W	L	Q	K	[illegible]
WLVLCNPGLAEVIAER	TNGITPRRWLVLCNPG	LAEVIAERIGEDFISD	R	W	L	E	R	[illegible]
WNFYYSPQSSPDK	DEDDDFSKWNFYYSPQ	SPQSSPDKKLTIFKTE	K	W	N	D	K	[illegible]
WNFYYSPQSSPDKK	DEDDDFSKWNFYYSPQ	PQSSPDKKLTIFKTEL	K	W	N	K	K	[illegible]
WNILEFDEPTENDAQCLTR	PGETYTYKWNILEFDE	NDAQCLTRPYYSDVDI	K	W	N	T	R	[illegible]
WNPCLEPHR	MPSPQVVRWNPCLEPH	NPCLEPHRFNDTEVLQ	R	W	N	H	R	[illegible]
WNPEVDCTEK	HSVCINGKWNPEVDCT	PEVDCTEKREQFCPPP	K	W	N	E	K	[illegible]
WNPSPPACEPNSCINLPDIPHASWETYPR	IHCDADSKWNPSPPAC	ASWETYPRPTKEDVYV	K	W	N	P	R	[illegible]
WNTTLYEGTWR	LKSRTIRKWNTTLYEG	TLYEGTWRRGSTAGGC	K	W	N	W	R	[illegible]
WNYIEGTK	SQNEKINRWNYIEGTK	WNYIEGTKLFAAFFLE	R	W	N	T	K	[illegible]
WPEPVFGR	VATPLGPKWPEPVFGR	WPEPVFGRLASPGFPG	K	W	P	G	R	[illegible]
WQEEMELYR	YLDDFQKKWQEEMELY	QEEMELYRQKVEPLRA	K	W	Q	Y	R	[illegible]
WQSIPLCVEK	EITCKDGRWQSIPLCV	SIPLCVEKIPCSQPPQ	R	W	Q	E	K	[illegible]
WSAGLTSSQVDLYIPK	LSRDTINRWSAGLTSS	QVDLYIPKVTISGVYD	R	W	S	P	K	[illegible]
WSGQTAICDNGAGYCSNPGIPIGTR	RTCQVNGRWSGQTAIC	PGIPIGTRKVGSQYRL	R	W	S	T	R	[illegible]
WSHPPSCIK	IAKCLGEKWSHPPSCI	SHPPSCIKTDCLSLPS	K	W	S	I	K	[illegible]
WSPELPVCAPIICPPPSIPTFATLR	AKCTEEGKWSPELPVC	IPTFATLRVYKPSAGN	K	W	S	L	R	[illegible]
WSRPQAPITGYR	DDTSIVVRWSRPQAPI	QAPITGYRIVYSPSVE	R	W	S	Y	R	[illegible]
WSSPPQCEGLPCK	ETTCYMGKWSSPPQCE	QCEGLPCKSPPEISHG	K	W	S	C	K	[illegible]
WSSTSPHRPR	KNGITCQKWSSTSPHR	STSPHRPRFSPATHPS	K	W	S	P	R	[illegible]
WTGRPTCR	NVTCINSRWTGRPTCR	WTGRPTCRDTSCVNPP	R	W	T	C	R	[illegible]
WTLTAPPGYR	YANDQERRWTLTAPPG	LTAPPGYRLRLYFTHF	R	W	T	Y	R	[illegible]
WTPYQGCEALCCPEPK	VICQKNLRWTPYQGCE	ALCCPEPKLNNGEITQ	R	W	T	P	K	[illegible]
WVLTAAHCLLYPPWDK	GASLISDRWVLTAAHC	LLYPPWDKNFTENDLL	R	W	V	D	K	[illegible]
WVNLPEESLLR	QAPTNYSRWVNLPEES	LPEESLLRPAYVVPWQ	R	W	V	L	R	[illegible]
WVQTLSEQVQEELLSSQVTQELR	GRFWDYLRWVQTLSEQ	SQVTQELRALMDETMK	R	W	V	L	R	[illegible]
WYNLAIGSTCPWLK	NISRIYGKWYNLAIGS	GSTCPWLKKIMDRMTV	K	W	Y	L	K	[illegible]
WYQMGIVSWGEGCDR	MKSPFNNRWYQMGIVS	SWGEGCDRDGKYGFYT	R	W	Y	D	R	[illegible]
WYVDGVEVHNAK	EDPEVQFKWYVDGVEV	GVEVHNAKTKPREEQY	K	W	Y	A	K	[illegible]
YAASSYLSLTPEQWK	PSKQSNNKYAASSYLS	SLTPEQWKSHKSYSCQ	K	Y	A	W	K	[illegible]
YAASSYLSLTPEQWR	PSKQSNNKYAASSYLS	SLTPEQWRSRRSYSCQ	K	Y	A	W	R	[illegible]
YAATSQVLLPSK	PSVLRGGKYAATSQVL	SQVLLPSKDVMQGTDE	K	Y	A	S	K	[illegible]
YADAQLSCQGR	LLVKEEKRYADAQLSC	AQLSCQGRGGTLSMPK	R	Y	A	G	R	[illegible]
YAFVNWINK	HSYSEEEKYAFVNWIN	AFVNWINKALENDPDC	K	Y	A	N	K	[illegible]
YAGSQVASTSEVLK	RRIASQVKYAGSQVAS	ASTSEVLKYTLFQIFS	K	Y	A	L	K	[illegible]
YAISMAR	DEKLNNAKYAISMARK	KYAISMARKIGARVYA	K	Y	A	A	R	[illegible]
YALYDATYETK	MLPDKDCRYALYDATY	YDATYETKESKKEDLV	R	Y	A	T	K	[illegible]
YAPLSFRNPMMSK	AEMEEELRYAPLSFRN	FRNPMMSKLRNYRKDL	R	Y	A	S	K	[illegible]
YAVLYQPLFDK	EVHDLERKYAVLYQPL	LYQPLFDKRFEIINAI	K	Y	A	D	K	[illegible]
YAVSEAAAHK	YRSNLNGRYAVSEAAA	VSEAAAHKYGIDWASG	R	Y	A	H	K	[illegible]
YAYLLQPSQFHGEPCNFSDK	PCQKKRYRYAYLLQPS	EPCNFSDKEVEDCVTN	R	Y	A	D	K	[illegible]
YCFEIVPK	GGLFPGTKYCFEIVPK	YCFEIVPKGPNGTEGA	K	Y	C	P	K	[illegible]
YCQVVCTYHPR	TVFTPSAKYCQVVCTY	VVCTYHPRCLLFTFTA	K	Y	C	P	R	[illegible]
YCTDTGVLFR	QGGEETQRYCTDTGVL	TDTGVLFRQESFFHWA	R	Y	C	F	R	[illegible]
YDAAQLR	FPIGIGDRYDAAQLRI	RYDAAQLRILAGPAGD	R	Y	D	L	R	[illegible]
YDALEVFAGSGTSGQR	LELHPACRYDALEVFA	GSGTSGQRLGRFCGTF	R	Y	D	Q	R	[illegible]
YDGGSEIINYVLESR	TLTWNPPKYDGGSEII	INYVLESRLIGTEKFH	K	Y	D	S	R	[illegible]
YDLAFVVASQATK	PTLASQSRYDLAFVVA	VVASQATKLTYNHGGI	R	Y	D	T	K	[illegible]
YDLLDLTR	LATSPAFRYDLLDLTR	YDLLDLTRQAVQELVS	R	Y	D	T	R	[illegible]
YDLSALVR	GATDGKKRYDLSALVR	YDLSALVRHAEPEQNW	R	Y	D	V	R	[illegible]
YDPSLKPLSVSYDQATSLR	DIDTHTAKYDPSLKPL	YDQATSLRILNNGHAF	K	Y	D	L	R	[illegible]
YDVDTQMWTILK	RLADDLYRYDVDTQMW	TQMWTILKDSRFFRYL	R	Y	D	L	K	[illegible]
YDVENCLANK	DVIGDSAKYDVENCLA	VENCLANKVDLSFSPS	K	Y	D	N	K	[illegible]
YEASILTHDSSIR	KMLEEIMKYEASILTH	LTHDSSIRYLQEIYNS	K	Y	E	I	R	[illegible]
YEDFGPLFTAK	DNYPPSFKYEDFGPLF	FGPLFTAKFFNANQWA	K	Y	E	A	K	[illegible]
YEDGTLSLTSTSDLQSGIIK	GTNQITGRYEDGTLSL	DLQSGIIKNTASLKYE	R	Y	E	I	K	[illegible]
YEDLYSNCK	GLCTNGCKYEDLYSNC	EDLYSNCKSLKLTLTC	K	Y	E	C	K	[illegible]
YEDVNPVLLSGPEAPWR	PYFGTKTRYEDVNPVL	SGPEAPWRDPELLEGT	R	Y	E	W	R	[illegible]
YEFLNGR	IENKVCNRYEFLNGRV	RYEFLNGRVQSTELCA	R	Y	E	G	R	[illegible]
YEGLQEWEGK	AEYEEDGKYEGLQEWE	GLQEWEGKAHLNIKSP	K	Y	E	G	K	[illegible]
YEGSYALTSEEAER	LEQAQKVRYEGSYALT	LTSEEAERSDGDPVQP	R	Y	E	E	R	[illegible]
YEITTIHNLFR	DFVNASSKYEITTIHN	TTIHNLFRKLTHRLFR	K	Y	E	F	R	[illegible]
YEIVVEAR	LDREKQARYEIVVEAR	YEIVVEARDAQGLRGD	R	Y	E	A	R	[illegible]
YEKPGSPPR	RITGYIIKYEKPGSPP	EKPGSPPREVVPRPRP	K	Y	E	P	R	[illegible]
YEMHELLR	PKFKLDQKYEMHELLR	YEMHELLRQMGIRRIF	K	Y	E	L	R	[illegible]
YENYELTLK	IKNTASLKYENYELTL	ENYELTLKSDTNGKYK	K	Y	E	L	K	[illegible]
YEVDQQIQVLMDK	KVHELIERYEVDQQIQ	QIQVLMDKLVELAHQY	R	Y	E	D	K	[illegible]
YEVQGEVFTK	VLNWRALKYEVQGEVF	VQGEVFTKPQLWP___	K	Y	E	T	K	[illegible]
YEVQGEVFTKPQLWP	VLNWRALKYEVQGEVF	FTKPQLWP________	K	Y	E	W	P	[illegible]
YEVSVYALK	SGLMVATKYEVSVYAL	EVSVYALKDTLTSRPA	K	Y	E	L	K	[illegible]
YEVTVVSVR	QGLIPGARYEVTVVSV	EVTVVSVRGFEESEPL	R	Y	E	V	R	[illegible]
YEYLEGGDR	EKCFDETRYEYLEGGD	EYLEGGDRWARVRQGH	R	Y	E	D	R	[illegible]
YFDTECVPMNFR	TFFNLSKRYFDTECVP	ECVPMNFRNASQAKRL	R	Y	F	F	R	[illegible]
YFIDFVAR	VQVVAGKKYFIDFVAR	YFIDFVARETTCSKES	K	Y	F	A	R	[illegible]
YFIIQDR	SNATDPLKYFIIQDRC	KYFIIQDRCPHTRDST	K	Y	F	D	R	[illegible]
YFSTTEDYDHEITGLR	MYGPGGGKYFSTTEDY	DHEITGLRVSVGLLLV	K	Y	F	L	R	[illegible]
YGAATFTR	VALHALSKYGAATFTR	YGAATFTRTGKAAQVT	K	Y	G	T	R	[illegible]
YGFCEAADQFHVLDEVR	DQIFYFPKYGFCEAAD	FHVLDEVRR_______	K	Y	G	V	R	[illegible]
YGFIEGHVVIPR	SIGFETCRYGFIEGHV	EGHVVIPRIHPNSICA	R	Y	G	P	R	[illegible]
YGFYTHVFR	EGCDRDGKYGFYTHVF	GFYTHVFRLKKWIQKV	K	Y	G	F	R	[illegible]
YGGDEIPFSPYR	GRYTILIKYGGDEIPF	EIPFSPYRVRAVPTGD	K	Y	G	Y	R	[illegible]
YGGDEIPYSPFR	GRYTITIKYGGDEIPY	EIPYSPFRIHALPTGD	K	Y	G	F	R	[illegible]
YGHSLALYK	SVNNVVVRYGHSLALY	GHSLALYKDKIYMYGG	R	Y	G	Y	K	[illegible]
YGIDWASGR	VSEAAAHKYGIDWASG	GIDWASGRGVGHPYRR	K	Y	G	G	R	[illegible]
YGILADAR	QTHWTLDKYGILADAR	YGILADARLFFGPQHR	K	Y	G	A	R	[illegible]
YGLDSDLSCK	SDPKAVLRYGLDSDLS	LDSDLSCKIAQLPLTG	R	Y	G	C	K	[illegible]
YGLVTYATYPK	ASYGVKPRYGLVTYAT	VTYATYPKIWVKVSEA	R	Y	G	P	K	[illegible]
YGMVAQVTQTLK	LPFSYKNKYGMVAQVT	AQVTQTLKLEDTPKIN	K	Y	G	L	K	[illegible]
YGPPCPSCPAPEFLGGPSVFLFPPKPK	VDKRVESKYGPPCPSC	FLFPPKPKDTLMISRT	K	Y	G	P	K	[illegible]
YGQPLPGYTTK	GFCWCVDKYGQPLPGY	PLPGYTTKGKEDVHCY	K	Y	G	T	K	[illegible]
YGQTIRPICLPCTEGTTR	IKLKNKLKYGQTIRPI	PCTEGTTRALRLPPTT	K	Y	G	T	R	[illegible]
YGSQLAPETFYR	PGGGVLLRYGSQLAPE	LAPETFYRECDMQLFG	R	Y	G	Y	R	[illegible]
YGTCIYQGR	ACIAGERRYGTCIYQG	GTCIYQGRLWAFCC__	R	Y	G	G	R	[illegible]
YGVSGYPTLK	ANTNTCNKYGVSGYPT	VSGYPTLKIFRDGEEA	K	Y	G	L	K	[illegible]
YHFEALADTGISSEFYDNANDLLSK	RKPYNFLKYHFEALAD	NANDLLSKVKKDKSDS	K	Y	H	S	K	[illegible]
YHWEHTGLTLR	VLYDYVNKYHWEHTGL	EHTGLTLREVSSKLRR	K	Y	H	L	R	[illegible]
YIAGPYECEIR	LYLFGVTKYIAGPYEC	GPYECEIRNPVSASRS	K	Y	I	I	R	[illegible]
YIDQEELNK	KTKKIKEKYIDQEELN	IDQEELNKTKPIWTRN	K	Y	I	N	K	[illegible]
YIFHNFMER	EAEFQSPKYIFHNFME	IFHNFMERLWAYLTIQ	K	Y	I	E	R	[illegible]
YIFSDDSSQLTIK	EQEEDDEKYIFSDDSS	DSSQLTIKKVDKNDEA	K	Y	I	I	K	[illegible]
YIILLLGK	FEVVESGRYIILLLGK	YIILLLGKALSVVWDR	R	Y	I	G	K	[illegible]
YILQGVTSWGLGCAR	LVCFEKDKYILQGVTS	SWGLGCARPNKPGVYV	K	Y	I	A	R	[illegible]

© 2020 Mongan D et al. *JAMA Psychiatry*.

YIPPCLDSELTEFPLR	LDYIGPCKYIPPCLDS	ELTEFPLRMRDWLKNV	K	Y	I	L	R	M	0	1	0	1	1	0	2	0	0	1	3	0	0	1	3	1	1	0	1	0	0	0	16	0	1948.96	sp|P09486	sp|P09486|SP	151	166	yes	yes	2;3	7.60E-06	108.56	3986		240	4069	634822;6348	297431;29	297432		7
YISPDQLADLYK	KSPDDPSRYISPDQLA	DQLADLYKSFIKDYPV	R	Y	I	Y	K	S	1	0	0	2	0	1	0	0	0	1	2	1	0	0	1	1	0	0	2	0	0	0	12	0	1424.719	sp|P06733	sp|P06733|EN	270	281	yes	yes	2	1.00E-20	140.16	3987		208	4070	634901;6349	297438;29	297509		75
YKEENDDFASFR	QANKALEKYKEENDDF	NDDFASFRVDRIERVA	K	Y	K	F	R	V	1	1	1	2	0	0	2	0	0	0	0	1	0	2	0	1	0	0	1	0	0	0	12	1	1519.658	sp|P04196	sp|P04196|HR	165	176	yes	yes	2;3	1.33E-218	251.43	3989		176	4072	635013;6350	297516;29	297517		202
YLAEFATGNDR	KMKGDYHRYLAEFATG	EFATGNDRKEAAENSL	R	Y	L	D	R	K	2	1	1	1	0	0	1	1	0	0	1	0	0	1	0	0	1	0	1	0	0	0	11	0	1255.583	sp|P62258	sp|P62258|14	131	141	yes	yes	2	0.0005498	100.25	3990		404	4073	635336	297718	297718		1
YLAEVAAGDDK	KMKGDYYRYLAEVAAG	EVAAGDDKKGIVDQSQ	R	Y	L	D	K	K	3	0	0	2	0	0	1	1	0	0	1	1	0	0	0	0	0	0	1	1	0	0	11	0	1150.551	sp|P63104	sp|P63104|14	128	138	yes	yes	2	8.44E-06	109.66	3991		411	4074	635337;6353	297719;29	297757		40
YLAEVATGEK	KMKGDYYRYLAEVATG	AEVATGEKRATVVESS	R	Y	L	E	K	R	2	0	0	0	0	0	2	1	0	0	1	1	0	0	0	0	1	0	1	1	0	0	10	0	1079.55	sp|P61981	sp|P61981|14	133	142	yes	yes	2	0.022427	61.65	3992		402	4075	635457;6354	297760;29	297760		2
YLFLNGNK	LLPQPDLRYLFLNGNK	YLFLNGNKLARVAAGA	R	Y	L	N	K	L	0	0	2	0	0	0	0	1	0	0	2	1	0	1	0	0	0	0	1	0	0	0	8	0	967.5127	sp|P02750	sp|P02750|A2	240	247	yes	yes	2	0.0008949	119.22	3994		152	4077	635522;6355	297765;29	297766		24
YLGPQYVAGITNLK	HGKTTYEKYLGPQYVA	VAGITNLKKCSTSPLL	K	Y	L	L	K	K	1	0	1	0	0	1	0	2	0	1	2	1	0	0	1	0	1	0	2	1	0	0	14	0	1535.835	sp|P02788	sp|P02788|TR	681	694	yes	yes	2	0.0067559	66.289	3995		163	4078	635633;6356	297789	297789		1
YLGQDYEQLR	GRHENAIKYLGQDYEQ	GQDYEQLRVRCLQSGT	K	Y	L	L	R	V	0	1	0	1	0	2	1	1	0	0	2	0	0	0	0	0	0	0	2	0	0	0	10	0	1283.615	sp|P07384	sp|P07384|CA	37	46	yes	yes	2	4.27E-08	134.48	3996		219	4079	635655;6356	297790;29	297792		16
YLPCSVLR	HHWYLHDRYLPCSVLR	YLPCSVLRMDYKGDAT	R	Y	L	L	R	M	0	1	0	0	1	0	0	0	0	0	2	0	0	0	1	1	0	0	1	1	0	0	8	0	1006.527	sp|P29622	sp|P29622|KA	260	267	yes	yes	2	0.013083	107.67	3998		336	4081	635799;6358	297812;29	297814		45
YLQEIYNSNNQK	LTHDSSIRYLQEIYNS	IYNSNNQKIVNLKEKV	R	Y	L	Q	K	I	0	0	3	0	0	2	1	0	0	1	1	1	0	0	0	1	0	0	2	0	0	0	12	0	1512.721	sp|P02679	sp|P02679|FIB	135	146	yes	yes	2;3	2.15E-119	218.55	3999		144	4082	635967;6359	297857;29	297999		173
YLQGSSVQLR	SLRVGLQRYLQGSSVQ	QGSSVQLRSLRLTYRN	R	Y	L	L	R	S	0	1	0	0	0	2	0	1	0	0	2	0	0	0	0	2	0	0	1	1	0	0	10	0	1149.614	sp|Q6EMK	sp|Q6EMK4|V	483	492	yes	yes	2	2.89E-31	174.16	4000		485	4083	636313;6363	298030;29	298036		101
YLSDHSFLVSQGDR	TNTGLALRYLSDHSFL	FLVSQGDREQAPNLVY	R	Y	L	D	R	E	0	1	0	2	0	1	0	1	1	0	2	0	0	1	0	3	0	0	1	1	0	0	14	0	1622.769	sp|P04275	sp|P04275|VW	1584	1597	yes	yes	3	5.66E-39	116.98	4001		179	4084	636486;6364	298131;29	298131		7
YLSVQALEAPR	HDKHCHKKYLSVQALE	VQALEAPRLTQNLTDL	K	Y	L	P	R	L	2	1	0	0	0	1	1	0	0	0	2	0	0	0	1	1	0	0	1	1	0	0	11	0	1245.672	sp|P35916	sp|P35916|VG	669	679	yes	yes	2	0.0084373	91.584	4002		352	4085	636547;6365	298138;29	298139		1
YLSYTLNPDLIR	KELWILNRYLSYTLNP	TLNPDLIRKQDATSTI	R	Y	L	I	R	K	0	1	1	1	0	0	0	0	0	1	3	0	0	0	1	1	1	0	2	0	0	0	12	0	1466.777	sp|P15144	sp|P15144|AM	844	855	yes	yes	2	4.67E-19	131.44	4003		285	4086	636604;6366	298140;29	298160		37
YLTLNTESTR	VFKSTEQKYLTLNTES	TLNTESTRIMTKLRAQ	K	Y	L	T	R	I	0	1	1	0	0	0	1	0	0	0	2	0	0	0	0	1	3	0	1	0	0	0	10	0	1196.604	sp|P06276	sp|P06276|CH	528	537	yes	yes	2	8.62E-62	234.82	4004		202	4087	636685;6366	298179;29	298184		116
YLTWASR	SQELPREKYLTWASRQ	KYLTWASRQEPSQGTT	K	Y	L	S	R	Q	1	1	0	0	0	0	0	0	0	0	1	0	0	0	0	1	1	1	1	0	0	0	7	0	895.4552	sp|P01876	sp|P01876|IG	276	282	no	no	2	0.000646	139.27	4005	129;130		4088	636847;6368	298295;29	298302		50
YLYHGQTLETLGGK	IKDQLASKYLYHGQTL	TLETLGGKKLRVFVYR	K	Y	L	G	K	K	0	0	0	0	0	1	1	3	1	0	3	1	0	0	0	0	2	0	2	0	0	0	14	0	1578.804	sp|Q15582	sp|Q15582|BG	448	461	yes	yes	3	3.19E-09	94.287	4007		470	4090	637361;6373	298459;29	298459		4
YNALDLTNNGK	TTLNSDLKYNALDLTN	LDLTNNGKLRLEPLKL	K	Y	N	G	K	L	1	0	3	1	0	0	0	1	0	0	2	1	0	0	0	0	1	0	1	0	0	0	11	0	1221.599	sp|P04114	sp|P04114|AP	1803	1813	yes	yes	2	6.13E-95	209.58	4008		174	4091	637393;6373	298463;29	298556		109
YNILPEKEDSPFALK	VYLQTSMKYNILPEKE	EDSPFALKVQTVPQTC	K	Y	N	L	K	V	1	0	1	1	0	0	2	0	0	1	2	2	0	1	2	1	0	0	1	0	0	0	15	1	1762.914	sp|P20742	sp|P20742|PZ	1336	1350	yes	yes	3	2.52E-13	82.925	4009		307	4092	637529;6375	298572;29	298572		6
YNILPEKEEFPFALGVQTLPQTCDEPK	VYLQTSLKYNILPEKE	PQTCDEPKAHTSFQIS	K	Y	N	P	K	A	1	0	1	1	1	2	4	1	0	1	3	2	0	2	4	0	2	0	1	1	0	0	27	1	3162.558	sp|P01023	sp|P01023|A2	1330	1356	yes	yes	3;4	3.56E-37	100.27	4010		96	4093	637566;6375	298578;29	298649		83
YNPVVIDFEMQPIHEVLR	RSWGRSLKYNPVVIDF	QPIHEVLRHTSLGPLE	K	Y	N	L	R	H	0	1	1	1	0	1	2	0	1	2	1	0	1	1	2	0	0	0	1	3	0	0	18	0	2198.119	sp|P07357	sp|P07357|CO	452	469	yes	yes	2;3	1.34E-30	117.2	4011		215	4094	637734;6377	298662;29	298662		74
YNSQNQSNNQFVLYR	AVDAALKKYNSQNQSN	NNQFVLYRITEATKTV	K	Y	N	Y	R	I	0	1	4	0	0	3	0	0	0	0	1	0	0	1	0	2	0	0	2	1	0	0	15	0	1873.871	sp|P01042	sp|P01042|KN	44	58	yes	yes	2;3	0	300.35	4012		103	4095	637919;6379	298736;29	298754		268
YPIEHGIITNWDDMEK	KRGILTLKYPIEHGII	TNWDDMEKIWHHSFYN	K	Y	P	E	K	I	0	0	1	2	0	0	2	1	1	3	0	1	1	0	1	0	1	1	1	0	0	0	16	0	1959.904	sp|P63267	sp|P63267|AC	70	85	yes	no	3	3.74E-05	68.257	4013		406	4096	638271;6382	299004;29	299004		17
YPLYVLK	HIGSSFEKYPLYVLKV	KYPLYVLKVSGKEQAA	K	Y	P	L	K	V	0	0	0	0	0	0	0	0	0	0	2	1	0	0	1	0	0	0	2	1	0	0	7	0	894.5215	sp|Q96IY4	sp|Q96IY4|CB	156	162	yes	no	2	0.051214	84.025	4014		516	4097	638392;6383	299021;29	299021		2
YPPTVSMVEGQGEK	LCTPLLPKYPPTVSMV	MVEGQGEKNVTFWGRP	K	Y	P	E	K	N	0	0	0	0	0	1	2	2	0	0	0	1	1	0	2	1	1	0	1	2	0	0	14	0	1520.718	sp|P27918	sp|P27918|PR	414	427	yes	yes	2	0.0019852	64.64	4015		335	4098	638469;6384	299023;29	299023		0
YPSLSIHGIEGAFDEPGTK	EILMHLWRYPSLSIHG	AFDEPGTKTVIPGRVI	R	Y	P	T	K	T	1	0	0	1	0	0	2	3	1	2	1	1	0	1	2	2	1	0	1	0	0	0	19	0	2016.979	sp|Q96KN	sp|Q96KN2|C	344	362	yes	yes	3	6.98E-05	59.372	4016		517	4099	638491;6384	299025;29	299026		3
YPVYGVQWHPEK	ISTMEGYKYPVYGVQW	GVQWHPEKAPYEWKNL	K	Y	P	E	K	A	0	0	0	0	0	1	1	1	1	0	0	1	0	0	2	0	0	1	2	2	0	0	12	0	1501.735	sp|Q9282	sp|Q92820|GG	236	247	yes	yes	2;3	2.23E-05	68.676	4017		510	4100	638540;6385	299028;29	299028		4
YQCYCYGR	EKYVHGVRYQCYCYGR	YQCYCYGRGIGEWHCQ	R	Y	Q	G	R	G	0	1	0	0	2	1	0	1	0	0	0	0	0	0	0	0	0	0	3	0	0	0	8	0	1168.443	sp|P02751	sp|P02751|FIN	585	592	yes	yes	2	5.61E-22	170.94	4018		153	4101	638646;6386	299032;29	299040		39
YQISVNK	TVQNEANKYQISVNKY	KYQISVNKYRGTAGNA	K	Y	Q	N	K	Y	0	0	1	0	0	1	0	0	0	1	0	1	0	0	0	1	0	0	1	1	0	0	7	0	850.4549	sp|P02675	sp|P02675|FIB	368	374	yes	yes	2	0.0050988	132.72	4019		143	4102	638832;6388	299071;29	299075		7
YQLAVTQR	ARGFSWERYQLAVTQR	YQLAVTQRKEEEPSSS	R	Y	Q	Q	R	K	1	1	0	0	0	2	0	0	0	0	1	0	0	0	0	0	1	0	1	1	0	0	8	0	977.5294	sp|Q16853	sp|Q16853|AC	630	637	yes	yes	2	0.010703	112.37	4020		478	4103	638918;6389	299078;29	299082		5
YSAWAESVTNLPQVIK	NPAGNKRRYSAWAESV	TNLPQVIKQKLTPLYE	R	Y	S	I	K	Q	2	0	1	0	0	1	1	0	0	1	1	1	0	0	1	2	1	1	1	2	0	0	16	0	1804.936	sp|P10643	sp|P10643|CO	403	418	yes	yes	2;3	1.06E-96	179.94	4021		252	4104	639004;6390	299083;29	299342		286
YSDASDCHGEDSQAFCEK	VTVSICRKYSDASDCH	DSQAFCEKFSGQLNSH	K	Y	S	E	K	F	2	0	0	3	2	1	2	1	1	0	0	1	0	1	0	3	0	0	1	0	0	0	18	0	2104.774	sp|P01023	sp|P01023|A2	272	289	yes	yes	2;3	1.36E-215	231.17	4022		96	4105	639456;6394	299370;29	299376		19
YSFCTDHTVLVQTR	SNYEQDQKYSFCTDHT	HTVLVQTRGGNSNGAL	K	Y	S	T	R	G	0	1	0	1	1	1	0	0	1	0	1	0	0	1	0	1	3	0	1	2	0	0	14	0	1725.815	sp|P02751	sp|P02751|FIN	398	411	yes	yes	2;3	0	316.34	4023		153	4106	639555;6395	299389;29	299493		109
YSFTIELR	WIYDLGIKYSFTIELR	YSFTIELRDTGTYGFL	K	Y	S	L	R	D	0	1	0	0	0	0	1	0	0	1	1	0	0	1	0	1	1	0	1	0	0	0	8	0	1027.534	sp|Q96IY4	sp|Q96IY4|CB	380	387	yes	yes	2	2.51E-10	158.36	4024		516	4107	639922;6399	299498;29	299580		100
YSLEPVAVELK	DGVPMPDKYSLEPVAV	EPVAVELKSLLGKDVL	K	Y	S	L	K	S	1	0	0	0	0	0	2	0	0	0	2	1	0	0	1	1	0	0	1	2	0	0	11	0	1246.681	sp|P00558	sp|P00558|PG	76	86	yes	yes	2	0.0001096	96.143	4025		78	4108	640304;6403	299598;29	299598		10
YSLTYIYTGLSK	PQENQDGRYSLTYIYT	YIYTGLSKHVEDVPAF	R	Y	S	S	K	H	0	0	0	0	0	0	0	1	0	1	2	1	0	0	0	2	2	0	3	0	0	0	12	0	1407.729	sp|P25311	sp|P25311|ZA	28	39	yes	yes	2	2.15E-19	157.78	4026		326	4109	640371;6403	299608;29	299754		187
YSPGGTPTAIK	YRNNCLLKYSPGGTPT	GGTPTAIKVLSNVESG	K	Y	S	I	K	V	1	0	0	0	0	0	0	2	0	1	0	1	0	0	2	1	2	0	1	0	0	0	11	0	1090.566	sp|P03952	sp|P03952|KL	170	180	yes	yes	2	0.001259	81.95	4027		166	4110	640594;6405	299795;29	299802		24
YSSDYFQAPSDYR	SRRGPLVKYSSDYFQA	FQAPSDYRYYPYQSFQ	K	Y	S	Y	R	Y	1	1	0	2	0	1	0	0	0	0	0	0	0	1	1	3	0	0	3	0	0	0	13	0	1597.669	sp|Q08380	sp|Q08380|LG	442	454	yes	yes	2	1.59E-16	138.66	4028		440	4111	640735;6407	299819;29	299825		4
YSSLAEAASK	VAHWNSAKYSSLAEAA	SLAEAASKADGLAVIG	K	Y	S	S	K	A	3	0	0	0	0	0	1	0	0	0	1	1	0	0	0	3	0	0	1	0	0	0	10	0	1025.503	sp|P00915	sp|P00915|CA	129	138	yes	yes	2	0.0038886	81.95	4029		90	4112	640812;6408	299826;29	299827		12
YTACETAR	ATDVFWAKYTACETAR	YTACETARTPRDKLAA	K	Y	T	A	R	T	2	1	0	0	1	0	1	0	0	0	0	0	0	0	0	0	2	0	1	0	0	0	8	0	970.4178	sp|P00734	sp|P00734|TH	87	94	yes	yes	2	0.020285	98.299	4030		80	4113	640918;6409	299838;29	299839		3
YTAGPYECEIR	LFLLGVTKYTAGPYEC	GPYECEIRNPVSASRS	K	Y	T	I	R	N	1	1	0	0	1	0	2	1	0	1	0	0	0	0	1	0	1	0	2	0	0	0	11	0	1357.597	sp|P11464	sp|P11464|PS	210	220	no	no	2	0.0001353	94.309	4031		260	4114	640967	299841	299841		1
YTCLPGYVR	FKTGTTLKYTCLPGYV	TCLPGYVRSHSTQTLT	K	Y	T	V	R	S	0	1	0	0	1	0	0	1	0	0	1	0	0	0	1	0	1	0	2	1	0	0	9	0	1127.543	sp|P04003	sp|P04003|C4	79	87	yes	yes	2	4.35E-09	148.33	4032		167	4115	640968;6409	299842;29	299868		104
YTFEIFDGK	PTEKDKGKYTFEIFDG	TFEIFDGKDNHQRSLD	K	Y	T	G	K	D	0	0	0	1	0	0	1	1	0	1	0	1	0	2	0	0	1	0	1	0	0	0	9	0	1118.528	sp|P54296	sp|P54296|MY	1301	1309	yes	yes	2	0.054618	56.434	4033		385	4116	641141;6411	299946	299946		1
YTFELSR	GNTKVMDKYTFELSRR	KYTFELSRRTHLPEVF	K	Y	T	S	R	R	0	1	0	0	0	0	1	0	0	0	1	0	0	1	0	1	1	0	1	0	0	0	7	0	914.4498	sp|P02774	sp|P02774|VT	346	352	yes	yes	2;3	2.50E-13	163.84	4034		159	4117	641148;6411	299947;29	299991		262
YTFVVPEDTR	FPFFTQTKYTFVVPED	FVVPEDTRVGTSVGSL	K	Y	T	T	R	V	0	1	0	1	0	0	1	0	0	0	0	0	0	1	1	0	2	0	1	2	0	0	10	0	1225.598	sp|P33151	sp|P33151|CA	263	272	yes	yes	2	4.90E-11	143.7	4035		344	4118	642154;6421	300210;30	300278		82
YTGNASALFILPDQDK	SCTVVELKYTGNASAL	FILPDQDKMEEVEAML	K	Y	T	D	K	M	2	0	1	2	0	1	0	1	0	1	2	1	0	1	1	1	1	0	1	0	0	0	16	0	1751.873	sp|P01011	sp|P01011|AA	268	283	yes	yes	2	0.0001382	98.407	4036		94	4119	642273;6422	300293;30	300293		3
YTGNTYR	AEETCFDKYTGNTYRV	KYTGNTYRVGDTYERP	K	Y	T	Y	R	V	0	1	1	0	0	0	0	1	0	0	0	0	0	0	0	0	2	0	2	0	0	0	7	0	873.3981	sp|P02751	sp|P02751|FIN	101	107	yes	yes	2	0.048467	85.359	4037		153	4120	642286;6422	300296	300296		1
YTHGHGSETLYLAPGGGDDWIYDLGIK	EKISKNTRYTHGHGSE	WIYDLGIKYSFTIELR	R	Y	T	I	K	Y	1	0	0	3	0	0	1	6	2	2	3	1	0	0	1	1	2	1	3	0	0	0	27	0	2934.382	sp|Q96IY4	sp|Q96IY4|CB	353	379	yes	yes	4	0.0025637	23.89	4038		516	4121	642305;6423	300297	300297		1
YTIAALLSPYSYSTTAVVTNPK	ANDSGPRRYTIAALLS	TAVVTNPKE________	R	Y	T	P	K	E	3	0	1	0	0	0	0	0	0	1	2	1	0	0	2	3	4	0	3	2	0	0	22	0	2359.231	sp|P02766	sp|P02766|TT	125	146	yes	yes	2;3	8.16E-05	85.006	4039		158	4122	642309;6423	300298;30	300299		2
YTIAALLSPYSYSTTAVVTNPKE	ANDSGPRRYTIAALLS	AVVTNPKE________	R	Y	T	K	E	-	3	0	1	0	0	0	1	0	0	1	2	1	0	0	2	3	4	0	3	2	0	0	23	1	2488.274	sp|P02766	sp|P02766|TT	125	147	yes	yes	3	0.0007801	33.342	4040		158	4123	642325;6423	300300;30	300301		2
YTLFQIFSK	ASTSEVLKYTLFQIFS	TLFQIFSKIDRPEASR	K	Y	T	S	K	I	0	0	0	0	0	1	0	0	0	1	1	1	0	2	0	1	1	0	1	0	0	0	9	0	1145.612	sp|P04275	sp|P04275|VW	1363	1371	yes	yes	2	0.0047962	90.709	4041		179	4124	642328;6423	300302;30	300302		2
YTPVQQGPVGVNVTYGGDPIPK	HDNTYTVKYTPVQQGP	YGGDPIPKSPFSVAVS	K	Y	T	P	K	S	0	0	1	1	0	2	0	4	0	1	0	1	0	0	4	0	2	0	2	4	0	0	22	0	2285.169	sp|P21333	sp|P21333|FL	937	958	yes	yes	2;3	3.10E-15	84.842	4042		310	4125	642351;6423	300304;30	300308		8
YTSLMLR	CSMDDTVRYTSLMLRD	RYTSLMLRDYSGQGVV	R	Y	T	L	R	D	0	1	0	0	0	0	0	0	0	0	2	0	1	0	0	1	1	0	1	0	0	0	7	0	882.4633	sp|O75083	sp|O75083|W	390	396	yes	yes	2	0.03407	92.191	4043		59	4126	642433;6424	300312	300312		1
YTTTMGVNTYK	NLPNGDFRYTTTMGVN	TMGVNTYKARIQYYCH	R	Y	T	Y	K	A	0	0	1	0	0	0	0	1	0	0	0	1	1	0	0	0	4	0	2	1	0	0	11	0	1277.596	sp|P00736	sp|P00736|C1	389	399	yes	yes	2	2.52E-110	218.48	4044		81	4127	642499;6425	300313;30	300328		88
YTVNQCR	YTGGMKQKYTVNQCRR	KYTVNQCRRQSEDSTF	K	Y	T	C	R	R	0	1	1	0	1	1	0	0	0	0	0	0	0	0	0	0	1	0	1	1	0	0	7	0	939.4233	sp|P00450	sp|P00450|CE	713	719	yes	yes	2	1.22E-08	159.65	4045		74	4128	642665;6426	300401;30	300420		22
YTYNYEAESSSGVPGTADSR	TRFKHLRKYTYNYEAE	VPGTADSRSATRINCK	K	Y	T	S	R	S	2	1	1	1	0	0	2	2	0	0	0	0	0	0	1	4	2	0	3	1	0	0	20	0	2152.919	sp|P04114	sp|P04114|AP	52	71	yes	yes	2;3	6.88E-152	239.75	4046		174	4129	642809;6428	300423;30	300429		139
YVDQVLQLVYK	GKANKRLRYVDQVLQL	QVLQLVYKDGSPCPSK	R	Y	V	Y	K	D	0	0	0	1	0	2	0	0	0	0	2	1	0	0	0	0	0	0	2	3	0	0	11	0	1366.75	sp|P11717	sp|P11717|MP	1583	1593	yes	yes	2	0.0361	64.711	4047		261	4130	643069;6430	300562	300562		1
YVLSPYK	EAEIPGIKYVLSPYKL	KYVLSPYKLNLVATPL	K	Y	V	Y	K	L	0	0	0	0	0	0	0	0	0	0	1	1	0	0	1	1	0	0	2	1	0	0	7	0	868.4695	sp|P01031	sp|P01031|CO	347	353	yes	no	2	0.039591	89.673	4049		98	4132	643654;6436	300761;30	300769		14
YVLSQPPSVSVAPGQTAR	________________	VAPGQTARITCGGDGI	-	Y	V	A	R	I	2	1	0	0	0	2	0	1	0	0	1	0	0	0	3	3	1	0	1	3	0	0	18	0	1855.979	sp|P01719	sp|P01719|LV	1	18	yes	yes	2	0.0005628	58.674	4050		117	4133	643726	300775	300775		1
YVLTNLNIGAELLR	AHQEDTERYVLTNLNI	NIGAELLRDPSLGAQF	R	Y	V	L	R	D	1	1	2	0	0	0	1	1	0	1	4	0	0	0	0	0	1	0	1	1	0	0	14	0	1587.898	sp|Q76LX8	sp|Q76LX8|AT	103	116	yes	yes	2	5.41E-06	88.596	4051		491	4134	643727;6437	300776;30	300777		5
YVMLPVADQDQCIR	FKFTDHLKYVMLPVAD	ADQDQCIRHYEGSTVP	K	Y	V	I	R	H	1	1	0	2	1	2	0	0	0	1	1	0	1	0	1	0	0	0	1	2	0	0	14	0	1706.812	sp|P00738	sp|P00738|HP	298	311	yes	yes	2;3	1.69E-110	201.33	4052		82	4135;4136	643771;6437	300781;30	300905	17	183
YVMLPVADQYDCITHYEGSTCPK	FKLTDHLKYVMLPVAD	YEGSTCPKWKAPKSPV	K	Y	V	P	K	W	1	0	0	2	2	1	1	1	1	1	1	1	1	0	2	1	2	0	3	2	0	0	23	0	2746.208	sp|P00739	sp|P00739|HP	240	262	yes	yes	3	5.18E-06	64.295	4053		83	4137	644021	300967	300967		1
YVSHFETEGPHVLLYFDSVPTSR	KLTSLSDRYVSHFETE	FDSVPTSRECVGFEAV	R	Y	V	S	R	E	0	1	0	1	0	0	2	1	2	0	2	0	0	2	2	3	2	0	2	3	0	0	23	0	2679.297	sp|P0C0L4	sp|P0C0L4|CO	1511	1533	no	no	4	4.14E-12	77.847	4054	245;246		4138	644022;6440	300968;30	300986		45
YVTSAPMPEPQAPGR	GQPLSPEKYVTSAPMP	PEPQAPGRYFAHSILT	K	Y	V	G	R	Y	2	1	0	0	0	1	1	1	0	0	0	0	1	0	4	1	1	0	1	1	0	0	15	0	1599.772	sp|P01871	sp|P01871|IG	377	391	yes	no	2	4.93E-13	100.55	4055		128	4139	644131;6441	301013;30	301027		26
YVVIAELLAHK	INISPDGKYVVIAELL	IAELLAHKIHVYEKHA	K	Y	V	H	K	I	2	0	0	0	0	0	1	0	1	1	2	1	0	0	0	0	0	0	2	1	0	0	11	0	1318.729	sp|P27169	sp|P27169|PO	234	244	yes	yes	2;3	4.14E-05	76.143	4056		332	4140	644214;6442	301039;30	301039		38
YWGVASFLQK	DPAKFKMKYWGVASFL	GVASFLQKGNDDHWIV	K	Y	W	Q	K	G	1	0	0	0	0	1	0	1	0	0	1	1	0	1	0	1	0	1	1	1	0	0	10	0	1197.618	sp|P02753	sp|P02753|RE	108	117	yes	yes	1;2	1.84E-15	152.63	4057		154	4141	644394;6443	301077;30	301178		213
YWNDCEPPDSR	DCSVLSRKYWNDCEPP	DCEPPDSRRPSEIVIG	K	Y	W	S	R	R	0	1	1	2	1	0	1	0	0	0	0	0	0	0	2	1	0	1	1	0	0	0	11	0	1437.562	sp|P04196	sp|P04196|HR	85	95	yes	yes	2	7.32E-16	144.77	4058		176	4142	644955;6449	301292;30	301427		174
YYCFQGNQFLR	SALRWLGRYYCFQGNQ	FQGNQFLRFDPVRGEV	R	Y	Y	L	R	F	0	1	1	0	1	2	0	1	0	0	1	0	0	2	0	0	0	0	2	0	0	0	11	0	1494.671	sp|P02790	sp|P02790|HE	198	208	yes	yes	2;3	4.22E-203	213.35	4059		164	4143	645152;6451	301466;30	301696		944
YYELEEK	SIVGWTVKYYELEEKI	KYYELEEKIVSLIKNL	K	Y	Y	E	K	I	0	0	0	0	0	0	3	0	0	0	1	1	0	0	0	0	0	0	2	0	0	0	7	0	972.444	sp|P04114	sp|P04114|AP	4400	4406	yes	yes	2	0.009715	108.24	4060		174	4144	647190;6471	302456;30	302469		24
YYGYTGAFR	CVPNSNERYYGYTGAF	YGYTGAFRCLAENAGD	R	Y	Y	F	R	C	1	1	0	0	0	0	0	2	0	0	0	0	0	1	0	0	1	0	3	0	0	0	9	0	1096.498	sp|P02788	sp|P02788|TR	544	552	yes	no	2	0.0007316	112.17	4061		163	4145	647335;6473	302480;30	302490		12
YYLQGAK	HSGSTFFKYYLQGAKI	KYYLQGAKIPKPEASF	K	Y	Y	A	K	I	1	0	0	0	0	1	0	1	0	0	1	1	0	0	0	0	0	0	2	0	0	0	7	0	841.4334	sp|Q14624	sp|Q14624|IT	627	633	yes	yes	2	0.051214	84.025	4062		463	4146	647425;6474	302492	302492		1
YYLQICTLLK	GLHTVEGRYYLQICTL	LQICTLLKCKTTNLNT	R	Y	Y	L	K	C	0	0	0	0	1	1	0	0	0	1	3	1	0	0	0	0	1	0	2	0	0	0	10	0	1313.705	sp|O95497	sp|O95497|VN	393	402	yes	yes	2	0.014305	68.735	4063		68	4147	647472;6474	302493	302493		1
YYPLGEVFYPGPECER	CGCLHDGRYYPLGEVF	YPGPECERRCECGPGG	R	Y	Y	E	R	R	0	1	0	0	1	0	3	2	0	0	1	0	0	1	3	0	0	0	3	1	0	0	16	0	1974.882	sp|Q9Y6R7	sp|Q9Y6R7|FC	4800	4815	yes	yes	2	4.63E-05	102.62	4064		559	4148	647489;6474	302494;30	302495		7
YYPLGQTFYPGPGCDSLCR	CGCLHDDRYYPLGQTF	PGCDSLCRCREGGEVS	R	Y	Y	C	R	C	0	1	0	1	2	1	0	3	0	0	2	0	0	1	3	1	1	0	3	0	0	0	19	0	2249.987	sp|Q9Y6R7	sp|Q9Y6R7|FC	2398	2416	yes	no	2	0.0002917	62.042	4065		559	4149	647542;6475	302501;30	302501		8
YYQENFCEQICSK	HRQIVVDKYYQENFCE	FCEQICSKQETRECNW	K	Y	Y	S	K	Q	0	0	1	0	2	2	2	0	0	1	0	1	0	1	0	1	0	0	2	0	0	0	13	0	1767.723	sp|P13671	sp|P13671|CO	55	67	yes	yes	2	3.19E-229	255.7	4066		277	4150	647634;6476	302509;30	302518		131
YYSFFDLDPK	DALWLRFKYYSFFDLD	SFFDLDPKTDPVRLTQ	K	Y	Y	P	K	T	0	0	0	2	0	0	0	0	0	0	1	1	0	2	1	1	0	0	2	0	0	0	10	0	1293.592	sp|Q86UX	sp|Q86UX7|U	253	262	yes	yes	2	0.01032	72.643	4067		497	4151	647795;6477	302640;30	302641		8
YYTAINFVATPDEQNR	SLSQEDDRYYTAINFV	ATPDEQNRDLDMFINA	R	Y	Y	N	R	D	2	1	2	1	0	1	1	0	0	1	0	0	0	1	1	0	2	0	2	1	0	0	16	0	1900.896	sp|O75882	sp|O75882|AT	1176	1191	yes	yes	2	1.60E-122	197.3	4068		64	4152	647834;6478	302648;30	302666		49
YYTYLIMNK	RAHEAKIRYYTYLIMN	YTYLIMNKGRLLKAGR	R	Y	Y	N	K	G	0	0	1	0	0	0	0	0	0	1	1	1	1	0	0	0	1	0	3	0	0	0	9	0	1207.595	sp|P01024	sp|P01024|CO	489	497	yes	yes	2	0.0007733	111.04	4069		97	4153	647901;6479	302697;30	302712		74
YYWGGQYTWDMAK	HAANPNGRYYWGGQYT	QYTWDMAKHGTDDGVV	R	Y	Y	A	K	H	1	0	0	1	0	1	0	2	0	0	0	1	1	0	0	0	1	2	3	0	0	0	13	0	1667.708	sp|P02675	sp|P02675|FIB	446	458	yes	yes	2	5.58E-16	121.6	4070		143	4154	648030;6480	302771;30	302863		109

© 2020 Mongan D et al. *JAMA Psychiatry*.
